# Supplementary material for: Meta-review of the barriers and facilitators to women accessing perinatal mental healthcare
Source: BMJ Open. 2023 Jul 20;13(7):e066703. doi: 10.1136/bmjopen-2022-066703 (PMC10360426; doi:10.1136/bmjopen-2022-066703)
Supplement: Supplementary data [file bmjopen-2022-066703supp001.pdf]

A meta-review of the barriers to women accessing perinatal mental health care

Appendices

Contents

Appendix 1: PROSPERO Registration: CRD42019142854 2

Appendix 2: Table 1. Search terms 14

Appendix 3: Table 2. Search Results 28

Appendix 4: Table 3. Inclusion and exclusion criteria 29

Appendix 5: Detailed methodology 30

Appendix 6: Table 4. Sensitivity analysis 33

Appendix 7: *Figure 1.* Synthesis contribution vs quality appraisal criteria met 35

Appendix 8: Sensitivity analysis results 36

Appendix 9: Table 5. Summary review characteristics 37

Appendix 10: Table 6. Characteristics of included reviews 37

Appendix 11: Table 7. Quality appraisal 48

Appendix 12: Table 8. Themes 51

References 56

Appendix 1: PROSPERO Registration: CRD42019142854

PROSPERO
International prospective register of systematic reviews

NHS
National Institute for Health Research

UNIVERSITY of York
Centre for Reviews and Dissemination

Systematic review

1. * Review title.

Give the title of the review in English

Meta-review of barriers to women accessing perinatal mental healthcare and treatment

2. Original language title.

For reviews in languages other than English, give the title in the original language. This will be displayed with the English language title.

3. * Anticipated or actual start date.

Give the date the systematic review started or is expected to start.

07/06/2020

4. * Anticipated completion date.

Give the date by which the review is expected to be completed.

28/02/2021

5. * Stage of review at time of this submission.

Tick the boxes to show which review tasks have been started and which have been completed. Update this field each time any amendments are made to a published record.

Reviews that have started data extraction (at the time of initial submission) are not eligible for inclusion in PROSPERO. If there is later evidence that incorrect status and/or completion date has been supplied, the published PROSPERO record will be marked as retracted.

This field uses answers to initial screening questions. It cannot be edited until after registration.

The review has not yet started: No

Review stage	Started	Completed
Preliminary searches	Yes	No
Piloting of the study selection process	Yes	No
Formal screening of search results against eligibility criteria	No	No
Data extraction	No	No
Risk of bias (quality) assessment	No	No
Data analysis	No	No

Provide any other relevant information about the stage of the review here.

6. * Named contact.

The named contact is the guarantor for the accuracy of the information in the register record. This may be any member of the review team.

Rebecca Webb

Email salutation (e.g. "Dr Smith" or "Joanne") for correspondence:

Dr Webb

7. * Named contact email.

Give the electronic email address of the named contact.

Rebecca.Webb.2@city.ac.uk

8. Named contact address

Give the full institutional/organisational postal address for the named contact.

Centre for Maternal and Child Health Research, School of Health Sciences, City, University of London, EC1V 0HB

9. Named contact phone number.

Give the telephone number for the named contact, including international dialling code.

(+44)07810255328

10. * Organisational affiliation of the review.

Full title of the organisational affiliations for this review and website address if available. This field may be completed as 'None' if the review is not affiliated to any organisation.

City, University of London

Organisation web address:

11. * Review team members and their organisational affiliations.

Give the personal details and the organisational affiliations of each member of the review team. Affiliation refers to groups or organisations to which review team members belong. **NOTE: email and country now MUST be entered for each person, unless you are amending a published record.**

Dr Rebecca Webb. City, University of London
Dr Elizabeth Ford. Brighton and Sussex Medical School
Dr Judy Shakespeare. Retired GP
Dr Abigail Easter. King's College London
Professor Simon Gilbody. University of York
Professor Fiona Alderdice. University of Oxford
Dr Nia Roberts. Nuffield Department of Population Health, Bodleian Health Care Libraries
Professor Debra Salmon. City, University of London

Professor Helen Cheyne. University of Stirling
Ms Clare Thompson. Maternal Mental Health Change Agents
Miss Nazihah Uddin. City, University of London
Professor Susan Ayers. City, University of London

12. * Funding sources/sponsors.

Details of the individuals, organizations, groups, companies or other legal entities who have funded or sponsored the review.

NIHR Health Services and Delivery Research Grant

Grant number(s)

State the funder, grant or award number and the date of award

NIHR128068

13. * Conflicts of interest.

List actual or perceived conflicts of interest (financial or academic).

None

14. Collaborators.

Give the name and affiliation of any individuals or organisations who are working on the review but who are not listed as review team members. **NOTE: email and country must be completed for each person, unless you are amending a published record.**

Dr Agnes Hann. National Childbirth Trust
Dr Sarah McMullen. National Childbirth Trust
Dr Rose Coates. City, University of London
Dr Camilla Rosan. Anna Freud National Centre for Children and Families
Dr Sally Hogg. Parent-Infant Partnership UK
Mr Andrea Sinesi. University of Stirling

15. * Review question.

State the review question(s) clearly and precisely. It may be appropriate to break very broad questions down into a series of related more specific questions. Questions may be framed or refined using PI(E)COS or similar where relevant.

What are the individual, interpersonal, organizational, political and social factors that prevent women with perinatal mental health problems accessing care or treatment from the NHS or other health and social care services? A review of reviews.

Objectives are to:

1. Determine the barriers and facilitators to women accessing perinatal mental health care or treatment.
2. Identify differences in barriers and facilitators across different health and social care settings.
3. Evaluate the quality of published reviews.
4. Map the geographical distribution of the evidence to establish generalisability and gaps in the evidence.

16. * Searches.

State the sources that will be searched (e.g. Medline). Give the search dates, and any restrictions (e.g. language or publication date). Do NOT enter the full search strategy (it may be provided as a link or attachment below.)

Systematic searches will be conducted through online databases such as: MEDLINE; PsycINFO; PubMed; Cumulative Index to Nursing and Allied Health Literature (CINAHL); EMBASE; Cochrane Library; Web of Science and Scopus. The Turning Research Into Practice (TRIP) database will also be searched. Other search strategies will include: tracking citations of key papers (forward searching); examining reference lists of key papers (backwards searching). When conducting the searches search terms will be combined using Boolean terms "OR" and "AND".

To identify papers, the following parameters will be used:

• Population: Women in the perinatal period (conception to 1 year postpartum). (Search terms will include, but are not limited to: Perinatal OR postnatal OR postpartum OR antenatal).

• Intervention: Assessment, care or treatment for perinatal mental health (search terms will include: help-seeking OR Screening OR assessment OR service* AND Access* OR implement* OR seeking OR decision OR employ OR treatment seeking OR treatment engagement)

• Outcome: Barriers and facilitators, women's experiences, health and social care professionals' experiences (search terms will include: Barriers OR drawbacks OR obstacles OR issues)

Study design: Review papers where a clear systematic search strategy is used and reported (search terms will include: meta-synthesis* OR meta-ethnograph* OR meta-study)

Papers will be selected in two stages according to PRISMA guidelines. In the first stage, titles and abstracts will be screened by one researcher for relevance to the topic. Papers that are clearly not relevant will be excluded. A random selection will be screened by a second researcher and agreement between the two will be calculated using Cohen's Kappa statistic. In the second stage, full texts for papers that appear to be relevant will be obtained and final selection made by the researcher and project management group. Again, a random selection will be screened by a second researcher and agreement between the two will be calculated.

17. URL to search strategy.

Upload a file with your search strategy, or an example of a search strategy for a specific database, (including the keywords) in pdf or word format. In doing so you are consenting to the file being made publicly accessible. Or provide a URL or link to the strategy. Do NOT provide links to your search **results**.

Alternatively, upload your search strategy to CRD in pdf format. Please note that by doing so you are consenting to the file being made publicly accessible.

Do not make this file publicly available until the review is complete

18. * Condition or domain being studied.

Give a short description of the disease, condition or healthcare domain being studied in your systematic review.

Mental health problems affect up to one in five women during pregnancy and the first year after birth (the perinatal period). These include anxiety, depression, stress-related conditions and adjustment disorders. This costs the UK £8.1 billion for every year of babies born. Mental health problems can have a negative effect on women, their partners and their children. They are also one of the leading causes of maternal death. It is vitally important that women who experience mental health difficulties are identified quickly and get the treatment they need. However, only about half of women with perinatal mental health problems are identified by healthcare services and even fewer receive treatment. We will therefore include the following perinatal mental health problems in our review: depression, anxiety, adjustment disorders, OCD, PTSD, psychosis, eating disorders, personality disorders.

19. * Participants/population.

Specify the participants or populations being studied in the review. The preferred format includes details of both inclusion and exclusion criteria.

The population of interest is women in the perinatal period, who are at risk of, or who are experiencing perinatal mental health problems (including, but not limited to, anxiety, depression, PTSD, adjustment disorders). No restrictions will be placed on women's age or ethnicity. If papers include multiple stakeholder views, we will include reviews where the women's responses can be separated out.

Papers will be excluded if they: are non-English publications; are text or opinion pieces, do not have a PRISMA guided search strategy, include people outside the target population (e.g. men/partners or children) where their views cannot be separated from women's views; include interventions targeted at the parent-infant, couple or family relationships; focus on perinatal loss due to the unique focus of the treatment, or focus on substance misuse which has unique challenges in terms of assessment and treatment, or focus on oxidative stress or fetal distress.

20. * Intervention(s), exposure(s).

Give full and clear descriptions or definitions of the interventions or the exposures to be reviewed. The preferred format includes details of both inclusion and exclusion criteria.

We are interested in reviews that look at women's views and experiences of accessing perinatal mental health care across the treatment pathway from deciding to consult or help seeking and disclosing symptoms to assessment, referral, care and treatment.

21. * Comparator(s)/control.

Where relevant, give details of the alternatives against which the intervention/exposure will be compared (e.g. another intervention or a non-exposed control group). The preferred format includes details of both inclusion and exclusion criteria.

Not applicable

22. * Types of study to be included.

Give details of the study designs (e.g. RCT) that are eligible for inclusion in the review. The preferred format

includes both inclusion and exclusion criteria. If there are no restrictions on the types of study, this should be stated.

Review papers that have used a search strategy according the PRISMA guidelines, such as systematic reviews, about access to mental health care or treatment by women in the perinatal period across all stages of the care pathway (deciding to consult, contact with healthcare professionals, assessment/screening, deciding to disclose, referral, access to treatment and provision of optimal treatment). Assessment refers to identifying women who may be at risk for perinatal mental health problems, or who have perinatal mental health problems. Care refers to supportive care or care pathways such as that provided by health professionals who provide care to women with perinatal mental health difficulties across the care pathway. Treatment refers to any active intervention, programmes or protocols to reduce women's perinatal mental health symptoms. The perinatal period is defined as from conception to 1 year postpartum. Mental health measures need to be gold standard clinical interviews or validated self-report questionnaires.

23. Context.

Give summary details of the setting or other relevant characteristics, which help define the inclusion or exclusion criteria.

As women move through pregnancy and the postnatal period, they come into contact with different services that provide women with opportunities to disclose any mental health difficulties and access perinatal mental health care. Despite the services available for women with perinatal mental health difficulties, it is estimated that half of women are not identified despite regular routine contact with these healthcare services, and still fewer receive treatment. For example, a study of postnatal depression suggested only 40% of women with postnatal depression were identified, 24% received treatment, 10% received adequate treatment, and only 3-6% of women recovered. This is likely to be due to a range of factors at individual, interpersonal, organisational and social levels, such as healthcare professionals not asking about mental health, lack of effective assessment, barriers to women seeking help or attending treatment, clinician barriers to diagnosis and treatment, lack of services to refer onto, or limited understanding of effective treatments.

Given that women accessing care or treatment might be important in preventing adverse outcomes for women and their families, we need to identify the wide range of factors that may prevent women from accessing these. The literature on why women with perinatal mental health problems do not access care or treatment is varied and some areas are more clearly synthesised than others. At present, there is no clear overview and synthesis of how these factors may operate at different levels, which is why a systematic review of reviews is needed.

24. * Main outcome(s).

Give the pre-specified main (most important) outcomes of the review, including details of how the outcome is defined and measured and when these measurement are made, if these are part of the review inclusion criteria.

The main aim is to determine the individual, interpersonal, organisational and social factors that prevent women accessing care or treatment. Therefore, the main outcome will be evaluation parameters for assessment, care or treatment for perinatal mental health, including barriers, facilitators, women's experiences, and health and social care professionals' experiences.

Measures of effect

Please specify the effect measure(s) for you main outcome(s) e.g. relative risks, odds ratios, risk difference, and/or 'number needed to treat.

Not applicable.

25. * Additional outcome(s).

List the pre-specified additional outcomes of the review, with a similar level of detail to that required for main outcomes. Where there are no additional outcomes please state 'None' or 'Not applicable' as appropriate to the review

None.

Measures of effect

Please specify the effect measure(s) for you additional outcome(s) e.g. relative risks, odds ratios, risk difference, and/or 'number needed to treat.

Not applicable.

26. * Data extraction (selection and coding).

Describe how studies will be selected for inclusion. State what data will be extracted or obtained. State how this will be done and recorded.

Data will be extracted from eligible reviews using a standard data extraction form in Excel or using the data extraction tool on EPPI-Reviewer. If information is missing, corresponding authors will be contacted with a request for the information. If they do not respond within 2 weeks they will be contacted again. If they do not respond within 4 weeks, missing data will not be included.

A range of data will be extracted to enable a comprehensive meta-review. Key variables will include: Authors; Year; Country; Review design; Method; Sample size and characteristics (of parents, of health care provider); Mental health problem; Outcome measures; Type of intervention(s); Methodological quality rating.

27. * Risk of bias (quality) assessment.

State which characteristics of the studies will be assessed and/or any formal risk of bias/quality assessment tools that will be used.

Quality will be assessed using the AMSTAR checklist which is a reliable and valid measure for assessing the methodological quality of systematic reviews. One reviewer will conduct the quality assessments and reliability of these ratings will be checked="checked" value="1" by a second reviewer rating a random selection of 25% of papers. If disagreements arise then all papers will be double-rated, and disagreements resolved through discussion and consensus. Where consensus cannot be reached the project management group will be consulted and make the final decision. If agreement is low, the second rater will look at a larger

group of papers. If meta-analyses are identified, we will assess the quality of the analysis using the Scottish Intercollegiate Guidelines Network (SIGN) evidence grading system. This system grades the risk of bias associated with a particular piece of evidence on a hierarchy from meta-analysis and RCT evidence (grade 1) down to expert opinion (grade 4), with additional indicators (++, + or -) to indicate methodological quality.

28. * Strategy for data synthesis.

Describe the methods you plan to use to synthesise data. This **must not be generic text** but should be **specific to your review** and describe how the proposed approach will be applied to your data. If meta-analysis is planned, describe the models to be used, methods to explore statistical heterogeneity, and software package to be used.

We will follow a similar strategy to McNeill et al (2012). Firstly, a table summarizing the findings will be presented. Within the table we will include the following information: authors/year; aim; search strategy; number of studies included; total number of participants; results (i.e. key barriers and facilitators identified); quality rating. We will then perform a narrative synthesis of reviews identified, discussing both barriers and facilitators to women accessing perinatal mental health assessment, care and treatment across the care pathway. Where quantitative data is included, we will carry out a narrative synthesis where we indicate both the quality of the evidence (low, medium, high) and whether it is causal or associative in nature as done by Greaves et al., (2011).

Greaves, C.J., Sheppard, K.E., Abraham, C. et al. Systematic review of reviews of intervention components associated with increased effectiveness in dietary and physical activity interventions. BMC Public Health 11, 119 (2011). https://doi.org/10.1186/1471-2458-11-119

McNeill, J., Lynn, F. & Alderdice, F. Public health interventions in midwifery: a systematic review of systematic reviews. BMC Public Health 12, 955 (2012). https://doi.org/10.1186/1471-2458-12-955

29. * Analysis of subgroups or subsets.

State any planned investigation of 'subgroups'. Be clear and specific about which type of study or participant will be included in each group or covariate investigated. State the planned analytic approach.

We will examine recommendations for healthcare practice and research separately, including which countries have a sparsity of data and recommendations for quality improvement in research methods.

30. * Type and method of review.

Select the type of review, review method and health area from the lists below.

Type of review

Cost effectiveness
No

Diagnostic
No

Epidemiologic
No

Individual patient data (IPD) meta-analysis
No

Intervention
No

Living systematic review
No

Meta-analysis
No

Methodology
No

Narrative synthesis
Yes

Network meta-analysis
No

Pre-clinical
No

Prevention
No

Prognostic
No

Prospective meta-analysis (PMA)
No

Review of reviews
Yes

Service delivery
No

Synthesis of qualitative studies
Yes

Systematic review
Yes

Other
No

Health area of the review

Alcohol/substance misuse/abuse
No

Blood and immune system
No

Cancer
No

Cardiovascular
No

Care of the elderly

No

Child health
No

Complementary therapies
No

COVID-19
No

Crime and justice
No

Dental
No

Digestive system
No

Ear, nose and throat
No

Education
No

Endocrine and metabolic disorders
No

Eye disorders
No

General interest
No

Genetics
No

Health inequalities/health equity
No

Infections and infestations
No

International development
No

Mental health and behavioural conditions
Yes

Musculoskeletal
No

Neurological
No

Nursing
No

Obstetrics and gynaecology
No

Oral health
No

Webb R, et al. BMJ Open 2023; 13:e066703. doi: 10.1136/bmjopen-2022-066703

Palliative care
No

Perioperative care
No

Physiotherapy
No

Pregnancy and childbirth
Yes

Public health (including social determinants of health)
No

Rehabilitation
No

Respiratory disorders
No

Service delivery
No

Skin disorders
No

Social care
No

Surgery
No

Tropical Medicine
No

Urological
No

Wounds, injuries and accidents
No

Violence and abuse
No

31. Language.

Select each language individually to add it to the list below, use the bin icon to remove any added in error.
English

There is not an English language summary

32. * Country.

Select the country in which the review is being carried out. For multi-national collaborations select all the countries involved.

England

33. Other registration details.

Name any other organisation where the systematic review title or protocol is registered (e.g. Campbell, or

The Joanna Briggs Institute) together with any unique identification number assigned by them. If extracted data will be stored and made available through a repository such as the Systematic Review Data Repository (SRDR), details and a link should be included here. If none, leave blank.

34. Reference and/or URL for published protocol.

If the protocol for this review is published provide details (authors, title and journal details, preferably in Vancouver format)

Add web link to the published protocol.

Or, upload your published protocol here in pdf format. Note that the upload will be publicly accessible.

No I do not make this file publicly available until the review is complete

Please note that the information required in the PROSPERO registration form must be completed in full even if access to a protocol is given.

35. Dissemination plans.

Do you intend to publish the review on completion?

Yes

Give brief details of plans for communicating review findings.?

We plan to publish this review in a high impact peer reviewed journal.

36. Keywords.

Give words or phrases that best describe the review. Separate keywords with a semicolon or new line. Keywords help PROSPERO users find your review (keywords do not appear in the public record but are included in searches). Be as specific and precise as possible. Avoid acronyms and abbreviations unless these are in wide use.

Perinatal mental health; Barriers; Access; Healthcare; Treatment

37. Details of any existing review of the same topic by the same authors.

If you are registering an update of an existing review give details of the earlier versions and include a full bibliographic reference, if available.

38. * Current review status.

Update review status when the review is completed and when it is published.New registrations must be ongoing so this field is not editable for initial submission.
Please provide anticipated publication date

Review_Ongoing

39. Any additional information.

Provide any other information relevant to the registration of this review.

40. Details of final report/publication(s) or preprints if available.

Leave empty until publication details are available OR you have a link to a preprint (NOTE: this field is not editable for initial submission). List authors, title and journal details preferably in Vancouver format.

Give the link to the published review or preprint.

Appendix 2: Table 1. Search terms

# ▲	MEDLINE (1946-present)
	**Searches**
1	prenatal care/ or perinatal care/ or postnatal care/
2	Pregnancy/
3	Pregnant Women/
4	(pregnancy or pregnant or pre-nat* or prenat* or prepart* or prepart* or ante-nat* or antenat* or ante-part* or antepart* or peri-nat* or perinat* or peri-part* or peripart* or puerper* or post-nat* or postnat* or post-part* or postpart*).ti.
5	((parent? or mother* or maternal or father* or paternal) and (infan* or newborn? or neonat* or baby or babies)).ti.
6	1 or 2 or 3 or 4 or 5
7	mental disorders/ or exp anxiety disorders/ or exp mood disorders/ or exp "trauma and stressor related disorders"/
8	Stress, Psychological/
9	Adaptation, Psychological/
10	(mental* or psych* or anxiety or anxious or depress* or mood? or affect* or distress* or stress or trauma* or posttrauma* or post-trauma* or adjustment disorder* or phobia* or phobic or obsessive compulsive or wellbeing or well-being).ti.
11	7 or 8 or 9 or 10
12	6 and 11
13	Depression, Postpartum/
14	Pregnant Women/px [Psychology]
15	((pregnancy or pregnant or pre-nat* or prenat* or prepart* or prepart* or ante-nat* or antenat* or ante-part* or antepart* or peri-nat* or perinat* or peri-part* or peripart* or puerper* or post-nat* or postnat* or post-part* or postpart*) adj5 (mental* or psych* or anxiety or anxious or depress* or mood? or affect* or distress* or stress or trauma* or posttrauma* or post-trauma* or adjustment disorder* or phobia* or phobic or obsessive compulsive or wellbeing or well-being)).ti,ab.
16	(((parent? or mother* or maternal or father* or paternal) adj5 (infan* or newborn? or neonat* or baby or babies)) and (mental* or psych* or anxiety or anxious or depress* or mood? or affect* or distress* or stress or trauma* or posttrauma* or post-trauma* or adjustment disorder* or phobia* or phobic or obsessive compulsive or wellbeing or well-being)).ti,ab.

17	12 or 13 or 14 or 15 or 16
18	Mass Screening/
19	diagnosis/ or early diagnosis/
20	(screen* or detect* or diagnos* or assess* or identifi* or prevent* or prophyla*).ti.
21	psychotherapy/ or behavior therapy/ or exp cognitive behavioral therapy/
22	counseling/ or exp directive counseling/
23	exp antidepressive agents/ or exp anti-anxiety agents/
24	(intervention? or counsel* or therap* or healing or listen* support* or care or healthcare or service or medication* or drug? or antidepress* or anti-depress* or antianxiety or anti-anxiety).ti.
25	("improving access to psychological therap*" or iapt).ti,ab.
26	((mental* or psych* or anxiety or anxious or depress* or mood? or affect* or distress* or stress or trauma* or posttrauma* or post-trauma* or adjustment disorder* or phobia* or phobic or obsessive compulsive or wellbeing or well-being) adj5 (screen* or detect* or diagnos* or assess* or identifi* or prevent* or prophyla*)).ti,ab.
27	((mental* or psych* or anxiety or anxious or depress* or mood? or affect* or distress* or stress or trauma* or posttrauma* or post-trauma* or adjustment disorder* or phobia* or phobic or obsessive compulsive or wellbeing or well-being) adj5 (intervention? or counsel* or therap* or healing or listen* support* or care or healthcare or service or medication* or drug? or antidepress* or anti-depress* or antianxiety or anti-anxiety)).ti,ab.
28	18 or 19 or 20 or 21 or 22 or 23 or 24 or 25 or 26 or 27
29	17 and 28
30	Depression, Postpartum/di, dh, dt, pc, th
31	29 or 30
32	Implementation Science/ or Health Plan Implementation/
33	Program Evaluation/
34	(implement* or impact*).ti,ab.
35	(feasib* or acceptab*).ti,ab.
36	(barrier? or challenge? or obstacle? or hurdle? or obstruct* or drawback? or issue? or difficult? or promot* or support or encourag* or factor? or facilitat* or enabl* or opportunit* or engage* or assist*).ti,ab.
37	((process or project* or system*) adj5 evaluat*).ti,ab.
38	32 or 33 or 34 or 35 or 36 or 37

39	31 and 38
40	medline.ti,ab.
41	systematic review.pt.
42	meta-analysis.pt.
43	systematic review.ti,ab.
44	(evidence synthesis or realist synthesis or realist review).ti,ab.
45	(Qualitative and synthesis).ti,ab.
46	(meta-synthesis* or meta synthesis* or metasynthesis).ti,ab.
47	(meta-ethnograph* or metaethnograph* or meta ethnograph*).ti,ab.
48	(meta-study or metastudy or meta study).ti,ab.
49	40 or 41 or 42 or 43 or 44 or 45 or 46 or 47 or 48
50	39 and 49
51	(comment or editorial or letter or historical article).pt.
52	50 not 51
53	exp animals/ not humans/
54	52 not 53
55	limit 54 to english language
	**EMBASE (1974 – present)**
1	prenatal care/ or newborn period/ or perinatal period/ or prenatal period/
2	*Pregnancy/
3	(pregnancy or pregnant or pre-nat* or prenat* or prepart* or prepart* or ante-nat* or antenat* or ante-part* or antepart* or peri-nat* or perinat* or peri-part* or peripart* or puerper* or post-nat* or postnat* or post-part* or postpart*).ti.
4	((parent? or mother* or maternal or father* or paternal) and (infan* or newborn? or neonat* or baby or babies)).ti.
5	1 or 2 or 3 or 4
6	mental disease/ or exp anxiety disorder/ or exp mood disorder/
7	mental stress/
8	(mental* or psych* or anxiety or anxious or depress* or mood? or affect* or distress* or stress or trauma* or posttrauma* or post-trauma* or adjustment disorder* or phobia* or phobic or obsessive compulsive or wellbeing or well-being).ti.
9	6 or 7 or 8

10	5 and 9
11	exp perinatal depression/
12	((pregnancy or pregnant or pre-nat* or prenat* or prepart* or prepart* or ante-nat* or antenat* or ante-part* or antepart* or peri-nat* or perinat* or peri-part* or peripart* or puerper* or post-nat* or postnat* or post-part* or postpart*) adj5 (mental* or psych* or anxiety or anxious or depress* or mood? or affect* or distress* or stress or trauma* or posttrauma* or post-trauma* or adjustment disorder* or phobia* or phobic or obsessive compulsive or wellbeing or well-being)).ti,ab.
13	(((parent? or mother* or maternal or father* or paternal) adj5 (infan* or newborn? or neonat* or baby or babies)) and (mental* or psych* or anxiety or anxious or depress* or mood? or affect* or distress* or stress or trauma* or posttrauma* or post-trauma* or adjustment disorder* or phobia* or phobic or obsessive compulsive or wellbeing or well-being)).ti,ab.
14	10 or 11 or 12 or 13
15	mass screening/ or screening test/ or screening/
16	diagnosis/ or early diagnosis/
17	(screen* or detect* or diagnos* or assess* or identifi* or prevent* or prophyla*).ti.
18	exp counseling/ or early intervention/ or exp psychotherapy/
19	(intervention? or counsel* or therap* or healing or listen* support* or care or healthcare or service or medication* or drug? or antidepress* or anti-depress* or antianxiety or anti-anxiety).ti.
20	("improving access to psychological therap*" or iapt).ti,ab.
21	((mental* or psych* or anxiety or anxious or depress* or mood? or affect* or distress* or stress or trauma* or posttrauma* or post-trauma* or adjustment disorder* or phobia* or phobic or obsessive compulsive or wellbeing or well-being) adj5 (screen* or detect* or diagnos* or assess* or identifi* or prevent* or prophyla*)).ti,ab.
22	((mental* or psych* or anxiety or anxious or depress* or mood? or affect* or distress* or stress or trauma* or posttrauma* or post-trauma* or adjustment disorder* or phobia* or phobic or obsessive compulsive or wellbeing or well-being) adj5 (intervention? or counsel* or therap* or healing or listen* support* or care or healthcare or service or medication* or drug? or antidepress* or anti-depress* or antianxiety or anti-anxiety)).ti,ab.
23	15 or 16 or 17 or 18 or 19 or 20 or 21 or 22
24	14 and 23
25	exp perinatal depression/di, dt, pc, th
26	24 or 25

27	therapy delay/
28	exp Program Evaluation/ or Implementation Science/
29	(implement* or impact*).ti,ab.
30	(feasib* or acceptab*).ti,ab.
31	(barrier? or challenge? or obstacle? or hurdle? or obstruct* or drawback? or issue? or difficult? or promot* or support or encourag* or factor? or facilitat* or enabl* or opportunit* or engage* or assist*).ti,ab.
32	((process or project* or system*) adj5 evaluat*).ti,ab.
33	27 or 28 or 29 or 30 or 31 or 32
34	26 and 33
35	medline.tw.
36	"systematic review"/
37	meta-analysis/
38	systematic review.ti,ab.
39	(evidence synthesis or realist synthesis or realist review).ti,ab.
40	(Qualitative and synthesis).ti,ab.
41	(meta-synthesis* or meta synthesis* or metasynthesis).ti,ab.
42	(meta-ethnograph* or metaethnograph* or meta ethnograph*).ti,ab.
43	(meta-study or metastudy or meta study).ti,ab.
44	35 or 36 or 37 or 38 or 39 or 40 or 41 or 42 or 43
45	34 and 44
46	(editorial or letter or note or conference*).pt.
47	45 not 46
48	(exp animals/ or nonhuman/) not human/
49	47 not 48
50	limit 49 to english language
	**PSYCHINFO (1806 – present)**
1	prenatal care/ or postnatal period/ or antepartum period/ or intrapartum period/ or perinatal period/
2	Pregnancy/

3	(pregnancy or pregnant or pre-nat* or prenat* or prepart* or prepart* or ante-nat* or antenat* or ante-part* or antepart* or peri-nat* or perinat* or peri-part* or peripart* or puerper* or post-nat* or postnat* or post-part* or postpart*).ti.
4	((parent? or mother* or maternal or father* or paternal) and (infan* or newborn? or neonat* or baby or babies)).ti.
5	1 or 2 or 3 or 4
6	mental disorders/ or exp affective disorders/ or exp anxiety disorders/ or exp "stress and trauma related disorders"/
7	psychological stress/
8	Emotional Adjustment/
9	(mental* or psych* or anxiety or anxious or depress* or mood? or affect* or distress* or stress or trauma* or posttrauma* or post-trauma* or adjustment disorder* or phobia* or phobic or obsessive compulsive or wellbeing or well-being).ti.
10	6 or 7 or 8 or 9
11	5 and 10
12	postpartum depression/ or postpartum psychosis/
13	((pregnancy or pregnant or pre-nat* or prenat* or prepart* or prepart* or ante-nat* or antenat* or ante-part* or antepart* or peri-nat* or perinat* or peri-part* or peripart* or puerper* or post-nat* or postnat* or post-part* or postpart*) adj5 (mental* or psych* or anxiety or anxious or depress* or mood? or affect* or distress* or stress or trauma* or posttrauma* or post-trauma* or adjustment disorder* or phobia* or phobic or obsessive compulsive or wellbeing or well-being)).ti,ab.
14	(((parent? or mother* or maternal or father* or paternal) adj5 (infan* or newborn? or neonat* or baby or babies)) and (mental* or psych* or anxiety or anxious or depress* or mood? or affect* or distress* or stress or trauma* or posttrauma* or post-trauma* or adjustment disorder* or phobia* or phobic or obsessive compulsive or wellbeing or well-being)).ti,ab.
15	11 or 12 or 13 or 14
16	screening/ or exp health screening/ or exp screening tests/
17	diagnosis/
18	(screen* or detect* or diagnos* or assess* or identifi* or prevent* or prophyla*).ti.
19	treatment/ or exp cognitive behavior therapy/ or exp cognitive techniques/ or exp counseling/ or mindfulness-based interventions/ or exp psychotherapy/
20	(intervention? or counsel* or therap* or healing or listen* support* or care or healthcare or service or medication* or drug? or antidepress* or anti-depress* or antianxiety or anti-anxiety).ti.
21	("improving access to psychological therap*" or iapt).ti,ab.

22	((mental* or psych* or anxiety or anxious or depress* or mood? or affect* or distress* or stress or trauma* or posttrauma* or post-trauma* or adjustment disorder* or phobia* or phobic or obsessive compulsive or wellbeing or well-being) adj5 (screen* or detect* or diagnos* or assess* or identifi* or prevent* or prophyla*)).ti,ab.
23	((mental* or psych* or anxiety or anxious or depress* or mood? or affect* or distress* or stress or trauma* or posttrauma* or post-trauma* or adjustment disorder* or phobia* or phobic or obsessive compulsive or wellbeing or well-being) adj5 (intervention? or counsel* or therap* or healing or listen* support* or care or healthcare or service or medication* or drug? or antidepress* or anti-depress* or antianxiety or anti-anxiety)).ti,ab.
24	16 or 17 or 18 or 19 or 20 or 21 or 22 or 23
25	15 and 24
26	treatment barriers/
27	exp Program Evaluation/
28	(implement* or impact*).ti,ab.
29	(feasib* or acceptab*).ti,ab.
30	(barrier? or challenge? or obstacle? or hurdle? or obstruct* or drawback? or issue? or difficult? or promot* or support or encourag* or factor? or facilitat* or enabl* or opportunit* or engage* or assist*).ti,ab.
31	((process or project* or system*) adj5 evaluat*).ti,ab.
32	26 or 27 or 28 or 29 or 30 or 31
33	25 and 32
34	medline.ti,ab.
35	exp "Systematic Review"/
36	Meta Analysis/
37	systematic review.ti,ab.
38	(evidence synthesis or realist synthesis or realist review).ti,ab.
39	(Qualitative and synthesis).ti,ab.
40	(meta-synthesis* or meta synthesis* or metasynthesis).ti,ab.
41	(meta-ethnograph* or metaethnograph* or meta ethnograph*).ti,ab.
42	(meta-study or metastudy or meta study).ti,ab.
43	34 or 35 or 36 or 37 or 38 or 39 or 40 or 41 or 42
44	33 and 43

45	(comment reply or editorial or letter or "review book" or "review media" or "review software other").dt.
46	44 not 45
47	limit 46 to english language
	**CINAHL (1982 – present)**
S30	S28 NOT S29
S29	S23 AND S27 Limiters - English Language; Publication Type: Book Review, Commentary, Editorial, Letter
S28	S23 AND S27
S27	S24 OR S25 OR S26
S26	TX ("evidence synthesis" or "realist synthesis" or "realist review") OR TX (Qualitative and synthesis) OR TX ((meta-synthesis* or "meta synthesis*" or metasynthesis) OR TX (meta-ethnograph* or metaethnograph* or "meta ethnograph*") OR TX (meta-study or metastudy or "meta study")
S25	TI (medline or "systematic review") OR AB (medline or "systematic review")
S24	(MH "Systematic Review") OR (MH "Meta Analysis") OR (MH "Meta Synthesis")
S23	S19 AND S22
S22	S20 OR S21
S21	((implement* or impact*)) OR ((implement* or impact*)) OR ((feasib* or acceptab*)) OR ((feasib* or acceptab*)) OR ((barrier? or challenge? or obstacle? or hurdle? or obstruct* or drawback? or issue? or difficult? or promot* or support or encourag* or factor? or facilitat* or enabl* or opportunit* or engage* or assist*)) OR ((barrier? or challenge? or obstacle? or hurdle? or obstruct* or drawback? or issue? or difficult? or promot* or support or encourag* or factor? or facilitat* or enabl* or opportunit* or engage* or assist*)) OR (((process or project* or system*) N5 evaluat*)) OR (((process or project* or system*) N5 evaluat*))
S20	(MH "Implementation Science") OR (MH "Program Development+")
S19	S17 OR S18
S18	(MH "Depression, Postpartum/DI/DH/DT/PC/TH") OR (MH "Postpartum Psychosis/DI/DH/DT/TH/PC")
S17	S11 AND S16
S16	S12 OR S13 OR S14 OR S15

S15	TI ((intervention? or counsel* or therap* or healing or listen* support* or care or healthcare or service or medication* or drug? or antidepress* or anti-depress* or antianxiety or anti-anxiety)) OR TI (("improving access to psychological therap*" or iapt)) OR AB (("improving access to psychological therap*" or iapt)) OR TI (((mental* or psych* or anxiety or anxious or depress* or mood? or affect* or distress* or stress or trauma* or posttrauma* or post-trauma* or adjustment disorder* or phobia* or phobic or obsessive compulsive or wellbeing or well-being) N5 (screen* or detect* or diagnos* or assess* or identifi* or prevent* or prophyla*))) OR AB (((mental* or psych* or anxiety or anxious or depress* or mood? or affect* or distress* or stress or trauma* or posttrauma* or post-trauma* or adjustment disorder* or phobia* or phobic or obsessive compulsive or wellbeing or well-being) N5 (screen* or detect* or diagnos* or assess* or identifi* or prevent* or prophyla*))) OR TI (((mental* or psych* or anxiety or anxious or depress* or mood? or affect* or distress* or stress or trauma* or posttrauma* or post-trauma* or adjustment disorder* or phobia* or phobic or obsessive compulsive or wellbeing or well-being) N5 (intervention? or counsel* or therap* or healing or listen* support* or care or healthcare or service or medication* or drug? or antidepress* or anti-depress* or antianxiety or anti-anxiety))) OR AB (((mental* or psych* or anxiety or anxious or depress* or mood? or affect* or distress* or stress or trauma* or posttrauma* or post-trauma* or adjustment disorder* or phobia* or phobic or obsessive compulsive or wellbeing or well-being) N5 (intervention? or counsel* or therap* or healing or listen* support* or care or healthcare or service or medication* or drug? or antidepress* or anti-depress* or antianxiety or anti-anxiety)))
S14	(MH "Antidepressive Agents+")
S13	(MH "Psychotherapy+") OR (MH "Cognitive Therapy+") OR (MH "Counseling+")
S12	(MH "Diagnosis") OR (MH "Early Diagnosis") OR (MH "Health Screening")
S11	S8 OR S9 OR S10

S10	TI (((pregnancy or pregnant or pre-nat* or prenat* or prepart* or prepart* or ante-nat* or antenat* or ante-part* or antepart* or peri-nat* or perinat* or peri-part* or peripart* or puerper* or post-nat* or postnat* or post-part* or postpart*) N5 (mental* or psych* or anxiety or anxious or depress* or mood? or affect* or distress* or stress or trauma* or posttrauma* or post-trauma* or "adjustment disorder*" or phobia* or phobic or "obsessive compulsive" or wellbeing or well-being))) OR AB (((pregnancy or pregnant or pre-nat* or prenat* or prepart* or prepart* or ante-nat* or antenat* or ante-part* or antepart* or peri-nat* or perinat* or peri-part* or peripart* or puerper* or post-nat* or postnat* or post-part* or postpart*) N5 (mental* or psych* or anxiety or anxious or depress* or mood? or affect* or distress* or stress or trauma* or posttrauma* or post-trauma* or "adjustment disorder*" or phobia* or phobic or "obsessive compulsive" or wellbeing or well-being))) OR TI ((((parent? or mother* or maternal or father* or paternal) N5 (infan* or newborn? or neonat* or baby or babies)) and (mental* or psych* or anxiety or anxious or depress* or mood? or affect* or distress* or stress or trauma* or posttrauma* or post-trauma* or "adjustment disorder*" or phobia* or phobic or "obsessive compulsive" or wellbeing or well-being))) AND AB ((((parent? or mother* or maternal or father* or paternal) N5 (infan* or newborn? or neonat* or baby or babies)) and (mental* or psych* or anxiety or anxious or depress* or mood? or affect* or distress* or stress or trauma* or posttrauma* or post-trauma* or "adjustment disorder*" or phobia* or phobic or "obsessive compulsive" or wellbeing or well-being)))
S9	(MH "Depression, Postpartum") OR (MH "Postpartum Psychosis") OR (MH "Expectant Mothers/PF")
S8	S4 AND S7
S7	S5 OR S6
S6	TI mental* or psych* or anxiety or anxious or depress* or mood? or affect* or distress* or stress or trauma* or posttrauma* or post-trauma* or adjustment disorder* or phobia* or phobic or obsessive compulsive or wellbeing or well-being
S5	(MH "Mental Disorders") OR (MH "Anxiety Disorders+") OR (MH "Affective Disorders+") OR (MH "Stress Disorders, Post-Traumatic+") OR (MH "Adaptation, Psychological")
S4	S1 OR S2 OR S3
S3	TI ((pregnancy or pregnant or pre-nat* or prenat* or prepart* or prepart* or ante-nat* or antenat* or ante-part* or antepart* or peri-nat* or perinat* or peri-part* or peripart* or puerper* or post-nat* or postnat* or post-part* or postpart*) OR TI (((parent? or mother* or maternal or father* or paternal) and (infan* or newborn? or neonat* or baby or babies)))
S2	(MH "Expectant Mothers")
S1	(MH "Prenatal Care") OR (MH "Postnatal Period") OR (MH "Pregnancy") OR (MH "Puerperium")
	**SCOPUS**

11	(((TITLE (pregnancy OR pregnant OR pre-nat* OR prenat* OR prepart* OR prepart* OR ante-nat* OR antenat* OR ante-part* OR antepart* OR peri-nat* OR perinat* OR peri-part* OR peripart* OR puerper* OR post-nat* OR postnat* OR post-part* OR postpart*) AND TITLE (mental* OR psych* OR anxiety OR anxious OR depress* OR mood* OR affect* OR distress* OR stress OR trauma* OR posttrauma* OR post-trauma* OR {adjustment disorder*} OR phobia* OR phobic OR {obsessive compulsive} OR wellbeing OR well-being))) AND ((TITLE-ABS-KEY ((screen* OR detect* OR diagnos* OR assess* OR identifi* OR prevent* OR prophyla*)) OR TITLE-ABS-KEY ((intervention* OR counsel* OR therap* OR healing OR listen* AND support* OR care OR healthcare OR service OR medication* OR drugs OR drug OR antidepress* OR anti-depress* OR antianxiety OR anti-anxiety)) OR TITLE-ABS-KEY (("improving access to psychological therap*" OR iapt)))) AND ((TITLE-ABS-KEY (implement* OR impact* OR feasib* OR acceptab*) OR TITLE-ABS-KEY ((barrier* OR challenge* OR obstacle* OR hurdle* OR obstruct* OR drawback* OR issue* OR difficult* OR promot* OR support OR encourag* OR factor? OR facilitat* OR enabl* OR opportunit* OR engage* OR assist*)) OR TITLE-ABS-KEY ("process evaluation*" OR "project evaluation*" OR "systems evaluation*" OR "system evaluation*")))) AND ((TITLE-ABS-KEY ("systematic review" OR meta-analys*) OR TITLE-ABS-KEY ("evidence synthesis" OR "realist synthesis" OR "realist review" OR meta-synthesis* OR "meta synthesis" OR metasynthesis OR meta-ethnograph* OR metaethnograph* OR "meta ethnograph*" OR meta-study OR metastudy OR "meta study") OR TITLE-ABS-KEY (qualitative AND synthesis))) AND (LIMIT-TO (LANGUAGE , "English"))
10	(((TITLE (pregnancy OR pregnant OR pre-nat* OR prenat* OR prepart* OR prepart* OR ante-nat* OR antenat* OR ante-part* OR antepart* OR peri-nat* OR perinat* OR peri-part* OR peripart* OR puerper* OR post-nat* OR postnat* OR post-part* OR postpart*) AND TITLE (mental* OR psych* OR anxiety OR anxious OR depress* OR mood* OR affect* OR distress* OR stress OR trauma* OR posttrauma* OR post-trauma* OR {adjustment disorder*} OR phobia* OR phobic OR {obsessive compulsive} OR wellbeing OR well-being))) AND ((TITLE-ABS-KEY ((screen* OR detect* OR diagnos* OR assess* OR identifi* OR prevent* OR prophyla*)) OR TITLE-ABS-KEY ((intervention* OR counsel* OR therap* OR healing OR listen* AND support* OR care OR healthcare OR service OR medication* OR drugs OR drug OR antidepress* OR anti-depress* OR antianxiety OR anti-anxiety)) OR TITLE-ABS-KEY (("improving access to psychological therap*" OR iapt)))) AND ((TITLE-ABS-KEY (implement* OR impact* OR feasib* OR acceptab*) OR TITLE-ABS-KEY ((barrier* OR challenge* OR obstacle* OR hurdle* OR obstruct* OR drawback* OR issue* OR difficult* OR promot* OR support OR encourag* OR factor? OR facilitat* OR enabl* OR opportunit* OR engage* OR assist*)) OR TITLE-ABS-KEY ("process evaluation*" OR "project evaluation*" OR "systems evaluation*" OR "system evaluation*")))) AND ((TITLE-ABS-KEY ("systematic review" OR meta-analys*) OR TITLE-ABS-KEY ("evidence synthesis" OR "realist synthesis" OR "realist

	review" OR meta-synthesis* OR "meta synthesis" OR metasynthesis OR meta-ethnograph* OR metaethnograph* OR "meta ethnograph*" OR meta-study OR metastudy OR "meta study") OR TITLE-ABS-KEY (qualitative AND synthesis)))
9	(TITLE-ABS-KEY ("systematic review" OR meta-analys*) OR TITLE-ABS-KEY ("evidence synthesis" OR "realist synthesis" OR "realist review" OR meta-synthesis* OR "meta synthesis" OR metasynthesis OR meta-ethnograph* OR metaethnograph* OR "meta ethnograph*" OR meta-study OR metastudy OR "meta study") OR TITLE-ABS-KEY (qualitative AND synthesis))
8	((TITLE (pregnancy OR pregnant OR pre-nat* OR prenat* OR prepart* OR prepart* OR ante-nat* OR antenat* OR ante-part* OR antepart* OR peri-nat* OR perinat* OR peri-part* OR peripart* OR puerper* OR post-nat* OR postnat* OR post-part* OR postpart*) AND TITLE (mental* OR psych* OR anxiety OR anxious OR depress* OR mood* OR affect* OR distress* OR stress OR trauma* OR posttrauma* OR post-trauma* OR {adjustment disorder*} OR phobia* OR phobic OR {obsessive compulsive} OR wellbeing OR well-being))) AND ((TITLE-ABS-KEY ((screen* OR detect* OR diagnos* OR assess* OR identifi* OR prevent* OR prophyla*)) OR TITLE-ABS-KEY ((intervention* OR counsel* OR therap* OR healing OR listen* AND support* OR care OR healthcare OR service OR medication* OR drugs OR drug OR antidepress* OR anti-depress* OR antianxiety OR anti-anxiety)) OR TITLE-ABS-KEY (("improving access to psychological therap*" OR iapt)))) AND ((TITLE-ABS-KEY (implement* OR impact* OR feasib* OR acceptab*) OR TITLE-ABS-KEY ((barrier* OR challenge* OR obstacle* OR hurdle* OR obstruct* OR drawback* OR issue* OR difficult* OR promot* OR support OR encourag* OR factor? OR facilitat* OR enabl* OR opportunit* OR engage* OR assist*)) OR TITLE-ABS-KEY ("process evaluation*" OR "project evaluation*" OR "systems evaluation*" OR "system evaluation*")))
7	(TITLE-ABS-KEY (implement* OR impact* OR feasib* OR acceptab*) OR TITLE-ABS-KEY ((barrier* OR challenge* OR obstacle* OR hurdle* OR obstruct* OR drawback* OR issue* OR difficult* OR promot* OR support OR encourag* OR factor? OR facilitat* OR enabl* OR opportunit* OR engage* OR assist*)) OR TITLE-ABS-KEY ("process evaluation*" OR "project evaluation*" OR "systems evaluation*" OR "system evaluation*"))
	**COCHRANE**
#1	MeSH descriptor: [Prenatal Care] explode all trees
#2	MeSH descriptor: [Perinatal Care] explode all trees

Webb R, *et al. BMJ Open* 2023; 13:e066703. doi: 10.1136/bmjopen-2022-066703

#3	MeSH descriptor: [Pregnancy] this term only
#4	MeSH descriptor: [Pregnant Women] explode all trees
#5	(pregnancy or pregnant or pre-nat* or prenat* or prepart* or prepart* or ante-nat* or antenat* or ante-part* or antepart* or peri-nat* or perinat* or peri-part* or peripart* or puerper* or post-nat* or postnat* or post-part* or postpart*):ti OR (((parent* or mother* or maternal or father* or paternal) and (infan* or newborn* or neonat* or baby or babies))):ti
#6	#1 or #2 or #3 or #4 or #5
#7	MeSH descriptor: [Mental Disorders] this term only
#8	MeSH descriptor: [Anxiety Disorders] explode all trees
#9	MeSH descriptor: [Mood Disorders] explode all trees
#10	MeSH descriptor: [Trauma and Stressor Related Disorders] explode all trees
#11	MeSH descriptor: [Stress, Psychological] explode all trees
#12	MeSH descriptor: [Adaptation, Psychological] this term only
#13	(mental* or psych* or anxiety or anxious or depress* or mood* or affect* or distress* or stress or trauma* or posttrauma* or post-trauma* or adjustment disorder* or phobia* or phobic or obsessive compulsive or wellbeing or well-being):ti
#14	#7 or #8 or #9 or #10 or #11 or #12 or #13
#15	#6 and #14
#16	MeSH descriptor: [Depression, Postpartum] explode all trees
#17	MeSH descriptor: [Pregnant Women] explode all trees and with qualifier(s): [psychology - PX]
#18	(((pregnancy or pregnant or pre-nat* or prenat* or prepart* or prepart* or ante-nat* or antenat* or ante-part* or antepart* or peri-nat* or perinat* or peri-part* or peripart* or puerper* or post-nat* or postnat* or post-part* or postpart*) NEAR/5 (mental* or psych* or anxiety or anxious or depress* or mood* or affect* or distress* or stress or trauma* or posttrauma* or post-trauma* or adjustment disorder* or phobia* or phobic or obsessive compulsive or wellbeing or well-being))):ti,ab,kw
#19	((((parent or parents or mother* or maternal or father* or paternal) NEAR/5 (infan* or newborn* or neonat* or baby or babies)) and (mental* or psych* or anxiety or anxious or depress* or mood* or affect* or distress* or stress or trauma* or posttrauma* or post-trauma* or adjustment disorder* or phobia* or phobic or obsessive compulsive or wellbeing or well-being))):ti,ab,kw
#20	#15 or #16 or #17 or #18 or #19
#21	MeSH descriptor: [Implementation Science] explode all trees

#22	MeSH descriptor: [Health Plan Implementation] explode all trees
#23	MeSH descriptor: [Program Evaluation] explode all trees
#24	(implement* or impact*):ti,ab,kw OR (feasib* or acceptab*):ti,ab,kw OR ((barrier* or challenge* or obstacle* or hurdle* or obstruct* or drawback* or issue* or difficult* or promot* or support or encourag* or factor* or facilitat* or enabl* or opportunit* or engage* or assist*)):ti,ab,kw OR (((process or project* or system*) NEAR/5 evaluat*)):ti,ab,kw
#25	#21 or #22 or #23 or #24
#26	#20 and #25

Appendix 3: Table 2. Search Results

**Database:**	**Interface:**	**Coverage:**	**Date:**	**Hits:**
CINAHL	EBSCOHost	1982-present	04/08/2021	759
Cochrane Database of Systematic Reviews	Cochrane Library, Wiley	Issue 8 of 12, August 2021	04/08/2021	384
Embase	OvidSP	1974-present	04/08/2021	1081
Medline	OvidSP	1946-present	04/08/2021	977
PsycINFO	OvidSP	1806-present	04/08/2021	286
Scopus	Elsevier		04/08/2021	599
**Total:**				**4086**
Duplicates:				1992
Papers excluded:				66
**Final total:**				**2028**

**Papers excluded:** available if needed	
Fetal distress	46
Oxidative stress	9
Non-English	11
	66
Included - 27th May 2020	1671
Included - 4th August 2021	374

Webb R, *et al. BMJ Open* 2023; 13:e066703. doi: 10.1136/bmjopen-2022-066703

Appendix 4: Table 3. Inclusion and exclusion criteria

Category	Criteria
Population	Women in the perinatal period (conception to 12 months after birth) experiencing mental health problems, who may or may not have decided to seek help, accessed help, or engaged in PNMH care. PNMH care was defined as assessment, referrals, and/or treatment/intervention programmes) from health or social care services. Conception to 12 months after birth was chosen as the target population because this is the period that many perinatal mental health services cover [1–5].<br><br>Reviews were excluded if they were not conducted on the target population (e.g., men/partners, healthcare professionals), focused on substance misuse (which has unique challenges in terms of assessment and treatment), did not focus on the mental health of perinatal women.
Outcome	Barriers and facilitators (defined as any individual, healthcare professional, interpersonal, organisational, political, or societal factors that women believed impeded (barriers) or aided (facilitators) them) to seeking, accessing, or engaging in help for PNMH problems. Studies were included if they made descriptive statements about barriers and facilitators to women deciding to seek help, accessing help, and engaging in PNMH care. These descriptions had to be drawn from perinatal women's experiences.<br><br>Reviews were excluded if they did not examine any barriers/facilitators regarding seeking help, accessing help and engaging in PNMH care.
Design	Only systematic reviews were included. Studies that did not use a clearly reported PRISMA search strategy [6]were excluded.<br><br>Non-English publications were also excluded.

Webb R, *et al. BMJ Open* 2023; 13:e066703. doi: 10.1136/bmjopen-2022-066703

Appendix 5: Detailed methodology

Protocol and registration

The protocol for this review has been registered on PROSPERO (CRD42020193107) (see appendix for full protocol).

Eligibility criteria

Studies with the following characteristics were eligible for inclusion in the review: Population: Women in the perinatal period (conception to 12 months after birth) experiencing mental health problems, who may or may not have decided to seek help, accessed help, or engaged in PNMH care. PNMH care was defined as assessment, referrals, and/or treatment/intervention programmes) from health or social care services. Conception to 12 months after birth was chosen as the target population because this is the period that many perinatal mental health services cover [1–5].

Outcome: Barriers and facilitators (defined as any individual, healthcare professional, interpersonal, organisational, political, or societal factors that women believed impeded (barriers) or aided (facilitators) them) to seeking, accessing, or engaging in help for PNMH problems.

Studies were included if they made descriptive statements about barriers and facilitators to women deciding to seek help, accessing help, and engaging in PNMH care. These descriptions had to be drawn from perinatal women's experiences. Only systematic reviews were included. Studies that did not use a clearly reported PRISMA search strategy [6]were excluded. Reviews were also excluded if they were not conducted on the target population (e.g., men/partners, healthcare professionals), focused on substance misuse (which has unique challenges in terms of assessment and treatment), did not focus on the mental health of perinatal women, did not examine any barriers/facilitators regarding seeking help, accessing help and engaging in PNMH care, and were non-English publications.

Information sources

Searches were carried out in CINAHL (1982- present); Embase (1974 – present); Medline (1946-present); and PsycINFO (1806 – present), Cochrane, SCOPUS and TRIP (Turning Research into practice) Medical Database. The date of the last search was 28th May 2020. Forward and backward searches of included studies were carried out and completed by the 26th June 2020. Searches were updated on 4th August 2021 and forward and backward searches of new included studies were completed by 8th September 2021.

Search

Pre-planned searches were carried out using both MeSH terms (i.e. prenatal care/anxiety/ diagnosis) and search terms were combined with Boolean operators "OR" and "AND" (e.g. pregnancy OR perinatal OR postnat* AND anxiety OR depress* OR wellbeing AND intervention? OR counsel* OR support OR identifi* AND OR barrier? OR facilitate*).

Review selection

Search results were imported into Endnote and duplicates and papers not meeting initial inclusion criteria (foetal distress, oxidative stress, Non-English papers) were removed by NR. The remaining studies were imported into Eppi-Reviewer 4, where results were double screened by title and abstract by two people (RW & GC). An additional proportion (n = 166, ~7%) of titles and abstracts were triple screened by NU. Decisions to include or exclude were concordant between RW & GC in 94.2% of cases and between RW & NU in 99.39% of cases. Disagreements were discussed and resolved by NU, GC, and RW by applying the relevant inclusion criteria.

Once title and abstract screening was complete, full text screening was carried out by two people (RW & GC). An additional proportion (n = 9, ~10%) were triple screened by NU. Decisions to include or exclude were concordant between RW & GC in 91.4% of cases and between RW & NU in 100% of cases.

Data collection process and data items

Data extraction was carried out using Microsoft Excel by RW. Each paper was read in full, and relevant parts of the text input into the relevant part of the spreadsheet. Review methodology was copied onto one sheet and results onto another to aid analysis. Double coding of extracted data was carried out for a proportion of included reviews (n = 3, 10%) by GC. Data extraction matched in 85% of cases.

The data that were extracted was guided by the Cochrane Systematic Review for Intervention Data Collection form [7] and the Assessing the Methodological Quality of Systematic Reviews (AMSTAR 2;[8]) Data collected included the following: Review Characteristics (year of publication, author(s), design, aim, search strategy, inclusion/exclusion criteria, screening/study selection, data extraction, quality assessment, analysis methods); Sample Characteristics (Number of studies included, total number of participants, participant demographics); Assessment/Care/Treatment Characteristics (Healthcare setting, intervention description, screening description) and outcomes (barriers and facilitators).

Critical appraisal of reviews

Methodology sections of included systematic reviews were appraised using the AMSTAR 2. Critical domains in the appraisal of systematic reviews according to AMSTAR 2 include protocol registration, adequacy of literature search, justification of study exclusion, risk of bias, appropriateness of meta-analytic methods, consideration of risk of bias when interpreting results, and assessment of publication bias. If more than one critical domain is not met (critical flaw), a systematic review should be evaluated as having critically low confidence in the results of the review. One critical flaw means reviews should be evaluated as low confidence [8].

Given that all studies in this review were qualitative, the AMSTAR 2 items related to meta-analysis were not relevant and were thus removed. Further, given the debate in the literature regarding the appropriateness of conducting risk of bias assessments on qualitative research, we downgraded the items relating to risk of bias from being critical flaws, to flaws. Quality appraisal of all studies was carried out by NU and RW. Ratings were concordant in 90% of cases.

A decision was made to continue to include reviews where confidence in results was evaluated as low and critically low because these reviews focused more on marginalised women, such as refugees, migrants, women with a low income, and women living in LMIC, to ensure the experiences of these seldom-heard women were captured. To improve the validity of results, a qualitative sensitivity analysis was carried out to assess whether themes remained consistent across all reviews, regardless of their quality rating. The methods proposed by Harden[9] and Carroll et al[10] was followed and therefore sensitivity analysis was carried out in two ways: (1) synthesis contribution; (2) evidence of adequate description of themes.

To examine whether higher quality studies contributed more to the themes, a measure of "synthesis contribution" was calculated for each study (as done by Harden, 2007[9]) by dividing the number of barriers and facilitators identified by that study, by the total number of barriers and facilitators identified in the review overall. For example, the findings from Bina (2020) [11]contributed to 31 out of 62 themes, giving this review a synthesis contribution score of 50% (see appendix, Table 3). Each study's synthesis contribution scores was plotted against the number of quality criteria the study

met (see appendix, Figure 1). Statistical analysis (Pearson's correlation) was used to help interpret the plots. To examine whether removing lower quality reviews influenced the number of themes, themes that were only supported by lower quality reviews were identified (see appendix, Table 6).

To examine whether removing lower quality reviews influenced the description of themes, data were assessed for "thickness" or "thinness" (as done by Carroll et al., 2012[10]). Thin description refers to a set of statements (e.g. "O'Mahoney et al. found that women also felt that providers were downplaying the symptoms they were experiencing", Hansotte et al., 2017, [12]p.12), whereas thick description provides the context of experience and circumstances [13] (e.g. "Having symptoms dismissed or attributed to factors other than PPD by health care professionals led to women 'remaining silent.' Some women perceived that their difficulties would only be taken seriously when there were concerns about risk of harm to themselves or the infant. One woman said, 'I kept going to this doctor and he used to give me a pep talk and send me home…'", Hadfield & Wittkowski, 2017[14], p.732). It is argued that the extent to which a text provides a thick description shows evidence of the authenticity of the results [15].

Synthesis of results

Results were analysed by RW using a thematic synthesis [16] in NVivo and Microsoft Excel. First, line by line data coding of statements referring to facilitators or barriers to accessing PNMH care from the results section of each paper was carried out. Next, codes were revisited and assigned a descriptive theme based on their meaning and content. Themes were developed and revised as each review was re-read. Once all codes had been assigned into themes, the themes were mapped onto a multi-level framework adapted from Ferlie and Shortell's Levels of Change framework [17] and a previous systematic review on barriers and facilitators to implementation of PNMH care carried out by the review authors. The mapping of descriptive themes was developed deductively from the initial theoretical framework and then inductively revised as new themes emerged. The mapping of descriptive themes was discussed by all review authors before being finalised. A decision was taken to analyse all reviews together, regardless of the specific aims or individual inclusion criteria. This is because the majority of the reviews (n = 27) included studies carried out in a wide range of countries/settings. This, therefore, made it difficult to parse apart reviews based on sample characteristics, settings, or country of included studies.

Webb R, *et al. BMJ Open* 2023; 13:e066703. doi: 10.1136/bmjopen-2022-066703

Appendix 6: Table 4. Sensitivity analysis

Study	Number of themes	Unique synthesis contribution	Overall synthesis contribution (all themes)
Bina, 2020[11]	31	3.03030303	50
Brealey et al., 2010[18]	13	1.515151515	20.96774194
Button et al., 2017[19]	26	0	41.93548387
Dennis & Chung-Lee, 2006[20]	28	0	45.16129032
Evans et al., 2020[21]	8	0	12.90322581
Giscombe et al., 2020[22]	6	0	9.677419355
Forde,et al. 2020[46]	20	0	29.03
Hadfield & Wittkowski, 2017[14]	25	0	40.32258065
Hansotte et al., 2017[12]	19	1.515151515	30.64516129
Hewitt et al., 2009[23]	13	0	20.96774194
Holopainen & Hakulinen, 2019[24]	6	0	9.677419355
Jones et al., 2014[25]	10	0	16.12903226
Jones, 2019[26]	19	0	30.64516129
Kassam, 2019[27]	8	0	12.90322581
Lucas et al., 2019[28]	9	0	14.51612903
Megnin-Viggars et al., 2015[29]	26	0	41.93548387
Mollard et al., 2016[30]	5	1.515151515	8.064516129
Morrell et al., 2016[31]	16	0	25.80645161
Newman et al., 2019[32]	13	0	20.96774194
Nilaweera et al., 2014[33]	6	0	9.677419355
Praetorius et al., 2020[34]	3	0	4.838709677
Randall & Briscoe, 2018[35]	2	0	3.225806452
Sambrook-Smith et al., 2019[36]	19	0	30.64516129
Schmied et al., 2017[37]	27	1.515151515	43.5483871
Scope et al., 2017[38]	13	0	20.96774194
Slade et al., 2020[39]	15	0	24.19354839
Sorsa et al., 2021[40]	19	0	30.64516129

Staneva et al., 2015[41]	11	0	17.74193548
Tobin et al., 2018[42]	19	0	30.64516129
Viveiros & Darling, 2018[43]	16	1.515151515	25.80645161
Watson et al., 2019[44]	28	0	45.16129032
Wittkowski et al., 2014[45]	2	1.515151515	3.225806452

Webb R, et al. BMJ Open 2023; 13:e066703. doi: 10.1136/bmjopen-2022-066703

Appendix 7: *Figure 1.* Synthesis contribution vs quality appraisal criteria met

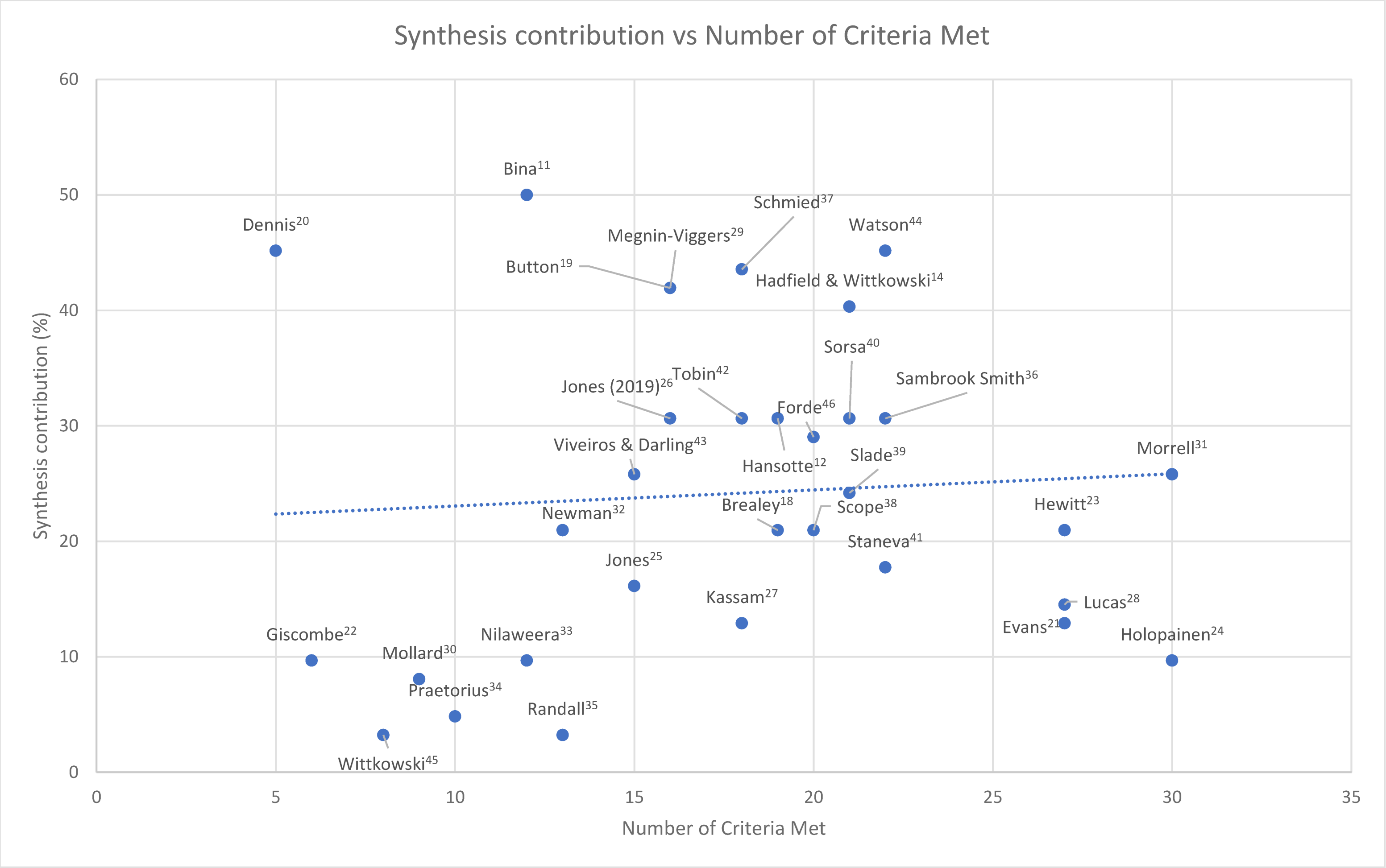

Appendix 8: Sensitivity analysis results

Synthesis contribution. There was no correlation between synthesis contribution and the number of criteria each review met (r = .142, p = .437; see appendix, Figure 1). Furthermore, only four themes (**cultural/spiritual** causes of mental illness, **age, previous diagnoses,** and **appropriateness of care)** were only identified by lower quality studies showing the majority of themes (58 out of 62; 93.55%) were supported by both higher quality and lower quality papers.

Richness of data. The removal of lower quality papers meant that the theme **language barriers** lost some of its richness. For example, it led to the removal of quotes expressing frustration from women whose first language was not English:

> '. . .you don't know where to go, what to do, who to trust, especially when you are coming by yourself. . . you believe that you speak English, but when you get here you realize that you don't.' [37]
>
> 'Sometimes when you have a baby, a woman comes from the hospital. Bengali girls don't come with the midwife, we don't understand what they say, we just sit there staring at their faces.' [19]

The removal of lower quality papers from the theme **fear of being seen as a bad mum** led to the loss of richness of data including the removal of a quote from women who have migrated from their country of birth:

> 'Back home, if someone has this problem, everyone gossips, you get this feeling that people are not dealing with you normally or as if you are abnormal almost. . .' [37]

Lastly, the removal of lower quality studies meant important information was removed from the **characteristics of service** theme, such as women feeling services prioritise physical needs (n = 2), lack information about screening guidelines (n = 2), and the logistics (e.g. location, time of appointments) of the care (n = 3)

Webb R, *et al. BMJ Open* 2023; 13:e066703. doi: 10.1136/bmjopen-2022-066703

Appendix 9: Table 5. Summary review characteristics

Characteristics	Range; Mean (M), Median (Mdn); Interquartile Range (IQR)
Year	2006-2022; M = 2017, Mdn = 2018; IQR = 2016-2019
No. studies included in each review	4-40; M = 16, Mdn = 13, IQR = 9-19
No. women included in each review	95-85,190; M = 5080; Mdn = 463; IQR = 226-1,715
Countries	N = 24

Appendix 10: Table 6. Characteristics of included reviews

	Review details			Participant details					
Author & Year	N studies about women (Total N)<br><br>Years (Range)	Inclusion criteria	Country of studies	N<br><br>M (SD)	Age	Perinatal period	Ethnicity	Mental illness	Socioeconomic status
Bina (2020)[11]	31 (35)<br><br>1993-2018	Service use for postpartum depression or "distress" from women (and HCP) perspectives	11 countries (4 LMIC)	7219<br><br>232.9 (414.7)	NR	2 weeks - up to 3 years postnatal	6 studies specified recruitment of migrant women or women of colour (EA; H; Ar)	Depressive symptoms, emotional difficulties or current/past diagnosis of mood disorder	2 studies recruited women with low income.<br>1 study recruited women using Medicaid.
Brealey et al. (2010)[18]	13 (16)<br><br>1997-2007	Acceptability to women (and HCP) of screening to identify women with increased risk of	5 countries (all HIC)	1715<br><br>131.9 (253.06)	24-34<br>M (n = 8) = 29.63	First antenatal appointment – 12 months after birth	2 studies recruited women of colour (B; EA; Ar)	Women at risk of postnatal depression	One study reported marriage (29/30 women were married)

		postnatal depression							
Button et al. (2017)[19]	24 (24)<br><br>1993-2016	Help seeking for postnatal depression	9 studies carried out in UK. No other countries reported.	NR	NR	Postnatal	9 studies recruited women of colour. 3 studies had mixed samples.	Postnatal depression	NR
Dennis and Chung-Lee (2006)[20]	40 (40)<br><br>NR	Maternal help-seeking barriers and facilitators and treatment preferences for postnatal depression	3 were explicitly stated (all HIC)	NR	NR	Up to 1 year after birth	Three studies recruited women of colour(SA; EA;B;Ar)	Postnatal depression	NR
Evans et al. (2020)[21]	14 (14)<br><br>2009-2015	Acceptability of non-pharmacologic al interventions for antenatal anxiety	6 countries (all HIC)	235<br><br>16.8 (9.6)	NR	Between 6-40 weeks gestation	NR	8 studies recruited women with a history of mood concerns/ anxiety or depression	2 studies recruited women with "social risk factors"
Forde, Peters & Wittkowski (2020)[46]	13 (15)<br><br>2003-2018	Published empirical studies exploring women's or family members' experiences of PP and/or recovery using	4 countries (all HIC)	103<br>7.92 (2.96)	Range: 23-62	All postnatal, ranging from 4 months to 26 years after onset of postnatal psychosis	One woman was an Orthodox Jewish woman	All women had recovered from, or were currently experiencing postnatal psychosis	NR

		a qualitative methodology							
Giscombe, Hui & Stickley (2020)[22]	8 (8)<br><br>2008-2017	Refugee or asylum-seeking women, with mental health complications during perinatal period	3 countries (all HIC)	NR	NR	NR	Syrian refugees, Eritrean refugees	6 studies recruited women with depression; 3 with PTSD (1 study recruited both depression & PTSD)	All women were refugees or asylum seekers
Hadfield and Wittkowski (2017)[14]	17 (17)<br><br>2004-2015	Mothers with postnatal depression and their experiences about help seeking for psychosocial support	4 countries (all HIC)	532<br><br>31.3 (25.97)	Range 18-45<br><br>M (n = 2) = 30.2	Postnatal	3 studies recruited women who weren't born in the UK[(B;EA)]	Postnatal depression	NR
Hansotte et al. (2017)[12]	18 (18)<br><br>2004-2015	Screening for postnatal depression and barriers to accessing treatment in low-income women in western countries.	2 countries (all HIC)	85190<br><br>5011 (11613)	M (n = 11) = 25.11	Postnatal	All studies recruited a diverse sample of migrant women or women of colour [(B; L; W; As; NI)]	Self-report depression symptoms or depression diagnosis	All women were low income living in high income western country.
Hewitt et al. (2009)[23]	13 (16)<br><br>1997-2007	Acceptability to women (and HCP) about methods to	5 countries (all HIC)	1715<br><br>131.9 (253.06)	M (n = 8) = 29.63	Postnatal: 1-12 months	4 studies recruited women of	Perinatal depression	2 studies looked at marriage. The majority of women

		identify postnatal depression				Antenatal: all trimesters	colour (Ar; EA; B; NI; NS)		were married (87-97%)
Holopainen and Hakulinen (2019)[24]	13 (15) 2005-2015	Mothers (and fathers) experiences of postnatal depression symptoms	7 countries (all HIC)	199 15.31 (8.21)	Ages ranged from 16-45	1-12 months after birth	5 studies recruited women of colour (B, L, H, SA, EA)	Most studies looked at symptoms of depression, 2 looked at diagnoses	1 study recruited low-income women, one recruited adolescent mothers. Most women were married (n = 3; 59-66%). Most women had more than 9 years of education (n = 2; 87-100%)
Jones et al. (2014)[25]	5 (5) 1995-2012	Women's experiences of peer support for any degree of perinatal mental illness	3 countries (all HIC)	95 19 (18.93)	NR	6 weeks - 2 years after birth	NR	Postnatal depression diagnosis or symptoms	NR
Jones (2019)[26]	19 (19) 2008-2017	Help seeking in women with perinatal depression	All USA	6089 358.90 (1226.22)	NR	Pregnancy – 6 months after birth	6 studies recruited women of colour (B, L, SA, EA, NS)	All had perinatal depression identified through screening measures, or self-reported.	All women had pregnancy complications. 3 studies recruited women on a low income.
Kassam (2009)[27]	11 separate populations 1999-2013	Voices of immigrant and refugee women with postnatal depression in terms of social support as a	3 countries stated (HIC & UMIC countries)	191 23.88 (10.89)	All aged over 17	Screened high on a postnatal depression scale within 2 weeks - 5 years after birth	All studies recruited migrant women or women of colour (NS; As; Ar; SA; H)	Most had postnatal depressive symptoms, identified through screening. One study reported	One study looked at risk profile of women (e.g. low income, experienced violence, experienced war, previous mental health difficulty).

		coping resource						depression diagnosis	All women in 2 studies were married or in a relationship. One study recruited low-income women.
Lucas et al. (2019)[28]	19 (19)<br><br>1999-2017	Young women's perception of their mental health and wellbeing	3 countries (all HIC)	356<br><br>18.74 (10.02)	Ages ranged from 13-25.<br><br>M (n = 2) = 18.75	11 studies recruited were parents (3 months - 2 years postnatal). 2 studies recruited pregnant women. Remaining studies recruited both pregnant and postnatal women	Majority of studies (15) recruited ethnically diverse (L, B, H, SA, M, As) samples. 4 studies did not report ethnicity	Depressive symptoms, depression diagnosis, other diagnoses (bipolar, panic disorder, mood disorder).	All women were young (maximum age 25)
Megnin-Viggars et al. (2015)[29]	39 (39)<br><br>2001-2013	Women with, or at risk of developing a postnatal mental health problem and their views on factors that improve or	Only reported for 3 studies (all UK)	955<br><br>24.49 (43.77)	1 study recruited teenage mother. No other ages reported.	Antenatal and postnatal	5 studies recruited ethnically diverse samples (B, NS, SA)	Most studies recruited women with depression (n = 14) or women at risk (n = 18) of perinatal mental health problems.	1 study recruited teenage mothers

		diminish access to perinatal mental health services							
Mollard et al. (2016)[30]	11 (11)<br><br>1995-2014	Women living in rural areas of the USA with PPD. Looking at screening uptake, intervention acceptability, lived experience, help-seeking.	All USA	1610<br><br>146.36 (159.57)	NR	Postnatal	5 studies recruited ethnically diverse samples (NI; B; NS; H)	Postnatal depression symptoms, most used EPDS[1] (n = 6) screen	All women lived in rural location, 3 studies recruited low income women
Morrell et al. (2016)[31]	38 individual samples of women in the qualitative review<br><br>1987-2013	Pregnant and postnatal women, views on preventative or targeted services for PND	8 countries (1 LMIC – India)	1673 (34 studies reported sample size)<br><br>49.21 (98.49)	Ages ranged from 15-54<br><br>M (n = 12) = 28.62	Pregnancy and postnatal	10 recruited ethnically diverse samples (SA; EA, B; H; NI; L; M; NS)	Depression - both symptoms and diagnoses	25 studies reported sociodemographic characteristics. 16 studies reported marital status, in all but 1 study the majority of women were married/cohabiting/in a relationship. 8 studies reported education status: most had completed high school or above. 4 studies recruited low-income women or those living in an

									impoverished/deprived area
Newman et al. (2019)[32]	4 (4)<br><br>2008-2016	Women with depression during the postnatal period sharing views on help-seeking	3 countries (all HIC)	118<br><br>29.5 (9)	M (n = 3) = 31.97	Postnatal	NR	Depressive symptoms, measured by EPDS[1]	NR
Nilaweera et al. (2014)[33]	9 (15)	Women who have migrated from South Asian countries to live in high-income countries, barriers and enablers to health care access	4 countries (all HIC)	20,788<br><br>2309.78 (3926.13)	NR	2 weeks to 5 years postnatal	All studies recruited women born in South Asia	Most (n = 5) used EPDS[1] to assess postnatal depression symptoms	NR
Praetorius, Maxwell & Alam (2020)[34]	8 (8)<br><br>1999-2016	Mothers with depression and suicidality	5 countries (3 HIC, 1 UMIC, 1LMIC)	199<br><br>24.88 (12.52)	Ages range from 17-44	Pregnancy and postnatal	All studies recruited diverse samples [B, L, M, SA, EA, Ar, W]	All women had depression and suicidality	NR
Randall and Briscoe (2018)[35]	4 (4)<br><br>2005-2014	Women's decision-making process around antidepressant	2 countries (all HIC)	368<br><br>92 (37.09)	Ages ranged from 25-34	Pregnancy	3 studies reported ethnicity. The majority of women	Depression – 1 study used the CES-D[2] to identify depressive symptoms	3 studies report education, the majority (82.5-100%) were educated to above high school level. 3 studies

		use during pregnancy			M (n = 2) = 31		were white (77.5-95%)		reported relationship status, the majority (80-98%) were married/living with partner
Slade, Molyneux & Watt (2021)[39]	13 (13 – qualitative papers only)<br><br>2007-2019	Help seeking for birth trauma/ postnatal PTSD	7 countries (1 UMIC; 6 HIC)	394<br><br>30.31 (32.85)	Ages range from 2-45<br><br>M (n=4) = 32	Up to 18 months after birth	8 studies reported ethnicity. One study reported recruiting women of colour[(B, H)]	All PTSD after birth	One study recruited low-income women. 2 studies reported marital status, over 58% were married. 2 studies reported higher education, at least 50% of women had completed this.
Sambrook-Smith et al. (2019)[36]	24 (35)<br><br>2007 - 2018	Barriers to accessing perinatal mental health care from the perspective of women (families & HCP)	All UK	384<br><br>16 (8.80)	NR	Postnatal	9 recruited women of colour [(B; SA; EA)]	Most looked at depressive symptoms (n = 12). Studies also recruited women with antenatal anxiety (n = 1), postnatal psychosis (n = 5), PTSD (n = 1) and substance misuse (n =1)	NR
Sorsa, Kylma and Bondas (2021)[40]	14 (14)<br><br>2002-2018	Helpseeking in women with perinatal distress	5 countries (all HIC)	345<br><br>24.65 (11.99)	Ages ranged from 18-55	Antenatal and postnatal	NR	Postnatal depression (n -= 8); prenatal depression (n = 2); Perinatal	NR

					M (n = 7) = 30.21			mental health needs (n = 2); Postpartum mood disorder (n = 1), Bipolar disorder (n = 1)	
Schmied et al. (2017)[37]	12 individual samples<br><br>1999-2015	Migrant women living in high income countries	4 countries (all HIC)	250<br><br>20.83 (12.52)	M (n = 5) = 29.4	Postnatal	All studies recruited migrant women or women of colour (SA; EA; H; B; Ar; L)	Depressive symptoms or formal diagnosis	1 study recruited low income women
Scope et al. (2017)[38]	22 individual samples<br><br>1987 - 2014	Service user views on uptake, acceptability of preventative interventions for PND	7 countries (all HIC)	982 (reported by author)	13-45 years	Antenatal and postnatal	NR	NR	NR
Staneva et al. (2015)[41]	8 (8)<br><br>2006-2012	Womens experience of antenatal mental health difficulties	5 countries (1 LMIC - Cambodia)	1094<br><br>14 (6.26)	Ages ranged from 16-47	Antenatal	Most studies (n = 6) recruited ethnically diverse samples (B; M)	Self-report distress, depression (n = 5); diagnoses depression/anxiety (n = 2); FOC = 1	50-100% of women were in a relationship
Tobin et al. (2018)[42]	8 (individual samples)	Refugee or immigrant women's experiences of	3 countries (all HIC)	139<br><br>17.38 (7.98)	Age ranges between	Postnatal	All studies recruited migrant women	Postnatal depression	6 studies reported relationship status 50-85% of women were

	2004-2013	postpartum depression			17-54 years		and women of colour(L; H; SA;EA;B)		married/in a relationship
Viveiros and Darling (2019)[43]	7 (26)<br><br>2009-2018	To explore women (and midwives) perceptions on factors that impede access to perinatal mental health care in high resource settings	2 countries (both HIC)	301<br><br>43 (66.30)	1 study reported age range from 23-40	Antenatal and postnatal	2 studies recruited 'BAME' women, one recruited all Black women	PTSD symptoms (n = 1); mental health problems (n = 2); mental illness diagnosis (n = 1)	NR
Watson et al. (2019)[44]	15 (15)<br><br>1994-2015	Ethnic minority women's experience of perinatal mental ill health, help-seeking and perinatal mental health services in Europe	All UK	4970<br><br>331.33 (1173.09)	NR	Antenatal and postnatal	All studies recruited women of colour (SA; NS; N; EA; M)	Distress, depression, mood and mental health, well-being	NR
Wittkowski et al. (2014)[45]	12 (12)<br><br>1983 - 2009	Culturally determined risk factors of PND in Sub-Saharan Africa	3 countries – all Sub-Saharan Africa	3642<br><br>404.67 (343.16)	NR	Postnatal	NR	All used self-report measures of depression	NR

Note. Where studies recruited populations that were not perinatal women, the information from these studies are not included in this table. HCP = Healthcare professional; LMIC = Lower-Middle Income Country; HIC = Higher Income Country; PTSD = Post-traumatic stress disorder; FOC = Fear of Childbirth. 1 = Edinburgh Postnatal Depression Scale (Cox et al., 1987); 2 = Center for Epidemiological Studies-Depression (Radloff, 1977).

For ethnicities: As = Asian (where the area of Asia was not specified in the study); EA = East Asian (e.g. Vietnamese; Chinese; Thai); SA = South Asian (e.g. Indian; Bangladeshi; Pakistani; Sri-Lankan); Ar = Arab countries (e.g. Jordanian, Egyptian); Ar = Arabic; B = Black; H = Hispanic; L = Latina; M = mixed or multiple ethnic groups; NI = Native/Indigenous; NS = Not specified; W = White.

Appendix 11: Table 7. Quality appraisal

Author, year	Q1. PICO	Q2. Protocol*	Q3. Study design	Q4. Literature search*	Q5. Study selection	Q6. Data extraction	Q7. Excluded studies*	Q8. Included studies	Q9. ROB	Q.10 Funding	Q13. ROB interpretation	Q14. Heterogeneity	Q16. conflict of interest*	Rating
**Bina, 2020[11]**	Yes	Yes	No	Partial yes	Yes	No	Partial yes	Yes	No	No	No	Yes	No	LOW
**Brealey et al., 2010[18]**	Yes	Yes	Yes	Yes	Yes	Yes	No	Yes	Partial yes	No	Yes	Yes	No	CRITICALLY LOW
**Button et al., 2017[19]**	Yes	No	Yes	Yes	No	No	Partial yes	Partial yes	Yes	No	Yes	Yes	Yes	LOW
**Dennis & Chung-Lee, 2006[20]**	Yes	No	Yes	Yes	No	Yes	No	Partial yes	No	No	No	No	No	CRITICALLY LOW
**Evans et al., 2020[21]**	Yes	Yes	Yes	Yes	Yes	Yes	Partial yes	Yes	Yes	No	Yes	Yes	Yes	HIGH
**Forde et al., 2020[46]**	Yes	Yes	Yes	Partial yes	Yes	No	Partial yes	Ys	Yes	No	Yes	Yes	Yes	MODERATE
**Giscombe et al., 2020[22]**	Yes	No	Yes	Partial yes	No	No	Partial yes	Yes	Yes	No	No	Yes	No	CRITICALLY LOW
**Hadfield & Wittkowski, 2017[14]**	Yes	Yes	Yes	Yes	No	No	Partial yes	Yes	Yes	No	Yes	Yes	Yes	MODERATE
**Hansotte et al., 2017[12]**	Yes	No	Yes	Yes	Yes	Yes	Partial yes	Yes	No	No	No	Yes	Yes	LOW
**Hewitt et al., 2009[23]**	Yes	Yes	Yes	Yes	Yes	Yes	Partial yes	Yes	Yes	No	Yes	Yes	Yes	HIGH
**Holopainen & Hakulinen, 2019[24]**	Yes	Yes	Yes	Yes	Yes	Yes	Yes	Yes	Yes	No	Yes	Yes	Yes	HIGH

**Jones et al., 2014[25]**	Yes	No	Yes	Yes	No	Yes	No	Partial yes	Yes	No	Yes	Yes	Yes	CRITICALLY LOW
**Jones, 2019[26]**	Yes	No	Yes	Partial yes	Yes	No	Partial yes	Yes	No	No	Yes	Yes	Yes	LOW
**Kassam, 2019[27]**	Yes	Yes	Yes	Partial yes	No	No	Partial yes	Yes	Yes	No	Yes	Yes	Yes	MODERATE
**Lucas et al., 2019[28]**	Yes	Yes	Yes	Yes	Yes	Yes	Partial yes	Yes	Yes	No	Yes	Yes	Yes	HIGH
**Megnin-Viggars et al., 2015[29]**	Yes	Yes	Yes	Partial yes	No	Yes	Partial yes	Yes	Yes	No	Yes	Yes	No	LOW
**Mollard et al., 2016[30]**	Yes	No	Yes	Partial yes	No	No	Partial yes	Partial yes	No	No	No	Yes	Yes	LOW
**Morell et al. 2016[31]**	Yes	Yes	Yes	Yes	Yes	Yes	Yes	Yes	Ye	No	Yes	Yes	Yes	HIGH
**Newman et al., 2019[32]**	Yes	No	Yes	Yes	No	No	Partial yes	Yes	Yes	No	No	No	Yes	LOW
**Nilaweera et al., 2014[33]**	Yes	No	No	Partial yes	No	No	Partial yes	Yes	Yes	No	Yes	Yes	Yes	LOW
**Praetorius et al., 2020[34]**	No	No	Yes	Yes	No	Yes	Partial yes	Partial yes	No	No	No	No	Yes	LOW
**Randall & Briscoe, 2018[35]**	Yes	No	No	Partial yes	Yes	No	Partial yes	Partial yes	Yes	No	No	Yes	Yes	LOW
**Sambrook-Smith et al., 2019[36]**	Yes	Yes	Yes	Partial yes	Yes	No	Partial yes	Yes	Yes	No	Yes	Yes	Yes	MODERATE
**Schmied et al., 2017[37]**	Yes	No	Yes	Partial yes	Yes	No	Partial yes	Yes	Yes	No	Yes	Yes	Yes	LOW
**Scope et al., 2017[38]**	Yes	Yes	Yes	Partial yes	Yes	Yes	Partial yes	Yes	Yes	No	Yes	Yes	No	LOW

**Staneva et al., 2015[41]**	Yes	Yes	Yes	Partial yes	Yes	No	Partial yes	Yes	Yes	No	Yes	Yes	Yes	MODERATE
**Slade et al., 2020[39]**	Yes	Yes	Yes	Yes	No	No	Partial yes	Yes	Yes	No	Yes	Yes	Yes	MODERATE
**Sorsa et al., 2021[40]**	Yes	No	Yes	Partial yes	Partial yes	Yes	Partial yes	Yes	Yes	No	Yes	Yes	Yes	LOW
**Tobin et al., 2018[42]**	Yes	Yes	Yes	Yes	No	No	No	Yes	Yes	No	No	Yes	Yes	MODERATE
**Viveiros & Darling, 2018[43]**	Yes	No	No	Yes	Yes	No	Partial yes	Yes	No	No	No	Yes	Yes	LOW
**Watson et al., 2019[44]**	Yes	Yes	Yes	Partial yes	Yes	No	Partial yes	Yes	Yes	No	Yes	Yes	Yes	MODERATE
**Wittkowski et al., 2014[45]**	Yes	No	Yes	Partial yes	No	No	Partial yes	Yes	Yes	No	Yes	Yes	No	CRITICALLY LOW

* = Critical domain

1. Did the research questions and inclusion criteria for the review include the components of PICO? *2.* Did the report of the review contain an explicit statement that the review methods were established prior to the conduct of the review and did the report justify any significant deviations from the protocol?*3.* Did the review authors explain their selection of the study designs for inclusion in the review?4. Did the review authors use a comprehensive literature search strategy? 5. Did the review authors perform study selection in duplicate? 6. Did the review authors perform data extraction in duplicate? 7. Did the review authors provide a list of excluded studies and justify the exclusions? 8. Did the review authors describe the included studies in adequate detail? 9. Did the review authors use a satisfactory technique for assessing the risk of bias (RoB) in individual studies that were included in the review? 10. Did the review authors report on the sources of funding for the studies included in the review? 11. If meta-analysis was performed, did the review authors use appropriate methods for statistical combination of results? *(not applicable)* 12. If meta-analysis was performed, did the review authors assess the potential impact of RoB in individual studies on the results of the meta-analysis or other evidence synthesis? *(not applicable)* 13. Did the review authors account for RoB in primary studies when interpreting/discussing the results of the review? 14. Did the review authors provide a satisfactory explanation for, and discussion of, any heterogeneity observed in the results of the review? 15. If they performed quantitative synthesis did the review authors carry out an adequate investigation of publication bias (small study bias) and discuss its likely impact on the results of the review? *(not applicable)* 16. Did the review authors report any potential sources of conflict of interest, including any funding they received for conducting the review?

Appendix 12: Table 8. Themes

Theme	Studies reflecting this theme
**1. Women**	
**1.1 Beliefs about health services**	
**1.1.1 Medication only**	Bina, 2020; Button et al., 2017; Jones, 2019; Megnin-Viggars et al., 2015; Nilaweera et al., 2014; Sorsa et al., 2021; Tobin et al., 2018
**1.1.2 Stretched**	Hadfield & Wittkowski, 2017
**1.2 Beliefs about healthcare professionals**	
**1.2.1 What is their role?**	Brealey et al., 2010; Button et al., 2017; Dennis & Chung-Lee, 2006; Hadfield & Wittkowski, 2017; Hewitt et al., 2009; Megnin-Viggars et al., 2015; Mollard et al., 2016; Morrell et al., 2016; Nilaweera et al., 2014; Schmied et al., 2017; Scope et al., 2017; Smith et al., 2019
**1.2.2 They won't be interested**	Bina, 2020; Hadfield & Wittkowski, 2017
**1.3 Beliefs about mental illness**	
**1.3.1 Not knowing what it is**	Bina, 2020; Button et al., 2017; Dennis & Chung-Lee, 2006; Hadfield & Wittkowski, 2017; Hansotte et al., 2017; Jones, 2019; Lucas et al., 2019; Megnin-Viggars et al., 2015; Morrell et al., 2016; Newman et al., 2019; Schmied et al., 2017; Scope et al., 2017; Smith et al., 2019; Staneva et al., 2015; Tobin et al., 2018; Watson et al., 2019
**1.3.1.1. Not having the language to describe perinatal mental illness**	Brealey et al., 2010; Staneva et al., 2015; Tobin et al., 2018; Watson et al., 2019
**1.3.2 Causes**	
**1.3.2.1 Cultural/spiritual**	Schmied et al., 2017; Wittkowski et al., 2014
**1.3.2.2 External factors**	Bina, 2020; Button et al., 2017; Dennis & Chung-Lee, 2006; Lucas et al., 2019; Schmied et al., 2017; Staneva et al., 2015; Tobin et al., 2018; Watson et al., 2019
**1.3.2.3 Physical factors**	Bina, 2020; Button et al., 2017; Dennis & Chung-Lee, 2006; Forde et al., 2020; Jones et al., 2014; Newman et al., 2019; Schmied et al., 2017; Smith et al., 2019; Staneva et al., 2015; Watson et al., 2019
**1.3.2.4 A normal response to motherhood?**	Dennis & Chung-Lee, 2006; Giscombe et al., 2020; Jones et al., 2014; Schmied et al., 2017; Slade et al., 2020; Smith et al., 2019; Sorsa et al., 2021
**1.3.3 How to deal with symptoms**	
**1.3.3.1 Ignore them**	Bina, 2020; Hadfield & Wittkowski, 2017; Jones, 2019; Newman et al., 2019; Schmied et al., 2017; Slade et al., 2020
**1.3.3.2 Seek spiritual guidance**	Hansotte et al., 2017; Kassam, 2019; Schmied et al., 2017; Watson et al., 2019
**1.4 Deciding to seek help**	

**1.4.1 Recognising something is wrong**	Bina, 2020; Button et al., 2017; Forde et al., 2020; Hadfield & Wittkowski, 2017; Hansotte et al., 2017; Slade et al., 2020; Sorsa et al., 2021; Staneva et al., 2015; Viveiros & Darling, 2018
**1.4.2 Where do I go to seek help?**	Bina, 2020; Dennis & Chung-Lee, 2006; Hansotte et al., 2017; Jones, 2019; Megnin-Viggars et al., 2015; Schmied et al., 2017; Smith et al., 2019; Sorsa et al., 2021; Tobin et al., 2018
**1.5 Fear of judgement**	
**1.5.1 Fear of being seen as a bad mum**	Bina, 2020; Brealey et al., 2010; Button et al., 2017; Forde et al., 2020; Jones et al., 2014; Lucas et al., 2019; Schmied et al., 2017; Slade et al., 2020; Sorsa et al., 2021; Viveiros & Darling, 2018; Watson et al., 2019
**1.5.2 Social services/removal of child**	Bina, 2020; Brealey et al., 2010; Button et al., 2017; Dennis & Chung-Lee, 2006; Evans et al., 2020; Forde et al., 2020; Hadfield & Wittkowski, 2017; Hewitt et al., 2009; Jones, 2019; Megnin-Viggars et al., 2015; Newman et al., 2019; Tobin et al., 2018; Watson et al., 2019
**1.5.3 Symptom minimisation**	Bina, 2020; Dennis & Chung-Lee, 2006; Forde et al., 2020; Hewitt et al., 2009; Holopainen & Hakulinen, 2019; Jones et al., 2014; Kassam, 2019; Lucas et al., 2019; Megnin-Viggars et al., 2015; Schmied et al., 2017; Staneva et al., 2015; Watson et al., 2019
**1.6 Logistics**	
**1.6.1 Childcare**	Bina, 2020; Button et al., 2017; Dennis & Chung-Lee, 2006; Hansotte et al., 2017; Morrell et al., 2016; Newman et al., 2019; Scope et al., 2017; Smith et al., 2019; Tobin et al., 2018; Watson et al., 2019
**1.6.2 Timing**	Bina, 2020; Dennis & Chung-Lee, 2006; Newman et al., 2019; Scope et al., 2017; Watson et al., 2019
**1.6.3 Location/travel**	Bina, 2020; Hansotte et al., 2017; Jones, 2019; Mollard et al., 2016; Morrell et al., 2016; Newman et al., 2019; Schmied et al., 2017; Sorsa et al., 2021; Tobin et al., 2018; Watson et al., 2019
**1.7 Social and family life**	
**1.7.1 Social isolation/support**	Bina, 2020; Giscombe et al., 2020; Holopainen & Hakulinen, 2019; Jones, 2019; Jones et al., 2014; Kassam, 2019; Lucas et al., 2019; Tobin et al., 2018; Viveiros & Darling, 2018
**1.7.1. 1 Exacerbated by mental illness**	Holopainen & Hakulinen, 2019; Jones et al., 2014; Watson et al., 2019
**1.7.2 Family and friends**	Bina, 2020; Button et al., 2017; Dennis & Chung-Lee, 2006; Forde et al., 2020; Hadfield & Wittkowski, 2017; Hansotte et al., 2017; Holopainen & Hakulinen, 2019; Jones, 2019; Lucas et al., 2019; Nilaweera et al., 2014; Schmied et al., 2017; Smith et al., 2019; Sorsa et al., 2021; Viveiros & Darling, 2018; Watson et al., 2019
**1.8 Sociodemographic factors**	
**1.8.1 Ethnicity**	Bina, 2020; Dennis & Chung-Lee, 2006; Hansotte et al., 2017; Watson et al., 2019
**1.8.2 Age**	Bina, 2020; Hansotte et al., 2017
**1.8.3 Previous experiences**	Button et al., 2017; Evans et al., 2020; Hadfield & Wittkowski, 2017; Hansotte et al., 2017; Jones, 2019; Sorsa et al., 2021; Watson et al., 2019
**1.8.4 Previous Diagnoses/symptoms**	Bina, 2020; Viveiros & Darling, 2018

**2. HCP**	
**2.1 HCP being dismissive or normalising symptoms**	Button et al., 2017; Dennis & Chung-Lee, 2006; Forde et al., 2020; Hadfield & Wittkowski, 2017; Hansotte et al., 2017; Megnin-Viggars et al., 2015; Newman et al., 2019; Sorsa et al., 2021; Watson et al., 2019
**2.2 HCP not recognising help seeking**	Bina, 2020; Button et al., 2017; Megnin-Viggars et al., 2015; Tobin et al., 2018; Watson et al., 2019
**2.3 HCP appearing too busy**	Bina, 2020; Button et al., 2017; Dennis & Chung-Lee, 2006; Hewitt et al., 2009; Megnin-Viggars et al., 2015; Slade et al., 2020; Viveiros & Darling, 2018; Watson et al., 2019
**2.3 Women's perceptions of HCPs knowledge**	
**2.3.1 Perception of HCP knowledge about PNMI**	Dennis & Chung-Lee, 2006; Forde et al., 2020; Hansotte et al., 2017; Jones, 2019; Megnin-Viggars et al., 2015; Morrell et al., 2016
**2.3.2 Perception of HCP knowledge about services/referral pathways**	Dennis & Chung-Lee, 2006; Smith et al., 2019; Viveiros & Darling, 2018
**2.4 The way the HCP delivers the care**	Button et al., 2017; Forde et al., 2020; Kassam, 2019; Megnin-Viggars et al., 2015; Nilaweera et al., 2014; Schmied et al., 2017; Slade et al., 2020; Smith et al., 2019; Sorsa et al., 2021
**2.5 HCP characteristics**	Brealey et al., 2010; Button et al., 2017; Dennis & Chung-Lee, 2006; Forde et al., 2020; Hadfield & Wittkowski, 2017; Hewitt et al., 2009; Megnin-Viggars et al., 2015; Morrell et al., 2016; Newman et al., 2019; Schmied et al., 2017; Slade et al., 2020; Staneva et al., 2015; Viveiros & Darling, 2018
**3. Interpersonal**	
**3.1 Relationship and rapport**	Bina, 2020; Brealey et al., 2010; Dennis & Chung-Lee, 2006; Hadfield & Wittkowski, 2017; Hewitt et al., 2009; Megnin-Viggars et al., 2015; Morrell et al., 2016; Scope et al., 2017; Slade et al., 2020; Tobin et al., 2018
**3.2 Language barriers**	Button et al., 2017; Dennis & Chung-Lee, 2006; Hansotte et al., 2017; Megnin-Viggars et al., 2015; Schmied et al., 2017; Smith et al., 2019
**3.3 Shared decision making**	Bina, 2020; Hadfield & Wittkowski, 2017; Megnin-Viggars et al., 2015; Morrell et al., 2016; Randall & Briscoe, 2018; Scope et al., 2017
**3.4 Communication**	Brealey et al., 2010; Forde et al., 2020; Hadfield & Wittkowski, 2017; Hewitt et al., 2009
**3.5 Information provision**	Bina, 2020; Button et al., 2017; Megnin-Viggars et al., 2015; Randall & Briscoe, 2018; Slade et al., 2020; Smith et al., 2019; Sorsa et al., 2021; Tobin et al., 2018
**4. Organisational**	
**4.1 Lack of services/Overstretched**	Bina, 2020; Button et al., 2017; Forde et al., 2020; Jones, 2019; Megnin-Viggars et al., 2015; Smith et al., 2019; Tobin et al., 2018; Viveiros & Darling, 2018
**4.2 Characteristics of service**	Bina, 2020; Button et al., 2017; Dennis & Chung-Lee, 2006; Forde et al., 2020; Jones, 2019; Newman et al., 2019; Scope et al., 2017; Viveiros & Darling, 2018; Watson et al., 2019

**4.3 Collaboration within and across services**	Bina, 2020; Megnin-Viggars et al., 2015; Newman et al., 2019; Smith et al., 2019; Watson et al., 2019
**4.4 Continuity of carer**	Brealey et al., 2010; Button et al., 2017; Hadfield & Wittkowski, 2017; Megnin-Viggars et al., 2015; Slade et al., 2020; Smith et al., 2019; Tobin et al., 2018; Viveiros & Darling, 2018; Watson et al., 2019
**4.5 Ideal care**	
**4.5.1 Screening**	
**4.5.1.1 Screening acceptability**	Brealey et al., 2010; Button et al., 2017; Evans et al., 2020; Hewitt et al., 2009; Megnin-Viggars et al., 2015; Mollard et al., 2016; Smith et al., 2019
**4.5.1.2 Wording/contents**	Brealey et al., 2010; Hewitt et al., 2009
**4.5.1.3 Delivery**	Brealey et al., 2010; Button et al., 2017; Evans et al., 2020; Hewitt et al., 2009; Smith et al., 2019
**4.5.2 Optimal treatment**	
**4.5.2.1 Opportunity to talk**	Dennis & Chung-Lee, 2006; Evans et al., 2020; Hadfield & Wittkowski, 2017; Jones, 2019; Kassam, 2019; Morrell et al., 2016; Praetorius et al., 2020; Staneva et al., 2015
**4.5.2.2 Location**	Hadfield & Wittkowski, 2017; Hansotte et al., 2017; Jones, 2019; Jones et al., 2014; Mollard et al., 2016; Newman et al., 2019; Praetorius et al., 2020; Sorsa et al., 2021
**4.5.2.3 Appropriate**	Evans et al., 2020; Megnin-Viggars et al., 2015; Scope et al., 2017; Sorsa et al., 2021
**4.5.2.4 Individualised**	Evans et al., 2020; Hadfield & Wittkowski, 2017; Megnin-Viggars et al., 2015; Morrell et al., 2016; Schmied et al., 2017; Scope et al., 2017; Slade et al., 2020; Sorsa et al., 2021; Viveiros & Darling, 2018; Watson et al., 2019
**4.5.2.5 Length**	Hadfield & Wittkowski, 2017; Morrell et al., 2016; Schmied et al., 2017; Watson et al., 2019
**4.5.2.6 Group/Peer support**	Dennis & Chung-Lee, 2006; Evans et al., 2020; Forde et al., 2020; Hadfield & Wittkowski, 2017; Jones et al., 2014; Megnin-Viggars et al., 2015; Morrell et al., 2016; Schmied et al., 2017; Scope et al., 2017; Slade et al., 2020; Tobin et al., 2018; Watson et al., 2019
**4.5.2.6.1 Validation provided by peer support**	Jones et al., 2014; Morrell et al., 2016; Schmied et al., 2017; Slade et al., 2020
**4.5.2.7 Culturally appropriate**	Button et al., 2017; Dennis & Chung-Lee, 2006; Giscombe et al., 2020; Hadfield & Wittkowski, 2017; Jones, 2019; Schmied et al., 2017; Smith et al., 2019; Tobin et al., 2018; Watson et al., 2019
**4.5.2.8 Information provision**	Forde et al., 2020; Hadfield & Wittkowski, 2017; A. Jones, 2019; Megnin-Viggars et al., 2015; Morrell et al., 2016; Schmied et al., 2017; Scope et al., 2017; Slade et al., 2020; Sorsa et al., 2021
**4.5.2.9 Medication**	Dennis & Chung-Lee, 2006; Forde et al., 2020; Jones, 2019; Sorsa et al., 2021; Tobin et al., 2018
**5. Political**	
**5.1 Immigration status**	Bina, 2020; Giscombe et al., 2020; Hansotte et al., 2017; Kassam, 2019; Lucas et al., 2019; Schmied et al., 2017

**5.2. Economic status**	Schmied et al., 2017; Tobin et al., 2018; Watson et al., 2019
**5.2.1 Healthcare costs**	Bina, 2020; Hansotte et al., 2017; Schmied et al., 2017; Tobin et al., 2018; Viveiros & Darling, 2018
**6. Societal**	
**6.1. Culture**	Bina, 2020; Brealey et al., 2010; Button et al., 2017; Dennis & Chung-Lee, 2006; Giscombe et al., 2020; Hansotte et al., 2017; Hewitt et al., 2009; Jones, 2019; Kassam, 2019; Megnin-Viggars et al., 2015; Nilaweera et al., 2014; Praetorius et al., 2020; Schmied et al., 2017; Smith et al., 2019; Sorsa et al., 2021; Staneva et al., 2015; Tobin et al., 2018; Viveiros & Darling, 2018; Watson et al., 2019; Wittkowski et al., 2014
**6.2. Maternal norms**	Bina, 2020; Brealey et al., 2010; Button et al., 2017; Dennis & Chung-Lee, 2006; Forde et al., 2020; Hadfield & Wittkowski, 2017; Hansotte et al., 2017; Hewitt et al., 2009; Holopainen & Hakulinen, 2019; Jones et al., 2014; Lucas et al., 2019; Mollard et al., 2016; Morrell et al., 2016; Newman et al., 2019; Schmied et al., 2017; Slade et al., 2020; Smith et al., 2019; Sorsa et al., 2021; Staneva et al., 2015; Viveiros & Darling, 2018
**6.3. Stigma**	Bina, 2020; Button et al., 2017; Dennis & Chung-Lee, 2006; Forde et al., 2020; Giscombe et al., 2020; Hadfield & Wittkowski, 2017; Hansotte et al., 2017; Hewitt et al., 2009; Holopainen & Hakulinen, 2019; Jones, 2019; Kassam, 2019; Lucas et al., 2019; Megnin-Viggars et al., 2015; Morrell et al., 2016; Nilaweera et al., 2014; Scope et al., 2017; Smith et al., 2019; Sorsa et al., 2021; Staneva et al., 2015; Tobin et al., 2018; Viveiros & Darling, 2018; Watson et al., 2019

*Note: highlighted yellow are themes only represented by lower-quality studies

Webb R, *et al. BMJ Open* 2023; 13:e066703. doi: 10.1136/bmjopen-2022-066703

References

1. Curry SJ, Krist AH, Owens DK, et al. Interventions to Prevent Perinatal Depression: US Preventive Services Task Force Recommendation Statement. *JAMA*. 2019;321(6):580-587. doi:10.1001/JAMA.2019.0007

2. What is the CPMHC? – CANADIAN PERINATAL MENTAL HEALTH COLLABORATIVE / COLLECTIF CANADIEN POUR LA SANTÉ MENTALE PÉRINATALE. Accessed April 12, 2022. https://cpmhc.ca/what-is-the-cpmhc/

3. PMHP - Perinatal Mental Health Project. Accessed April 12, 2022. https://pmhp.za.org/

4. Perinatal Anxiety & Depression Australia (PANDA). *Submission to the Productivity Commission Mental Health Inquiry: The Social and Economic Benefits of Improving Perinatal Mental Health*.; 2019.

5. National Institute for Health and Care Excellence (NICE). *Antenatal and Postnatal Mental Health Clinical Management and Service*.; 2014.

6. Moher D, Liberati A, Tetzlaff JM, Altman DG. Preferred reporting items for systematic reviews and meta-analyses: the PRISMA statement. *PLoS Med*. 2009;6(7):e1000097. doi:10.1016/j.jclinepi.2009.06.005

7. The Cochrane Collaboration. Cochrane data collection form for intervention reviews for RCTs only. *The cochrane Collaboration*. 2014;(April):2014. http://www.cochrane.org/

8. Shea BJ, Reeves BC, Wells GA, et al. AMSTAR 2: A critical appraisal tool for systematic reviews that include randomised or non-randomised studies of healthcare interventions, or both. *BMJ*. 2017;21:358. doi:10.1136/bmj.j4008

9. Harden A. *"Qualitative" Research, Systematic Reviews, and Evidence-Informed Policy and Practice*. University of London; 2007.

10. Carroll C, Booth A, Lloyd-Jones M. Should we exclude inadequately reported studies from qualitative systematic reviews? An evaluation of sensitivity analyses in two case study reviews. *Qualitative Health Research*. 2012;22(10):1425-1434.

11. Bina R. Predictors of postpartum depression service use: A theory-informed, integrative systematic review. *Women and Birth*. 2020;33(1):e24-e32. doi:10.1016/j.wombi.2019.01.006

12. Hansotte E, Payne SI, Babich SM. Positive postpartum depression screening practices and subsequent mental health treatment for low-income women in Western countries: A systematic literature review. *Public Health Reviews*. 2017;38(1):1-17. doi:10.1186/s40985-017-0050-y

13. Geertz C. *The Interpretation of Cultures: Selected Essays*. Basic Books; 1973.

14. Hadfield H, Wittkowski A. Women's Experiences of Seeking and Receiving Psychological and Psychosocial Interventions for Postpartum Depression: A Systematic Review and Thematic Synthesis of the Qualitative Literature. *Journal of Midwifery and Women's Health*. 2017;62(6):723-736. doi:10.1111/jmwh.12669

15. Popay J, Rogers A, Williams G. Rationale and standards for the systematic review of qualitative literature in health services research. *Qualitative Health Research*. 1998;8(3):341-351. doi:10.1177/104973239800800305

16. Thomas J, Harden A. Methods for the thematic synthesis of qualitative research in systematic reviews. *BMC Medical Research Methodology*. 2008;8(1):1-10. doi:10.1186/1471-2288-8-45

17. Ferlie EB, Shortell SM. Improving the Quality of Health Care in the United Kingdom and the United States: A Framework for Change. *Milbank Quarterly*. 2001;79(2):281-315. doi:10.1111/1468-0009.00206

18. Brealey SD, Hewitt C, Green JM, Morrell J, Gilbody S. Screening for postnatal depression - is it acceptable to women and healthcare professionals? A systematic review and meta-synthesis. *Journal of Reproductive and Infant Psychology*. 2010;28(4):328-344. doi:10.1080/02646838.2010.513045

19. Button S, Thornton A, Lee S, Ayers S, Shakespeare J. Seeking help for perinatal psychological distress: A meta-synthesis of women's experiences. *British Journal of General Practice*. 2017;67(663):e692-e699. doi:10.3399/bjgp17X692549

20. Dennis CL, Chung-Lee L. Postpartum depression help-seeking barriers and maternal treatment preferences: A qualitative systematic review. *Birth*. 2006;33(4):323-331. doi:10.1111/j.1523-536X.2006.00130.x

21. Evans K, Spiby H, Morrell J. Non-pharmacological interventions to reduce the symptoms of mild to moderate anxiety in pregnant women. A systematic review and narrative synthesis of women's views on the acceptability of and satisfaction with interventions. *Archives of Women's Mental Health*. 2020;23:11-28. doi:10.1007/s00737-018-0936-9

22. Giscombe T, Hui A, Stickley T. Perinatal mental health amongst refugee and asylum-seeking women in the UK. *Mental Health Review Journal*. 2020;25(3).

23. Hewitt CE, Gilbody S, Brealey SD, et al. Methods to identify postnatal depression in primary care: An integrated evidence synthesis and value of information analysis. *Health Technology Assessment*. 2009;13(36):1-145. doi:10.3310/hta13360

24. Holopainen A, Hakulinen T. New parents' experiences of postpartum depression: A systematic review of qualitative evidence. *JBI Database of Systematic Reviews and Implementation Reports*. 2019;17(9):1731-1769. doi:10.11124/JBISRIR-2017-003909

25. Jones CCG, Jomeen J, Hayter M. The impact of peer support in the context of perinatal mental illness: A meta-ethnography. *Midwifery*. 2014;30(5):491-498. doi:10.1016/j.midw.2013.08.003

26. Jones A. Help seeking in the perinatal period: a review of barriers and facilitators. *Soc Work Public Health*. 2019;34(7):596-605.

27. Kassam S. Understanding Experiences of Social Support as Coping Resources among Immigrant and Refugee Women with Postpartum Depression: An Integrative Literature Review. *Issues in Mental Health Nursing*. 2019;40(12):999-1011. doi:10.1080/01612840.2019.1585493

28. Lucas G, Olander EK, Ayers S, Salmon D. No straight lines - Young women's perceptions of their mental health and wellbeing during and after pregnancy: A systematic review and meta-ethnography. *BMC Women's Health*. 2019;19(1):1-17. doi:10.1186/s12905-019-0848-5

29. Megnin-Viggars O, Symington I, Howard LM, Pilling S. Experience of care for mental health problems in the antenatal or postnatal period for women in the UK: a systematic review and

meta-synthesis of qualitative research. *Archives of Women's Mental Health*. 2015;18(6):245-759. doi:10.1007/s00737-015-0548-6

30. Mollard E, Hudson DB, Ford A, Pullen C. An Integrative Review of Postpartum Depression in Rural U.S. Communities. *Archives of Psychiatric Nursing*. 2016;30(3):418-424. doi:10.1016/j.apnu.2015.12.003

31. Morrell J, Sutcliffe P, Booth A, et al. A systematic review, evidence synthesis and meta-analysis of quantitative and qualitative studies evaluating the clinical effectiveness, the cost-effectiveness, safety and acceptability of interventions to prevent postnatal depression. *Health Technology Assessment*. 2016;20(37). doi:10.3310/hta20370

32. Newman TC, Hirst J, Darwin Z. What enables or prevents women with depressive symptoms seeking help in the postnatal period? *British Journal of Midwifery*. 2019;27(4):219-227. doi:10.12968/bjom.2019.27.4.219

33. Nilaweera I, Doran F, Fisher J. Prevalence, nature and determinants of postpartum mental health problems among women who have migrated from South Asian to high-income countries: A systematic review of the evidence. *Journal of Affective Disorders*. 2014;166:213-226. doi:10.1016/j.jad.2014.05.021

34. Praetorius R, Maxwell D, Alam K. Wearing a happy mask: mother's expressions of suicidality with postpartum depression. *Social Work in Mental Health*. 2020;18(4):429-459.

35. Randall L, Briscoe L. Are women empowered to make decisions about the use of antidepressants in pregnancy? *British Journal of Midwifery*. 2018;26(5):329-335. doi:10.12968/bjom.2018.26.5.329

36. Sambrook Smith M, Lawrence V, Sadler E, Easter A. Barriers to accessing mental health services for women with perinatal mental illness: Systematic review and meta-synthesis of qualitative studies in the UK. *BMJ Open*. 2019;9(1):e024803. doi:10.1136/bmjopen-2018-024803

37. Schmied V, Black E, Naidoo N, Dahlen HG, Liamputtong P. Migrant women's experiences, meanings and ways of dealing with postnatal depression: A meta-ethnographic study. *PLoS ONE*. 2017;12(3). doi:10.1371/journal.pone.0172385

38. Scope A, Booth A, Morrell J, Sutcliffe P, Cantrell A. Perceptions and experiences of interventions to prevent postnatal depression. A systematic review and qualitative evidence synthesis. *Journal of Affective Disorders*. 2017;210:100-110. doi:10.1016/j.jad.2016.12.017

39. Slade P, Molyneux R, Watt A. A systematic review of clinical effectiveness of psychological interventions to reduce post traumatic stress symptoms following childbirth and a meta-synthesis of facilitators and barriers to uptake of psychological care. *J Affect Disord*. 2020;281:678-694.

40. Sorsa MA, Kylmä J, Bondas TE. Contemplating Help-Seeking in Perinatal Psychological Distress—A Meta-Ethnography. *Int J Environ Res Public Health*. 2021;18(10):5226.

41. Staneva AA, Bogossian F, Wittkowski A. The experience of psychological distress, depression, and anxiety during pregnancy: A meta-synthesis of qualitative research. *Midwifery*. 2015;31(6):563-573. doi:10.1016/j.midw.2015.03.015

42. Tobin CL, di Napoli P, Beck CT. Refugee and Immigrant Women's Experience of Postpartum Depression: A Meta-Synthesis. *J Transcult Nurs*. 2018;29(1):84-100. doi:10.1177/1043659616686167

43. Viveiros CJ, Darling EK. Perceptions of barriers to accessing perinatal mental health care in midwifery: A scoping review. *Midwifery*. 2019;70:106-118. doi:10.1016/j.midw.2018.11.011

44. Watson H, Harrop D, Walton E, Young A, Soltani H. A systematic review of ethnic minority women's experiences of perinatal mental health conditions and services in Europe. *PLoS ONE*. 2019;14(1):e0210587. doi:10.1371/journal.pone.0210587

45. Wittkowski A, Gardner PL, Bunton P, Edge D. Culturally determined risk factors for postnatal depression in Sub-Saharan Africa: A mixed method systematic review. *Journal of Affective Disorders*. 2014;163:115-124. doi:10.1016/j.jad.2013.12.028

46. Forde R, Peters S, Wittkowski A. Recovery from postpartum psychosis: a systematic review and metasynthesis of women's and families' experiences. *Archives of Women's Mental Health*. 2020;23(5):597-612. doi:10.1007/s00737-020-01025-z
