# Supplementary material for: Meta-review of the barriers and facilitators to women accessing perinatal mental healthcare
Source: BMJ Open. 2023 Jul 20;13(7):e066703. doi: 10.1136/bmjopen-2022-066703 (PMC10360426; doi:10.1136/bmjopen-2022-066703)
Supplement: Author's manuscript [file bmjopen-2022-066703.draft_revisions.pdf]

BMJ Open

BMJ Open is committed to open peer review. As part of this commitment we make the peer review history of every article we publish publicly available.

When an article is published we post the peer reviewers' comments and the authors' responses online. We also post the versions of the paper that were used during peer review. These are the versions that the peer review comments apply to.

The versions of the paper that follow are the versions that were submitted during the peer review process. They are not the versions of record or the final published versions. They should not be cited or distributed as the published version of this manuscript.

BMJ Open is an open access journal and the full, final, typeset and author-corrected version of record of the manuscript is available on our site with no access controls, subscription charges or pay-per-view fees (http://bmjopen.bmj.com).

If you have any questions on BMJ Open's open peer review process please email info.bmjopen@bmj.com

BMJ Open

A meta-review of the barriers and facilitators to women accessing perinatal mental health care

Journal:	*BMJ Open*
Manuscript ID	bmjopen-2022-066703
Article Type:	Original research
Date Submitted by the Author:	27-Jul-2022
Complete List of Authors:	Webb, Rebecca; City University of London, Centre for Maternal and Child Health Research<br>Uddin, Nazihah; City University of London, Centre for Maternal and Child Health Research<br>Constantinou, Georgie; City University of London, Centre for Maternal and Child Health Research<br>Ford, Elizabeth; Brighton and Sussex Medical School<br>Easter, Abigail; King's College London, Department of Women and Children's Health, School of Life Course Sciences, ; King's College London, Section of Women's Mental Health, Institute of Psychiatry, Psychology & Neuroscience<br>Shakespeare, Judy; Retired,<br>Hann, Agnes; National Childbirth Trust<br>Roberts, Nia; University of Oxford, Bodleian Health Care Libraries,<br>Alderdice, Fiona; National Perinatal Epidemiology Unit, Nuffield Department of Population Health<br>Sinesi, Andrea; University of Stirling, Nursing, Midwifery and Allied Health Professions Research Unit (NMAHP RU)<br>Coates, Rose ; City University of London, Centre for Maternal and Child Health Research<br>Hogg, Sally; Parent Infant Foundation<br>Ayers, Susan; City University, Centre for Maternal and Child Health Research<br>Study Team, The MATRIx; City University of London, Centre for Maternal and Child Health Research
Keywords:	PSYCHIATRY, Health policy < HEALTH SERVICES ADMINISTRATION & MANAGEMENT, Depression & mood disorders < PSYCHIATRY, Anxiety disorders < PSYCHIATRY

SCHOLARONE™
Manuscripts

I, the Submitting Author has the right to grant and does grant on behalf of all authors of the Work (as defined in the below author licence), an exclusive licence and/or a non-exclusive licence for contributions from authors who are: i) UK Crown employees; ii) where BMJ has agreed a CC-BY licence shall apply, and/or iii) in accordance with the terms applicable for US Federal Government officers or employees acting as part of their official duties; on a worldwide, perpetual, irrevocable, royalty-free basis to BMJ Publishing Group Ltd ("BMJ") its licensees and where the relevant Journal is co-owned by BMJ to the co-owners of the Journal, to publish the Work in this journal and any other BMJ products and to exploit all rights, as set out in our licence.

The Submitting Author accepts and understands that any supply made under these terms is made by BMJ to the Submitting Author unless you are acting as an employee on behalf of your employer or a postgraduate student of an affiliated institution which is paying any applicable article publishing charge ("APC") for Open Access articles. Where the Submitting Author wishes to make the Work available on an Open Access basis (and intends to pay the relevant APC), the terms of reuse of such Open Access shall be governed by a Creative Commons licence – details of these licences and which Creative Commons licence will apply to this Work are set out in our licence referred to above.

Other than as permitted in any relevant BMJ Author's Self Archiving Policies, I confirm this Work has not been accepted for publication elsewhere, is not being considered for publication elsewhere and does not duplicate material already published. I confirm all authors consent to publication of this Work and authorise the granting of this licence.

For peer review only

A meta-review of the barriers and facilitators to women accessing perinatal mental health care

Rebecca Webb, Nazihah Uddin, Georgina Constantinou, Elizabeth Ford, Abigail Easter, Judy Shakespeare, Agnes Hann, Nia Roberts, Fiona Alderdice, Andrea Sinesi, Rose Coates, Sally Hogg, Susan Ayers and the MATRIx Study Team

Rebecca Webb (PhD) Centre for Maternal and Child Health, City, University of London, EC1V 0HB, United Kingdom

Nazihah Uddin (MSc) Centre for Maternal and Child Health, City, University of London, EC1V 0HB, United Kingdom

Georgina Constantinou (PhD) Centre for Maternal and Child Health, City, University of London, EC1V 0HB, United Kingdom

Elizabeth Ford (PhD) Brighton & Sussex Medical School, Village Way, Falmer, BN1 9PH, United Kingdom

Abigail Easter (PhD) Department of Women and Children's Health, School of Life Course Sciences, King's College London, London, SE5 8AB, United Kingdom

Judy Shakespeare (BM, BCh) Retired GP, Oxford, OX2 7AG

Agnes Hann (PhD) NCT, Brunel House, 11 The Promenade, Clifton Down, Bristol BS8 3NG

Nia Roberts (PhD) Nuffield Department of Population Health, Bodleian Health Care Libraries, Oxford, OX3 9DU

Fiona Alderdice (Professor, PhD) National Perinatal Epidemiology Unit, Nuffield Department of Population Health, University of Oxford, Oxford, OX3 7LF

For peer review only

Andrea Sinesi (PhD) Nursing, Midwifery and Allied Health Professions Research Unit (NMAHP RU), University of Stirling, FK9 4LA, United Kingdom

Rose Coates (PhD), Centre for Maternal and Child Health, City, University of London, EC1V 0HB, United Kingdom

Sally Hogg (MA) Parent Infant Foundation, Room 202, Place2Be, 175 St John's Street, London, EC1V 4LW

Susan Ayers (Professor, PhD) Professor of Maternal and Child Health, Centre for Maternal and Child Health, City, University of London, EC1V 0HB, United Kingdom

The MATRIx Study Team: Elaine Clark, Helen Cheyne, Evelyn Frame, Simon Gilbody, Agnes Hann, Sarah McMullen Camilla Rosan, Debra Salmon, Andrea Sinesi, Clare Thompson, and Louise Williams.

*Corresponding Author: Rebecca Webb, Centre for Maternal and Child Health, City, University of London, EC1V 0HB, United Kingdom; Rebecca.Webb.2@city.ac.uk

Acknowledgements: We gratefully acknowledge the contribution of the MATRIx study team: Elaine Clark, Evelyn Frame, Simon Gilbody, Sarah McMullen, Camilla Rosan, Debra Salmon, Clare Thompson, and Louise R Williams.

Word count: 5478

Abstract

Perinatal mental health (PMH) problems are common and can have an adverse impact on women and their families. However, research suggests that a substantial proportion of women with PMH problems do not access care.

Objectives: To synthesise the results from previous systematic reviews of barriers and facilitators to women to seeking help, accessing help and engaging in PMH care, and suggest recommendations for clinical practice and policy.

Design: A meta-review of systematic reviews

Review methods: Seven databases were searched and reviews using a PRISMA (Preferred Reporting Items for Systematic Reviews and Meta Analyses) search strategy focusing on the views of women seeking help and accessing PMH care were included. Data were analysed using thematic synthesis. Assessing the Methodological Quality of Systematic Reviews (AMSTAR 2) was used to assess review methodology. To improve validity of results, a qualitative sensitivity analysis was carried out to assess whether themes remained consistent across all reviews, regardless of their quality rating.

Results: A total of 32 reviews were included. A wide range of barriers and facilitators to women accessing perinatal mental health care were identified, that mapped across a multi-level model of influential factors (individual, healthcare professional, interpersonal, organisational, political and societal) and across the care pathway (from decision to consult to receiving care). Evidence based recommendations to support the design and delivery of perinatal mental health care were produced based on identified barriers and facilitators.

Conclusion: The identified barriers and facilitators point to a complex interplay of many factors highlighting the need for an international effort to increase awareness of PMH, reduce mental

For peer review only

health stigma, and provide woman-centred, flexible care, delivered by well-trained and culturally sensitive primary care, maternity and psychiatric health professionals.

Funding: NIHR128068

Registration: PROSPERO CRD42019142854

Keywords: Perinatal mental health; Implementation; Mental health services; barriers; facilitators

For peer review only

Strengths and Weaknesses

- One strength of this meta-review is the synthesis of a large amount of information from 32 systematic reviews from many different countries
- Another strength is that the synthesis of this large amount of evidence led to the development of evidence-based recommendations for the design and delivery of care.
- A limitation is that only reviews published in academic journals and written in English language were included. Relevant reviews from other publications may have been missed.
- Another limitation is that only 10% of studies had duplicate data extraction. However, concordance was high, and it is therefore unlikely that any key themes were missed.

For peer review only

Introduction

Perinatal mental health (PMH) problems are associated adverse outcomes for women[3], their children[1], and families[2] There is a large cost to society and healthcare services with PMH problems costing the UK £8.1 billion every year[3].

Evidence-based PMH care can reduce the negative impacts to women and their families[4–6] and globally, evidence-based guidelines for PMH care exist[7]. However, research suggests access to PMH care is variable[8–11] with only 30-50% of women with PMH problems identified and less than 10% referred to specialist care[12–14]. This variable access could be due to difficulties with implementing PMH services[15] or due to help-seeking barriers experienced by women.

Multiple systematic reviews have explored women's barriers and facilitators to accessing PMH care. Each systematic review varies slightly in relation to its aim and methods making it hard to extract the information needed to design PMH services in a more accessible way. As most PMH services are designed for all women within a population, regardless of their background, a summary of all the literature is needed. A meta-review is arguably the most suitable way to do this[16,17]. Therefore, the primary aim of this research is to determine the key barriers and facilitators to women deciding to seek help, accessing help, and engaging in PMH care using a meta-review.

Method

The protocol for this review has been registered on PROSPERO (CRD42020193107) (see Appendix 1).

Patient and Public Involvement

For peer review only

This project was developed with PPI representatives from the NCT in England and the Maternal Mental Health Change Agents (MMHCA), a group of women with lived experience of PMH difficulties in Scotland.

Data sources and searches

Searches were carried out in CINAHL (1982- present); Embase (1974 – present); Medline (1946- present); PsycINFO (1806 – present), Cochrane, SCOPUS and TRIP (Turning Research into practice) Medical Database. Searches were completed on 4th August 2021 and forward and backward searches were completed by 8th September 2021. See Appendix 2 & 3 for full search syntax and results.

Study selection

Reviews were included if they used a Preferred Reporting Items for Systematic Reviews and Meta Analyses (PRISMA[18]) search strategy and focused on the views of women seeking help and accessing care for perinatal mental illness. See Appendix 4 for full inclusion criteria. Search results were imported into Endnote and duplicates and papers not meeting initial inclusion criteria (foetal distress, oxidative stress, Non-English papers) were removed by NR. The remaining studies were imported into Eppi-Reviewer 4, where results were double screened by title and abstract by two people (RW & GC). Following this, full text screening was carried out by two people (RW & GC).

Data collection process and data items

Data extraction was carried out using Microsoft Excel by RW. Double coding of extracted data was carried out for a proportion of included reviews (n = 3, 10%) by GC.

For peer review only

Critical appraisal of reviews

Methodology sections of included systematic reviews were appraised using the Assessing the Methodological Quality of Systematic Reviews (AMSTAR 2[19]). A decision was made to include reviews where confidence in results was evaluated as low and critically low because these reviews focused more on marginalised women, such as refugees, migrants, women with a low income, and women living in lower-middle income countries, to ensure the experiences of these seldom-heard women were captured. To improve the validity of results, a qualitative sensitivity analysis was carried out to assess whether themes remained consistent across all reviews, regardless of their quality rating (see Appendix 5-8, references for appendices can be found in Appendix 13).

Synthesis of results

Results were analysed by RW using a thematic synthesis[20] in NVivo and Microsoft Excel. Themes were mapped onto a multi-level framework adapted from Ferlie and Shortell's Levels of Change framework[21] and a previous systematic review on barriers and facilitators to implementation of PMH care carried out by the review authors[15]. The mapping of descriptive themes was developed deductively from the initial theoretical framework and then inductively revised as new themes emerged. The mapping of descriptive themes was discussed by all review authors before being finalised. Recommendations were developed for policy and practice based on the most cited themes. For a more detailed methodology please see Appendix 5.

Results

Review selection and review characteristics

Screening identified 32 reviews to be included in the meta-review (see Figure 1). See Appendix 9-10 for review characteristics).

For peer review only

Risk of bias within studies

Most reviews were evaluated as having low (n = 14) or critically low (n = 5) confidence with their results. The remainder had moderate (n = 8) or high (n = 5) confidence (see Appendix 11).

Synthesis of results.

Determining the barriers and facilitators to women help-seeking and accessing PMH care

A total of six overarching themes, mapped onto a multi-level framework (individual, healthcare professional (HP), interpersonal, organisational, political, and societal level factors) made up of 62 subthemes were identified (see Appendix 12). Each level of the multi-level framework (Figure 2) maps on to at least one part of the care pathway (Figure 3). Each level of the multi-level framework will be outlined below, and within each level, the most cited barriers and facilitators will be presented following the chronology of the care pathway outlined in Figure 3. Reflective quotes can be found in Table 1. Recommendations for practice and policy can be found in Table 2. It should be noted that the review draws on international evidence, and not all the factors identified will exist to the same extent in all places.

For peer review only

Table 1. Themes and reflective quotes

**Individual level**	
**Theme**	**Reflective quote**
Not understanding what perinatal mental illness is	'I don't know what postnatal depression is — how you're supposed to feel, look, or whatever. I don't know. I have no idea … what exactly is postnatal depression? What are you supposed to be doing, saying, or whatever? I don't know.' [22]
Physical factors as the cause	'I thought it was just lack of sleep and this heavy cold. I thought that after a good night's sleep it would get better, and I would be able to manage' [22]
Recognising something is wrong	'That's when I thought, you know: "Something is really wrong here, I need to go to the doctors if I'm thinking about killing myself."' [22]
Minimising symptoms	'I even went in at 3 months and I talked to a health nurse, and I just lied through my teeth because I thought, what are they going to do if they find out I can't be a good mom?' [23]
Not understanding the role of health professionals	'I don't really know what their job is. Nobody gave me, like, the parameters of this role of the health visitor [maternal and child health nurse]….' [22]
Supportive family and friends	'It was sort of my partner saying to me: "Right, if you don't go, I'm basically making you an appointment … You can't just keep feeling like this."' [22]
**Health professional level**	
**Theme**	**Reflective quote**
Health professional's appearing to not have enough time	'The health visitor said something like: "You know, in this community we have to look after a thousand and something babies." And that instilled in me the feeling, like: "Oh, they are very busy these people, and I don't have to be bothering them all the time' [22]
Health professionals being dismissive/normalising symptoms	'I did ask for support, but I didn't really get any. And the health visitor's response — "Well you seem like you're doing all right" – which kind of closes it off, doesn't it' [22]
Not recognising women's help-seeking or symptoms	'I purposely circled the things 'cos I'm struggling … the health visitor didn't get back to me, which I'm really disappointed about.' [22]
**Interpersonal level**	
**Theme**	**Reflective quote**
Trusting relationship between women and health professionals	'She's a supplement to my own mother. She's easy to talk to. I depend on her. She's not just there to take care of the baby but for the mothers too. She started a group for us new mothers.' [24]
Language barriers	'When the midwife visits, I can only speak the sentences about requesting a translator … They said that this kind of service is limited … that is what is difficult being Chinese—language

For peer review only

	barrier.' [25]
Shared decision making	'… it would have been good I think to have been listened to about the side effects. I was on a very high dose of Olanzapine [sic] and it just knocks you out and makes you into a complete zombie…' [26]
**Organisational level**	
**Theme**	**Reflective quote**
Assessment acceptability	'There's so much more that you want to say, rather than just answering quite closed questions.'[22]
Lack of child care at the service	'You have to have someone to look after your baby so who am I going to get to look after [my baby]' [22]
Lack of services, or overstretched services	'You shouldn't have to press that danger button of 'I'm gonna self-harm' or 'I'm gonna hurt my children' for someone to help you.' [26]
Lack of continuity of carer	'Every time I went to see the midwife, or…, I always had somebody different, and I don't want to tell 10 people my story.' [26]
Lack of collaboration across services	'My GP [general practitioner/family doctor] says go the HV [health visitor] and HV says go to GP. I don't know what to do, I need help, don't know where to go, or who to turn to' [27]
**Ideal care**	
**Theme**	**Reflective quote**
Culturally appropriate	'In Pakistan we only saw lady professionals, but here you don't have a choice, you have to see the men as well otherwise you don't get to see a doctor...' [28]
**Political level**	
**Theme**	**Reflective quote**
Immigration status	'Because when you're legal you can take the child to the daycare and look for a job. . . if you don't work, it's like you're dead, being alive. We want our papers so we can progress; not so we can leave or be a load to anyone, but just to work—to buy a home and give our kids a good life. . .' [29]
Economic status	'…if she has no money, how is she going to find help [with PPD]?' [30]
**Societal level**	
**Theme**	**Reflective quote**
Culture	'There is a huge stigma of being mentally ill in the public, but for us Asians there is a double disadvantage. I really fear that work will find out.' Pakistani woman living in the UK [27]

For peer review only

Maternal norms	'Mothers tend to think they should always be there. And mothers are supposed to be always rock solid, aren't they? Everyone assumes that.' [31]

Table 2. Recommendations for improving perinatal mental healthcare for women

System level factor	Theme	Recommendation
Societal	Stigma<br>Culture<br>Maternal norms	International, culturally sensitive public mental health campaigns to increase knowledge about mental illness and improve attitudes about people with mental illness[32–37]<br><br>The continuation of international policies to promote gender equality[38–40]
Political	Immigration and economic status<br>Healthcare costs	Equal rights to healthcare<br>Free healthcare<br>Laws to protect those with immigration status
Organisational	Lack of services/overstretched services<br>Characteristics of the service<br>Collaboration across services	Individualised and culturally appropriate care co-designed with women.<br>Improved funding for perinatal mental health services.<br>Improved guidance for implementing perinatal mental health care*.
Interpersonal	Continuity of carer<br>Relationship and rapport<br>Language barriers<br>Shared decision making<br>Communication<br>Information provision	Training in communication skills<br>Training in perinatal mental health to reduce stigma<br>Training in cross-cultural presentations of mental health difficulties
Healthcare professional	Characteristics<br>Time<br>Training and knowledge	Training in communication skills<br>Training in perinatal mental health to reduce stigma

For peer review only

		Training in cross-cultural presentations of mental health difficulties
Individual	Beliefs about health services<br>Beliefs about health professionals<br>Beliefs about mental illness<br>Fear of judgement<br>Logistics	Improvement of mental health literacy<br>Free access to healthcare<br>Woman-centred care

*Recommendations for implementing perinatal mental health assessment, care and treatment can be found in [15]

For peer review only

Individual level factors.

Individual level factors were identified by 25 reviews. Barriers that prevented women from *help-seeking* included **not understanding the role of health professionals** (n = 6), **not knowing what perinatal mental illness is** (n = 14) and therefore attributing it to **external causes** (e.g. job loss; n = 8), **physical causes** such as hormones (n = 9), or symptoms being a **normal part of motherhood** (n = 8). Dealing with symptoms by **ignoring them** (n = 6) or **minimising them** (n = 12) were barriers to help-seeking. **Not knowing where to go** (n = 7); **fear of being seen as a bad mum** or **social services involvement** (n = 7) and **lack of support** from family and friends (n = 9) were also barriers. A facilitator was **recognising something is wrong** (n = 9).

Barriers to *disclosing symptoms* were **not understanding health professional's role,** perceiving them as agents of social control (n = 4), fear of **social services involvement** and the removal of their child (n = 7), and fears of **being judged as a bad mum** (n = 8). The most cited barrier to women *accessing care* was **logistical reasons** (n = 13) such as travel costs, lack of childcare and timing of services. Women's *experience of care* was positively affected by **supportive family and friends** (n = 2), but a barrier was **social isolation** (n = 6).

HP related factors.

HP level factors were reported by 18 reviews. During *first contact* with women, HPs being **dismissive or normalising** women's symptoms (n = 8), **not recognising women's help-seeking or symptoms** (n = 4) and **appearing to not have enough time** (n = 3) were barriers to care. A barrier during *assessment* was the **way care was delivered** for example, in a formulaic tick-box way, or not being carried out at all (n = 3). The most reported barrier to women's *decision to disclose* was HPs **appearing to not have enough time** (n = 4) or HPs being

For peer review only

dismissive or normalising women's symptoms (n = 4). **Not recognising women's help-seeking or symptoms** (n = 2) and women's **perception of HPs knowledge of referral pathways/other services** (n = 3) were barriers to *referral. Accessing care, receiving optimal care* and *women's experiences of care* were mainly influenced by the **characteristics of HPs.** For example, HPs who were trustworthy, responsive, non-judgemental, understanding, caring, interested, warm, empathetic and positive (n = 12) were facilitators. On the other hand, unhelpful or uninterested staff were barriers (n = 2).

Interpersonal factors.

Interpersonal level factors were identified by 14 reviews. Having the opportunity to develop a **strong and trusting relationship** with a HP (n = 10) was a facilitator to women deciding to *seek help*, *disclosing* their symptoms, *accessing care*, and a positive *experience* of care. **Language difficulties** (n = 6) or **poor communication** (n = 4) between women and HPs influenced the *first contact with HPs*, *assessment*, *access,* and *provision of optimal* care.

Organisational factors.

Organisational level factors were identified by 21 reviews. The most cited barrier to *screening/assessment* was tick-box delivery (n = 6). Some women found screening tools particularly problematic if the tool was not in her first language. For example, one review reported that certain questions may not elicit true feelings from Vietnamese women living in the UK because of the shame of admitting to these[41]. Further, question Q10 on the EPDS[42] ('the thought of harming myself has occurred to me') was seen as problematic to Arabic, Vietnamese, and Black Caribbean mothers[41] living in the UK or USA, highlighting the need for culturally sensitive and relevant assessment tools.

Access to care was influenced by the **practical characteristics** (n = 5) of the organisation

For peer review only

and services offered, such as a lack of childcare facilities, hard to reach locations, and timing of appointments. A **lack of services or overstretched services** (n = 7) and a **lack of collaboration** (n = 3) across services were barriers.

In terms of **ideal care,** women reported wanting care that provided them with an opportunity to talk to someone and discuss their emotional difficulties (n = 8); some women wanted this opportunity within a peer support or group setting (n = 12) and reported that an appropriate peer group could provide them with validation for their feelings (n = 3). Care also needed to be individualised (n = 10), and be culturally sensitive (n = 8). Women also appreciated care that provided them with information about PMH difficulties (n = 5). Further, the location of the care should be easy to reach or carried out in women's homes (n = 7), and women should not be discharged too early from these services (n = 4).

Political factors.

Political factors were identified by 8 reviews and were defined as factors that governmental agencies have influence over (e.g. poverty, immigration, housing). **Immigration status** and **economic status** influenced women's *decision to consult* (n = 7) and *access to care* (n = 5). This is due to the costs of healthcare and women's fear of being deported if they access help. **Economic status** was often exacerbated by **immigration status** with women reporting not being able to get health insurance due to their immigration status (n = 4). **Economic status** also impacted *Women's experience of care* in terms of women not being able to feel any sense of wellbeing when they were unable to fulfil 'basic needs' such as 'not having enough money to make ends meet'[43] (n = 4).

Societal factors.

Societal factors were identified in 24 reviews. The main societal factors that influenced

For peer review only

women's journey along the care pathway were **culture**, societies' norms of what a "good mum" should look like (**maternal norms**), and **stigma**. All these factors intertwine and influence one another. There was only one review that only included studies from Lower Middle-Income Countries (LMICs)[44], therefore these results mainly refer to western cultures.

For women living in sub-Saharan Africa, the **cultural** tradition of confinement meant women felt unable to leave their house for fear of being shamed, acting as a barrier to *accessing care.* This was further exacerbated by the attribution of postnatal ill health to inadequate adherence to tradition[44]. Adherence to **cultural** traditions also prevented the *decision to consult* in women who had moved to western countries (n = 7). Two reviews reported that Hispanic women living in the USA felt they needed to remain strong (n = 2), feeling they needed to show that they could cope, and that stigma prevented them from seeking help due to not wanting to be seen as "crazy" or "loco" (Tobin et al., 2018[45], p.97).

Four reviews found that South Asian women living in the UK did not *consult* or *disclose* for similar **cultural** reasons e.g. the importance of fulfilling traditional gender and maternal roles, perceiving symptoms in religious terms, and stigma[22,27,45,46]:

Black African and Caribbean women living in the UK or USA were deterred from *consulting* and *disclosing* PMH problems because of the expectation of women to be strong and be able to cope (n = 4), but also the fear of what could happen if women were to seek help (n = 1). Women's cultural backgrounds highlighted the need for culturally sensitive care. The lack of this care was as a barrier to *access* (n = 3). Two reviews explained how Hispanic women living in the USA felt that language barriers, cultural insensitivity, and financial barriers were a barrier to them accessing care. Further, Jordanian women (living in Australia) spoke of being torn between their own cultural practices and Western health advice, having health professionals

placing pressure and unrealistic demands upon them to change their beliefs and behaviours[29]. Furthermore, during the *provision of care*, some women found the clash between western traditions and cultural traditions a cause of conflict, therefore an understanding of this aided the provision of good care (n = 3).

The **maternal norm** for women to show they are strong, that they can cope and be a good mother (n = 19), prevented women from *deciding to consult, disclosing, accessing care* and their *experience of care.* The **stigma** of perinatal mental illness (n = 23) prevented women *deciding to consult, disclosing symptoms, accessing car*e, and their *experience of care*.

Discussion

This meta-review identified a wide range of barriers and facilitators to women accessing PMH care, that were influential at different levels as identified in Figure 2.

Recommendations for PMH care

The results from this meta-review can be used to inform healthcare providers and policy makers on the optimal characteristics of PMH care and are summarised in Table 2. This meta-review showed a complex interplay of multi-level factors that influence women's help-seeking and access to PMH care. Societal factors such as stigma, maternal norms, and culture play a large role in women accessing care. Research suggests that public mental health campaigns can increase knowledge about mental illness and improve attitudes about people with mental illness[37]. Therefore, increasing women's, families', and the public's mental health literacy through public health campaigns, and education within the community, such as antenatal education, and at healthcare appointments, should be carried out on an international level.

Maternal norms identified in this meta-review related to women believing that they needed to be strong and show they could cope. There may be some potential to change societal

For peer review only

beliefs around maternal norms through increasing societal expectations about fathers' role in the family through more equal parental leave. For example, in countries where parental leave is more equal (e.g. Finland), the uptake of paid paternity leave is higher[47]. Changing society's maternal norms could also be done by increasing women's equality. For example, research suggests that stereotypes of what a mother or a woman should look like is beginning to change in countries where women have gained more participation in the labour force[40], and have the right to access contraception and abortion[48]. However, research is needed to corroborate these findings.

At the political level, immigration and economic status, and healthcare costs were barriers to women accessing healthcare. The results from this meta-review show how race and gender interact to influence women's experiences of the healthcare system (intersectionality)[49]. White women living within their country of birth who try to access PMH care are faced with barriers (e.g. no childcare support), but women of colour, migrant women, or migrant women of colour are faced with additional barriers (e.g. language barriers, structural/systematic discrimination). This finding is supported by research in general healthcare that has found ethnic minority and migrant women are disproportionately affected by existing barriers to accessing healthcare[50]. As found in this meta-review, these barriers include language and communication barriers, stigma, the cost of healthcare[51], and the inability to access culturally appropriate services[52]. This shows the need for equal rights to healthcare, regardless of immigration or economic status. Further, changes at the legislative level are needed to protect those who have migrated to a different country from being penalised for accessing healthcare[51].

For peer review only

At the organisational level this meta-review identified a range of factors that women viewed as ideal care. Women appreciated the opportunity to discuss screening results with HPs and for it not to be filled out as a “tick box” exercise[27]. In terms of treatment, women wanted the opportunity to talk to someone (a HP or a peer) about their difficulties[23,24,31,46,53,54]. They found peer support offered them a sense of validation which they appreciated[55]. To overcome logistical barriers, the location of services should be easily accessible, or in women’s homes[23,26,30,41]. Further, the length of treatment should be flexible and based on women’s needs. Women did not want a “one size fits all” approach but wanted individualised treatment that was culturally appropriate[22–24,26,29,45,53,56,57].

At the interpersonal and HP level the characteristics of the HPs were important, as was their communication with women. Women reported that many HPs normalised their symptoms or were dismissive of their attempts to seek help. This could be a reflection of inadequate training[58]. Within the UK, guidance states that all midwives and health visitors should receive training in order for them to identify, care for and refer perinatal women with mental health difficulties[59]. However, a synthesis of 30 studies found that midwives lack the confidence, knowledge, and training to do this[60], therefore training around mental health is important. Another key training need is cultural sensitivity and cross-cultural understanding of PMH. Some systematic reviews in this meta-review identified that women were treated in a culturally insensitive way by HPs and that women of colour were less likely to be offered treatment or be asked about their mental health. It has been suggested that training given at medical and nursing school does not do enough to reduce unconscious biases against marginalised groups, which in turn influences treatment provided by healthcare providers[61]. Improved interpretation services within perinatal mental health care may aid culturally sensitive care.

For peer review only

Further, it has been argued that the way the western world views mental illness is very ethnocentric[62] and that culture and society influences what is viewed as a mental illness[63]. This may mean that some women's attempts to seek help are missed by HPs. It is therefore crucial that cultural sensitivity and cross-cultural mental health training is provided to HPs.

In terms of individual level factors, many of these barriers can be improved through the recommendations suggested above. For example, improvement of knowledge around mental health is likely to reduce women's fear of judgement and self-stigma and increase her awareness of the symptoms she is experiencing which may encourage help-seeking[64]. Re-design of care, such as providing easily accessible healthcare may reduce the logistical barriers women experience.

Strengths and limitations

The strength of this meta-review is the synthesis of a large amount of information from 32 systematic reviews from many different countries in order to identify barriers and facilitators to women deciding to seek help, accessing help, and engaging in PMH care. This information was then used to provide recommendations for the design and delivery of care. A limitation of the methodology is that only reviews published in academic journals and written in English language were included. Relevant reviews from health services, charities, third sector organisations, and other grey literature may have been missed. Another limitation is that only 10% of studies had duplicate data extraction. However, concordance was high, and it is therefore unlikely that any key themes were missed.

Implications for future research

This review has revealed several limitations with the current evidence base on this topic. Very few systematic reviews (n = 2) addressed the severity of illness, only one review looked at

For peer review only - http://bmjopen.bmj.com/site/about/guidelines.xhtml

severe PMH difficulties[65] and most reviews (n = 24) focussed on depression. There may be different barriers for other PMH difficulties therefore future research should focus on researching the barriers and facilitators to women with disorders other than depression. Furthermore, reviews only covered the inclusion of studies carried out in 25 countries, and only one review included studies that were only carried out in LMICs[44]. More research is needed in other countries to further aid our understanding of help-seeking in women with perinatal mental illness. In addition, none of the identified reviews included studies from diverse families, including same-sex couples, and the transgender community. It is important that future research recruits more diverse populations to ensure all voices are heard. Most reviews were rated as having low or critically low quality meaning less confidence can be placed on their results. However, the qualitative sensitivity analysis found that most themes were supported in both the higher quality and lower quality reviews and including all reviews meant there was more focus on marginalised women, such as refugees, migrants, and women living in sub-Saharan Africa. This shows that the results from this meta-review can be interpreted with reasonable confidence.

Conclusion

The findings from this review point to a complex interplay of individual and system level factors across different stages of the care pathway that can influence whether women seek help and access care for perinatal mental illness. These factors should all be taken into account by policy makers to improve the identification and treatment of PMH problems. Recommendations for the design and delivery of PMH care have been produced building on the barriers and facilitators identified in this review. The identified barriers and facilitators point to the need for an international effort to reduce mental health stigma, and woman-centred, flexible care, delivered by well-trained and culturally competent HPs.

For peer review only

Figure 1. PRISMA Flow Diagram

Figure 2. The MATRIx multi-level model of barriers and facilitators to women accessing perinatal mental health care

Figure 3. Barriers and facilitators mapped onto the MATRIx care pathway

a. Contributorship statement: Rebecca Webb was involved in the design of the research and carried out screening, quality appraisal, analysis, write up of manuscript and editing of manuscript. Nazihah Uddin contributed to screening and quality appraisal of papers and provided detailed feedback on the manuscript. Georgina Constantinou contributed to screening and quality appraisal of papers provided detailed feedback on the manuscript. Elizabeth Ford was involved in the conceptualisation of the project, the design of the research, analysis, and provided detailed feedback on the manuscript. Abigail Easter was involved in the conceptualisation of the project, the design of the research, analysis, and provided detailed feedback on the manuscript. Judy Shakespeare was involved in the conceptualisation of the project, the design of the research, analysis, and provided detailed feedback on the manuscript. Agnes Hann provided PPI input and detailed feedback on the manuscript. Nia Roberts completed the literature searches. Fiona Alderdice contributed to the design of the research and provided detailed feedback on the manuscript. Andrea Sinesi contributed to the design of the research and provided detailed feedback on the manuscript. Rose Coates contributed to the design of the research and provided detailed feedback on the manuscript. Sally Hogg contributed to the design of the research and provided detailed feedback on the manuscript. Susan Ayers was the project manager, was involved in the conceptualisation of the project, the design of the research, analysis, and provided detailed feedback on the manuscript. The MATRIx study team Elaine Clark, Evelyn

For peer review only

Frame, Simon Gilbody, Sarah McMullen, Camilla Rosan, Debra Salmon, Clare Thompson, and Louise R Williams for their input throughout the project.

b. Competing interests: None

c. Funding: National Institute for Health Research. Health Services and Delivery Research Programme. NIHR128068

d. Data sharing statement: Data is available on request to the corresponding author: Rebecca.Webb.2@city.ac.uk

e. Ethics statement: This study does not involve human participants. This is an evidence synthesis, therefore approval by an ethics committee is not necessary.

For peer review only

References

1 Netsi E, Pearson RM, Murray L, *et al.* Association of persistent and severe postnatal depression with child outcomes. *JAMA Psychiatry* 2018;**75**:247–53. doi:10.1001/jamapsychiatry.2017.4363

2 Delicate A, Ayers S, Easter A, *et al.* The impact of childbirth-related post-traumatic stress on a couple's relationship: a systematic review and meta-synthesis. Journal of Reproductive and Infant Psychology. 2018. doi:10.1080/02646838.2017.1397270

3 Bauer A, Parsonage M, Knapp M, *et al.* Costs of perinatal mental health problems. London: 2014. https://www.centreformentalhealth.org.uk/publications/costs-perinatal-mental-health-problems

4 Huang L, Zhao Y, Qiang C, *et al.* Is cognitive behavioral therapy a better choice for women with postnatal depression? A systematic review and meta-analysis. *PLoS ONE* 2018;**13**:e0205243. doi:10.1371/journal.pone.0205243

5 Loughnan SA, Wallace M, Joubert AE, *et al.* A systematic review of psychological treatments for clinical anxiety during the perinatal period. *Archives of Women's Mental Health* 2018;**21**:481–90. doi:10.1007/s00737-018-0812-7

6 Molyneaux E, Telesia LA, Henshaw C, *et al.* Antidepressants for preventing postnatal depression. *Cochrane Database of Systematic Reviews* 2018;**4**:1–39. doi:10.1002/14651858.CD004363.pub3

7 World Health Organization. Improving Maternal Mental Health. Geneva: 2008.

8 Kozhimannil KB, Adams AS, Soumerai SB, *et al.* New Jersey's efforts to improve postpartum depression care did not change treatment patterns for women on medicaid. *Health Affairs* 2011;**30**:293–301. doi:10.1377/hlthaff.2009.1075

For peer review only - http://bmjopen.bmj.com/site/about/guidelines.xhtml

9 Perinatal Anxiety & Depression Australia (PANDA). Submission to the Productivity Commission Mental Health Inquiry: The Social and Economic Benefits of Improving Perinatal Mental Health. 2019.

10 Moore D, Drey N, Ayers S. Use of Online Forums for Perinatal Mental Illness, Stigma, and Disclosure: An Exploratory Model. *JMIR Mental Health* 2017;**4**:e5926. doi:10.2196/mental.5926

11 Baron EC, Hanlon C, Mall S, *et al.* Maternal mental health in primary care in five low- and middle-income countries: A situational analysis. *BMC Health Services Research* 2016;**16**. doi:10.1186/s12913-016-1291-z

12 Gavin NI, Meltzer-Brody S, Glover V, *et al.* Is population-based identification of perinatal depression and anxiety desirable? A public health perspective on the perinatal depression care continuum. In: Milgrom J, Gemmill AW, eds. *Identifying perinatal depression and anxiety: Evidence-based practice in screening, psychosocial assessment, and management*. Oxford: : Wiley-Blackwell 2015. 11–31.

13 Milgrom J, Gemmill AW. *Identifying Perinatal Depression and Anxiety*. John Wiley & Sons 2015.

14 Russell K, Ashley A, Chan G, *et al.* Maternal Mental Health – Women's Voices. *Royal Collage of Obstetricians and Ginaecologists* Published Online First: 2017. doi:10.1145/1518701.1518861

15 Webb R, Uddin N, Ford E, *et al.* Barriers and Facilitators to Implementing Perinatal Mental Health Care in the NHS and other Health and Social Care Settings: A Systematic Review. *The Lancet, Psychiatry* 2021;**8**:521–34. doi:0.1016/S2215-0366(20)30467-3.

For peer review only

16 Grant MJ, Booth A. A typology of reviews: An analysis of 14 review types and associated methodologies. *Health Information and Libraries Journal* 2009;**26**:91–108. doi:10.1111/j.1471-1842.2009.00848.x

17 Samnani SS, Vaska M, Ahmed S, *et al.* Review typology: The basic types of reviews for synthesizing evidence for the purpose of knowledge translation. *Journal of the College of Physicians and Surgeons Pakistan* 2017;**27**:635–41. doi:2724

18 Moher D, Liberati A, Tetzlaff JM, *et al.* Preferred reporting items for systematic reviews and meta-analyses: the PRISMA statement. *PLoS Med* 2009;**6**:e1000097. doi:10.1016/j.jclinepi.2009.06.005

19 Shea BJ, Reeves BC, Wells GA, *et al.* AMSTAR 2: A critical appraisal tool for systematic reviews that include randomised or non-randomised studies of healthcare interventions, or both. *BMJ* 2017;**21**:358. doi:10.1136/bmj.j4008

20 Thomas J, Harden A. Methods for the thematic synthesis of qualitative research in systematic reviews. *BMC Medical Research Methodology* 2008;**8**:1–10. doi:10.1186/1471-2288-8-45

21 Ferlie EB, Shortell SM. Improving the Quality of Health Care in the United Kingdom and the United States: A Framework for Change. *Milbank Quarterly* 2001;**79**:281–315. doi:10.1111/1468-0009.00206

22 Button S, Thornton A, Lee S, *et al.* Seeking help for perinatal psychological distress: A meta-synthesis of women's experiences. *British Journal of General Practice* 2017;**67**:e692–9. doi:10.3399/bjgp17X692549

23 Hadfield H, Wittkowski A. Women's Experiences of Seeking and Receiving Psychological and Psychosocial Interventions for Postpartum Depression: A Systematic

For peer review only

Review and Thematic Synthesis of the Qualitative Literature. *Journal of Midwifery and Women's Health* 2017;**62**:723–36. doi:10.1111/jmwh.12669

24 Morrell J, Sutcliffe P, Booth A, *et al.* A systematic review, evidence synthesis and meta-analysis of quantitative and qualitative studies evaluating the clinical effectiveness, the cost-effectiveness, safety and acceptability of interventions to prevent postnatal depression. *Health Technology Assessment* 2016;**20**. doi:10.3310/hta20370

25 Sambrook Smith M, Lawrence V, Sadler E, *et al.* Barriers to accessing mental health services for women with perinatal mental illness: Systematic review and meta-synthesis of qualitative studies in the UK. *BMJ Open* 2019;**9**:e024803. doi:10.1136/bmjopen-2018-024803

26 Megnin-Viggars O, Symington I, Howard LM, *et al.* Experience of care for mental health problems in the antenatal or postnatal period for women in the UK: a systematic review and meta-synthesis of qualitative research. *Archives of Women's Mental Health* 2015;**18**:245–759. doi:10.1007/s00737-015-0548-6

27 Sambrook Smith M, Lawrence V, Sadler E, *et al.* Barriers to accessing mental health services for women with perinatal mental illness: Systematic review and meta-synthesis of qualitative studies in the UK. *BMJ Open* 2019;**9**:e024803. doi:10.1136/bmjopen-2018-024803

28 Watson H, Harrop D, Walton E, *et al.* A systematic review of ethnic minority women's experiences of perinatal mental health conditions and services in Europe. *PLoS ONE* 2019;**14**:e0210587. doi:10.1371/journal.pone.0210587

For peer review only

29 Schmied V, Black E, Naidoo N, *et al.* Migrant women's experiences, meanings and ways of dealing with postnatal depression: A meta-ethnographic study. *PLoS ONE* 2017;**12**. doi:10.1371/journal.pone.0172385

30 Hansotte E, Payne SI, Babich SM. Positive postpartum depression screening practices and subsequent mental health treatment for low-income women in Western countries: A systematic literature review. *Public Health Reviews* 2017;**38**:1–17. doi:10.1186/s40985-017-0050-y

31 Staneva AA, Bogossian F, Wittkowski A. The experience of psychological distress, depression, and anxiety during pregnancy: A meta-synthesis of qualitative research. *Midwifery* 2015;**31**:563–73. doi:10.1016/j.midw.2015.03.015

32 Dietrich S, Mergl R, Freudenberg P, *et al.* Impact of a campaign on the public's attitudes towards depression. *Health Education Research* 2010;**25**:135–50. doi:10.1093/her/cyp050

33 Evans-Lacko S, Malcolm E, West K, *et al.* Influence of Time to Change's social marketing interventions on stigma in England 2009-2011. *British Journal of Psychiatry* 2013;**202**. doi:10.1192/bjp.bp.113.126672

34 Jorm AF, Christensen H, Griffiths KM. Changes in depression awareness and attitudes in Australia: The impact of beyondblue: The national depression initiative. *Australian and New Zealand Journal of Psychiatry* 2006;**40**:42–6. doi:10.1111/j.1440-1614.2006.01739.x

35 Jorm AF, Christensen H, Griffiths KM. The impact of beyondblue: The national depression initiative on the Australian public's recognition of depression and beliefs about treatments. *Australian and New Zealand Journal of Psychiatry* 2005;**39**:248–54. doi:10.1111/j.1440-1614.2005.01561.x

For peer review only

36 Makowski AC, Mnich EE, Ludwig J, *et al.* Changes in beliefs and attitudes toward people with depression and schizophrenia - results of a public campaign in Germany. *Psychiatry Research* 2016;**237**:271–8. doi:10.1016/j.psychres.2016.01.029

37 Sampogna G, Bakolis I, Evans-Lacko S, *et al.* The impact of social marketing campaigns on reducing mental health stigma: Results from the 2009–2014 Time to Change programme. *European Psychiatry* 2017;**40**:116–22. doi:10.1016/j.eurpsy.2016.08.008

38 Eagly AH, Nater C, Miller DI, *et al.* Gender stereotypes have changed: A cross-temporal meta-analysis of U.S. public opinion polls from 1946 to 2018. *American Psychologist* 2020;**75**:301. doi:10.1037/amp0000494

39 Lopez-Zafra E, Garcia-Retamero R. Do gender stereotypes change? The dynamic of gender stereotypes in Spain. *Journal of Gender Studies* 2012;**21**:169–83. doi:10.1080/09589236.2012.661580

40 Valiquette-Tessier SC, Gosselin J, Young M, *et al.* A Literature Review of Cultural Stereotypes Associated with Motherhood and Fatherhood. *Marriage and Family Review* 2019;**55**:299–329. doi:10.1080/01494929.2018.1469567

41 Brealey SD, Hewitt C, Green JM, *et al.* Screening for postnatal depression - is it acceptable to women and healthcare professionals? A systematic review and meta-synthesis. *Journal of Reproductive and Infant Psychology* 2010;**28**:328–44. doi:10.1080/02646838.2010.513045

42 Cox JL, Holden JM, Sagovsky R. Detection of Postnatal Depression: Development of the 10-item Edinburgh Postnatal Depression scale. *British Journal of Psychiatry* 1987;**150**:782–6. doi:10.1192/bjp.150.6.782

For peer review only

43 Lucas G, Olander EK, Ayers S, *et al.* No straight lines - Young women's perceptions of their mental health and wellbeing during and after pregnancy: A systematic review and meta-ethnography. *BMC Women's Health* 2019;**19**:1–17. doi:10.1186/s12905-019-0848-5

44 Wittkowski A, Gardner PL, Bunton P, *et al.* Culturally determined risk factors for postnatal depression in Sub-Saharan Africa: A mixed method systematic review. *Journal of Affective Disorders* 2014;**163**:115–24. doi:10.1016/j.jad.2013.12.028

45 Tobin CL, Di Napoli P, Beck CT. Refugee and Immigrant Women's Experience of Postpartum Depression: A Meta-Synthesis. *J Transcult Nurs* 2018;**29**:84–100. doi:10.1177/1043659616686167

46 Dennis CL, Chung-Lee L. Postpartum depression help-seeking barriers and maternal treatment preferences: A qualitative systematic review. *Birth* 2006;**33**:323–31. doi:10.1111/j.1523-536X.2006.00130.x

47 OECD - Social Policy Division. Parents' use of childbirth-related leave. 2019. https://www.oecd.org/els/family/PF2-2-Use-childbirth-leave.pdf

48 Centre for Reproductive Rights. The World's Abortion Laws. Resources. 2021.https://reproductiverights.org/worldabortionlaws

49 Crenshaw K. Mapping the Margins: Intersectionality, Identity Politics, and Violence against Women of Color. *Stanford Law Review* 1991;**43**:1241. doi:10.2307/1229039

50 Jayaweera H. Access to healthcare for vulnerable migrant women in England: A human security approach. *Current Sociology* 2018;**66**:273–85. doi:10.1177/0011392117736307

51 Germain S, Yong A. COVID-19 Highlighting Inequalities in Access to Healthcare in England: A Case Study of Ethnic Minority and Migrant Women. *Feminist Legal Studies* 2020;**28**:301–10. doi:10.1007/s10691-020-09437-z

For peer review only

52 Grey T, Sewell H, Shapiro G, *et al.* Mental Health Inequalities Facing U.K. Minority Ethnic Populations. *Journal of Psychological Issues in Organizational Culture* 2013;**3**:146–57. doi:10.1002/jpoc.21080

53 Evans K, Spiby H, Morrell J. Non-pharmacological interventions to reduce the symptoms of mild to moderate anxiety in pregnant women. A systematic review and narrative synthesis of women's views on the acceptability of and satisfaction with interventions. *Archives of Women's Mental Health* 2020;**23**:11–28. doi:10.1007/s00737-018-0936-9

54 Kassam S. Understanding Experiences of Social Support as Coping Resources among Immigrant and Refugee Women with Postpartum Depression: An Integrative Literature Review. *Issues in Mental Health Nursing* 2019;**40**:999–1011. doi:10.1080/01612840.2019.1585493

55 Jones CCG, Jomeen J, Hayter M. The impact of peer support in the context of perinatal mental illness: A meta-ethnography. *Midwifery* 2014;**30**:491–8. doi:10.1016/j.midw.2013.08.003

56 Scope A, Booth A, Morrell J, *et al.* Perceptions and experiences of interventions to prevent postnatal depression. A systematic review and qualitative evidence synthesis. *Journal of Affective Disorders* 2017;**210**:100–10. doi:10.1016/j.jad.2016.12.017

57 Viveiros CJ, Darling EK. Perceptions of barriers to accessing perinatal mental health care in midwifery: A scoping review. *Midwifery* 2019;**70**:106–18. doi:10.1016/j.midw.2018.11.011

58 Knaak S, Mantler E, Szeto A. Mental illness-related stigma in healthcare: Barriers to access and care and evidence-based solutions. *Healthcare Management Forum* 2017;**30**:111–6. doi:10.1177/0840470416679413

For peer review only

59 Royal College of Midwives. Specialist Mental Health Midwives: What they do and why they matter. 2018.

60 Coates D, Foureur M. The role and competence of midwives in supporting women with mental health concerns during the perinatal period: A scoping review. *Health and Social Care in the Community* 2019;**27**:e389–405. doi:10.1111/hsc.12740

61 Crear-Perry J, Maybank A, Keeys MR, *et al.* Moving towards anti-racist praxis in medicine. *The Lancet* 2020;**396**:451–3. doi:10.1016/S0140-6736(20)31543-9

62 Watters E. *Crazy Like Us: The Globalization of the American Psyche*. New York: : Free Press 2010.

63 Ventevogel P, Jordans M, Reis R, *et al.* Madness or sadness? Local concepts of mental illness in four conflict-affected African communities. *Conflict and Health* 2013;**7**:1–16. doi:10.1186/1752-1505-7-3

64 Hom MA, Stanley IH, Joiner TE. Evaluating factors and interventions that influence help-seeking and mental health service utilization among suicidal individuals: A review of the literature. *Clinical Psychology Review* 2015;**40**:28–39. doi:10.1016/j.cpr.2015.05.006

65 Forde R, Peters S, Wittkowski A. Recovery from postpartum psychosis: a systematic review and metasynthesis of women's and families' experiences. *Archives of Women's Mental Health* 2020;**23**:597–612. doi:10.1007/s00737-020-01025-z

For peer review only

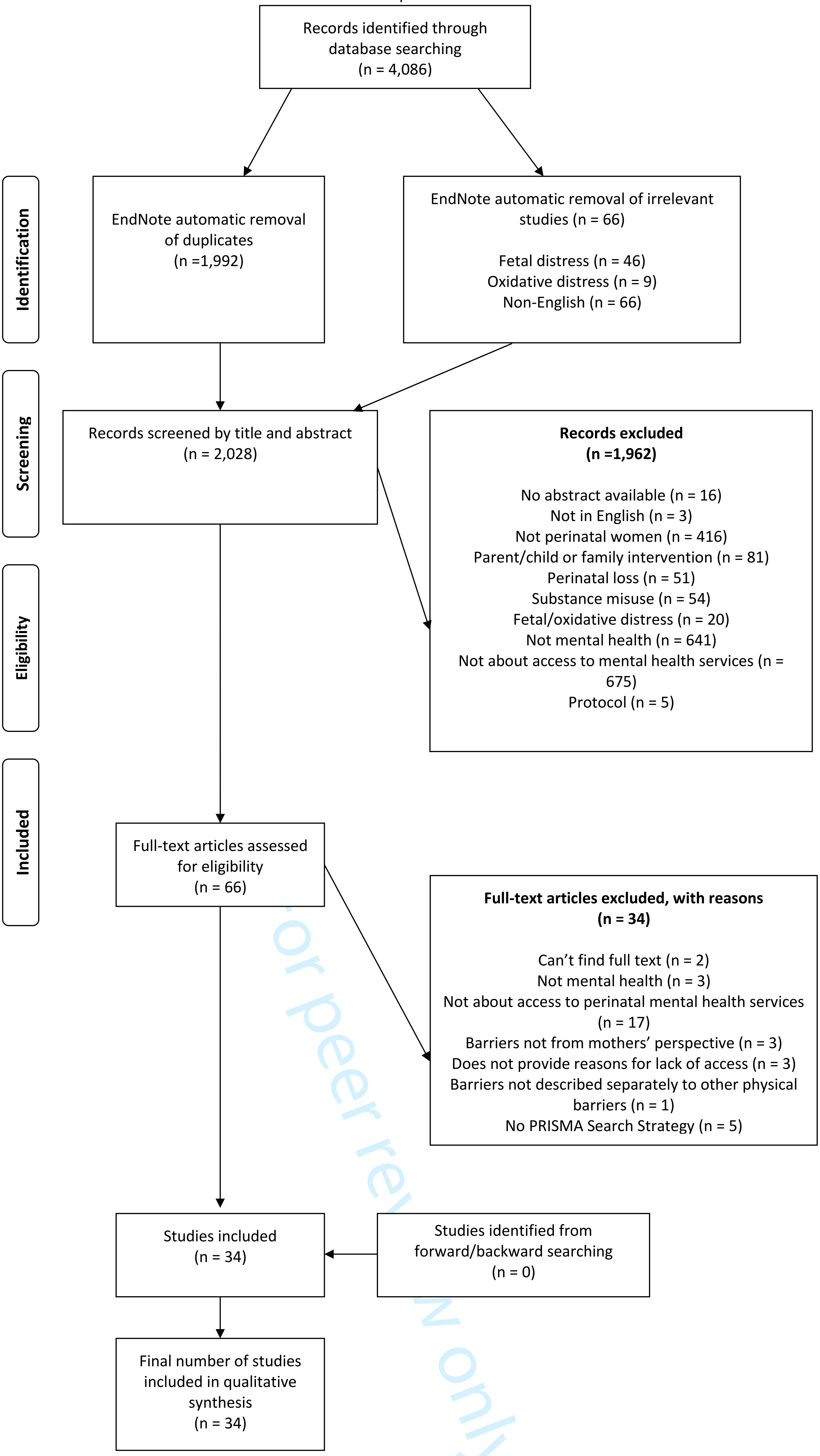

From: Moher D, Liberati A, Tetzlaff J, Altman DG, The PRISMA Group (2009). *P*referred *R*eporting *I*tems for *S*ystematic Reviews and *M*eta-*A*nalyses: The PRISMA Statement. PLoS Med 6(7): e1000097. doi:10.1371/journal.pmed1000097

For more information, visit www.prisma-statement.org.

For peer review only - http://bmjopen.bmj.com/site/about/guidelines.xhtml

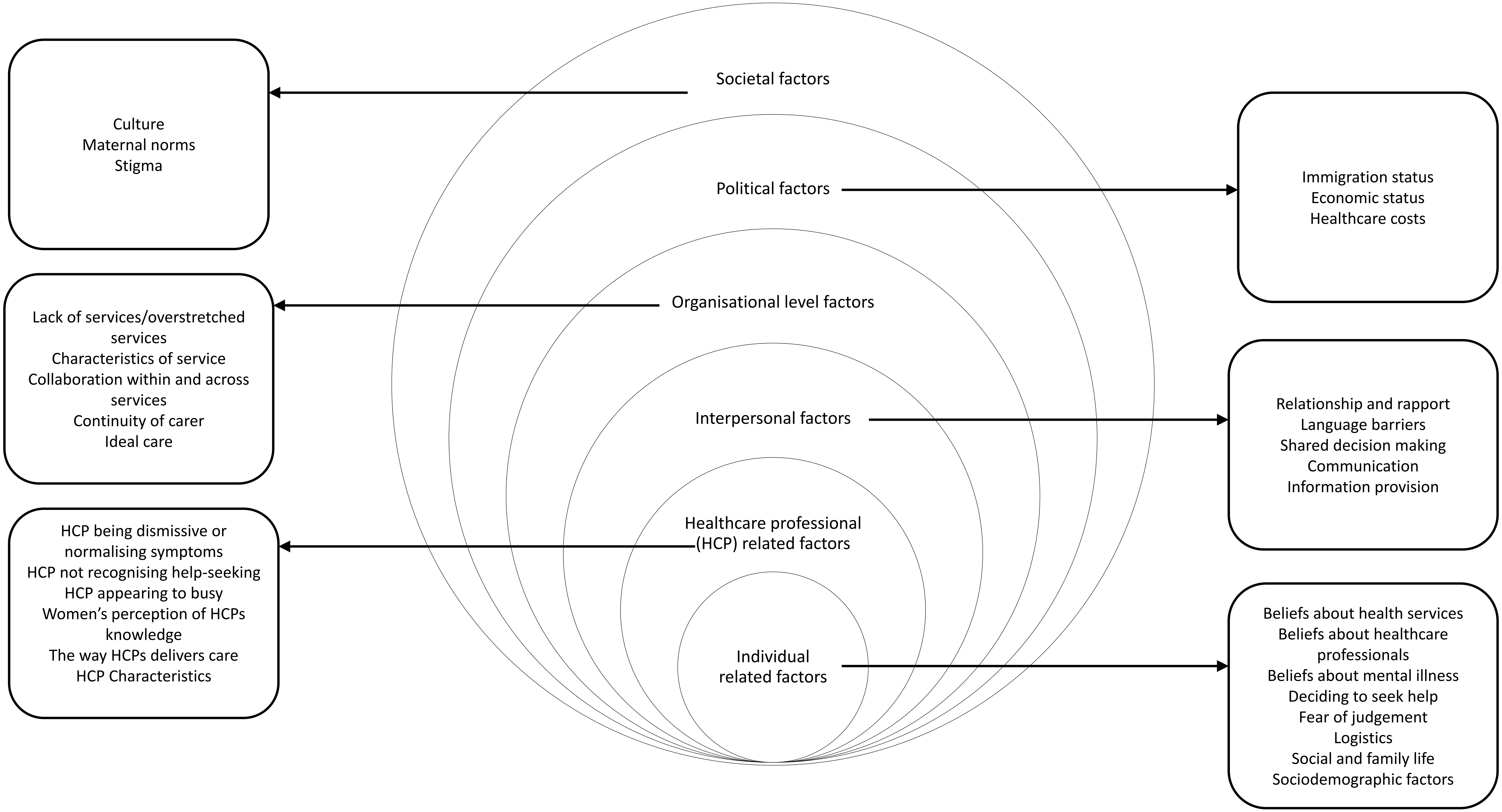
Societal factors
Culture
Maternal norms
Stigma
Political factors
Immigration status
Economic status
Healthcare costs
Organisational level factors
Lack of services/overstretched services
Characteristics of service
Collaboration within and across services
Continuity of carer
Ideal care
Interpersonal factors
Relationship and rapport
Language barriers
Shared decision making
Communication
Information provision
Healthcare professional (HCP) related factors
HCP being dismissive or normalising symptoms
HCP not recognising help-seeking
HCP appearing to busy
Women's perception of HCPs knowledge
The way HCPs delivers care
HCP Characteristics
Individual related factors
Beliefs about health services
Beliefs about healthcare professionals
Beliefs about mental illness
Deciding to seek help
Fear of judgement
Logistics
Social and family life
Sociodemographic factors

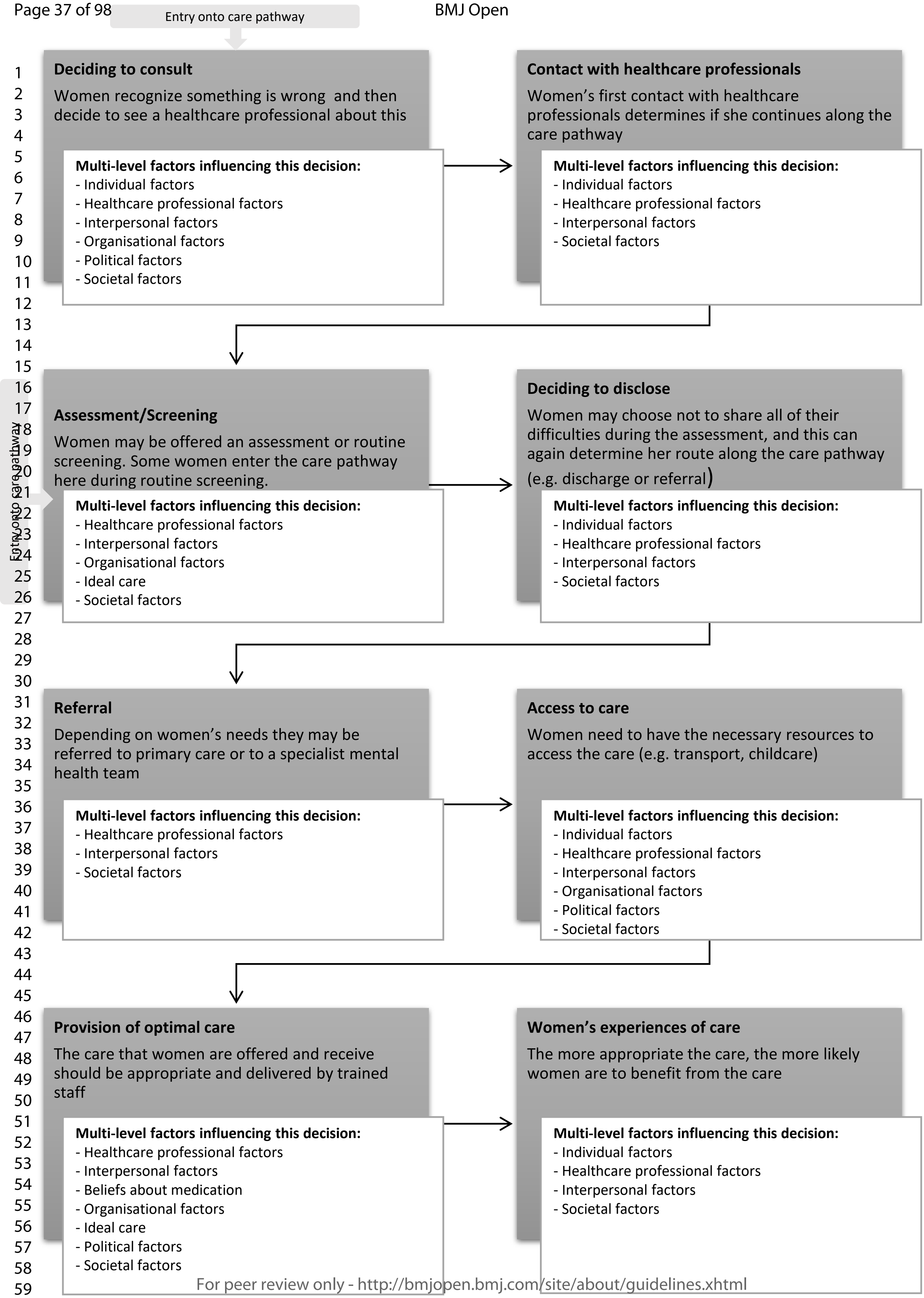

For peer review only - http://bmjopen.bmj.com/site/about/guidelines.xhtml

Note. Some parts of the pathway are redundant in health care systems where the woman can contact mental health services directly (e.g. France or via Improving Access to Psychological Therapies services in the UK). Further, the process is not always linear women might jump over certain stages.

A meta-review of the barriers to women accessing perinatal mental health care

Appendices

Contents

Appendix 1: PROSPERO Registration: CRD42019142854 2
Appendix 2: Table 1. Search terms 14
Appendix 3: Table 2. Search Results 28
Appendix 4: Table 3. Inclusion and exclusion criteria 29
Appendix 5: Detailed methodology 30
Appendix 6: Table 4. Sensitivity analysis 33
Appendix 7: *Figure 1.* Synthesis contribution vs quality appraisal criteria met 35
Appendix 8: Sensitivity analysis results 36
Appendix 9: Table 5. Summary review characteristics 37
Appendix 10: Table 6. Characteristics of included reviews 37
Appendix 11: Table 7. Quality appraisal 48
Appendix 12: Table 8. Themes 51
Appendix 13: References 56

For peer review only

Appendix 1: PROSPERO Registration: CRD42019142854

PROSPERO
International prospective register of systematic reviews

National Institute for Health Research

UNIVERSITY of York
Centre for Reviews and Dissemination

Systematic review

1. * Review title.

Give the title of the review in English

Meta-review of barriers to women accessing perinatal mental healthcare and treatment

2. Original language title.

For reviews in languages other than English, give the title in the original language. This will be displayed with the English language title.

3. * Anticipated or actual start date.

Give the date the systematic review started or is expected to start.

07/06/2020

4. * Anticipated completion date.

Give the date by which the review is expected to be completed.

28/02/2021

5. * Stage of review at time of this submission.

Tick the boxes to show which review tasks have been started and which have been completed. Update this field each time any amendments are made to a published record.

Reviews that have started data extraction (at the time of initial submission) are not eligible for inclusion in PROSPERO. If there is later evidence that incorrect status and/or completion date has been supplied, the published PROSPERO record will be marked as retracted.

This field uses answers to initial screening questions. It cannot be edited until after registration.

The review has not yet started: No

Review stage	Started	Completed
Preliminary searches	Yes	No
Piloting of the study selection process	Yes	No
Formal screening of search results against eligibility criteria	No	No
Data extraction	No	No
Risk of bias (quality) assessment	No	No
Data analysis	No	No

Provide any other relevant information about the stage of the review here.

6. * Named contact.

The named contact is the guarantor for the accuracy of the information in the register record. This may be any member of the review team.

Rebecca Webb

Email salutation (e.g. "Dr Smith" or "Joanne") for correspondence:

Dr Webb

7. * Named contact email.

Give the electronic email address of the named contact.

Rebecca.Webb.2@city.ac.uk

8. Named contact address

Give the full institutional/organisational postal address for the named contact.

Centre for Maternal and Child Health Research, School of Health Sciences, City, University of London, EC1V 0HB

9. Named contact phone number.

Give the telephone number for the named contact, including international dialling code.

(+44)07810255328

10. * Organisational affiliation of the review.

Full title of the organisational affiliations for this review and website address if available. This field may be completed as 'None' if the review is not affiliated to any organisation.

City, University of London

Organisation web address:

11. * Review team members and their organisational affiliations.

Give the personal details and the organisational affiliations of each member of the review team. Affiliation refers to groups or organisations to which review team members belong. **NOTE: email and country now MUST be entered for each person, unless you are amending a published record.**

Dr Rebecca Webb. City, University of London
Dr Elizabeth Ford. Brighton and Sussex Medical School
Dr Judy Shakespeare. Retired GP
Dr Abigail Easter. King's College London
Professor Simon Gilbody. University of York
Professor Fiona Alderdice. University of Oxford
Dr Nia Roberts. Nuffield Department of Population Health, Bodleian Health Care Libraries
Professor Debra Salmon. City, University of London

Professor Helen Cheyne. University of Stirling
Ms Clare Thompson. Maternal Mental Health Change Agents
Miss Nazihah Uddin. City, University of London
Professor Susan Ayers. City, University of London

12. * Funding sources/sponsors.

Details of the individuals, organizations, groups, companies or other legal entities who have funded or sponsored the review.

NIHR Health Services and Delivery Research Grant

Grant number(s)

State the funder, grant or award number and the date of award

NIHR128068

13. * Conflicts of interest.

List actual or perceived conflicts of interest (financial or academic).

None

14. Collaborators.

Give the name and affiliation of any individuals or organisations who are working on the review but who are not listed as review team members. **NOTE: email and country must be completed for each person, unless you are amending a published record.**

Dr Agnes Hann. National Childbirth Trust
Dr Sarah McMullen. National Childbirth Trust
Dr Rose Coates. City, University of London
Dr Camilla Rosan. Anna Freud National Centre for Children and Families
Dr Sally Hogg. Parent-Infant Partnership UK
Mr Andrea Sinesi. University of Stirling

15. * Review question.

State the review question(s) clearly and precisely. It may be appropriate to break very broad questions down into a series of related more specific questions. Questions may be framed or refined using PI(E)COS or similar where relevant.

What are the individual, interpersonal, organizational, political and social factors that prevent women with perinatal mental health problems accessing care or treatment from the NHS or other health and social care services? A review of reviews.

Objectives are to:

1. Determine the barriers and facilitators to women accessing perinatal mental health care or treatment.

2. Identify differences in barriers and facilitators across different health and social care settings.

3. Evaluate the quality of published reviews.

4. Map the geographical distribution of the evidence to establish generalisability and gaps in the evidence.

16. * Searches.

State the sources that will be searched (e.g. Medline). Give the search dates, and any restrictions (e.g. language or publication date). Do NOT enter the full search strategy (it may be provided as a link or attachment below.)

PROSPERO
International prospective register of systematic reviews

National Institute for Health Research

Systematic searches will be conducted through online databases such as: MEDLINE; PsycINFO; PubMed; Cumulative Index to Nursing and Allied Health Literature (CINAHL); EMBASE; Cochrane Library; Web of Science and Scopus. The Turning Research Into Practice (TRIP) database will also be searched. Other search strategies will include: tracking citations of key papers (forward searching); examining reference lists of key papers (backwards searching). When conducting the searches search terms will be combined using Boolean terms "OR" and "AND".

To identify papers, the following parameters will be used:

• Population: Women in the perinatal period (conception to 1 year postpartum). (Search terms will include, but are not limited to: Perinatal OR postnatal OR postpartum OR antenatal).

• Intervention: Assessment, care or treatment for perinatal mental health (search terms will include: help-seeking OR Screening OR assessment OR service* AND Access* OR implement* OR seeking OR decision OR employ OR treatment seeking OR treatment engagement)

• Outcome: Barriers and facilitators, women's experiences, health and social care professionals' experiences (search terms will include: Barriers OR drawbacks OR obstacles OR issues)

Study design: Review papers where a clear systematic search strategy is used and reported (search terms will include: meta-synthesis* OR meta-ethnograph* OR meta-study)

Papers will be selected in two stages according to PRISMA guidelines. In the first stage, titles and abstracts will be screened by one researcher for relevance to the topic. Papers that are clearly not relevant will be excluded. A random selection will be screened by a second researcher and agreement between the two will be calculated using Cohen's Kappa statistic. In the second stage, full texts for papers that appear to be relevant will be obtained and final selection made by the researcher and project management group. Again, a random selection will be screened by a second researcher and agreement between the two will be calculated.

17. URL to search strategy.

Upload a file with your search strategy, or an example of a search strategy for a specific database, (including the keywords) in pdf or word format. In doing so you are consenting to the file being made publicly accessible. Or provide a URL or link to the strategy. Do NOT provide links to your search **results**.

Alternatively, upload your search strategy to CRD in pdf format. Please note that by doing so you are consenting to the file being made publicly accessible.

Do not make this file publicly available until the review is complete

18. * Condition or domain being studied.

Give a short description of the disease, condition or healthcare domain being studied in your systematic review.

For peer review only - http://bmjopen.bmj.com/site/about/guidelines.xhtml

Mental health problems affect up to one in five women during pregnancy and the first year after birth (the perinatal period). These include anxiety, depression, stress-related conditions and adjustment disorders. This costs the UK £8.1 billion for every year of babies born. Mental health problems can have a negative effect on women, their partners and their children. They are also one of the leading causes of maternal death. It is vitally important that women who experience mental health difficulties are identified quickly and get the treatment they need. However, only about half of women with perinatal mental health problems are identified by healthcare services and even fewer receive treatment. We will therefore include the following perinatal mental health problems in our review: depression, anxiety, adjustment disorders, OCD, PTSD, psychosis, eating disorders, personality disorders.

19. * Participants/population.

Specify the participants or populations being studied in the review. The preferred format includes details of both inclusion and exclusion criteria.

The population of interest is women in the perinatal period, who are at risk of, or who are experiencing perinatal mental health problems (including, but not limited to, anxiety, depression, PTSD, adjustment disorders). No restrictions will be placed on women's age or ethnicity. If papers include multiple stakeholder views, we will include reviews where the women's responses can be separated out.

Papers will be excluded if they: are non-English publications; are text or opinion pieces, do not have a PRISMA guided search strategy, include people outside the target population (e.g. men/partners or children) where their views cannot be separated from women's views; include interventions targeted at the parent-infant, couple or family relationships; focus on perinatal loss due to the unique focus of the treatment, or focus on substance misuse which has unique challenges in terms of assessment and treatment, or focus on oxidative stress or fetal distress.

20. * Intervention(s), exposure(s).

Give full and clear descriptions or definitions of the interventions or the exposures to be reviewed. The preferred format includes details of both inclusion and exclusion criteria.

We are interested in reviews that look at women's views and experiences of accessing perinatal mental health care across the treatment pathway from deciding to consult or help seeking and disclosing symptoms to assessment, referral, care and treatment.

21. * Comparator(s)/control.

Where relevant, give details of the alternatives against which the intervention/exposure will be compared (e.g. another intervention or a non-exposed control group). The preferred format includes details of both inclusion and exclusion criteria.

Not applicable

22. * Types of study to be included.

Give details of the study designs (e.g. RCT) that are eligible for inclusion in the review. The preferred format

PROSPERO
International prospective register of systematic reviews

National Institute for Health Research

includes both inclusion and exclusion criteria. If there are no restrictions on the types of study, this should be stated.

Review papers that have used a search strategy according the PRISMA guidelines, such as systematic reviews, about access to mental health care or treatment by women in the perinatal period across all stages of the care pathway (deciding to consult, contact with healthcare professionals, assessment/screening, deciding to disclose, referral, access to treatment and provision of optimal treatment). Assessment refers to identifying women who may be at risk for perinatal mental health problems, or who have perinatal mental health problems. Care refers to supportive care or care pathways such as that provided by health professionals who provide care to women with perinatal mental health difficulties across the care pathway. Treatment refers to any active intervention, programmes or protocols to reduce women's perinatal mental health symptoms. The perinatal period is defined as from conception to 1 year postpartum. Mental health measures need to be gold standard clinical interviews or validated self-report questionnaires.

23. Context.

Give summary details of the setting or other relevant characteristics, which help define the inclusion or exclusion criteria.

As women move through pregnancy and the postnatal period, they come into contact with different services that provide women with opportunities to disclose any mental health difficulties and access perinatal mental health care. Despite the services available for women with perinatal mental health difficulties, it is estimated that half of women are not identified despite regular routine contact with these healthcare services, and still fewer receive treatment. For example, a study of postnatal depression suggested only 40% of women with postnatal depression were identified, 24% received treatment, 10% received adequate treatment, and only 3-6% of women recovered. This is likely to be due to a range of factors at individual, interpersonal, organisational and social levels, such as healthcare professionals not asking about mental health, lack of effective assessment, barriers to women seeking help or attending treatment, clinician barriers to diagnosis and treatment, lack of services to refer onto, or limited understanding of effective treatments.

Given that women accessing care or treatment might be important in preventing adverse outcomes for women and their families, we need to identify the wide range of factors that may prevent women from accessing these. The literature on why women with perinatal mental health problems do not access care or treatment is varied and some areas are more clearly synthesised than others. At present, there is no clear overview and synthesis of how these factors may operate at different levels, which is why a systematic review of reviews is needed.

24. * Main outcome(s).

Give the pre-specified main (most important) outcomes of the review, including details of how the outcome is defined and measured and when these measurement are made, if these are part of the review inclusion criteria.

For peer review only - http://bmjopen.bmj.com/site/about/guidelines.xhtml

The main aim is to determine the individual, interpersonal, organisational and social factors that prevent women accessing care or treatment. Therefore, the main outcome will be evaluation parameters for assessment, care or treatment for perinatal mental health, including barriers, facilitators, women's experiences, and health and social care professionals' experiences.

Measures of effect

Please specify the effect measure(s) for you main outcome(s) e.g. relative risks, odds ratios, risk difference, and/or 'number needed to treat.

Not applicable.

25. * Additional outcome(s).

List the pre-specified additional outcomes of the review, with a similar level of detail to that required for main outcomes. Where there are no additional outcomes please state 'None' or 'Not applicable' as appropriate to the review

None.

Measures of effect

Please specify the effect measure(s) for you additional outcome(s) e.g. relative risks, odds ratios, risk difference, and/or 'number needed to treat.

Not applicable.

26. * Data extraction (selection and coding).

Describe how studies will be selected for inclusion. State what data will be extracted or obtained. State how this will be done and recorded.

Data will be extracted from eligible reviews using a standard data extraction form in Excel or using the data extraction tool on EPPI-Reviewer. If information is missing, corresponding authors will be contacted with a request for the information. If they do not respond within 2 weeks they will be contacted again. If they do not respond within 4 weeks, missing data will not be included.

A range of data will be extracted to enable a comprehensive meta-review. Key variables will include: Authors; Year; Country; Review design; Method; Sample size and characteristics (of parents, of health care provider); Mental health problem; Outcome measures; Type of intervention(s); Methodological quality rating.

27. * Risk of bias (quality) assessment.

State which characteristics of the studies will be assessed and/or any formal risk of bias/quality assessment tools that will be used.

Quality will be assessed using the AMSTAR checklist which is a reliable and valid measure for assessing the methodological quality of systematic reviews. One reviewer will conduct the quality assessments and reliability of these ratings will be checked="checked" value="1" by a second reviewer rating a random selection of 25% of papers. If disagreements arise then all papers will be double-rated, and disagreements resolved through discussion and consensus. Where consensus cannot be reached the project management group will be consulted and make the final decision. If agreement is low, the second rater will look at a larger

For peer review only - http://bmjopen.bmj.com/site/about/guidelines.xhtml

group of papers. If meta-analyses are identified, we will assess the quality of the analysis using the Scottish Intercollegiate Guidelines Network (SIGN) evidence grading system. This system grades the risk of bias associated with a particular piece of evidence on a hierarchy from meta-analysis and RCT evidence (grade 1) down to expert opinion (grade 4), with additional indicators (++, + or -) to indicate methodological quality.

28. * Strategy for data synthesis.

Describe the methods you plan to use to synthesise data. This **must not be generic text** but should be **specific to your review** and describe how the proposed approach will be applied to your data. If meta-analysis is planned, describe the models to be used, methods to explore statistical heterogeneity, and software package to be used.

We will follow a similar strategy to McNeill et al (2012). Firstly, a table summarizing the findings will be presented. Within the table we will include the following information: authors/year; aim; search strategy; number of studies included; total number of participants; results (i.e. key barriers and facilitators identified); quality rating. We will then perform a narrative synthesis of reviews identified, discussing both barriers and facilitators to women accessing perinatal mental health assessment, care and treatment across the care pathway. Where quantitative data is included, we will carry out a narrative synthesis where we indicate both the quality of the evidence (low, medium, high) and whether it is causal or associative in nature as done by Greaves et al., (2011).

Greaves, C.J., Sheppard, K.E., Abraham, C. et al. Systematic review of reviews of intervention components associated with increased effectiveness in dietary and physical activity interventions. BMC Public Health 11, 119 (2011). https://doi.org/10.1186/1471-2458-11-119

McNeill, J., Lynn, F. & Alderdice, F. Public health interventions in midwifery: a systematic review of systematic reviews. BMC Public Health 12, 955 (2012). https://doi.org/10.1186/1471-2458-12-955

29. * Analysis of subgroups or subsets.

State any planned investigation of 'subgroups'. Be clear and specific about which type of study or participant will be included in each group or covariate investigated. State the planned analytic approach.

We will examine recommendations for healthcare practice and research separately, including which countries have a sparsity of data and recommendations for quality improvement in research methods.

30. * Type and method of review.

Select the type of review, review method and health area from the lists below.

Type of review

Cost effectiveness
No

Diagnostic
No

Epidemiologic
No

Individual patient data (IPD) meta-analysis
No

Intervention
No

Living systematic review
No

Meta-analysis
No

Methodology
No

Narrative synthesis
Yes

Network meta-analysis
No

Pre-clinical
No

Prevention
No

Prognostic
No

Prospective meta-analysis (PMA)
No

Review of reviews
Yes

Service delivery
No

Synthesis of qualitative studies
Yes

Systematic review
Yes

Other
No

Health area of the review

Alcohol/substance misuse/abuse
No

Blood and immune system
No

Cancer
No

Cardiovascular
No

Care of the elderly

For peer review only - http://bmjopen.bmj.com/site/about/guidelines.xhtml

PROSPERO
International prospective register of systematic reviews

National Institute for Health Research

No

Child health
No

Complementary therapies
No

COVID-19
No

Crime and justice
No

Dental
No

Digestive system
No

Ear, nose and throat
No

Education
No

Endocrine and metabolic disorders
No

Eye disorders
No

General interest
No

Genetics
No

Health inequalities/health equity
No

Infections and infestations
No

International development
No

Mental health and behavioural conditions
Yes

Musculoskeletal
No

Neurological
No

Nursing
No

Obstetrics and gynaecology
No

Oral health
No

For peer review only - http://bmjopen.bmj.com/site/about/guidelines.xhtml

Palliative care
No

Perioperative care
No

Physiotherapy
No

Pregnancy and childbirth
Yes

Public health (including social determinants of health)
No

Rehabilitation
No

Respiratory disorders
No

Service delivery
No

Skin disorders
No

Social care
No

Surgery
No

Tropical Medicine
No

Urological
No

Wounds, injuries and accidents
No

Violence and abuse
No

31. Language.

Select each language individually to add it to the list below, use the bin icon to remove any added in error.

English

There is not an English language summary

32. * Country.

Select the country in which the review is being carried out. For multi-national collaborations select all the countries involved.

England

33. Other registration details.

Name any other organisation where the systematic review title or protocol is registered (e.g. Campbell, or

PROSPERO
International prospective register of systematic reviews

National Institute for Health Research

The Joanna Briggs Institute) together with any unique identification number assigned by them. If extracted data will be stored and made available through a repository such as the Systematic Review Data Repository (SRDR), details and a link should be included here. If none, leave blank.

34. Reference and/or URL for published protocol.

If the protocol for this review is published provide details (authors, title and journal details, preferably in Vancouver format)

Add web link to the published protocol.

Or, upload your published protocol here in pdf format. Note that the upload will be publicly accessible.

No I do not make this file publicly available until the review is complete

Please note that the information required in the PROSPERO registration form must be completed in full even if access to a protocol is given.

35. Dissemination plans.

Do you intend to publish the review on completion?

Yes

Give brief details of plans for communicating review findings.?

We plan to publish this review in a high impact peer reviewed journal.

36. Keywords.

Give words or phrases that best describe the review. Separate keywords with a semicolon or new line. Keywords help PROSPERO users find your review (keywords do not appear in the public record but are included in searches). Be as specific and precise as possible. Avoid acronyms and abbreviations unless these are in wide use.

Perinatal mental health; Barriers; Access; Healthcare; Treatment

37. Details of any existing review of the same topic by the same authors.

If you are registering an update of an existing review give details of the earlier versions and include a full bibliographic reference, if available.

38. * Current review status.

Update review status when the review is completed and when it is published.New registrations must be ongoing so this field is not editable for initial submission.
Please provide anticipated publication date

Review_Ongoing

39. Any additional information.

Provide any other information relevant to the registration of this review.

40. Details of final report/publication(s) or preprints if available.

Leave empty until publication details are available OR you have a link to a preprint (NOTE: this field is not editable for initial submission). List authors, title and journal details preferably in Vancouver format.

Give the link to the published review or preprint.

Appendix 2: Table 1. Search terms

# ▲	MEDLINE (1946-present)<br>Searches
1	prenatal care/ or perinatal care/ or postnatal care/
2	Pregnancy/
3	Pregnant Women/
4	(pregnancy or pregnant or pre-nat* or prenat* or prepart* or prepart* or ante-nat* or antenat* or ante-part* or antepart* or peri-nat* or perinat* or peri-part* or peripart* or puerper* or post-nat* or postnat* or post-part* or postpart*).ti.
5	((parent? or mother* or maternal or father* or paternal) and (infan* or newborn? or neonat* or baby or babies)).ti.
6	1 or 2 or 3 or 4 or 5
7	mental disorders/ or exp anxiety disorders/ or exp mood disorders/ or exp "trauma and stressor related disorders"/
8	Stress, Psychological/
9	Adaptation, Psychological/
10	(mental* or psych* or anxiety or anxious or depress* or mood? or affect* or distress* or stress or trauma* or posttrauma* or post-trauma* or adjustment disorder* or phobia* or phobic or obsessive compulsive or wellbeing or well-being).ti.
11	7 or 8 or 9 or 10
12	6 and 11
13	Depression, Postpartum/
14	Pregnant Women/px [Psychology]
15	((pregnancy or pregnant or pre-nat* or prenat* or prepart* or prepart* or ante-nat* or antenat* or ante-part* or antepart* or peri-nat* or perinat* or peri-part* or peripart* or puerper* or post-nat* or postnat* or post-part* or postpart*) adj5 (mental* or psych* or anxiety or anxious or depress* or mood? or affect* or distress* or stress or trauma* or posttrauma* or post-trauma* or adjustment disorder* or phobia* or phobic or obsessive compulsive or wellbeing or well-being)).ti,ab.
16	(((parent? or mother* or maternal or father* or paternal) adj5 (infan* or newborn? or neonat* or baby or babies)) and (mental* or psych* or anxiety or anxious or depress* or mood? or affect* or distress* or stress or trauma* or posttrauma* or post-trauma* or adjustment disorder* or phobia* or phobic or obsessive compulsive or wellbeing or well-being)).ti,ab.

For peer review only - http://bmjopen.bmj.com/site/about/guidelines.xhtml

17	12 or 13 or 14 or 15 or 16
18	Mass Screening/
19	diagnosis/ or early diagnosis/
20	(screen* or detect* or diagnos* or assess* or identifi* or prevent* or prophyla*).ti.
21	psychotherapy/ or behavior therapy/ or exp cognitive behavioral therapy/
22	counseling/ or exp directive counseling/
23	exp antidepressive agents/ or exp anti-anxiety agents/
24	(intervention? or counsel* or therap* or healing or listen* support* or care or healthcare or service or medication* or drug? or antidepress* or anti-depress* or antianxiety or anti-anxiety).ti.
25	("improving access to psychological therap*" or iapt).ti,ab.
26	((mental* or psych* or anxiety or anxious or depress* or mood? or affect* or distress* or stress or trauma* or posttrauma* or post-trauma* or adjustment disorder* or phobia* or phobic or obsessive compulsive or wellbeing or well-being) adj5 (screen* or detect* or diagnos* or assess* or identifi* or prevent* or prophyla*)).ti,ab.
27	((mental* or psych* or anxiety or anxious or depress* or mood? or affect* or distress* or stress or trauma* or posttrauma* or post-trauma* or adjustment disorder* or phobia* or phobic or obsessive compulsive or wellbeing or well-being) adj5 (intervention? or counsel* or therap* or healing or listen* support* or care or healthcare or service or medication* or drug? or antidepress* or anti-depress* or antianxiety or anti-anxiety)).ti,ab.
28	18 or 19 or 20 or 21 or 22 or 23 or 24 or 25 or 26 or 27
29	17 and 28
30	Depression, Postpartum/di, dh, dt, pc, th
31	29 or 30
32	Implementation Science/ or Health Plan Implementation/
33	Program Evaluation/
34	(implement* or impact*).ti,ab.
35	(feasib* or acceptab*).ti,ab.
36	(barrier? or challenge? or obstacle? or hurdle? or obstruct* or drawback? or issue? or difficult? or promot* or support or encourag* or factor? or facilitat* or enabl* or opportunit* or engage* or assist*).ti,ab.
37	((process or project* or system*) adj5 evaluat*).ti,ab.
38	32 or 33 or 34 or 35 or 36 or 37

For peer review only

39	31 and 38
40	medline.ti,ab.
41	systematic review.pt.
42	meta-analysis.pt.
43	systematic review.ti,ab.
44	(evidence synthesis or realist synthesis or realist review).ti,ab.
45	(Qualitative and synthesis).ti,ab.
46	(meta-synthesis* or meta synthesis* or metasynthesis).ti,ab.
47	(meta-ethnograph* or metaethnograph* or meta ethnograph*).ti,ab.
48	(meta-study or metastudy or meta study).ti,ab.
49	40 or 41 or 42 or 43 or 44 or 45 or 46 or 47 or 48
50	39 and 49
51	(comment or editorial or letter or historical article).pt.
52	50 not 51
53	exp animals/ not humans/
54	52 not 53
55	limit 54 to english language
	**EMBASE (1974 – present)**
1	prenatal care/ or newborn period/ or perinatal period/ or prenatal period/
2	*Pregnancy/
3	(pregnancy or pregnant or pre-nat* or prenat* or prepart* or prepart* or ante-nat* or antenat* or ante-part* or antepart* or peri-nat* or perinat* or peri-part* or peripart* or puerper* or post-nat* or postnat* or post-part* or postpart*).ti.
4	((parent? or mother* or maternal or father* or paternal) and (infan* or newborn? or neonat* or baby or babies)).ti.
5	1 or 2 or 3 or 4
6	mental disease/ or exp anxiety disorder/ or exp mood disorder/
7	mental stress/
8	(mental* or psych* or anxiety or anxious or depress* or mood? or affect* or distress* or stress or trauma* or posttrauma* or post-trauma* or adjustment disorder* or phobia* or phobic or obsessive compulsive or wellbeing or well-being).ti.
9	6 or 7 or 8

For peer review only - http://bmjopen.bmj.com/site/about/guidelines.xhtml

For peer review only

10	5 and 9
11	exp perinatal depression/
12	((pregnancy or pregnant or pre-nat* or prenat* or prepart* or prepart* or ante-nat* or antenat* or ante-part* or antepart* or peri-nat* or perinat* or peri-part* or peripart* or puerper* or post-nat* or postnat* or post-part* or postpart*) adj5 (mental* or psych* or anxiety or anxious or depress* or mood? or affect* or distress* or stress or trauma* or posttrauma* or post-trauma* or adjustment disorder* or phobia* or phobic or obsessive compulsive or wellbeing or well-being)).ti,ab.
13	(((parent? or mother* or maternal or father* or paternal) adj5 (infan* or newborn? or neonat* or baby or babies)) and (mental* or psych* or anxiety or anxious or depress* or mood? or affect* or distress* or stress or trauma* or posttrauma* or post-trauma* or adjustment disorder* or phobia* or phobic or obsessive compulsive or wellbeing or well-being)).ti,ab.
14	10 or 11 or 12 or 13
15	mass screening/ or screening test/ or screening/
16	diagnosis/ or early diagnosis/
17	(screen* or detect* or diagnos* or assess* or identifi* or prevent* or prophyla*).ti.
18	exp counseling/ or early intervention/ or exp psychotherapy/
19	(intervention? or counsel* or therap* or healing or listen* support* or care or healthcare or service or medication* or drug? or antidepress* or anti-depress* or antianxiety or anti-anxiety).ti.
20	("improving access to psychological therap*" or iapt).ti,ab.
21	((mental* or psych* or anxiety or anxious or depress* or mood? or affect* or distress* or stress or trauma* or posttrauma* or post-trauma* or adjustment disorder* or phobia* or phobic or obsessive compulsive or wellbeing or well-being) adj5 (screen* or detect* or diagnos* or assess* or identifi* or prevent* or prophyla*)).ti,ab.
22	((mental* or psych* or anxiety or anxious or depress* or mood? or affect* or distress* or stress or trauma* or posttrauma* or post-trauma* or adjustment disorder* or phobia* or phobic or obsessive compulsive or wellbeing or well-being) adj5 (intervention? or counsel* or therap* or healing or listen* support* or care or healthcare or service or medication* or drug? or antidepress* or anti-depress* or antianxiety or anti-anxiety)).ti,ab.
23	15 or 16 or 17 or 18 or 19 or 20 or 21 or 22
24	14 and 23
25	exp perinatal depression/di, dt, pc, th
26	24 or 25

For peer review only

For peer review only - http://bmjopen.bmj.com/site/about/guidelines.xhtml

27	therapy delay/
28	exp Program Evaluation/ or Implementation Science/
29	(implement* or impact*).ti,ab.
30	(feasib* or acceptab*).ti,ab.
31	(barrier? or challenge? or obstacle? or hurdle? or obstruct* or drawback? or issue? or difficult? or promot* or support or encourag* or factor? or facilitat* or enabl* or opportunit* or engage* or assist*).ti,ab.
32	((process or project* or system*) adj5 evaluat*).ti,ab.
33	27 or 28 or 29 or 30 or 31 or 32
34	26 and 33
35	medline.tw.
36	"systematic review"/
37	meta-analysis/
38	systematic review.ti,ab.
39	(evidence synthesis or realist synthesis or realist review).ti,ab.
40	(Qualitative and synthesis).ti,ab.
41	(meta-synthesis* or meta synthesis* or metasynthesis).ti,ab.
42	(meta-ethnograph* or metaethnograph* or meta ethnograph*).ti,ab.
43	(meta-study or metastudy or meta study).ti,ab.
44	35 or 36 or 37 or 38 or 39 or 40 or 41 or 42 or 43
45	34 and 44
46	(editorial or letter or note or conference*).pt.
47	45 not 46
48	(exp animals/ or nonhuman/) not human/
49	47 not 48
50	limit 49 to english language
	**PSYCHINFO (1806 – present)**
1	prenatal care/ or postnatal period/ or antepartum period/ or intrapartum period/ or perinatal period/
2	Pregnancy/

For peer review only - http://bmjopen.bmj.com/site/about/guidelines.xhtml

3	(pregnancy or pregnant or pre-nat* or prenat* or prepart* or prepart* or ante-nat* or antenat* or ante-part* or antepart* or peri-nat* or perinat* or peri-part* or peripart* or puerper* or post-nat* or postnat* or post-part* or postpart*).ti.
4	((parent? or mother* or maternal or father* or paternal) and (infan* or newborn? or neonat* or baby or babies)).ti.
5	1 or 2 or 3 or 4
6	mental disorders/ or exp affective disorders/ or exp anxiety disorders/ or exp "stress and trauma related disorders"/
7	psychological stress/
8	Emotional Adjustment/
9	(mental* or psych* or anxiety or anxious or depress* or mood? or affect* or distress* or stress or trauma* or posttrauma* or post-trauma* or adjustment disorder* or phobia* or phobic or obsessive compulsive or wellbeing or well-being).ti.
10	6 or 7 or 8 or 9
11	5 and 10
12	postpartum depression/ or postpartum psychosis/
13	((pregnancy or pregnant or pre-nat* or prenat* or prepart* or prepart* or ante-nat* or antenat* or ante-part* or antepart* or peri-nat* or perinat* or peri-part* or peripart* or puerper* or post-nat* or postnat* or post-part* or postpart*) adj5 (mental* or psych* or anxiety or anxious or depress* or mood? or affect* or distress* or stress or trauma* or posttrauma* or post-trauma* or adjustment disorder* or phobia* or phobic or obsessive compulsive or wellbeing or well-being)).ti,ab.
14	(((parent? or mother* or maternal or father* or paternal) adj5 (infan* or newborn? or neonat* or baby or babies)) and (mental* or psych* or anxiety or anxious or depress* or mood? or affect* or distress* or stress or trauma* or posttrauma* or post-trauma* or adjustment disorder* or phobia* or phobic or obsessive compulsive or wellbeing or well-being)).ti,ab.
15	11 or 12 or 13 or 14
16	screening/ or exp health screening/ or exp screening tests/
17	diagnosis/
18	(screen* or detect* or diagnos* or assess* or identifi* or prevent* or prophyla*).ti.
19	treatment/ or exp cognitive behavior therapy/ or exp cognitive techniques/ or exp counseling/ or mindfulness-based interventions/ or exp psychotherapy/
20	(intervention? or counsel* or therap* or healing or listen* support* or care or healthcare or service or medication* or drug? or antidepress* or anti-depress* or antianxiety or anti-anxiety).ti.
21	("improving access to psychological therap*" or iapt).ti,ab.

For peer review only - http://bmjopen.bmj.com/site/about/guidelines.xhtml

22	((mental* or psych* or anxiety or anxious or depress* or mood? or affect* or distress* or stress or trauma* or posttrauma* or post-trauma* or adjustment disorder* or phobia* or phobic or obsessive compulsive or wellbeing or well-being) adj5 (screen* or detect* or diagnos* or assess* or identifi* or prevent* or prophyla*)).ti,ab.
23	((mental* or psych* or anxiety or anxious or depress* or mood? or affect* or distress* or stress or trauma* or posttrauma* or post-trauma* or adjustment disorder* or phobia* or phobic or obsessive compulsive or wellbeing or well-being) adj5 (intervention? or counsel* or therap* or healing or listen* support* or care or healthcare or service or medication* or drug? or antidepress* or anti-depress* or antianxiety or anti-anxiety)).ti,ab.
24	16 or 17 or 18 or 19 or 20 or 21 or 22 or 23
25	15 and 24
26	treatment barriers/
27	exp Program Evaluation/
28	(implement* or impact*).ti,ab.
29	(feasib* or acceptab*).ti,ab.
30	(barrier? or challenge? or obstacle? or hurdle? or obstruct* or drawback? or issue? or difficult? or promot* or support or encourag* or factor? or facilitat* or enabl* or opportunit* or engage* or assist*).ti,ab.
31	((process or project* or system*) adj5 evaluat*).ti,ab.
32	26 or 27 or 28 or 29 or 30 or 31
33	25 and 32
34	medline.ti,ab.
35	exp "Systematic Review"/
36	Meta Analysis/
37	systematic review.ti,ab.
38	(evidence synthesis or realist synthesis or realist review).ti,ab.
39	(Qualitative and synthesis).ti,ab.
40	(meta-synthesis* or meta synthesis* or metasynthesis).ti,ab.
41	(meta-ethnograph* or metaethnograph* or meta ethnograph*).ti,ab.
42	(meta-study or metastudy or meta study).ti,ab.
43	34 or 35 or 36 or 37 or 38 or 39 or 40 or 41 or 42
44	33 and 43

For peer review only - http://bmjopen.bmj.com/site/about/guidelines.xhtml

45	(comment reply or editorial or letter or "review book" or "review media" or "review software other").dt.
46	44 not 45
47	limit 46 to english language
	**CINAHL (1982 – present)**
S30	S28 NOT S29
S29	S23 AND S27 Limiters - English Language; Publication Type: Book Review, Commentary, Editorial, Letter
S28	S23 AND S27
S27	S24 OR S25 OR S26
S26	TX ("evidence synthesis" or "realist synthesis" or "realist review") OR TX (Qualitative and synthesis) OR TX ((meta-synthesis* or "meta synthesis*" or metasynthesis) OR TX (meta-ethnograph* or metaethnograph* or "meta ethnograph*") OR TX (meta-study or metastudy or "meta study")
S25	TI (medline or "systematic review") OR AB (medline or "systematic review")
S24	(MH "Systematic Review") OR (MH "Meta Analysis") OR (MH "Meta Synthesis")
S23	S19 AND S22
S22	S20 OR S21
S21	((implement* or impact*)) OR ((implement* or impact*)) OR ((feasib* or acceptab*)) OR ((feasib* or acceptab*)) OR ((barrier? or challenge? or obstacle? or hurdle? or obstruct* or drawback? or issue? or difficult? or promot* or support or encourag* or factor? or facilitat* or enabl* or opportunit* or engage* or assist*)) OR ((barrier? or challenge? or obstacle? or hurdle? or obstruct* or drawback? or issue? or difficult? or promot* or support or encourag* or factor? or facilitat* or enabl* or opportunit* or engage* or assist*)) OR (((process or project* or system*) N5 evaluat*)) OR (((process or project* or system*) N5 evaluat*))
S20	(MH "Implementation Science") OR (MH "Program Development+")
S19	S17 OR S18
S18	(MH "Depression, Postpartum/DI/DH/DT/PC/TH") OR (MH "Postpartum Psychosis/DI/DH/DT/TH/PC")
S17	S11 AND S16
S16	S12 OR S13 OR S14 OR S15

For peer review only - http://bmjopen.bmj.com/site/about/guidelines.xhtml

S15	TI ((intervention? or counsel* or therap* or healing or listen* support* or care or healthcare or service or medication* or drug? or antidepress* or anti-depress* or antianxiety or anti-anxiety)) OR TI (("improving access to psychological therap*" or iapt)) OR AB (("improving access to psychological therap*" or iapt)) OR TI (((mental* or psych* or anxiety or anxious or depress* or mood? or affect* or distress* or stress or trauma* or posttrauma* or post-trauma* or adjustment disorder* or phobia* or phobic or obsessive compulsive or wellbeing or well-being) N5 (screen* or detect* or diagnos* or assess* or identifi* or prevent* or prophyla*))) OR AB (((mental* or psych* or anxiety or anxious or depress* or mood? or affect* or distress* or stress or trauma* or posttrauma* or post-trauma* or adjustment disorder* or phobia* or phobic or obsessive compulsive or wellbeing or well-being) N5 (screen* or detect* or diagnos* or assess* or identifi* or prevent* or prophyla*))) OR TI (((mental* or psych* or anxiety or anxious or depress* or mood? or affect* or distress* or stress or trauma* or posttrauma* or post-trauma* or adjustment disorder* or phobia* or phobic or obsessive compulsive or wellbeing or well-being) N5 (intervention? or counsel* or therap* or healing or listen* support* or care or healthcare or service or medication* or drug? or antidepress* or anti-depress* or antianxiety or anti-anxiety))) OR AB (((mental* or psych* or anxiety or anxious or depress* or mood? or affect* or distress* or stress or trauma* or posttrauma* or post-trauma* or adjustment disorder* or phobia* or phobic or obsessive compulsive or wellbeing or well-being) N5 (intervention? or counsel* or therap* or healing or listen* support* or care or healthcare or service or medication* or drug? or antidepress* or anti-depress* or antianxiety or anti-anxiety)))
S14	(MH "Antidepressive Agents+")
S13	(MH "Psychotherapy+") OR (MH "Cognitive Therapy+") OR (MH "Counseling+")
S12	(MH "Diagnosis") OR (MH "Early Diagnosis") OR (MH "Health Screening")
S11	S8 OR S9 OR S10

For peer review only

For peer review only - http://bmjopen.bmj.com/site/about/guidelines.xhtml

S10	TI (((pregnancy or pregnant or pre-nat* or prenat* or prepart* or prepart* or ante-nat* or antenat* or ante-part* or antepart* or peri-nat* or perinat* or peri-part* or peripart* or puerper* or post-nat* or postnat* or post-part* or postpart*) N5 (mental* or psych* or anxiety or anxious or depress* or mood? or affect* or distress* or stress or trauma* or posttrauma* or post-trauma* or "adjustment disorder*" or phobia* or phobic or "obsessive compulsive" or wellbeing or well-being))) OR AB (((pregnancy or pregnant or pre-nat* or prenat* or prepart* or prepart* or ante-nat* or antenat* or ante-part* or antepart* or peri-nat* or perinat* or peri-part* or peripart* or puerper* or post-nat* or postnat* or post-part* or postpart*) N5 (mental* or psych* or anxiety or anxious or depress* or mood? or affect* or distress* or stress or trauma* or posttrauma* or post-trauma* or "adjustment disorder*" or phobia* or phobic or "obsessive compulsive" or wellbeing or well-being))) OR TI ((((parent? or mother* or maternal or father* or paternal) N5 (infan* or newborn? or neonat* or baby or babies)) and (mental* or psych* or anxiety or anxious or depress* or mood? or affect* or distress* or stress or trauma* or posttrauma* or post-trauma* or "adjustment disorder*" or phobia* or phobic or "obsessive compulsive" or wellbeing or well-being))) AND AB ((((parent? or mother* or maternal or father* or paternal) N5 (infan* or newborn? or neonat* or baby or babies)) and (mental* or psych* or anxiety or anxious or depress* or mood? or affect* or distress* or stress or trauma* or posttrauma* or post-trauma* or "adjustment disorder*" or phobia* or phobic or "obsessive compulsive" or wellbeing or well-being)))
S9	(MH "Depression, Postpartum") OR (MH "Postpartum Psychosis") OR (MH "Expectant Mothers/PF")
S8	S4 AND S7
S7	S5 OR S6
S6	TI mental* or psych* or anxiety or anxious or depress* or mood? or affect* or distress* or stress or trauma* or posttrauma* or post-trauma* or adjustment disorder* or phobia* or phobic or obsessive compulsive or wellbeing or well-being
S5	(MH "Mental Disorders") OR (MH "Anxiety Disorders+") OR (MH "Affective Disorders+") OR (MH "Stress Disorders, Post-Traumatic+") OR (MH "Adaptation, Psychological")
S4	S1 OR S2 OR S3
S3	TI ((pregnancy or pregnant or pre-nat* or prenat* or prepart* or prepart* or ante-nat* or antenat* or ante-part* or antepart* or peri-nat* or perinat* or peri-part* or peripart* or puerper* or post-nat* or postnat* or post-part* or postpart*) OR TI (((parent? or mother* or maternal or father* or paternal) and (infan* or newborn? or neonat* or baby or babies)))
S2	(MH "Expectant Mothers")
S1	(MH "Prenatal Care") OR (MH "Postnatal Period") OR (MH "Pregnancy") OR (MH "Puerperium")
	**SCOPUS**

For peer review only - http://bmjopen.bmj.com/site/about/guidelines.xhtml

11	(((TITLE (pregnancy OR pregnant OR pre-nat* OR prenat* OR prepart* OR prepart* OR ante-nat* OR antenat* OR ante-part* OR antepart* OR peri-nat* OR perinat* OR peri-part* OR peripart* OR puerper* OR post-nat* OR postnat* OR post-part* OR postpart*) AND TITLE (mental* OR psych* OR anxiety OR anxious OR depress* OR mood* OR affect* OR distress* OR stress OR trauma* OR posttrauma* OR post-trauma* OR {adjustment disorder*} OR phobia* OR phobic OR {obsessive compulsive} OR wellbeing OR well-being))) AND ((TITLE-ABS-KEY ((screen* OR detect* OR diagnos* OR assess* OR identifi* OR prevent* OR prophyla*)) OR TITLE-ABS-KEY ((intervention* OR counsel* OR therap* OR healing OR listen* AND support* OR care OR healthcare OR service OR medication* OR drugs OR drug OR antidepress* OR anti-depress* OR antianxiety OR anti-anxiety)) OR TITLE-ABS-KEY (("improving access to psychological therap*" OR iapt)))) AND ((TITLE-ABS-KEY (implement* OR impact* OR feasib* OR acceptab*) OR TITLE-ABS-KEY ((barrier* OR challenge* OR obstacle* OR hurdle* OR obstruct* OR drawback* OR issue* OR difficult* OR promot* OR support OR encourag* OR factor? OR facilitat* OR enabl* OR opportunit* OR engage* OR assist*)) OR TITLE-ABS-KEY ("process evaluation*" OR "project evaluation*" OR "systems evaluation*" OR "system evaluation*")))) AND ((TITLE-ABS-KEY ("systematic review" OR meta-analys*) OR TITLE-ABS-KEY ("evidence synthesis" OR "realist synthesis" OR "realist review" OR meta-synthesis* OR "meta synthesis" OR metasynthesis OR meta-ethnograph* OR metaethnograph* OR "meta ethnograph*" OR meta-study OR metastudy OR "meta study") OR TITLE-ABS-KEY (qualitative AND synthesis))) AND (LIMIT-TO (LANGUAGE , "English"))
10	(((TITLE (pregnancy OR pregnant OR pre-nat* OR prenat* OR prepart* OR prepart* OR ante-nat* OR antenat* OR ante-part* OR antepart* OR peri-nat* OR perinat* OR peri-part* OR peripart* OR puerper* OR post-nat* OR postnat* OR post-part* OR postpart*) AND TITLE (mental* OR psych* OR anxiety OR anxious OR depress* OR mood* OR affect* OR distress* OR stress OR trauma* OR posttrauma* OR post-trauma* OR {adjustment disorder*} OR phobia* OR phobic OR {obsessive compulsive} OR wellbeing OR well-being))) AND ((TITLE-ABS-KEY ((screen* OR detect* OR diagnos* OR assess* OR identifi* OR prevent* OR prophyla*)) OR TITLE-ABS-KEY ((intervention* OR counsel* OR therap* OR healing OR listen* AND support* OR care OR healthcare OR service OR medication* OR drugs OR drug OR antidepress* OR anti-depress* OR antianxiety OR anti-anxiety)) OR TITLE-ABS-KEY (("improving access to psychological therap*" OR iapt)))) AND ((TITLE-ABS-KEY (implement* OR impact* OR feasib* OR acceptab*) OR TITLE-ABS-KEY ((barrier* OR challenge* OR obstacle* OR hurdle* OR obstruct* OR drawback* OR issue* OR difficult* OR promot* OR support OR encourag* OR factor? OR facilitat* OR enabl* OR opportunit* OR engage* OR assist*)) OR TITLE-ABS-KEY ("process evaluation*" OR "project evaluation*" OR "systems evaluation*" OR "system evaluation*")))) AND ((TITLE-ABS-KEY ("systematic review" OR meta-analys*) OR TITLE-ABS-KEY ("evidence synthesis" OR "realist synthesis" OR "realist

For peer review only - http://bmjopen.bmj.com/site/about/guidelines.xhtml

	review" OR meta-synthesis* OR "meta synthesis" OR metasynthesis OR meta-ethnograph* OR metaethnograph* OR "meta ethnograph*" OR meta-study OR metastudy OR "meta study") OR TITLE-ABS-KEY (qualitative AND synthesis)))
9	(TITLE-ABS-KEY ("systematic review" OR meta-analys*) OR TITLE-ABS-KEY ("evidence synthesis" OR "realist synthesis" OR "realist review" OR meta-synthesis* OR "meta synthesis" OR metasynthesis OR meta-ethnograph* OR metaethnograph* OR "meta ethnograph*" OR meta-study OR metastudy OR "meta study") OR TITLE-ABS-KEY (qualitative AND synthesis))
8	((TITLE (pregnancy OR pregnant OR pre-nat* OR prenat* OR prepart* OR prepart* OR ante-nat* OR antenat* OR ante-part* OR antepart* OR peri-nat* OR perinat* OR peri-part* OR peripart* OR puerper* OR post-nat* OR postnat* OR post-part* OR postpart*) AND TITLE (mental* OR psych* OR anxiety OR anxious OR depress* OR mood* OR affect* OR distress* OR stress OR trauma* OR posttrauma* OR post-trauma* OR {adjustment disorder*} OR phobia* OR phobic OR {obsessive compulsive} OR wellbeing OR well-being))) AND ((TITLE-ABS-KEY ((screen* OR detect* OR diagnos* OR assess* OR identifi* OR prevent* OR prophyla*)) OR TITLE-ABS-KEY ((intervention* OR counsel* OR therap* OR healing OR listen* AND support* OR care OR healthcare OR service OR medication* OR drugs OR drug OR antidepress* OR anti-depress* OR antianxiety OR anti-anxiety)) OR TITLE-ABS-KEY (("improving access to psychological therap*" OR iapt)))) AND ((TITLE-ABS-KEY (implement* OR impact* OR feasib* OR acceptab*) OR TITLE-ABS-KEY ((barrier* OR challenge* OR obstacle* OR hurdle* OR obstruct* OR drawback* OR issue* OR difficult* OR promot* OR support OR encourag* OR factor? OR facilitat* OR enabl* OR opportunit* OR engage* OR assist*)) OR TITLE-ABS-KEY ("process evaluation*" OR "project evaluation*" OR "systems evaluation*" OR "system evaluation*")))
7	(TITLE-ABS-KEY (implement* OR impact* OR feasib* OR acceptab*) OR TITLE-ABS-KEY ((barrier* OR challenge* OR obstacle* OR hurdle* OR obstruct* OR drawback* OR issue* OR difficult* OR promot* OR support OR encourag* OR factor? OR facilitat* OR enabl* OR opportunit* OR engage* OR assist*)) OR TITLE-ABS-KEY ("process evaluation*" OR "project evaluation*" OR "systems evaluation*" OR "system evaluation*"))
	**COCHRANE**
#1	MeSH descriptor: [Prenatal Care] explode all trees
#2	MeSH descriptor: [Perinatal Care] explode all trees

For peer review only

For peer review only - http://bmjopen.bmj.com/site/about/guidelines.xhtml

#3	MeSH descriptor: [Pregnancy] this term only
#4	MeSH descriptor: [Pregnant Women] explode all trees
#5	(pregnancy or pregnant or pre-nat* or prenat* or prepart* or prepart* or ante-nat* or antenat* or ante-part* or antepart* or peri-nat* or perinat* or peri-part* or peripart* or puerper* or post-nat* or postnat* or post-part* or postpart*):ti OR (((parent* or mother* or maternal or father* or paternal) and (infan* or newborn* or neonat* or baby or babies))):ti
#6	#1 or #2 or #3 or #4 or #5
#7	MeSH descriptor: [Mental Disorders] this term only
#8	MeSH descriptor: [Anxiety Disorders] explode all trees
#9	MeSH descriptor: [Mood Disorders] explode all trees
#10	MeSH descriptor: [Trauma and Stressor Related Disorders] explode all trees
#11	MeSH descriptor: [Stress, Psychological] explode all trees
#12	MeSH descriptor: [Adaptation, Psychological] this term only
#13	(mental* or psych* or anxiety or anxious or depress* or mood* or affect* or distress* or stress or trauma* or posttrauma* or post-trauma* or adjustment disorder* or phobia* or phobic or obsessive compulsive or wellbeing or well-being):ti
#14	#7 or #8 or #9 or #10 or #11 or #12 or #13
#15	#6 and #14
#16	MeSH descriptor: [Depression, Postpartum] explode all trees
#17	MeSH descriptor: [Pregnant Women] explode all trees and with qualifier(s): [psychology - PX]
#18	(((pregnancy or pregnant or pre-nat* or prenat* or prepart* or prepart* or ante-nat* or antenat* or ante-part* or antepart* or peri-nat* or perinat* or peri-part* or peripart* or puerper* or post-nat* or postnat* or post-part* or postpart*) NEAR/5 (mental* or psych* or anxiety or anxious or depress* or mood* or affect* or distress* or stress or trauma* or posttrauma* or post-trauma* or adjustment disorder* or phobia* or phobic or obsessive compulsive or wellbeing or well-being))):ti,ab,kw
#19	((((parent or parents or mother* or maternal or father* or paternal) NEAR/5 (infan* or newborn* or neonat* or baby or babies)) and (mental* or psych* or anxiety or anxious or depress* or mood* or affect* or distress* or stress or trauma* or posttrauma* or post-trauma* or adjustment disorder* or phobia* or phobic or obsessive compulsive or wellbeing or well-being))):ti,ab,kw
#20	#15 or #16 or #17 or #18 or #19
#21	MeSH descriptor: [Implementation Science] explode all trees

For peer review only

#22	MeSH descriptor: [Health Plan Implementation] explode all trees
#23	MeSH descriptor: [Program Evaluation] explode all trees
#24	(implement* or impact*):ti,ab,kw OR (feasib* or acceptab*):ti,ab,kw OR ((barrier* or challenge* or obstacle* or hurdle* or obstruct* or drawback* or issue* or difficult* or promot* or support or encourag* or factor* or facilitat* or enabl* or opportunit* or engage* or assist*)):ti,ab,kw OR (((process or project* or system*) NEAR/5 evaluat*)):ti,ab,kw
#25	#21 or #22 or #23 or #24
#26	#20 and #25

For peer review only

For peer review only - http://bmjopen.bmj.com/site/about/guidelines.xhtml

Appendix 3: Table 2. Search Results

Database:	Interface:	Coverage:	Date:	Hits:
CINAHL	EBSCOHost	1982-present	04/08/2021	759
Cochrane Database of Systematic Reviews	Cochrane Library, Wiley	Issue 8 of 12, August 2021	04/08/2021	384
Embase	OvidSP	1974-present	04/08/2021	1081
Medline	OvidSP	1946-present	04/08/2021	977
PsycINFO	OvidSP	1806-present	04/08/2021	286
Scopus	Elsevier		04/08/2021	599
**Total:**				**4086**
Duplicates:				1992
Papers excluded:				66
**Final total:**				**2045**

**Papers excluded:** available if needed	
Fetal distress	46
Oxidative stress	9
Non-English	11
	66

Included - 27th May 2020	1671
Included - 4th August 2021	374

For peer review only - http://bmjopen.bmj.com/site/about/guidelines.xhtml

Appendix 4: Table 3. Inclusion and exclusion criteria

Category	Criteria
Population	Women in the perinatal period (conception to 12 months after birth) experiencing mental health problems, who may or may not have decided to seek help, accessed help, or engaged in PNMH care. PNMH care was defined as assessment, referrals, and/or treatment/intervention programmes) from health or social care services. Conception to 12 months after birth was chosen as the target population because this is the period that many perinatal mental health services cover [1–5].<br><br>Reviews were excluded if they were not conducted on the target population (e.g., men/partners, healthcare professionals), focused on substance misuse (which has unique challenges in terms of assessment and treatment), did not focus on the mental health of perinatal women.
Outcome	Barriers and facilitators (defined as any individual, healthcare professional, interpersonal, organisational, political, or societal factors that women believed impeded (barriers) or aided (facilitators) them) to seeking, accessing, or engaging in help for PNMH problems. Studies were included if they made descriptive statements about barriers and facilitators to women deciding to seek help, accessing help, and engaging in PNMH care. These descriptions had to be drawn from perinatal women's experiences.<br><br>Reviews were excluded if they did not examine any barriers/facilitators regarding seeking help, accessing help and engaging in PNMH care.
Design	Only systematic reviews were included. Studies that did not use a clearly reported PRISMA search strategy [6]were excluded.<br><br>Non-English publications were also excluded.

For peer review only

Appendix 5: Detailed methodology

Protocol and registration

The protocol for this review has been registered on PROSPERO (CRD42020193107) (see appendix for full protocol).

Eligibility criteria

Studies with the following characteristics were eligible for inclusion in the review: Population: Women in the perinatal period (conception to 12 months after birth) experiencing mental health problems, who may or may not have decided to seek help, accessed help, or engaged in PNMH care. PNMH care was defined as assessment, referrals, and/or treatment/intervention programmes) from health or social care services. Conception to 12 months after birth was chosen as the target population because this is the period that many perinatal mental health services cover [1–5].

Outcome: Barriers and facilitators (defined as any individual, healthcare professional, interpersonal, organisational, political, or societal factors that women believed impeded (barriers) or aided (facilitators) them) to seeking, accessing, or engaging in help for PNMH problems.

Studies were included if they made descriptive statements about barriers and facilitators to women deciding to seek help, accessing help, and engaging in PNMH care. These descriptions had to be drawn from perinatal women's experiences. Only systematic reviews were included. Studies that did not use a clearly reported PRISMA search strategy [6]were excluded. Reviews were also excluded if they were not conducted on the target population (e.g., men/partners, healthcare professionals), focused on substance misuse (which has unique challenges in terms of assessment and treatment), did not focus on the mental health of perinatal women, did not examine any barriers/facilitators regarding seeking help, accessing help and engaging in PNMH care, and were non-English publications.

Information sources

Searches were carried out in CINAHL (1982- present); Embase (1974 – present); Medline (1946-present); and PsycINFO (1806 – present), Cochrane, SCOPUS and TRIP (Turning Research into practice) Medical Database. The date of the last search was 28th May 2020. Forward and backward searches of included studies were carried out and completed by the 26th June 2020. Searches were updated on 4th August 2021 and forward and backward searches of new included studies were completed by 8th September 2021.

Search

Pre-planned searches were carried out using both MeSH terms (i.e. prenatal care/anxiety/ diagnosis) and search terms were combined with Boolean operators "OR" and "AND" (e.g. pregnancy OR perinatal OR postnat* AND anxiety OR depress* OR wellbeing AND intervention? OR counsel* OR support OR identifi* AND OR barrier? OR facilitate*).

Review selection

Search results were imported into Endnote and duplicates and papers not meeting initial inclusion criteria (foetal distress, oxidative stress, Non-English papers) were removed by NR. The remaining studies were imported into Eppi-Reviewer 4, where results were double screened by title and abstract by two people (RW & GC). An additional proportion (n = 166, ~7%) of titles and abstracts were triple screened by NU. Decisions to include or exclude were concordant between RW & GC in 94.2% of cases and between RW & NU in 99.39% of cases. Disagreements were discussed and resolved by NU, GC, and RW by applying the relevant inclusion criteria.

For peer review only - http://bmjopen.bmj.com/site/about/guidelines.xhtml

Once title and abstract screening was complete, full text screening was carried out by two people (RW & GC). An additional proportion (n = 9, ~10%) were triple screened by NU. Decisions to include or exclude were concordant between RW & GC in 91.4% of cases and between RW & NU in 100% of cases.

Data collection process and data items

Data extraction was carried out using Microsoft Excel by RW. Each paper was read in full, and relevant parts of the text input into the relevant part of the spreadsheet. Review methodology was copied onto one sheet and results onto another to aid analysis. Double coding of extracted data was carried out for a proportion of included reviews (n = 3, 10%) by GC. Data extraction matched in 85% of cases.

The data that were extracted was guided by the Cochrane Systematic Review for Intervention Data Collection form [7] and the Assessing the Methodological Quality of Systematic Reviews (AMSTAR 2;[8]) Data collected included the following: Review Characteristics (year of publication, author(s), design, aim, search strategy, inclusion/exclusion criteria, screening/study selection, data extraction, quality assessment, analysis methods); Sample Characteristics (Number of studies included, total number of participants, participant demographics); Assessment/Care/Treatment Characteristics (Healthcare setting, intervention description, screening description) and outcomes (barriers and facilitators).

Critical appraisal of reviews

Methodology sections of included systematic reviews were appraised using the AMSTAR 2. Critical domains in the appraisal of systematic reviews according to AMSTAR 2 include protocol registration, adequacy of literature search, justification of study exclusion, risk of bias, appropriateness of meta-analytic methods, consideration of risk of bias when interpreting results, and assessment of publication bias. If more than one critical domain is not met (critical flaw), a systematic review should be evaluated as having critically low confidence in the results of the review. One critical flaw means reviews should be evaluated as low confidence [8].

Given that all studies in this review were qualitative, the AMSTAR 2 items related to meta-analysis were not relevant and were thus removed. Further, given the debate in the literature regarding the appropriateness of conducting risk of bias assessments on qualitative research, we downgraded the items relating to risk of bias from being critical flaws, to flaws. Quality appraisal of all studies was carried out by NU and RW. Ratings were concordant in 90% of cases.

A decision was made to continue to include reviews where confidence in results was evaluated as low and critically low because these reviews focused more on marginalised women, such as refugees, migrants, women with a low income, and women living in LMIC, to ensure the experiences of these seldom-heard women were captured. To improve the validity of results, a qualitative sensitivity analysis was carried out to assess whether themes remained consistent across all reviews, regardless of their quality rating. The methods proposed by Harden[9] and Carroll et al[10] was followed and therefore sensitivity analysis was carried out in two ways: (1) synthesis contribution; (2) evidence of adequate description of themes.

To examine whether higher quality studies contributed more to the themes, a measure of "synthesis contribution" was calculated for each study (as done by Harden, 2007[9]) by dividing the number of barriers and facilitators identified by that study, by the total number of barriers and facilitators identified in the review overall. For example, the findings from Bina (2020) [11]contributed to 31 out of 62 themes, giving this review a synthesis contribution score of 50% (see appendix, Table 3). Each study's synthesis contribution scores was plotted against the number of quality criteria the study

For peer review only - http://bmjopen.bmj.com/site/about/guidelines.xhtml

met (see appendix, Figure 1). Statistical analysis (Pearson's correlation) was used to help interpret the plots. To examine whether removing lower quality reviews influenced the number of themes, themes that were only supported by lower quality reviews were identified (see appendix, Table 6).

To examine whether removing lower quality reviews influenced the description of themes, data were assessed for "thickness" or "thinness" (as done by Carroll et al., 2012[10]). Thin description refers to a set of statements (e.g. "O'Mahoney et al. found that women also felt that providers were downplaying the symptoms they were experiencing", Hansotte et al., 2017, [12]p.12), whereas thick description provides the context of experience and circumstances [13] (e.g. "Having symptoms dismissed or attributed to factors other than PPD by health care professionals led to women 'remaining silent.' Some women perceived that their difficulties would only be taken seriously when there were concerns about risk of harm to themselves or the infant. One woman said, 'I kept going to this doctor and he used to give me a pep talk and send me home…'", Hadfield & Wittkowski, 2017[14], p.732). It is argued that the extent to which a text provides a thick description shows evidence of the authenticity of the results [15].

Synthesis of results

Results were analysed by RW using a thematic synthesis [16] in NVivo and Microsoft Excel. First, line by line data coding of statements referring to facilitators or barriers to accessing PNMH care from the results section of each paper was carried out. Next, codes were revisited and assigned a descriptive theme based on their meaning and content. Themes were developed and revised as each review was re-read. Once all codes had been assigned into themes, the themes were mapped onto a multi-level framework adapted from Ferlie and Shortell's Levels of Change framework [17] and a previous systematic review on barriers and facilitators to implementation of PNMH care carried out by the review authors. The mapping of descriptive themes was developed deductively from the initial theoretical framework and then inductively revised as new themes emerged. The mapping of descriptive themes was discussed by all review authors before being finalised. A decision was taken to analyse all reviews together, regardless of the specific aims or individual inclusion criteria. This is because the majority of the reviews (n = 27) included studies carried out in a wide range of countries/settings. This, therefore, made it difficult to parse apart reviews based on sample characteristics, settings, or country of included studies.

For peer review only - http://bmjopen.bmj.com/site/about/guidelines.xhtml

Appendix 6: Table 4. Sensitivity analysis

Study	Number of themes	Unique synthesis contribution	Overall synthesis contribution (all themes)
Bina, 2020[11]	31	3.03030303	50
Brealey et al., 2010[18]	13	1.515151515	20.96774194
Button et al., 2017[19]	26	0	41.93548387
Dennis & Chung-Lee, 2006[20]	28	0	45.16129032
Evans et al., 2020[21]	8	0	12.90322581
Giscombe et al., 2020[22]	6		9.677419355
Hadfield & Wittkowski, 2017[14]	25	0	40.32258065
Hansotte et al., 2017[12]	19	1.515151515	30.64516129
Hewitt et al., 2009[23]	13	0	20.96774194
Holopainen & Hakulinen, 2019[24]	6	0	9.677419355
Jones et al., 2014[25]	10	0	16.12903226
Jones, 2019[26]	19	0	30.64516129
Kassam, 2019[27]	8	0	12.90322581
Lucas et al., 2019[28]	9	0	14.51612903
Megnin-Viggars et al., 2015[29]	26	0	41.93548387
Mollard et al., 2016[30]	5	1.515151515	8.064516129
Morrell et al., 2016[31]	16	0	25.80645161
Newman et al., 2019[32]	13	0	20.96774194
Nilaweera et al., 2014[33]	6	0	9.677419355
Praetorius et al., 2020[34]	3		4.838709677
Randall & Briscoe, 2018[35]	2	0	3.225806452
Sambrook-Smith et al., 2019[36]	19	0	30.64516129
Schmied et al., 2017[37]	27	1.515151515	43.5483871
Scope et al., 2017[38]	13	0	20.96774194
Slade et al., 2020[39]	15		24.19354839
Sorsa et al., 2021[40]	19		30.64516129
Staneva et al., 2015[41]	11	0	17.74193548

For peer review only - http://bmjopen.bmj.com/site/about/guidelines.xhtml

Tobin et al., 2018[42]	19	0	30.64516129
Viveiros & Darling, 2018[43]	16	1.515151515	25.80645161
Watson et al., 2019[44]	28	0	45.16129032
Wittkowski et al., 2014[45]	2	1.515151515	3.225806452

For peer review only

Appendix 7: *Figure 1.* Synthesis contribution vs quality appraisal criteria met

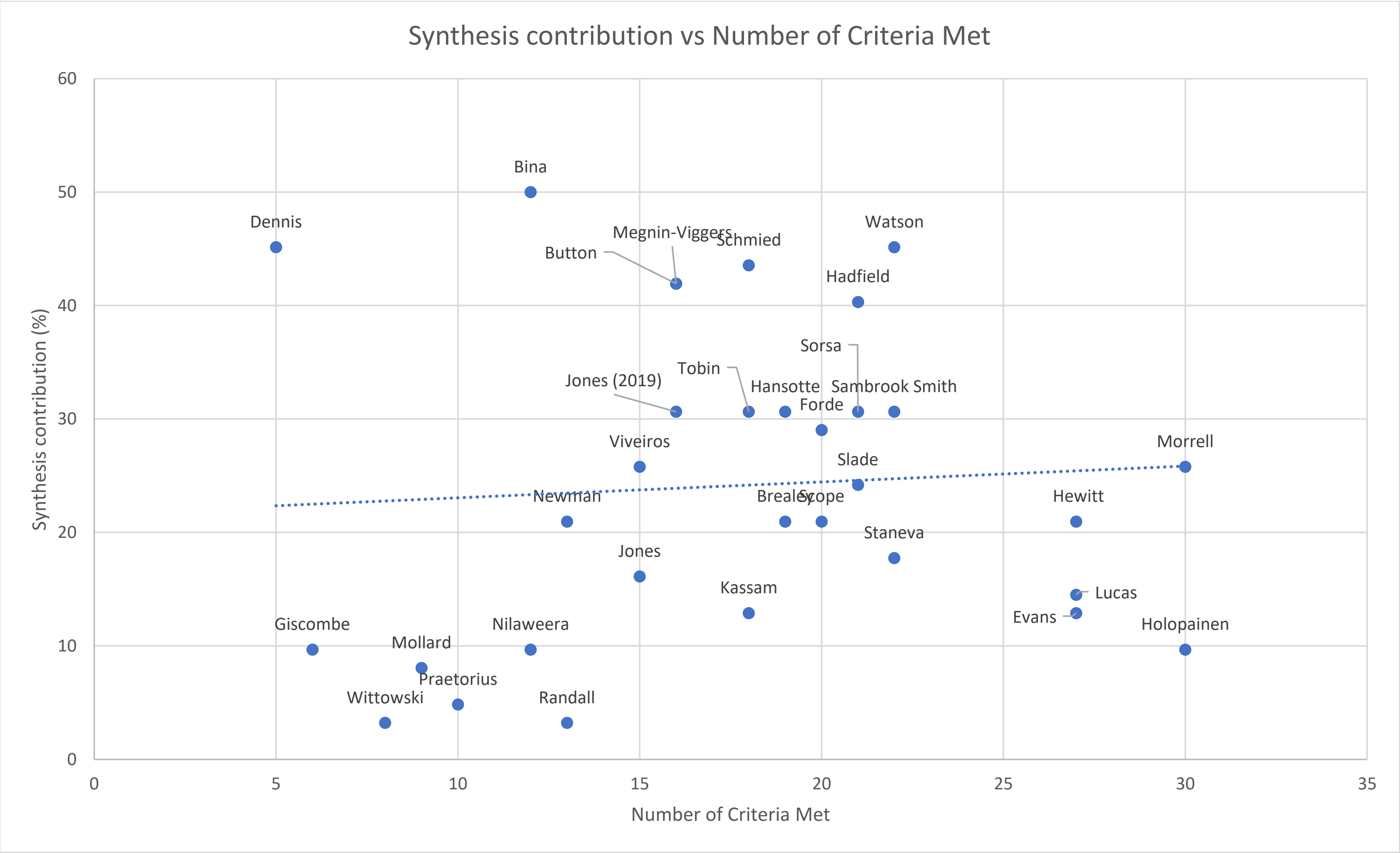

35

Appendix 8: Sensitivity analysis results

Synthesis contribution. There was no correlation between synthesis contribution and the number of criteria each review met (r = .142, p = .437; see appendix, Figure 1). Furthermore, only four themes (**cultural/spiritual** causes of mental illness, **age, previous diagnoses,** and **appropriateness of care)** were only identified by lower quality studies showing the majority of themes (58 out of 62; 93.55%) were supported by both higher quality and lower quality papers.

Richness of data. The removal of lower quality papers meant that the theme **language barriers** lost some of its richness. For example, it led to the removal of quotes expressing frustration from women whose first language was not English:

> '. . .you don't know where to go, what to do, who to trust, especially when you are coming by yourself. . . you believe that you speak English, but when you get here you realize that you don't.' [37]
>
> 'Sometimes when you have a baby, a woman comes from the hospital. Bengali girls don't come with the midwife, we don't understand what they say, we just sit there staring at their faces.' [19]

The removal of lower quality papers from the theme **fear of being seen as a bad mum** led to the loss of richness of data including the removal of a quote from women who have migrated from their country of birth:

> 'Back home, if someone has this problem, everyone gossips, you get this feeling that people are not dealing with you normally or as if you are abnormal almost. . .' [37]

Lastly, the removal of lower quality studies meant important information was removed from the **characteristics of service** theme, such as women feeling services prioritise physical needs (n = 2), lack information about screening guidelines (n = 2), and the logistics (e.g. location, time of appointments) of the care (n = 3)

For peer review only - http://bmjopen.bmj.com/site/about/guidelines.xhtml

Appendix 9: Table 5. Summary review characteristics

Characteristics	Range; Mean (M), Median (Mdn); Interquartile Range (IQR)
Year	2006-2022; M = 2017, Mdn = 2018; IQR = 2016-2019
No. studies included in each review	4-40; M = 16, Mdn = 13, IQR = 9-19
No. women included in each review	95-85,190; M = 5080; Mdn = 463; IQR = 226-1,715
Countries	N = 24

Appendix 10: Table 6. Characteristics of included reviews

	Review details			Participant details					
**Author & Year**	**N studies about women (Total N)**<br><br>**Years (Range)**	**Inclusion criteria**	**Country of studies**	**N**<br><br>**M (SD)**	**Age**	**Perinatal period**	**Ethnicity**	**Mental illness**	**Socioeconomic status**
Bina (2020)[11]	31 (35)<br><br>1993-2018	Service use for postpartum depression or "distress" from women (and HCP) perspectives	11 countries (4 LMIC)	7219<br><br>232.9 (414.7)	NR	2 weeks - up to 3 years postnatal	6 studies specified recruitmen t of migrant women or women of colour[(EA; H; Ar)]	Depressive symptoms, emotional difficulties or current/past diagnosis of mood disorder	2 studies recruited women with low income.<br>1 study recruited women using Medicaid.
Brealey et al. (2010)[18]	13 (16)<br><br>1997-2007	Acceptability to women (and HCP) of screening to identify women with increased risk of	5 countries (all HIC)	1715<br><br>131.9 (253.06)	24-34<br>M (n = 8) = 29.63	First antenatal appointmen t – 12 months after birth	2 studies recruited women of colour [(B; EA; Ar)]	Women at risk of postnatal depression	One study reported marriage (29/30 women were married)

		postnatal depression							
Button et al. (2017)[19]	24 (24)<br><br>1993-2016	Help seeking for postnatal depression	9 studies carried out in UK. No other countries reported.	NR	NR	Postnatal	9 studies recruited women of colour. 3 studies had mixed samples.	Postnatal depression	NR
Dennis and Chung-Lee (2006)[20]	40 (40)<br><br>NR	Maternal help-seeking barriers and facilitators and treatment preferences for postnatal depression	3 were explicitly stated (all HIC)	NR	NR	Up to 1 year after birth	Three studies recruited women of colour[SA; EA;B;Ar]	Postnatal depression	NR
Evans et al. (2020)[21]	14 (14)<br><br>2009-2015	Acceptability of non-pharmacological interventions for antenatal anxiety	6 countries (all HIC)	235<br><br>16.8 (9.6)	NR	Between 6-40 weeks gestation	NR	8 studies recruited women with a history of mood concerns/ anxiety or depression	2 studies recruited women with "social risk factors"
Forde, Peters & Wittkowski (2020)[46]	13 (15)<br><br>2003-2018	Published empirical studies exploring women's or family members' experiences of PP and/or recovery using	4 countries (all HIC)	103<br>7.92 (2.96)	Range: 23-62	All postnatal, ranging from 4 months to 26 years after onset of postnatal psychosis	One woman was an Orthodox Jewish woman	All women had recovered from, or were currently experiencing postnatal psychosis	NR

For peer review only - http://bmjopen.bmj.com/site/about/guidelines.xhtml

		a qualitative methodology							
Giscombe, Hui & Stickley (2020)[22]	8 (8)<br><br>2008-2017	Refugee or asylum-seeking women, with mental health complications during perinatal period	3 countries (all HIC)	NR	NR	NR	Syrian refugees, Eritrean refugees	6 studies recruited women with depression; 3 with PTSD (1 study recruited both depression & PTSD)	All women were refugees or asylum seekers
Hadfield and Wittkowski (2017)[14]	17 (17)<br><br>2004-2015	Mothers with postnatal depression and their experiences about help seeking for psychosocial support	4 countries (all HIC)	532<br><br>31.3 (25.97)	Range 18-45<br><br>M (n = 2) = 30.2	Postnatal	3 studies recruited women who weren't born in the UK[(B;EA)]	Postnatal depression	NR
Hansotte et al. (2017)[12]	18 (18)<br><br>2004-2015	Screening for postnatal depression and barriers to accessing treatment in low-income women in western countries.	2 countries (all HIC)	85190<br><br>5011 (11613)	M (n = 11) = 25.11	Postnatal	All studies recruited a diverse sample of migrant women or women of colour [(B; L; W; As; NI)]	Self-report depression symptoms or depression diagnosis	All women were low income living in high income western country.
Hewitt et al. (2009)[23]	13 (16)<br><br>1997-2007	Acceptability to women (and HCP) about methods to	5 countries (all HIC)	1715<br><br>131.9 (253.06)	M (n = 8) = 29.63	Postnatal: 1-12 months	4 studies recruited women of	Perinatal depression	2 studies looked at marriage. The majority of women

For peer review only - http://bmjopen.bmj.com/site/about/guidelines.xhtml

		identify postnatal depression				Antenatal: all trimesters	colour (Ar; EA; B; NI; NS)		were married (87-97%)
Holopainen and Hakulinen (2019)[24]	13 (15)<br><br>2005-2015	Mothers (and fathers) experiences of postnatal depression symptoms	7 countries (all HIC)	199<br><br>15.31 (8.21)	Ages ranged from 16-45	1-12 months after birth	5 studies recruited women of colour (B, L, H, SA, EA)	Most studies looked at symptoms of depression, 2 looked at diagnoses	1 study recruited low-income women, one recruited adolescent mothers. Most women were married (n = 3; 59-66%). Most women had more than 9 years of education (n = 2; 87-100%)
Jones et al. (2014)[25]	5 (5)<br><br>1995-2012	Women's experiences of peer support for any degree of perinatal mental illness	3 countries (all HIC)	95<br><br>19 (18.93)	NR	6 weeks - 2 years after birth	NR	Postnatal depression diagnosis or symptoms	NR
Jones (2019)[26]	19 (19)<br><br>2008-2017	Help seeking in women with perinatal depression	All USA	6089<br><br>358.90 (1226.22)	NR	Pregnancy – 6 months after birth	6 studies recruited women of colour (B, L, SA, EA, NS)	All had perinatal depression identified through screening measures, or self-reported.	All women had pregnancy complications. 3 studies recruited women on a low income.
Kassam (2009)[27]	11 separate populations<br><br>1999-2013	Voices of immigrant and refugee women with postnatal depression in terms of social support as a	3 countries stated (HIC & UMIC countries)	191<br><br>23.88 (10.89)	All aged over 17	Screened high on a postnatal depression scale within 2 weeks - 5 years after birth	All studies recruited migrant women or women of colour (NS; As; Ar; SA; H)	Most had postnatal depressive symptoms, identified through screening. One study reported	One study looked at risk profile of women (e.g. low income, experienced violence, experienced war, previous mental health difficulty).

For peer review only - http://bmjopen.bmj.com/site/about/guidelines.xhtml

		coping resource						depression diagnosis	All women in 2 studies were married or in a relationship. One study recruited low-income women.
Lucas et al. (2019)[28]	19 (19)<br><br>1999-2017	Young women's perception of their mental health and wellbeing	3 countries (all HIC)	356<br><br>18.74 (10.02)	Ages ranged from 13-25.<br><br>M (n = 2) = 18.75	11 studies recruited were parents (3 months - 2 years postnatal). 2 studies recruited pregnant women. Remaining studies recruited both pregnant and postnatal women	Majority of studies (15) recruited ethnically diverse (L, B, H, SA, M, As) samples. 4 studies did not report ethnicity	Depressive symptoms, depression diagnosis, other diagnoses (bipolar, panic disorder, mood disorder).	All women were young (maximum age 25)
Megnin-Viggars et al. (2015)[29]	39 (39)<br><br>2001-2013	Women with, or at risk of developing a postnatal mental health problem and their views on factors that improve or	Only reported for 3 studies (all UK)	955<br><br>24.49 (43.77)	1 study recruited teenage mother. No other ages reported.	Antenatal and postnatal	5 studies recruited ethnically diverse samples (B, NS, SA)	Most studies recruited women with depression (n = 14) or women at risk (n = 18) of perinatal mental health problems.	1 study recruited teenage mothers

For peer review only - http://bmjopen.bmj.com/site/about/guidelines.xhtml

		diminish access to perinatal mental health services							
Mollard et al. (2016)[30]	11 (11)<br><br>1995-2014	Women living in rural areas of the USA with PPD. Looking at screening uptake, intervention acceptability, lived experience, help-seeking.	All USA	1610<br><br>146.36 (159.57)	NR	Postnatal	5 studies recruited ethnically diverse samples (NI; B; NS; H)	Postnatal depression symptoms, most used EPDS[1] (n = 6) screen	All women lived in rural location, 3 studies recruited low income women
Morrell et al. (2016)[31]	38 individual samples of women in the qualitative review<br><br>1987-2013	Pregnant and postnatal women, views on preventative or targeted services for PND	8 countries (1 LMIC – India)	1673 (34 studies reported sample size)<br><br>49.21 (98.49)	Ages ranged from 15-54<br><br>M (n = 12) = 28.62	Pregnancy and postnatal	10 recruited ethnically diverse samples (SA; EA, B; H; NI; L; M; NS)	Depression - both symptoms and diagnoses	25 studies reported sociodemographic characteristics. 16 studies reported marital status, in all but 1 study the majority of women were married/cohabiting/in a relationship. 8 studies reported education status: most had completed high school or above. 4 studies recruited low-income women or those living in an

For peer review only - http://bmjopen.bmj.com/site/about/guidelines.xhtml

									impoverished/deprive d area
Newman et al. (2019)[32]	4 (4)<br><br>2008-2016	Women with depression during the postnatal period sharing views on help-seeking	3 countries (all HIC)	118<br><br>29.5 (9)	M (n = 3) = 31.97	Postnatal	NR	Depressive symptoms, measured by EPDS[1]	NR
Nilaweera et al. (2014)[33]	9 (15)	Women who have migrated from South Asian countries to live in high-income countries, barriers and enablers to health care access	4 countries (all HIC)	20,788<br><br>2309.78 (3926.13)	NR	2 weeks to 5 years postnatal	All studies recruited women born in South Asia	Most (n = 5) used EPDS[1] to assess postnatal depression symptoms	NR
Praetorius, Maxwell & Alam (2020)[34]	8 (8)<br><br>1999-2016	Mothers with depression and suicidality	5 countries (3 HIC, 1 UMIC, 1LMIC)	199<br><br>24.88 (12.52)	Ages range from 17-44	Pregnancy and postnatal	All studies recruited diverse samples (B, L, M, SA, EA, Ar, W)	All women had depression and suicidality	NR
Randall and Briscoe (2018)[35]	4 (4)<br><br>2005-2014	Women's decision-making process around antidepressant	2 countries (all HIC)	368<br><br>92 (37.09)	Ages ranged from 25-34	Pregnancy	3 studies reported ethnicity. The majority of women	Depression – 1 study used the CES-D[2] to identify depressive symptoms	3 studies report education, the majority (82.5-100%) were educated to above high school level. 3 studies

For peer review only - http://bmjopen.bmj.com/site/about/guidelines.xhtml

		use during pregnancy			M (n = 2) = 31		were white (77.5-95%)		reported relationship status, the majority (80-98%) were married/living with partner
Slade, Molyneux & Watt (2021)[39]	13 (13 – qualitative papers only)<br><br>2007-2019	Help seeking for birth trauma/ postnatal PTSD	7 countries (1 UMIC; 6 HIC)	394<br><br>30.31 (32.85)	Ages range from 2-45<br><br>M (n=4) = 32	Up to 18 months after birth	8 studies reported ethnicity. One study reported recruiting women of colour[B, H]	All PTSD after birth	One study recruited low-income women. 2 studies reported marital status, over 58% were married. 2 studies reported higher education, at least 50% of women had completed this.
Sambrook-Smith et al. (2019)[36]	24 (35)<br><br>2007 - 2018	Barriers to accessing perinatal mental health care from the perspective of women (families & HCP)	All UK	384<br><br>16 (8.80)	NR	Postnatal	9 recruited women of colour [B; SA; EA]	Most looked at depressive symptoms (n = 12). Studies also recruited women with antenatal anxiety (n = 1), postnatal psychosis (n = 5), PTSD (n = 1) and substance misuse (n =1)	NR
Sorsa, Kylma and Bondas (2021)[40]	14 (14)<br><br>2002-2018	Helpseeking in women with perinatal distress	5 countries (all HIC)	345<br><br>24.65 (11.99)	Ages ranged from 18-55	Antenatal and postnatal	NR	Postnatal depression (n -= 8); prenatal depression (n = 2); Perinatal	NR

For peer review only - http://bmjopen.bmj.com/site/about/guidelines.xhtml

					M (n = 7) = 30.21			mental health needs (n = 2); Postpartum mood disorder (n = 1), Bipolar disorder (n = 1)	
Schmied et al. (2017)[37]	12 individual samples<br><br>1999-2015	Migrant women living in high income countries	4 countries (all HIC)	250<br><br>20.83 (12.52)	M (n = 5) = 29.4	Postnatal	All studies recruited migrant women or women of colour (SA; EA; H; B; Ar; L)	Depressive symptoms or formal diagnosis	1 study recruited low income women
Scope et al. (2017)[38]	22 individual samples<br><br>1987 - 2014	Service user views on uptake, acceptability of preventative interventions for PND	7 countries (all HIC)	982 (reported by author)	13-45 years	Antenatal and postnatal	NR	NR	NR
Staneva et al. (2015)[41]	8 (8)<br><br>2006-2012	Womens experience of antenatal mental health difficulties	5 countries (1 LMIC - Cambodia)	1094<br><br>14 (6.26)	Ages ranged from 16-47	Antenatal	Most studies (n = 6) recruited ethnically diverse samples (B; M)	Self-report distress, depression (n = 5); diagnoses depression/anxiety (n = 2); FOC = 1	50-100% of women were in a relationship
Tobin et al. (2018)[42]	8 (individual samples)	Refugee or immigrant women's experiences of	3 countries (all HIC)	139<br><br>17.38 (7.98)	Age ranges between	Postnatal	All studies recruited migrant women	Postnatal depression	6 studies reported relationship status 50-85% of women were

For peer review only - http://bmjopen.bmj.com/site/about/guidelines.xhtml

	2004-2013	postpartum depression			17-54 years		and women of colour[L; H; SA;EA;B]		married/in a relationship
Viveiros and Darling (2019)[43]	7 (26)<br><br>2009-2018	To explore women (and midwives) perceptions on factors that impede access to perinatal mental health care in high resource settings	2 countries (both HIC)	301<br><br>43 (66.30)	1 study reported age range from 23-40	Antenatal and postnatal	2 studies recruited 'BAME' women, one recruited all Black women	PTSD symptoms (n = 1); mental health problems (n = 2); mental illness diagnosis (n = 1)	NR
Watson et al. (2019)[44]	15 (15)<br><br>1994-2015	Ethnic minority women's experience of perinatal mental ill health, help-seeking and perinatal mental health services in Europe	All UK	4970<br><br>331.33 (1173.09)	NR	Antenatal and postnatal	All studies recruited women of colour [SA; NS; N; EA; M]	Distress, depression, mood and mental health, well-being	NR
Wittkowski et al. (2014)[45]	12 (12)<br><br>1983 - 2009	Culturally determined risk factors of PND in Sub-Saharan Africa	3 countries – all Sub-Saharan Africa	3642<br><br>404.67 (343.16)	NR	Postnatal	NR	All used self-report measures of depression	NR

For peer review only - http://bmjopen.bmj.com/site/about/guidelines.xhtml

Note. Where studies recruited populations that were not perinatal women, the information from these studies are not included in this table. HCP = Healthcare professional; LMIC = Lower-Middle Income Country; HIC = Higher Income Country; PTSD = Post-traumatic stress disorder; FOC = Fear of Childbirth. 1 = Edinburgh Postnatal Depression Scale (Cox et al., 1987); 2 = Center for Epidemiological Studies-Depression (Radloff, 1977).

For ethnicities: As = Asian (where the area of Asia was not specified in the study); EA = East Asian (e.g. Vietnamese; Chinese; Thai); SA = South Asian (e.g. Indian; Bangladeshi; Pakistani; Sri-Lankan); Ar = Arab countries (e.g. Jordanian, Egyptian); Ar = Arabic; B = Black; H = Hispanic; L = Latina; M = mixed or multiple ethnic groups; NI = Native/Indigenous; NS = Not specified; W = White.

For peer review only

For peer review only - http://bmjopen.bmj.com/site/about/guidelines.xhtml

Appendix 11: Table 7. Quality appraisal

Author, year	Q1. PICO	Q2. Protocol*	Q3. Study design	Q4. Literature search*	Q5. Study selection	Q6. Data extraction	Q7. Excluded studies*	Q8. Included studies	Q9. ROB	Q.10 Funding	Q13. ROB interpretation	Q14. Heterogeneity	Q16. conflict of interest*	Rating
**Bina, 2020[11]**	Yes	Yes	No	Partial yes	Yes	No	Partial yes	Yes	No	No	No	Yes	No	LOW
**Brealey et al., 2010[18]**	Yes	Yes	Yes	Yes	Yes	Yes	No	Yes	Partial yes	No	Yes	Yes	No	CRITICALLY LOW
**Button et al., 2017[19]**	Yes	No	Yes	Yes	No	No	Partial yes	Partial yes	Yes	No	Yes	Yes	Yes	LOW
**Dennis & Chung-Lee, 2006[20]**	Yes	No	Yes	Yes	No	Yes	No	Partial yes	No	No	No	No	No	CRITICALLY LOW
**Evans et al., 2020[21]**	Yes	Yes	Yes	Yes	Yes	Yes	Partial yes	Yes	Yes	No	Yes	Yes	Yes	HIGH
**Forde et al., 2020[46]**	Yes	Yes	Yes	Partial yes	Yes	No	Partial yes	Ys	Yes	No	Yes	Yes	Yes	MODERATE
**Giscombe et al., 2020[22]**	Yes	No	Yes	Partial yes	No	No	Partial yes	Yes	Yes	No	No	Yes	No	CRITICALLY LOW
**Hadfield & Wittkowski, 2017[14]**	Yes	Yes	Yes	Yes	No	No	Partial yes	Yes	Yes	No	Yes	Yes	Yes	MODERATE
**Hansotte et al., 2017[12]**	Yes	No	Yes	Yes	Yes	Yes	Partial yes	Yes	No	No	No	Yes	Yes	LOW
**Hewitt et al., 2009[23]**	Yes	Yes	Yes	Yes	Yes	Yes	Partial yes	Yes	Yes	No	Yes	Yes	Yes	HIGH
**Holopainen & Hakulinen, 2019[24]**	Yes	Yes	Yes	Yes	Yes	Yes	Yes	Yes	Yes	No	Yes	Yes	Yes	HIGH

For peer review only - http://bmjopen.bmj.com/site/about/guidelines.xhtml

**Jones et al., 2014**[25]	Yes	No	Yes	Yes	No	Yes	No	Partial yes	Yes	No	Yes	Yes	Yes	CRITICALLY LOW
**Jones, 2019**[26]	Yes	No	Yes	Partial yes	Yes	No	Partial yes	Yes	No	No	Yes	Yes	Yes	LOW
**Kassam, 2019**[27]	Yes	Yes	Yes	Partial yes	No	No	Partial yes	Yes	Yes	No	Yes	Yes	Yes	MODERATE
**Lucas et al., 2019**[28]	Yes	Yes	Yes	Yes	Yes	Yes	Partial yes	Yes	Yes	No	Yes	Yes	Yes	HIGH
**Megnin-Viggars et al., 2015**[29]	Yes	Yes	Yes	Partial yes	No	Yes	Partial yes	Yes	Yes	No	Yes	Yes	No	LOW
**Mollard et al., 2016**[30]	Yes	No	Yes	Partial yes	No	No	Partial yes	Partial yes	No	No	No	Yes	Yes	LOW
**Morell et al. 2016**[31]	Yes	Yes	Yes	Yes	Yes	Yes	Yes	Yes	Ye	No	Yes	Yes	Yes	HIGH
**Newman et al., 2019**[32]	Yes	No	Yes	Yes	No	No	Partial yes	Yes	Yes	No	No	No	Yes	LOW
**Nilaweera et al., 2014**[33]	Yes	No	No	Partial yes	No	No	Partial yes	Yes	Yes	No	Yes	Yes	Yes	LOW
**Praetorius et al., 2020**[34]	No	No	Yes	Yes	No	Yes	Partial yes	Partial yes	No	No	No	No	Yes	LOW
**Randall & Briscoe, 2018**[35]	Yes	No	No	Partial yes	Yes	No	Partial yes	Partial yes	Yes	No	No	Yes	Yes	LOW
**Sambrook-Smith et al., 2019**[36]	Yes	Yes	Yes	Partial yes	Yes	No	Partial yes	Yes	Yes	No	Yes	Yes	Yes	MODERATE
**Schmied et al., 2017**[37]	Yes	No	Yes	Partial yes	Yes	No	Partial yes	Yes	Yes	No	Yes	Yes	Yes	LOW
**Scope et al., 2017**[38]	Yes	Yes	Yes	Partial yes	Yes	Yes	Partial yes	Yes	Yes	No	Yes	Yes	No	LOW

**Staneva et al., 2015[41]**	Yes	Yes	Yes	Partial yes	Yes	No	Partial yes	Yes	Yes	No	Yes	Yes	Yes	MODERATE
**Slade et al., 2020[39]**	Yes	Yes	Yes	Yes	No	No	Partial yes	Yes	Yes	No	Yes	Yes	Yes	MODERATE
**Sorsa et al., 2021[40]**	Yes	No	Yes	Partial yes	Partial yes	Yes	Partial yes	Yes	Yes	No	Yes	Yes	Yes	LOW
**Tobin et al., 2018[42]**	Yes	Yes	Yes	Yes	No	No	No	Yes	Yes	No	No	Yes	Yes	MODERATE
**Viveiros & Darling, 2018[43]**	Yes	No	No	Yes	Yes	No	Partial yes	Yes	No	No	No	Yes	Yes	LOW
**Watson et al., 2019[44]**	Yes	Yes	Yes	Partial yes	Yes	No	Partial yes	Yes	Yes	No	Yes	Yes	Yes	MODERATE
**Wittkowski et al., 2014[45]**	Yes	No	Yes	Partial yes	No	No	Partial yes	Yes	Yes	No	Yes	Yes	No	CRITICALLY LOW

* = Critical domain

1. Did the research questions and inclusion criteria for the review include the components of PICO? *2.* Did the report of the review contain an explicit statement that the review methods were established prior to the conduct of the review and did the report justify any significant deviations from the protocol?*3.* Did the review authors explain their selection of the study designs for inclusion in the review?4. Did the review authors use a comprehensive literature search strategy? 5. Did the review authors perform study selection in duplicate? 6. Did the review authors perform data extraction in duplicate? 7. Did the review authors provide a list of excluded studies and justify the exclusions? 8. Did the review authors describe the included studies in adequate detail? 9. Did the review authors use a satisfactory technique for assessing the risk of bias (RoB) in individual studies that were included in the review? 10. Did the review authors report on the sources of funding for the studies included in the review? 11. If meta-analysis was performed, did the review authors use appropriate methods for statistical combination of results? *(not applicable)* 12. If meta-analysis was performed, did the review authors assess the potential impact of RoB in individual studies on the results of the meta-analysis or other evidence synthesis? *(not applicable)* 13. Did the review authors account for RoB in primary studies when interpreting/discussing the results of the review? 14. Did the review authors provide a satisfactory explanation for, and discussion of, any heterogeneity observed in the results of the review? 15. If they performed quantitative synthesis did the review authors carry out an adequate investigation of publication bias (small study bias) and discuss its likely impact on the results of the review? *(not applicable)* 16. Did the review authors report any potential sources of conflict of interest, including any funding they received for conducting the review?

For peer review only - http://bmjopen.bmj.com/site/about/guidelines.xhtml

Appendix 12: Table 8. Themes

Theme	Studies reflecting this theme
**1. Women**	
**1.1 Beliefs about health services**	
**1.1.1 Medication only**	Bina, 2020; Button et al., 2017; Jones, 2019; Megnin-Viggars et al., 2015; Nilaweera et al., 2014; Sorsa et al., 2021; Tobin et al., 2018
**1.1.2 Stretched**	Hadfield & Wittkowski, 2017
**1.2 Beliefs about healthcare professionals**	
**1.2.1 What is their role?**	Brealey et al., 2010; Button et al., 2017; Dennis & Chung-Lee, 2006; Hadfield & Wittkowski, 2017; Hewitt et al., 2009; Megnin-Viggars et al., 2015; Mollard et al., 2016; Morrell et al., 2016; Nilaweera et al., 2014; Schmied et al., 2017; Scope et al., 2017; Smith et al., 2019
**1.2.2 They won't be interested**	Bina, 2020; Hadfield & Wittkowski, 2017
**1.3 Beliefs about mental illness**	
**1.3.1 Not knowing what it is**	Bina, 2020; Button et al., 2017; Dennis & Chung-Lee, 2006; Hadfield & Wittkowski, 2017; Hansotte et al., 2017; Jones, 2019; Lucas et al., 2019; Megnin-Viggars et al., 2015; Morrell et al., 2016; Newman et al., 2019; Schmied et al., 2017; Scope et al., 2017; Smith et al., 2019; Staneva et al., 2015; Tobin et al., 2018; Watson et al., 2019
**1.3.1.1. Not having the language to describe perinatal mental illness**	Brealey et al., 2010; Staneva et al., 2015; Tobin et al., 2018; Watson et al., 2019
**1.3.2 Causes**	
**1.3.2.1 Cultural/spiritual**	Schmied et al., 2017; Wittkowski et al., 2014
**1.3.2.2 External factors**	Bina, 2020; Button et al., 2017; Dennis & Chung-Lee, 2006; Lucas et al., 2019; Schmied et al., 2017; Staneva et al., 2015; Tobin et al., 2018; Watson et al., 2019
**1.3.2.3 Physical factors**	Bina, 2020; Button et al., 2017; Dennis & Chung-Lee, 2006; Forde et al., 2020; Jones et al., 2014; Newman et al., 2019; Schmied et al., 2017; Smith et al., 2019; Staneva et al., 2015; Watson et al., 2019
**1.3.2.4 A normal response to motherhood?**	Dennis & Chung-Lee, 2006; Giscombe et al., 2020; Jones et al., 2014; Schmied et al., 2017; Slade et al., 2020; Smith et al., 2019; Sorsa et al., 2021
**1.3.3 How to deal with symptoms**	
**1.3.3.1 Ignore them**	Bina, 2020; Hadfield & Wittkowski, 2017; Jones, 2019; Newman et al., 2019; Schmied et al., 2017; Slade et al., 2020
**1.3.3.2 Seek spiritual guidance**	Hansotte et al., 2017; Kassam, 2019; Schmied et al., 2017; Watson et al., 2019
**1.4 Deciding to seek help**	

For peer review only - http://bmjopen.bmj.com/site/about/guidelines.xhtml

**1.4.1 Recognising something is wrong**	Bina, 2020; Button et al., 2017; Forde et al., 2020; Hadfield & Wittkowski, 2017; Hansotte et al., 2017; Slade et al., 2020; Sorsa et al., 2021; Staneva et al., 2015; Viveiros & Darling, 2018
**1.4.2 Where do I go to seek help?**	Bina, 2020; Dennis & Chung-Lee, 2006; Hansotte et al., 2017; Jones, 2019; Megnin-Viggars et al., 2015; Schmied et al., 2017; Smith et al., 2019; Sorsa et al., 2021; Tobin et al., 2018
**1.5 Fear of judgement**	
**1.5.1 Fear of being seen as a bad mum**	Bina, 2020; Brealey et al., 2010; Button et al., 2017; Forde et al., 2020; Jones et al., 2014; Lucas et al., 2019; Schmied et al., 2017; Slade et al., 2020; Sorsa et al., 2021; Viveiros & Darling, 2018; Watson et al., 2019
**1.5.2 Social services/removal of child**	Bina, 2020; Brealey et al., 2010; Button et al., 2017; Dennis & Chung-Lee, 2006; Evans et al., 2020; Forde et al., 2020; Hadfield & Wittkowski, 2017; Hewitt et al., 2009; Jones, 2019; Megnin-Viggars et al., 2015; Newman et al., 2019; Tobin et al., 2018; Watson et al., 2019
**1.5.3 Symptom minimisation**	Bina, 2020; Dennis & Chung-Lee, 2006; Forde et al., 2020; Hewitt et al., 2009; Holopainen & Hakulinen, 2019; Jones et al., 2014; Kassam, 2019; Lucas et al., 2019; Megnin-Viggars et al., 2015; Schmied et al., 2017; Staneva et al., 2015; Watson et al., 2019
**1.6 Logistics**	
**1.6.1 Childcare**	Bina, 2020; Button et al., 2017; Dennis & Chung-Lee, 2006; Hansotte et al., 2017; Morrell et al., 2016; Newman et al., 2019; Scope et al., 2017; Smith et al., 2019; Tobin et al., 2018; Watson et al., 2019
**1.6.2 Timing**	Bina, 2020; Dennis & Chung-Lee, 2006; Newman et al., 2019; Scope et al., 2017; Watson et al., 2019
**1.6.3 Location/travel**	Bina, 2020; Hansotte et al., 2017; Jones, 2019; Mollard et al., 2016; Morrell et al., 2016; Newman et al., 2019; Schmied et al., 2017; Sorsa et al., 2021; Tobin et al., 2018; Watson et al., 2019
**1.7 Social and family life**	
**1.7.1 Social isolation/support**	Bina, 2020; Giscombe et al., 2020; Holopainen & Hakulinen, 2019; Jones, 2019; Jones et al., 2014; Kassam, 2019; Lucas et al., 2019; Tobin et al., 2018; Viveiros & Darling, 2018
**1.7.1. 1 Exacerbated by mental illness**	Holopainen & Hakulinen, 2019; Jones et al., 2014; Watson et al., 2019
**1.7.2 Family and friends**	Bina, 2020; Button et al., 2017; Dennis & Chung-Lee, 2006; Forde et al., 2020; Hadfield & Wittkowski, 2017; Hansotte et al., 2017; Holopainen & Hakulinen, 2019; Jones, 2019; Lucas et al., 2019; Nilaweera et al., 2014; Schmied et al., 2017; Smith et al., 2019; Sorsa et al., 2021; Viveiros & Darling, 2018; Watson et al., 2019
**1.8 Sociodemographic factors**	
**1.8.1 Ethnicity**	Bina, 2020; Dennis & Chung-Lee, 2006; Hansotte et al., 2017; Watson et al., 2019
**1.8.2 Age**	Bina, 2020; Hansotte et al., 2017
**1.8.3 Previous experiences**	Button et al., 2017; Evans et al., 2020; Hadfield & Wittkowski, 2017; Hansotte et al., 2017; Jones, 2019; Sorsa et al., 2021; Watson et al., 2019
**1.8.4 Previous Diagnoses/symptoms**	Bina, 2020; Viveiros & Darling, 2018

For peer review only - http://bmjopen.bmj.com/site/about/guidelines.xhtml

**2. HCP**	
**2.1 HCP being dismissive or normalising symptoms**	Button et al., 2017; Dennis & Chung-Lee, 2006; Forde et al., 2020; Hadfield & Wittkowski, 2017; Hansotte et al., 2017; Megnin-Viggars et al., 2015; Newman et al., 2019; Sorsa et al., 2021; Watson et al., 2019
**2.2 HCP not recognising help seeking**	Bina, 2020; Button et al., 2017; Megnin-Viggars et al., 2015; Tobin et al., 2018; Watson et al., 2019
**2.3 HCP appearing too busy**	Bina, 2020; Button et al., 2017; Dennis & Chung-Lee, 2006; Hewitt et al., 2009; Megnin-Viggars et al., 2015; Slade et al., 2020; Viveiros & Darling, 2018; Watson et al., 2019
**2.3 Women's perceptions of HCPs knowledge**	
**2.3.1 Perception of HCP knowledge about PNMI**	Dennis & Chung-Lee, 2006; Forde et al., 2020; Hansotte et al., 2017; Jones, 2019; Megnin-Viggars et al., 2015; Morrell et al., 2016
**2.3.2 Perception of HCP knowledge about services/referral pathways**	Dennis & Chung-Lee, 2006; Smith et al., 2019; Viveiros & Darling, 2018
**2.4 The way the HCP delivers the care**	Button et al., 2017; Forde et al., 2020; Kassam, 2019; Megnin-Viggars et al., 2015; Nilaweera et al., 2014; Schmied et al., 2017; Slade et al., 2020; Smith et al., 2019; Sorsa et al., 2021
**2.5 HCP characteristics**	Brealey et al., 2010; Button et al., 2017; Dennis & Chung-Lee, 2006; Forde et al., 2020; Hadfield & Wittkowski, 2017; Hewitt et al., 2009; Megnin-Viggars et al., 2015; Morrell et al., 2016; Newman et al., 2019; Schmied et al., 2017; Slade et al., 2020; Staneva et al., 2015; Viveiros & Darling, 2018
**3. Interpersonal**	
**3.1 Relationship and rapport**	Bina, 2020; Brealey et al., 2010; Dennis & Chung-Lee, 2006; Hadfield & Wittkowski, 2017; Hewitt et al., 2009; Megnin-Viggars et al., 2015; Morrell et al., 2016; Scope et al., 2017; Slade et al., 2020; Tobin et al., 2018
**3.2 Language barriers**	Button et al., 2017; Dennis & Chung-Lee, 2006; Hansotte et al., 2017; Megnin-Viggars et al., 2015; Schmied et al., 2017; Smith et al., 2019
**3.3 Shared decision making**	Bina, 2020; Hadfield & Wittkowski, 2017; Megnin-Viggars et al., 2015; Morrell et al., 2016; Randall & Briscoe, 2018; Scope et al., 2017
**3.4 Communication**	Brealey et al., 2010; Forde et al., 2020; Hadfield & Wittkowski, 2017; Hewitt et al., 2009
**3.5 Information provision**	Bina, 2020; Button et al., 2017; Megnin-Viggars et al., 2015; Randall & Briscoe, 2018; Slade et al., 2020; Smith et al., 2019; Sorsa et al., 2021; Tobin et al., 2018
**4. Organisational**	
**4.1 Lack of services/Overstretched**	Bina, 2020; Button et al., 2017; Forde et al., 2020; Jones, 2019; Megnin-Viggars et al., 2015; Smith et al., 2019; Tobin et al., 2018; Viveiros & Darling, 2018
**4.2 Characteristics of service**	Bina, 2020; Button et al., 2017; Dennis & Chung-Lee, 2006; Forde et al., 2020; Jones, 2019; Newman et al., 2019; Scope et al., 2017; Viveiros & Darling, 2018; Watson et al., 2019

For peer review only - http://bmjopen.bmj.com/site/about/guidelines.xhtml

**4.3 Collaboration within and across services**	Bina, 2020; Megnin-Viggars et al., 2015; Newman et al., 2019; Smith et al., 2019; Watson et al., 2019
**4.4 Continuity of carer**	Brealey et al., 2010; Button et al., 2017; Hadfield & Wittkowski, 2017; Megnin-Viggars et al., 2015; Slade et al., 2020; Smith et al., 2019; Tobin et al., 2018; Viveiros & Darling, 2018; Watson et al., 2019
**4.5 Ideal care**	
**4.5.1 Screening**	
**4.5.1.1 Screening acceptability**	Brealey et al., 2010; Button et al., 2017; Evans et al., 2020; Hewitt et al., 2009; Megnin-Viggars et al., 2015; Mollard et al., 2016; Smith et al., 2019
**4.5.1.2 Wording/contents**	Brealey et al., 2010; Hewitt et al., 2009
**4.5.1.3 Delivery**	Brealey et al., 2010; Button et al., 2017; Evans et al., 2020; Hewitt et al., 2009; Smith et al., 2019
**4.5.2 Optimal treatment**	
**4.5.2.1 Opportunity to talk**	Dennis & Chung-Lee, 2006; Evans et al., 2020; Hadfield & Wittkowski, 2017; Jones, 2019; Kassam, 2019; Morrell et al., 2016; Praetorius et al., 2020; Staneva et al., 2015
**4.5.2.2 Location**	Hadfield & Wittkowski, 2017; Hansotte et al., 2017; Jones, 2019; Jones et al., 2014; Mollard et al., 2016; Newman et al., 2019; Praetorius et al., 2020; Sorsa et al., 2021
**4.5.2.3 Appropriate**	Evans et al., 2020; Megnin-Viggars et al., 2015; Scope et al., 2017; Sorsa et al., 2021
**4.5.2.4 Individualised**	Evans et al., 2020; Hadfield & Wittkowski, 2017; Megnin-Viggars et al., 2015; Morrell et al., 2016; Schmied et al., 2017; Scope et al., 2017; Slade et al., 2020; Sorsa et al., 2021; Viveiros & Darling, 2018; Watson et al., 2019
**4.5.2.5 Length**	Hadfield & Wittkowski, 2017; Morrell et al., 2016; Schmied et al., 2017; Watson et al., 2019
**4.5.2.6 Group/Peer support**	Dennis & Chung-Lee, 2006; Evans et al., 2020; Forde et al., 2020; Hadfield & Wittkowski, 2017; Jones et al., 2014; Megnin-Viggars et al., 2015; Morrell et al., 2016; Schmied et al., 2017; Scope et al., 2017; Slade et al., 2020; Tobin et al., 2018; Watson et al., 2019
**4.5.2.6.1 Validation provided by peer support**	Jones et al., 2014; Morrell et al., 2016; Schmied et al., 2017; Slade et al., 2020
**4.5.2.7 Culturally appropriate**	Button et al., 2017; Dennis & Chung-Lee, 2006; Giscombe et al., 2020; Hadfield & Wittkowski, 2017; Jones, 2019; Schmied et al., 2017; Smith et al., 2019; Tobin et al., 2018; Watson et al., 2019
**4.5.2.8 Information provision**	Forde et al., 2020; Hadfield & Wittkowski, 2017; A. Jones, 2019; Megnin-Viggars et al., 2015; Morrell et al., 2016; Schmied et al., 2017; Scope et al., 2017; Slade et al., 2020; Sorsa et al., 2021
**4.5.2.9 Medication**	Dennis & Chung-Lee, 2006; Forde et al., 2020; Jones, 2019; Sorsa et al., 2021; Tobin et al., 2018
**5. Political**	
**5.1 Immigration status**	Bina, 2020; Giscombe et al., 2020; Hansotte et al., 2017; Kassam, 2019; Lucas et al., 2019; Schmied et al., 2017

For peer review only - http://bmjopen.bmj.com/site/about/guidelines.xhtml

**5.2. Economic status**	Schmied et al., 2017; Tobin et al., 2018; Watson et al., 2019
**5.2.1 Healthcare costs**	Bina, 2020; Hansotte et al., 2017; Schmied et al., 2017; Tobin et al., 2018; Viveiros & Darling, 2018
**6. Societal**	
**6.1. Culture**	Bina, 2020; Brealey et al., 2010; Button et al., 2017; Dennis & Chung-Lee, 2006; Giscombe et al., 2020; Hansotte et al., 2017; Hewitt et al., 2009; Jones, 2019; Kassam, 2019; Megnin-Viggars et al., 2015; Nilaweera et al., 2014; Praetorius et al., 2020; Schmied et al., 2017; Smith et al., 2019; Sorsa et al., 2021; Staneva et al., 2015; Tobin et al., 2018; Viveiros & Darling, 2018; Watson et al., 2019; Wittkowski et al., 2014
**6.2. Maternal norms**	Bina, 2020; Brealey et al., 2010; Button et al., 2017; Dennis & Chung-Lee, 2006; Forde et al., 2020; Hadfield & Wittkowski, 2017; Hansotte et al., 2017; Hewitt et al., 2009; Holopainen & Hakulinen, 2019; Jones et al., 2014; Lucas et al., 2019; Mollard et al., 2016; Morrell et al., 2016; Newman et al., 2019; Schmied et al., 2017; Slade et al., 2020; Smith et al., 2019; Sorsa et al., 2021; Staneva et al., 2015; Viveiros & Darling, 2018
**6.3. Stigma**	Bina, 2020; Button et al., 2017; Dennis & Chung-Lee, 2006; Forde et al., 2020; Giscombe et al., 2020; Hadfield & Wittkowski, 2017; Hansotte et al., 2017; Hewitt et al., 2009; Holopainen & Hakulinen, 2019; Jones, 2019; Kassam, 2019; Lucas et al., 2019; Megnin-Viggars et al., 2015; Morrell et al., 2016; Nilaweera et al., 2014; Scope et al., 2017; Smith et al., 2019; Sorsa et al., 2021; Staneva et al., 2015; Tobin et al., 2018; Viveiros & Darling, 2018; Watson et al., 2019

*Note: highlighted yellow are themes only represented by lower-quality studies

For peer review only

For peer review only - http://bmjopen.bmj.com/site/about/guidelines.xhtml

Appendix 13: References

1. Curry SJ, Krist AH, Owens DK, et al. Interventions to Prevent Perinatal Depression: US Preventive Services Task Force Recommendation Statement. *JAMA*. 2019;321(6):580-587. doi:10.1001/JAMA.2019.0007

2. What is the CPMHC? – CANADIAN PERINATAL MENTAL HEALTH COLLABORATIVE / COLLECTIF CANADIEN POUR LA SANTÉ MENTALE PÉRINATALE. Accessed April 12, 2022. https://cpmhc.ca/what-is-the-cpmhc/

3. PMHP - Perinatal Mental Health Project. Accessed April 12, 2022. https://pmhp.za.org/

4. Perinatal Anxiety & Depression Australia (PANDA). *Submission to the Productivity Commission Mental Health Inquiry: The Social and Economic Benefits of Improving Perinatal Mental Health*.; 2019.

5. National Institute for Health and Care Excellence (NICE). *Antenatal and Postnatal Mental Health Clinical Management and Service*.; 2014.

6. Moher D, Liberati A, Tetzlaff JM, Altman DG. Preferred reporting items for systematic reviews and meta-analyses: the PRISMA statement. *PLoS Med*. 2009;6(7):e1000097. doi:10.1016/j.jclinepi.2009.06.005

7. The Cochrane Collaboration. Cochrane data collection form for intervention reviews for RCTs only. *The cochrane Collaboration*. 2014;(April):2014. http://www.cochrane.org/

8. Shea BJ, Reeves BC, Wells GA, et al. AMSTAR 2: A critical appraisal tool for systematic reviews that include randomised or non-randomised studies of healthcare interventions, or both. *BMJ*. 2017;21:358. doi:10.1136/bmj.j4008

9. Harden A. *"Qualitative" Research, Systematic Reviews, and Evidence-Informed Policy and Practice*. University of London; 2007.

10. Carroll C, Booth A, Lloyd-Jones M. Should we exclude inadequately reported studies from qualitative systematic reviews? An evaluation of sensitivity analyses in two case study reviews. *Qualitative Health Research*. 2012;22(10):1425-1434.

11. Bina R. Predictors of postpartum depression service use: A theory-informed, integrative systematic review. *Women and Birth*. 2020;33(1):e24-e32. doi:10.1016/j.wombi.2019.01.006

12. Hansotte E, Payne SI, Babich SM. Positive postpartum depression screening practices and subsequent mental health treatment for low-income women in Western countries: A systematic literature review. *Public Health Reviews*. 2017;38(1):1-17. doi:10.1186/s40985-017-0050-y

13. Geertz C. *The Interpretation of Cultures: Selected Essays*. Basic Books; 1973.

14. Hadfield H, Wittkowski A. Women's Experiences of Seeking and Receiving Psychological and Psychosocial Interventions for Postpartum Depression: A Systematic Review and Thematic Synthesis of the Qualitative Literature. *Journal of Midwifery and Women's Health*. 2017;62(6):723-736. doi:10.1111/jmwh.12669

15. Popay J, Rogers A, Williams G. Rationale and standards for the systematic review of qualitative literature in health services research. *Qualitative Health Research*. 1998;8(3):341-351. doi:10.1177/104973239800800305

For peer review only

For peer review only - http://bmjopen.bmj.com/site/about/guidelines.xhtml

16. Thomas J, Harden A. Methods for the thematic synthesis of qualitative research in systematic reviews. *BMC Medical Research Methodology*. 2008;8(1):1-10. doi:10.1186/1471-2288-8-45

17. Ferlie EB, Shortell SM. Improving the Quality of Health Care in the United Kingdom and the United States: A Framework for Change. *Milbank Quarterly*. 2001;79(2):281-315. doi:10.1111/1468-0009.00206

18. Brealey SD, Hewitt C, Green JM, Morrell J, Gilbody S. Screening for postnatal depression - is it acceptable to women and healthcare professionals? A systematic review and meta-synthesis. *Journal of Reproductive and Infant Psychology*. 2010;28(4):328-344. doi:10.1080/02646838.2010.513045

19. Button S, Thornton A, Lee S, Ayers S, Shakespeare J. Seeking help for perinatal psychological distress: A meta-synthesis of women's experiences. *British Journal of General Practice*. 2017;67(663):e692-e699. doi:10.3399/bjgp17X692549

20. Dennis CL, Chung-Lee L. Postpartum depression help-seeking barriers and maternal treatment preferences: A qualitative systematic review. *Birth*. 2006;33(4):323-331. doi:10.1111/j.1523-536X.2006.00130.x

21. Evans K, Spiby H, Morrell J. Non-pharmacological interventions to reduce the symptoms of mild to moderate anxiety in pregnant women. A systematic review and narrative synthesis of women's views on the acceptability of and satisfaction with interventions. *Archives of Women's Mental Health*. 2020;23:11-28. doi:10.1007/s00737-018-0936-9

22. Giscombe T, Hui A, Stickley T. Perinatal mental health amongst refugee and asylum-seeking women in the UK. *Mental Health Review Journal*. 2020;25(3).

23. Hewitt CE, Gilbody S, Brealey SD, et al. Methods to identify postnatal depression in primary care: An integrated evidence synthesis and value of information analysis. *Health Technology Assessment*. 2009;13(36):1-145. doi:10.3310/hta13360

24. Holopainen A, Hakulinen T. New parents' experiences of postpartum depression: A systematic review of qualitative evidence. *JBI Database of Systematic Reviews and Implementation Reports*. 2019;17(9):1731-1769. doi:10.11124/JBISRIR-2017-003909

25. Jones CCG, Jomeen J, Hayter M. The impact of peer support in the context of perinatal mental illness: A meta-ethnography. *Midwifery*. 2014;30(5):491-498. doi:10.1016/j.midw.2013.08.003

26. Jones A. Help seeking in the perinatal period: a review of barriers and facilitators. *Soc Work Public Health*. 2019;34(7):596-605.

27. Kassam S. Understanding Experiences of Social Support as Coping Resources among Immigrant and Refugee Women with Postpartum Depression: An Integrative Literature Review. *Issues in Mental Health Nursing*. 2019;40(12):999-1011. doi:10.1080/01612840.2019.1585493

28. Lucas G, Olander EK, Ayers S, Salmon D. No straight lines - Young women's perceptions of their mental health and wellbeing during and after pregnancy: A systematic review and meta-ethnography. *BMC Women's Health*. 2019;19(1):1-17. doi:10.1186/s12905-019-0848-5

29. Megnin-Viggars O, Symington I, Howard LM, Pilling S. Experience of care for mental health problems in the antenatal or postnatal period for women in the UK: a systematic review and

For peer review only

For peer review only - http://bmjopen.bmj.com/site/about/guidelines.xhtml

meta-synthesis of qualitative research. *Archives of Women's Mental Health*. 2015;18(6):245-759. doi:10.1007/s00737-015-0548-6

30. Mollard E, Hudson DB, Ford A, Pullen C. An Integrative Review of Postpartum Depression in Rural U.S. Communities. *Archives of Psychiatric Nursing*. 2016;30(3):418-424. doi:10.1016/j.apnu.2015.12.003

31. Morrell J, Sutcliffe P, Booth A, et al. A systematic review, evidence synthesis and meta-analysis of quantitative and qualitative studies evaluating the clinical effectiveness, the cost-effectiveness, safety and acceptability of interventions to prevent postnatal depression. *Health Technology Assessment*. 2016;20(37). doi:10.3310/hta20370

32. Newman TC, Hirst J, Darwin Z. What enables or prevents women with depressive symptoms seeking help in the postnatal period? *British Journal of Midwifery*. 2019;27(4):219-227. doi:10.12968/bjom.2019.27.4.219

33. Nilaweera I, Doran F, Fisher J. Prevalence, nature and determinants of postpartum mental health problems among women who have migrated from South Asian to high-income countries: A systematic review of the evidence. *Journal of Affective Disorders*. 2014;166:213-226. doi:10.1016/j.jad.2014.05.021

34. Praetorius R, Maxwell D, Alam K. Wearing a happy mask: mother's expressions of suicidality with postpartum depression. *Social Work in Mental Health*. 2020;18(4):429-459.

35. Randall L, Briscoe L. Are women empowered to make decisions about the use of antidepressants in pregnancy? *British Journal of Midwifery*. 2018;26(5):329-335. doi:10.12968/bjom.2018.26.5.329

36. Sambrook Smith M, Lawrence V, Sadler E, Easter A. Barriers to accessing mental health services for women with perinatal mental illness: Systematic review and meta-synthesis of qualitative studies in the UK. *BMJ Open*. 2019;9(1):e024803. doi:10.1136/bmjopen-2018-024803

37. Schmied V, Black E, Naidoo N, Dahlen HG, Liamputtong P. Migrant women's experiences, meanings and ways of dealing with postnatal depression: A meta-ethnographic study. *PLoS ONE*. 2017;12(3). doi:10.1371/journal.pone.0172385

38. Scope A, Booth A, Morrell J, Sutcliffe P, Cantrell A. Perceptions and experiences of interventions to prevent postnatal depression. A systematic review and qualitative evidence synthesis. *Journal of Affective Disorders*. 2017;210:100-110. doi:10.1016/j.jad.2016.12.017

39. Slade P, Molyneux R, Watt A. A systematic review of clinical effectiveness of psychological interventions to reduce post traumatic stress symptoms following childbirth and a meta-synthesis of facilitators and barriers to uptake of psychological care. *J Affect Disord*. 2020;281:678-694.

40. Sorsa MA, Kylmä J, Bondas TE. Contemplating Help-Seeking in Perinatal Psychological Distress—A Meta-Ethnography. *Int J Environ Res Public Health*. 2021;18(10):5226.

41. Staneva AA, Bogossian F, Wittkowski A. The experience of psychological distress, depression, and anxiety during pregnancy: A meta-synthesis of qualitative research. *Midwifery*. 2015;31(6):563-573. doi:10.1016/j.midw.2015.03.015

For peer review only - http://bmjopen.bmj.com/site/about/guidelines.xhtml

42. Tobin CL, di Napoli P, Beck CT. Refugee and Immigrant Women's Experience of Postpartum Depression: A Meta-Synthesis. *J Transcult Nurs*. 2018;29(1):84-100. doi:10.1177/1043659616686167

43. Viveiros CJ, Darling EK. Perceptions of barriers to accessing perinatal mental health care in midwifery: A scoping review. *Midwifery*. 2019;70:106-118. doi:10.1016/j.midw.2018.11.011

44. Watson H, Harrop D, Walton E, Young A, Soltani H. A systematic review of ethnic minority women's experiences of perinatal mental health conditions and services in Europe. *PLoS ONE*. 2019;14(1):e0210587. doi:10.1371/journal.pone.0210587

45. Wittkowski A, Gardner PL, Bunton P, Edge D. Culturally determined risk factors for postnatal depression in Sub-Saharan Africa: A mixed method systematic review. *Journal of Affective Disorders*. 2014;163:115-124. doi:10.1016/j.jad.2013.12.028

46. Forde R, Peters S, Wittkowski A. Recovery from postpartum psychosis: a systematic review and metasynthesis of women's and families' experiences. *Archives of Women's Mental Health*. 2020;23(5):597-612. doi:10.1007/s00737-020-01025-z

For peer review only

For peer review only - http://bmjopen.bmj.com/site/about/guidelines.xhtml

PRISMA 2020 Checklist

Section and Topic	Item #	Checklist item	Location where item is reported
**TITLE**			
Title	1	Identify the report as a systematic review.	1
**ABSTRACT**			
Abstract	2	See the PRISMA 2020 for Abstracts checklist.	3
**INTRODUCTION**			
Rationale	3	Describe the rationale for the review in the context of existing knowledge.	5
Objectives	4	Provide an explicit statement of the objective(s) or question(s) the review addresses.	5
**METHODS**			
Eligibility criteria	5	Specify the inclusion and exclusion criteria for the review and how studies were grouped for the syntheses.	6 & Appendix
Information sources	6	Specify all databases, registers, websites, organisations, reference lists and other sources searched or consulted to identify studies. Specify the date when each source was last searched or consulted.	6 & Appendix
Search strategy	7	Present the full search strategies for all databases, registers and websites, including any filters and limits used.	Appendix
Selection process	8	Specify the methods used to decide whether a study met the inclusion criteria of the review, including how many reviewers screened each record and each report retrieved, whether they worked independently, and if applicable, details of automation tools used in the process.	6 & Appendix
Data collection process	9	Specify the methods used to collect data from reports, including how many reviewers collected data from each report, whether they worked independently, any processes for obtaining or confirming data from study investigators, and if applicable, details of automation tools used in the process.	6 & Appendix
Data items	10a	List and define all outcomes for which data were sought. Specify whether all results that were compatible with each outcome domain in each study were sought (e.g. for all measures, time points, analyses), and if not, the methods used to decide which results to collect.	6 & Appendix
	10b	List and define all other variables for which data were sought (e.g. participant and intervention characteristics, funding sources). Describe any assumptions made about any missing or unclear information.	6 & Appendix
Study risk of bias assessment	11	Specify the methods used to assess risk of bias in the included studies, including details of the tool(s) used, how many reviewers assessed each study and whether they worked independently, and if applicable, details of automation tools used in the process.	6-7 & Appendix
Effect measures	12	Specify for each outcome the effect measure(s) (e.g. risk ratio, mean difference) used in the synthesis or presentation of results.	N/A
Synthesis methods	13a	Describe the processes used to decide which studies were eligible for each synthesis (e.g. tabulating the study intervention characteristics and comparing against the planned groups for each synthesis (item #5)).	7
	13b	Describe any methods required to prepare the data for presentation or synthesis, such as handling of missing summary statistics, or data conversions.	7
	13c	Describe any methods used to tabulate or visually display results of individual studies and syntheses.	7
	13d	Describe any methods used to synthesize results and provide a rationale for the choice(s). If meta-analysis was performed, describe the model(s), method(s) to identify the presence and extent of statistical heterogeneity, and software package(s) used.	7
	13e	Describe any methods used to explore possible causes of heterogeneity among study results (e.g. subgroup analysis, meta-regression).	N/A
	13f	Describe any sensitivity analyses conducted to assess robustness of the synthesized results.	7 & Appendix
Reporting bias assessment	14	Describe any methods used to assess risk of bias due to missing results in a synthesis (arising from reporting biases).	7 & Appendix

For peer review only - http://bmjopen.bmj.com/site/about/guidelines.xhtml

PRISMA 2020 Checklist

Section and Topic	Item #	Checklist item	Location where item is reported
Certainty assessment	15	Describe any methods used to assess certainty (or confidence) in the body of evidence for an outcome.	7 & Appendix
**RESULTS**			
Study selection	16a	Describe the results of the search and selection process, from the number of records identified in the search to the number of studies included in the review, ideally using a flow diagram.	Figure 1
	16b	Cite studies that might appear to meet the inclusion criteria, but which were excluded, and explain why they were excluded.	Figure 1
Study characteristics	17	Cite each included study and present its characteristics.	7 & Appendix
Risk of bias in studies	18	Present assessments of risk of bias for each included study.	Appendix
Results of individual studies	19	For all outcomes, present, for each study: (a) summary statistics for each group (where appropriate) and (b) an effect estimate and its precision (e.g. confidence/credible interval), ideally using structured tables or plots.	N/A
Results of syntheses	20a	For each synthesis, briefly summarise the characteristics and risk of bias among contributing studies.	Appendix
	20b	Present results of all statistical syntheses conducted. If meta-analysis was done, present for each the summary estimate and its precision (e.g. confidence/credible interval) and measures of statistical heterogeneity. If comparing groups, describe the direction of the effect.	N/A
	20c	Present results of all investigations of possible causes of heterogeneity among study results.	N/A
	20d	Present results of all sensitivity analyses conducted to assess the robustness of the synthesized results.	Appendix
Reporting biases	21	Present assessments of risk of bias due to missing results (arising from reporting biases) for each synthesis assessed.	N/A
Certainty of evidence	22	Present assessments of certainty (or confidence) in the body of evidence for each outcome assessed.	Appendix
**DISCUSSION**			
Discussion	23a	Provide a general interpretation of the results in the context of other evidence.	18-21
	23b	Discuss any limitations of the evidence included in the review.	21
	23c	Discuss any limitations of the review processes used.	21
	23d	Discuss implications of the results for practice, policy, and future research.	21
**OTHER INFORMATION**			
Registration and protocol	24a	Provide registration information for the review, including register name and registration number, or state that the review was not registered.	4
	24b	Indicate where the review protocol can be accessed, or state that a protocol was not prepared.	4
	24c	Describe and explain any amendments to information provided at registration or in the protocol.	N/A
Support	25	Describe sources of financial or non-financial support for the review, and the role of the funders or sponsors in the review.	4
Competing interests	26	Declare any competing interests of review authors.	N/A
Availability of data, code and other materials	27	Report which of the following are publicly available and where they can be found: template data collection forms; data extracted from included studies; data used for all analyses; analytic code; any other materials used in the review.	Appendix

For peer review only

For peer review only - http://bmjopen.bmj.com/site/about/guidelines.xhtml

PRISMA 2020 Checklist

From: Page MJ, McKenzie JE, Bossuyt PM, Boutron I, Hoffmann TC, Mulrow CD, et al. The PRISMA 2020 statement: an updated guideline for reporting systematic reviews. BMJ 2021;372:n71. doi: 10.1136/bmj.n71

For more information, visit: http://www.prisma-statement.org/

For peer review only

A meta-review of the barriers and facilitators to women accessing perinatal mental health care

Journal:	*BMJ Open*
Manuscript ID	bmjopen-2022-066703.R1
Article Type:	Original research
Date Submitted by the Author:	28-Mar-2023

Complete List of Authors:	Webb, Rebecca; City University of London, Centre for Maternal and Child Health Research<br>Uddin, Nazihah; City University of London, Centre for Maternal and Child Health Research<br>Constantinou, Georgie; City University of London, Centre for Maternal and Child Health Research<br>Ford, Elizabeth; Brighton and Sussex Medical School<br>Easter, Abigail; King's College London, Department of Women and Children's Health, School of Life Course Sciences, ; King's College London, Section of Women's Mental Health, Institute of Psychiatry, Psychology & Neuroscience<br>Shakespeare, Judy; Retired,<br>Hann, Agnes; National Childbirth Trust<br>Roberts, Nia; University of Oxford, Bodleian Health Care Libraries,<br>Alderdice, Fiona; National Perinatal Epidemiology Unit, Nuffield Department of Population Health<br>Sinesi, Andrea; University of Stirling, Nursing, Midwifery and Allied Health Professions Research Unit (NMAHP RU)<br>Coates, Rose ; City University of London, Centre for Maternal and Child Health Research<br>Hogg, Sally; Parent Infant Foundation<br>Ayers, Susan; City University, Centre for Maternal and Child Health Research<br>Study Team, The MATRIx; City University of London, Centre for Maternal and Child Health Research

**Primary Subject Heading**:	Mental health
Secondary Subject Heading:	Evidence based practice, Obstetrics and gynaecology, Public health
Keywords:	PSYCHIATRY, Health policy < HEALTH SERVICES ADMINISTRATION & MANAGEMENT, Depression & mood disorders < PSYCHIATRY, Anxiety disorders < PSYCHIATRY

SCHOLARONE™
Manuscripts

I, the Submitting Author has the right to grant and does grant on behalf of all authors of the Work (as defined in the below author licence), an exclusive licence and/or a non-exclusive licence for contributions from authors who are: i) UK Crown employees; ii) where BMJ has agreed a CC-BY licence shall apply, and/or iii) in accordance with the terms applicable for US Federal Government officers or employees acting as part of their official duties; on a worldwide, perpetual, irrevocable, royalty-free basis to BMJ Publishing Group Ltd ("BMJ") its licensees and where the relevant Journal is co-owned by BMJ to the co-owners of the Journal, to publish the Work in this journal and any other BMJ products and to exploit all rights, as set out in our licence.

The Submitting Author accepts and understands that any supply made under these terms is made by BMJ to the Submitting Author unless you are acting as an employee on behalf of your employer or a postgraduate student of an affiliated institution which is paying any applicable article publishing charge ("APC") for Open Access articles. Where the Submitting Author wishes to make the Work available on an Open Access basis (and intends to pay the relevant APC), the terms of reuse of such Open Access shall be governed by a Creative Commons licence – details of these licences and which Creative Commons licence will apply to this Work are set out in our licence referred to above.

Other than as permitted in any relevant BMJ Author's Self Archiving Policies, I confirm this Work has not been accepted for publication elsewhere, is not being considered for publication elsewhere and does not duplicate material already published. I confirm all authors consent to publication of this Work and authorise the granting of this licence.

For peer review only

A meta-review of the barriers and facilitators to women accessing perinatal mental health care

Rebecca Webb, Nazihah Uddin, Georgina Constantinou, Elizabeth Ford, Abigail Easter, Judy Shakespeare, Agnes Hann, Nia Roberts, Fiona Alderdice, Andrea Sinesi, Rose Coates, Sally Hogg, Susan Ayers and the MATRIx Study Team

Rebecca Webb (PhD) Centre for Maternal and Child Health, City, University of London, EC1V 0HB, United Kingdom

Nazihah Uddin (MSc) Centre for Maternal and Child Health, City, University of London, EC1V 0HB, United Kingdom

Georgina Constantinou (PhD) Centre for Maternal and Child Health, City, University of London, EC1V 0HB, United Kingdom

Elizabeth Ford (PhD) Brighton & Sussex Medical School, Village Way, Falmer, BN1 9PH, United Kingdom

Abigail Easter (PhD) Department of Women and Children's Health, School of Life Course Sciences, King's College London, London, SE5 8AB, United Kingdom

Judy Shakespeare (BM, BCh) Retired GP, Oxford, OX2 7AG

Agnes Hann (PhD) NCT, Brunel House, 11 The Promenade, Clifton Down, Bristol BS8 3NG

Nia Roberts (PhD) Nuffield Department of Population Health, Bodleian Health Care Libraries, Oxford, OX3 9DU

Fiona Alderdice (Professor, PhD) National Perinatal Epidemiology Unit, Nuffield Department of Population Health, University of Oxford, Oxford, OX3 7LF

For peer review only

Andrea Sinesi (PhD) Nursing, Midwifery and Allied Health Professions Research Unit (NMAHP RU), University of Stirling, FK9 4LA, United Kingdom

Rose Coates (PhD), Centre for Maternal and Child Health, City, University of London, EC1V 0HB, United Kingdom

Sally Hogg (MA) Parent Infant Foundation, Room 202, Place2Be, 175 St John's Street, London, EC1V 4LW

Susan Ayers (Professor, PhD) Professor of Maternal and Child Health, Centre for Maternal and Child Health, City, University of London, EC1V 0HB, United Kingdom

The MATRIx Study Team: Elaine Clark, Helen Cheyne, Evelyn Frame, Simon Gilbody, Agnes Hann, Sarah McMullen Camilla Rosan, Debra Salmon, Andrea Sinesi, Clare Thompson, and Louise Williams.

*Corresponding Author: Rebecca Webb, Centre for Maternal and Child Health, City, University of London, EC1V 0HB, United Kingdom; Rebecca.Webb.2@city.ac.uk

Acknowledgements: We gratefully acknowledge the contribution of the MATRIx study team: Elaine Clark, Evelyn Frame, Simon Gilbody, Sarah McMullen, Camilla Rosan, Debra Salmon, Clare Thompson, and Louise R Williams.

Word count: 5478

Abstract

Perinatal mental health (PMH) problems are common and can have an adverse impact on women and their families. However, research suggests that a substantial proportion of women with PMH problems do not access care.

Objectives: To synthesise the results from previous systematic reviews of barriers and facilitators to women to seeking help, accessing help and engaging in PMH care, and suggest recommendations for clinical practice and policy.

Design: A meta-review of systematic reviews

Review methods: Seven databases were searched and reviews using a PRISMA (Preferred Reporting Items for Systematic Reviews and Meta Analyses) search strategy focusing on the views of women seeking help and accessing PMH care were included. Data were analysed using thematic synthesis. Assessing the Methodological Quality of Systematic Reviews (AMSTAR 2) was used to assess review methodology. To improve validity of results, a qualitative sensitivity analysis was carried out to assess whether themes remained consistent across all reviews, regardless of their quality rating.

Results: A total of 32 reviews were included. A wide range of barriers and facilitators to women accessing perinatal mental health care were identified, that mapped across a multi-level model of influential factors (individual, healthcare professional, interpersonal, organisational, political and societal) and across the care pathway (from decision to consult to receiving care). Evidence based recommendations to support the design and delivery of perinatal mental health care were produced based on identified barriers and facilitators.

Conclusion: The identified barriers and facilitators point to a complex interplay of many factors highlighting the need for an international effort to increase awareness of PMH difficulties,

For peer review only

reduce mental health stigma, and provide woman-centred, flexible care, delivered by well-trained and culturally sensitive primary care, maternity and psychiatric health professionals.

Funding: NIHR128068

Registration: PROSPERO CRD42019142854

Keywords: Perinatal mental health; Implementation; Mental health services; barriers; facilitators

For peer review only

Strengths and Weaknesses

- This meta-review synthesised a large amount of information from 32 systematic reviews
- Title and abstracts and full texts were double screened by two reviewers
- Only reviews published in academic journals and written in English language were included.
- Only 10% of studies had duplicate data extraction.

For peer review only

Introduction

Perinatal mental health (PMH) problems commonly consist of anxiety disorders, depression, post-traumatic stress disorder (PTSD), and stress-related conditions such as adjustment disorder. They can also include more severe difficulties such as postpartum psychosis, and many disorders are co-morbid(1,2).

PMH problems can adversely impact women and their families. They are associated with obstetric physical health complications, such as increased risk of pre-eclampsia, antepartum and postpartum haemorrhage, placental abruption, stillbirth(3–5) and pre-term birth(6,7). Furthermore, suicide is a leading cause of death during the perinatal period in higher-income countries (HIC) (accounting for 5 to 20% of maternal deaths)(2,8,9), and it accounts for between 0.65-3.55% of pregnancy-related deaths in lower-middle income countries (LMIC)(10). Research has also found PMH problems are associated with a child's cognitive and language development(11–14), can lead to behavioural problems in children(13,15,16), and may mean a woman's child is at an increased risk for developing mental health difficulties themselves(17–19). Furthermore, PMH problems can impact on a woman's relationships with her partner, such as a decline in relationship satisfaction(20) increased strain on the couple relationship(21,22) and relationship breakdown(23). There is also a large cost to society and healthcare services with PMH problems costing the UK £8.1 billion every year(24).

Evidence-based PMH care can reduce the negative impacts to women and their families. For example cognitive behavioural therapy (CBT)(25), psychological therapies (26), and certain anti-depressant medications(27) have been shown to be effective in reducing PMH symptoms.

Globally, evidence-based guidelines exist for PMH care. The World Health Organization Millennium Development Goal 5 is to improve maternal health(28), and states a mental health

For peer review only

component should be incorporated as an integral part of maternal health policies, plans and activities in all countries(29). However, research suggests access to PMH care is variable(30–33) with only 30-50% of women with PMH problems identified and less than 10% referred to specialist care(34–36). This variable access could be due to multiple reasons, such as difficulties with implementing PMH services(37) or due to barriers experienced by women.

Multiple systematic reviews have explored women's barriers and facilitators to accessing PMH care. Each systematic review varies slightly in relation to its aim and methods making it hard to extract the information needed to design PMH services in a more accessible way. A systematic review of systematic reviews, or a meta-review is arguably the most suitable way to synthesise results by combining evidence of multiple reviews into a single body of evidence allowing comparison of results from multiple reviews. This would make it easier for healthcare providers and policy makers to access the information and use it to inform their decisions(38,39). Therefore, the primary aim of this research is to determine the key barriers and facilitators to women deciding to seek help, accessing help, and engaging in PMH care using a meta-review.

Method

The protocol for this review has been registered on PROSPERO (CRD42020193107) (see Appendix 1).

Patient and Public Involvement

This project was developed with PPI representatives from the NCT in England and the Maternal Mental Health Change Agents (MMHCA), a group of women with lived experience of PMH difficulties in Scotland.

Data sources and searches

For peer review only

Searches were carried out by NR in CINAHL (1982- present); Embase (1974 – present); Medline (1946- present); PsycINFO (1806 – present), Cochrane, SCOPUS and TRIP (Turning Research into practice) Medical Database. Searches were completed on 4th August 2021 and forward and backward searches were completed by 8th September 2021. See Appendix 2 & 3 for full search syntax and results.

Study selection

Reviews were included if they used a Preferred Reporting Items for Systematic Reviews and Meta Analyses (PRISMA(40)) search strategy and focused on the views of women seeking help and accessing care for perinatal mental illness. See Appendix 4 for full inclusion criteria. Search results were imported into Endnote and duplicates and papers not meeting initial inclusion criteria (foetal distress, oxidative stress, and Non-English papers due to translation times and costs) were removed by NR. The remaining studies were imported into Eppi-Reviewer 4, where results were double screened by title and abstract by two people (RW & GC). Following this, full text screening was carried out by two people (RW & GC).

Data collection process and data items

Data extraction was carried out using Microsoft Excel by RW. Double coding of extracted data was carried out for a proportion of included reviews (n = 3, 10%) by GC.

Critical appraisal of reviews

Methodology sections of included systematic reviews were appraised using the Assessing the Methodological Quality of Systematic Reviews (AMSTAR 2(41)). A decision was made to include reviews where confidence in results was evaluated as low and critically low because these reviews focused more on marginalised women, such as refugees, migrants, women with a low income, and women living in lower-middle income countries, to ensure the experiences of

For peer review only

these seldom-heard women were captured. To improve the validity of results, a qualitative sensitivity analysis was carried out to assess whether themes remained consistent across all reviews, regardless of their quality rating (see Appendix 5-8).

Synthesis of results

Results were analysed by RW using a thematic synthesis(42) in NVivo and Microsoft Excel. Themes were mapped onto a multi-level framework adapted from Ferlie and Shortell's Levels of Change framework (individual level, group/team level, organisational level, and larger system/environment level(43) and utilised in a previous systematic review on barriers and facilitators to implementation of PMH care carried out by the review authors(37). The levels identified in the previous review reflect the reviewed literature and the complexities of the health services. The levels identified were individual, health professional, interpersonal, organisational, political and societal. These will be described in more detail below. The mapping of descriptive themes was developed deductively from the initial theoretical framework and then inductively revised as new themes emerged. The mapping of descriptive themes was discussed by all review authors before being finalised. Differences of opinion were resolved through discussion. Recommendations were developed for policy and practice based on the most cited themes. For a more detailed methodology please see Appendix 5.

Results

Review selection and review characteristics

Screening identified 32 reviews to be included in the meta-review (see Figure 1). See Appendix 9-10 for review characteristics).

Risk of bias within studies

For peer review only

Most reviews were evaluated as having low (n = 14) or critically low (n = 5) confidence with their results. The remainder had moderate (n = 8) or high (n = 5) confidence (see Appendix 11).

Synthesis of results.

Determining the barriers and facilitators to women help-seeking and accessing PMH care

A total of six overarching themes, mapped onto a multi-level framework(43), made up of 62 subthemes were identified (see Appendix 12). The multi-level framework is an extension of Ferlie and Shortell's Levels of Change framework(43) with six levels, instead of four. The first level is the individual level, which reflects factors related to the person themselves. The second level is health professional, which reflects factors related to the health professional. Interpersonal refers to the relationship between women and health professionals, this is an extension of Ferlie and Shortell's work and was included because this theme was represented in the literature(37). The next theme is organisational, which relates to how the organisation is run, and the type of care the organisation delivers. The literature provided multiple examples of how women wanted their care designed. As the organisation is in charge of designing and providing care, ideal care was mapped as a sub-theme under this theme. The political level relates to the policies and governing that may impact on women, and healthcare. The societal level relates to larger societal factors, such as stigma. It is important to note that these levels do not exist in isolation but often impact one another, for example lack of political funding and policy will have a negative impact on how an organisation is run, staff burnout and thus the care delivered to women

Each level of the multi-level framework (Figure 2) maps on to at least one part of the care pathway (Figure 3). Each level of the multi-level framework will be outlined below, and within each level, the most cited barriers and facilitators will be presented following the chronology of

For peer review only

the care pathway outlined in Figure 3. Reflective quotes can be found in Table 1 and are labelled to reflect the theme they are related to. Recommendations for practice and policy can be found in Table 2. It should be noted that the review draws on international evidence, and not all the factors identified will exist to the same extent in all places.

For peer review only

Table 1. Themes and reflective quotes

**Individual level**	
**Theme**	**Quote Number. Reflective quote**
Not understanding what perinatal mental illness is	**1.** 'I don't know what postnatal depression is — how you're supposed to feel, look, or whatever. I don't know. I have no idea … what exactly is postnatal depression? What are you supposed to be doing, saying, or whatever? I don't know.' (44)
Physical factors as the cause	**2.** 'I thought it was just lack of sleep and this heavy cold. I thought that after a good night's sleep it would get better, and I would be able to manage' (44)
Recognising something is wrong	**3.** 'That's when I thought, you know: "Something is really wrong here, I need to go to the doctors if I'm thinking about killing myself."' (44)
Not understanding the role of health professionals	**4.** 'I don't really know what their job is. Nobody gave me, like, the parameters of this role of the health visitor [maternal and child health nurse]….'(44)
Fear of being seen as a bad mum	**5.** 'I even went in at 3 months and I talked to a health nurse, and I just lied through my teeth because I thought, what are they going to do if they find out I can't be a good mom?'(45)
Supportive family and friends	**6.** 'It was sort of my partner saying to me: "Right, if you don't go, I'm basically making you an appointment … You can't just keep feeling like this."' (44)
**Health professional level**	
**Theme**	**Quote number. Reflective quote**
Health professionals being dismissive/normalising symptoms	**7.** 'I did ask for support, but I didn't really get any. And the health visitor's response — "Well you seem like you're doing all right" – which kind of closes it off, doesn't it' (44)
Health professional's appearing to not have enough time	**8.** 'The health visitor said something like: "You know, in this community we have to look after a thousand and something babies." And that instilled in me the feeling, like: "Oh, they are very busy these people, and I don't have to be bothering them all the time'(44)]
Not recognising women's help-seeking or symptoms	**9.** 'I purposely circled the things 'cos I'm struggling … the health visitor didn't get back to me, which I'm really disappointed about.'(44)
**Interpersonal level**	
**Theme**	**Quote number. Reflective quote**
Trusting relationship between women and health professionals	**10.** 'She's a supplement to my own mother. She's easy to talk to. I depend on her. She's not just there to take care of the baby but for the mothers too. She started a group for us new mothers.' (46)
Language barriers	**11.** 'When the midwife visits, I can only speak the sentences about requesting a translator …

For peer review only

	They said that this kind of service is limited … that is what is difficult being Chinese—language barrier.' (47)
Shared decision making	**12.**'… it would have been good I think to have been listened to about the side effects. I was on a very high dose of Olanzapine [sic] and it just knocks you out …' (48)
**Organisational level**	
**Theme**	**Quote number. Reflective quote**
Assessment acceptability	**13.** 'There's so much more that you want to say, rather than just answering quite closed questions.'(44)
Lack of child care at the service	**14.** 'You have to have someone to look after your baby so who am I going to get to look after [my baby]' (44)
Lack of services, or overstretched services	**15.** 'You shouldn't have to press that danger button of 'I'm gonna self-harm' or 'I'm gonna hurt my children' for someone to help you.' (48)
Lack of collaboration across services	**16.** 'My GP [general practitioner/family doctor] says go the HV [health visitor] and HV says go to GP. I don't know what to do, I need help, don't know where to go, or who to turn to' (47)
Lack of continuity of carer	**17.** 'Every time I went to see the midwife, or…, I always had somebody different, and I don't want to tell 10 people my story.' (48)
**Ideal care**	
**Theme**	**Quote number. Reflective quote**
Culturally appropriate	**18.** 'In Pakistan we only saw lady professionals, but here you don't have a choice, you have to see the men as well otherwise you don't get to see a doctor...' (49)
**Political level**	
**Theme**	**Quote number. Reflective quote**
Immigration status	**19.** 'Because when you're legal you can take the child to the daycare and look for a job. . . if you don't work, it's like you're dead, being alive.. . .'(50)
Economic status	**20.** '…if she has no money, how is she going to find help [with PPD]?'(51)
**Societal level**	
**Theme**	**Quote number. Reflective quote**
Culture	**21.** 'There is a huge stigma of being mentally ill in the public, but for us Asians there is a double disadvantage. I really fear that work will find out.' Pakistani woman living in the UK (47)
Maternal norms	**22.** 'Mothers tend to think they should always be there. And mothers are supposed to be always rock solid, aren't they? Everyone assumes that.' (52)

For peer review only

Table 2. Recommendations for improving perinatal mental healthcare for women

System level factor	Theme	Recommendation
Individual	Beliefs about health services<br>Beliefs about health professionals<br>Beliefs about mental illness<br>Fear of judgement<br>Logistics	Improvement of mental health literacy for, women, family, friends, and all who come into contact with perinatal women[1,2]<br>Free access to healthcare[3]<br>Woman-centred care[4]
Healthcare professional	Characteristics<br>Time<br>Training and knowledge	Attend training in communication skills[5]<br>Attend training in perinatal mental health to reduce stigma[5]<br>Attend training in cross-cultural presentations of mental health difficulties[5]
Interpersonal	Relationship and rapport<br>Language barriers<br>Shared decision making<br>Communication<br>Information provision	Healthcare professional to attend training in communication skills[5]<br>Healthcare professional to attend training in perinatal mental health to reduce stigma[5]<br>Healthcare professional to attend training in cross-cultural presentations of mental health difficulties[5]<br>Provision of continuity of carer[4]
Organisational (including ideal care)	Lack of services/overstretched services<br>Characteristics of the service including continuity of carer<br>Collaboration across services	Individualised and culturally appropriate care co-designed with women.[4]<br>Improved funding for perinatal mental health services.[3]<br>Improved guidance for implementing perinatal mental health care*.[1,6]
Political	Immigration and economic status<br>Healthcare costs	Equal rights to healthcare[3]<br>Free healthcare[3]<br>Laws to protect those with immigration status[3]

For peer review only

		International policy that supports the funding and implementation of personalised culturally appropriate care[1]
Societal	Stigma<br>Culture<br>Maternal norms	International, culturally sensitive public mental health campaigns to increase knowledge about mental illness and improve attitudes about people with mental illness(53–58)[1]<br><br>The continuation of international policies to promote gender equality, higher paid parental leave (59), increased opportunity for women in the labour force (60–62), the right to access contraception and abortion(63).[3]

*Recommendations for implementing perinatal mental health assessment, care and treatment can be found in (37)

1. Recommendations for public health services (e.g., the NHS, the European Public Health Association, Public Health Association of Australia etc.)
2. Recommendations for third sector organisations (e.g., the National Childbirth Trust, UK; The Babes Project, Australia etc.)
3. Recommendation for the government
4. Recommendation for organisation
5. Recommendations for healthcare professionals
6. Recommendations for academics/researchers

For peer review only

Individual level factors.

Individual level factors were identified by 25 reviews. Barriers that prevented women from *help-seeking* included **not understanding the role of health professionals** (n = 6), **not knowing what perinatal mental illness is** (Quote no.1, Table 1) (n = 14) and therefore attributing it to **external causes** (e.g. job loss; n = 8), **physical causes** (Quote 2) such as hormones (n = 9), or symptoms being a **normal part of motherhood** (n = 8). Dealing with symptoms by **ignoring them** (n = 6) or **minimising them** (n = 12) were barriers to help-seeking. **Not knowing where to go** (n = 7); **fear of being seen as a bad mum** or **social services involvement** (n = 7) and **lack of support** from family and friends (n = 9) were also barriers. A facilitator was **recognising something is wrong** (n = 9).

Barriers to *disclosing symptoms* were **not understanding health professional's role,** (Quote 4) perceiving them as agents of social control (n = 4), fear of **social services involvement** and the removal of their child (n = 7), and fears of **being judged as a bad mum** (Quote 5) (n = 8). The most cited barrier to women *accessing care* was **logistical reasons** (n = 13) such as travel costs, lack of childcare and timing of services. Encouragement from **family and friends** (n = 15) and **additional support networks** (n = 8) were all facilitators to *help seeking*, *women accessing care* and *women's experiences of care* (Quote 6). On the other hand, **family and friends'** stigmatising beliefs about perinatal mental illness could act as a barrier.

HP related factors.

HP level factors were reported by 18 reviews. During *first contact* with women, HPs being **dismissive or normalising** women's symptoms (Quote 7) (n = 8), **not recognising women's help-seeking or symptoms** (n = 4) and **appearing to not have enough time** (Quote 8) (n = 3) were barriers to care. A barrier during *assessment* was the **way care was delivered** for

For peer review only - http://bmjopen.bmj.com/site/about/guidelines.xhtml

example, in a formulaic tick-box way, or not being carried out at all (n = 3). The most reported barrier to women's *decision to disclose* was HPs **appearing to not have enough time** (n = 4) or HPs being **dismissive or normalising** women's symptoms (n = 4). **Not recognising women's help-seeking or symptoms** (Quote 9) (n = 2) and women's **perception of HPs knowledge of referral pathways/other services** (n = 3) were barriers to *referral. Accessing care, receiving optimal care* and *women's experiences of care* were mainly influenced by the **characteristics of HPs.** For example, HPs who were trustworthy, responsive, non-judgemental, understanding, caring, interested, warm, empathetic and positive (n = 12) were facilitators. On the other hand, unhelpful or uninterested staff were barriers (n = 2).

Interpersonal factors.

Interpersonal level factors were identified by 14 reviews. The development of a **strong and trusting relationship** with a HP (n = 10) was a facilitator to women deciding to *seek help*, *disclosing* their symptoms, *accessing care*, and a positive *experience* (Quote 10) of care. **Language difficulties** (Quote 11) (n = 6), **shared decision making** (Quote 12) (n = 6) or **poor communication** (n = 4) between women and HPs influenced the *first contact with HPs*, *assessment*, *access,* and *provision of optimal* care.

Organisational factors.

Organisational level factors were identified by 21 reviews. The most cited barrier to *screening/assessment* was low assessment acceptability because of the wording or contents of the tool (Quote 13) (n = 2) or if the tool was delivered in a tick-box way (n = 6). Some women found screening tools particularly problematic if the tool was not in her first language, indicating that cultural factors can overlap with organisational factors. For example, one review reported that certain questions may not elicit true feelings from Vietnamese women living in the UK because

For peer review only

of the shame of admitting to these(64). Further, question Q10 on the EPDS(65) ('the thought of harming myself has occurred to me') was seen as problematic to Arabic, Vietnamese, and Black Caribbean mothers(64) living in the UK or USA, highlighting the need for culturally sensitive and relevant assessment tools.

Access to care was influenced by the **practical characteristics** (Quote 14) (n = 5) of the organisation and services offered, such as a lack of childcare facilities, hard to reach locations, and timing of appointments. A **lack of services or overstretched services** (Quote 15) (n = 7), a **lack of collaboration** (Quote 16) (n = 3) across services, and **lack of continuity of care** (Quote 17) (n = 2) were barriers.

In terms of **ideal care,** women reported wanting care that provided them with an opportunity to talk to someone and discuss their emotional difficulties (n = 8); some women wanted this opportunity within a peer support or group setting (n = 12) and reported that an appropriate peer group could provide them with validation for their feelings (n = 3). Care also needed to be individualised (n = 10), and be culturally sensitive (Quote 18) (n = 8). Women also appreciated care that provided them with information about PMH difficulties (n = 5). Further, the location of the care should be easy to reach or carried out in women's homes (n = 7), and women should not be discharged too early from these services (n = 4).

Political factors.

Political factors were identified by 8 reviews and were defined as factors that governmental agencies have influence over (e.g. poverty, immigration, housing). **Immigration status** (Quote 19) and **economic status** (Quote 20) influenced women's *decision to consult* (n = 7) and *access to care* (n = 5). This is due to the costs of healthcare and women's fear of being deported if they access help. **Economic status** was often exacerbated by **immigration status**

For peer review only

with women reporting not being able to get health insurance due to their immigration status (n = 4). **Economic status** also impacted *Women's experience of care* in terms of women not being able to feel any sense of wellbeing when they were unable to fulfil 'basic needs' such as 'not having enough money to make ends meet'(66) (n = 4).

Societal factors.

Societal factors were identified in 24 reviews. The main societal factors that influenced women's journey along the care pathway were **culture**, societies' norms of what a "good mum" should look like (**maternal norms**), and **stigma**. All these factors intertwine and influence one another. There was only one review that only included studies from Lower Middle-Income Countries (LMICs)(67), therefore these results mainly refer to western cultures.

For women living in sub-Saharan Africa, the **cultural** tradition of confinement meant women felt unable to leave their house for fear of being shamed, acting as a barrier to *accessing care.* This was further exacerbated by the attribution of postnatal ill health to inadequate adherence to tradition(67). Adherence to **cultural** traditions also prevented the *decision to consult* in women who had moved to western countries (n = 7). Two reviews reported that Hispanic women living in the USA felt they needed to remain strong (n = 2), feeling they needed to show that they could cope, and that stigma prevented them from seeking help due to not wanting to be seen as "crazy" or "loco" (Tobin et al., 2018(68), p.97).

Four reviews found that South Asian women living in the UK did not *consult* or *disclose* for similar **cultural** reasons e.g. "for fear of an inability to perform their role as a woman and a mother" (Dennis, p. 325), perceiving symptoms in religious terms "All illness is coming from God" (Button p.e649) , and stigma (Quote 21)(44,47,68,69).

Black African and Caribbean women living in the UK or USA were deterred from

For peer review only

consulting and *disclosing* PMH problems because of the expectation of women to be strong and be able to cope (n = 4), but also the fear of what could happen if women were to seek help (n = 1). Women's cultural backgrounds highlighted the need for culturally sensitive care. The lack of this care was as a barrier to *access* (n = 3). Two reviews explained how Hispanic women living in the USA felt that language barriers, cultural insensitivity, and financial barriers were a barrier to them accessing care. Further, Jordanian women (living in Australia) spoke of being torn between their own cultural practices and Western health advice, having health professionals placing pressure and unrealistic demands upon them to change their beliefs and behaviours(50). Furthermore, during the *provision of care*, some women found the clash between western traditions and cultural traditions a cause of conflict, therefore an understanding of this aided the provision of good care (n = 3).

The **maternal norm** for women to show they are strong, that they can cope and be a good mother (Quote 22) (n = 19), prevented women from *deciding to consult, disclosing, accessing care* and their *experience of care.* The **stigma** of perinatal mental illness (n = 23) prevented women *deciding to consult, disclosing symptoms, accessing car*e, and their *experience of care*.

Discussion

This meta-review identified a wide range of barriers and facilitators to women accessing PMH care, that were influential at different levels as identified in Figure 2. Prior to the completion of this meta-review, research had identified multiple factors that act as barriers to women seeking and accessing help for PMH difficulties. The factors include women not recognising the need to seek help (44,52,70–72), the need for health professionals to receive

For peer review only

training on perinatal mental illness and cultural sensitivity(44–46,49,64,73–75), continuity of care(44–46,48,49,69,70,75), and stigma(44,45,48,50–52,66,68,74,76).

Our findings are in line with previous studies that have investigated barriers and facilitators to perinatal mental health care. This paper further adds to this body of evidence by identifying barriers and facilitators to perinatal mental health care, across the globe, and presenting them on a multi-level model, and at different stages of the care pathway. This provides opportunities for health professionals, service managers and policy makers to identify barriers and facilitators that are most relevant to their context. The mapping of barriers and facilitators in this way, has also led to the development of evidence-based recommendations for design and delivery of perinatal mental health care.

Recommendations for PMH care

The results from this meta-review can be used to inform healthcare providers and policy makers on the optimal characteristics of PMH care and are summarised in Table 2. This meta-review showed a complex interplay of multi-level factors that influence women's help-seeking and access to PMH care. Thus, recommendations for policy and practice also relate to both international level guidelines, and guidelines for national and individual level care. International level guidelines should facilitate more personalised care and should feed into national guidelines and be adopted where appropriate.

Societal factors such as stigma, maternal norms, and culture play a large role in women accessing care. Research suggests that public mental health campaigns can increase knowledge about mental illness and improve attitudes about people with mental illness(58). Therefore, increasing women's, families', those who have regular contact with women in the perinatal period, and the public's mental health literacy through public health campaigns, and education

For peer review only

within the community, such as antenatal education, and at healthcare appointments, should be carried out on an international level.

Maternal norms identified in this meta-review related to women believing that they needed to be strong and show they could cope. There may be some potential to change societal beliefs around maternal norms through increasing societal expectations about fathers' role in the family through more equal parental leave. For example, in countries where parental leave is more equal (e.g. Finland), the uptake of paid paternity leave is higher(59). Changing society's maternal norms could also be done by increasing women's equality. For example, research suggests that stereotypes of what a mother or a woman should look like is beginning to change in countries where women have gained more participation in the labour force(62), and have the right to access contraception and abortion(63). However, research is needed to corroborate these findings.

At the political level, immigration and economic status, and healthcare costs were barriers to women accessing healthcare. The results from this meta-review show how race and gender interact to influence women's experiences of the healthcare system (intersectionality)(77). White women living within their country of birth who try to access PMH care are faced with barriers (e.g. no childcare support), but women of colour, migrant women, or migrant women of colour are faced with additional barriers (e.g. language barriers, structural/systematic discrimination). This finding is supported by research in general healthcare that has found ethnic minority and migrant women are disproportionately affected by existing barriers to accessing healthcare(78). As found in this meta-review, these barriers include language and communication barriers, stigma, the cost of healthcare(79), and the inability to access culturally appropriate services (80). This shows the need for equal rights to healthcare,

For peer review only - http://bmjopen.bmj.com/site/about/guidelines.xhtml

regardless of immigration or economic status. Further, changes at the legislative level are needed to protect those who have migrated to a different country from being penalised for accessing healthcare(79).

At the organisational level this meta-review identified a range of factors that women viewed as ideal care. Women appreciated the opportunity to discuss screening results with HPs and for it not to be filled out as a "tick box" exercise(47). In terms of treatment, women wanted the opportunity to talk to someone (a HP or a peer) about their difficulties(45,46,52,69,81,82). They found peer support offered them a sense of validation which they appreciated(83). To overcome logistical barriers, the location of services should be easily accessible, or in women's homes(45,48,51,64). Further, the length of treatment should be flexible and based on women's needs. Women did not want a "one size fits all" approach but wanted individualised treatment that was culturally appropriate (44–46,48,50,68,72,75,81)

At the interpersonal and HP level the characteristics of the HPs were important, as was their communication with women. Women reported that many HPs normalised their symptoms or were dismissive of their attempts to seek help. This may be a reflection of the heavy workload experienced by many health professionals(84–86). For example, research suggests that consultations where mental health problems are discussed take longer, and health professionals often feel there is not enough time to address concerns fully(86,87). This finding could also be a reflection of inadequate training(88). Within the UK, guidance states that all midwives and health visitors should receive training in order for them to identify, care for and refer perinatal women with mental health difficulties(89). However, a synthesis of 30 studies found that midwives lack the confidence, knowledge, and training to do this(90), therefore training around mental health is important. Another key training need is cultural sensitivity and cross-cultural

For peer review only - http://bmjopen.bmj.com/site/about/guidelines.xhtml

understanding of PMH. Some systematic reviews in this meta-review identified that women were treated in a culturally insensitive way by HPs and that women of colour were less likely to be offered treatment or be asked about their mental health. It has been suggested that training given at medical and nursing school does not do enough to reduce unconscious biases against marginalised groups, which in turn influences treatment provided by healthcare providers(91). Improved interpretation services within perinatal mental health care may aid culturally sensitive care. Another potential way to improve culturally sensitive care is through the recruitment and retention of healthcare providers from diverse backgrounds (92) This strategy has the potential to improve interpersonal relationships between health professionals and patients (93,94), which may therefore increase disclosure of perinatal mental health difficulties to health professionals. In addition, research suggests increased representation of diverse populations in health care is associated with improved communication between health providers (95,96), which therefore may reduce the risk of women falling through gaps in the care pathway.

Further, it has been argued that the way the western world views mental illness is very ethnocentric(97) and that culture and society influences what is viewed as a mental illness(98). This may mean that some women's attempts to seek help are missed by HPs. It is therefore crucial that cultural sensitivity and cross-cultural mental health training is provided to HPs.

In terms of individual level factors, many of these barriers can be improved through the recommendations suggested above. For example, improvement of knowledge around mental health is likely to reduce women's fear of judgement and self-stigma and increase her awareness of the symptoms she is experiencing which may encourage help-seeking(99). Re-design of care, such as providing easily accessible healthcare may reduce the logistical barriers women experience.

For peer review only - http://bmjopen.bmj.com/site/about/guidelines.xhtml

Strengths and limitations

The strength of this meta-review is the synthesis of a large amount of information from 32 systematic reviews from many different countries in order to identify barriers and facilitators to women deciding to seek help, accessing help, and engaging in PMH care. This information was then used to provide recommendations for the design and delivery of care. A limitation of the methodology is that only reviews published in academic journals and written in English language were included. Relevant reviews from health services, charities, third sector organisations, and other grey literature may have been missed. Another limitation is that only 10% of studies had duplicate data extraction. However, concordance was high, and it is therefore unlikely that any key themes were missed. A limitation about the papers included in the meta-review was that the majority of them were rated as having low or critically low quality meaning less confidence can be placed on their results. However, the qualitative sensitivity analysis found that the majority of themes were supported in both the higher quality and lower quality reviews and including all reviews meant there was more focus on marginalised women, such as refugees, migrants and women living in sub-Saharan Africa. This shows that the results from this meta-review can be interpreted with reasonable confidence.

Implications for future research

This review has revealed several limitations with the current evidence base on this topic. Very few systematic reviews (n = 2) addressed the severity of illness, only one review looked at severe PMH difficulties(73) and most reviews (n = 24) focussed on depression. There may be different barriers for other PMH difficulties therefore future research should focus on researching the barriers and facilitators to women with disorders other than depression. Another limitation with the identified reviews is that no reviews specified whether women had given birth to

For peer review only

singletons only, or twins/higher-order multiples. This is important as parents of twins or multiples report unique experiences in accessing PMH care(100). Furthermore, reviews only covered the inclusion of studies carried out in 25 countries, and only one review included studies that were only carried out in LMICs(67). More research is needed in other countries to further aid our understanding of help-seeking in women with perinatal mental illness. In addition, none of the identified reviews included studies from diverse families, including same-sex couples, and the transgender community. It is important that future research recruits more diverse populations to ensure all voices are heard. Most reviews were rated as having low or critically low quality meaning less confidence can be placed on their results. However, the qualitative sensitivity analysis found that most themes were supported in both the higher quality and lower quality reviews and including all reviews meant there was more focus on marginalised women, such as refugees, migrants, and women living in sub-Saharan Africa. This shows that the results from this meta-review can be interpreted with reasonable confidence.

Conclusion

The findings from this review point to a complex interplay of individual and system level factors across different stages of the care pathway that can influence whether women seek help and access care for perinatal mental illness. These factors should all be taken into account by policy makers to improve the identification and treatment of PMH problems. Recommendations for the design and delivery of PMH care have been produced building on the barriers and facilitators identified in this review. The identified barriers and facilitators point to the need for an international effort to reduce mental health stigma, and increase woman-centred, flexible care, delivered by well-trained and culturally competent HPs.

For peer review only

Figure 1. PRISMA Flow Diagram

Figure 2. The MATRIx multi-level model of barriers and facilitators to women accessing perinatal mental health care

Figure 3. Barriers and facilitators mapped onto the MATRIx care pathway

a. Contributorship statement: Rebecca Webb was involved in the design of the research and carried out screening, quality appraisal, analysis, write up of manuscript and editing of manuscript. Nazihah Uddin contributed to screening and quality appraisal of papers and provided detailed feedback on the manuscript. Georgina Constantinou contributed to screening and quality appraisal of papers provided detailed feedback on the manuscript. Elizabeth Ford was involved in the conceptualisation of the project, the design of the research, analysis, and provided detailed feedback on the manuscript. Abigail Easter was involved in the conceptualisation of the project, the design of the research, analysis, and provided detailed feedback on the manuscript. Judy Shakespeare was involved in the conceptualisation of the project, the design of the research, analysis, and provided detailed feedback on the manuscript. Agnes Hann provided PPI input and detailed feedback on the manuscript. Nia Roberts completed the literature searches. Fiona Alderdice contributed to the design of the research and provided detailed feedback on the manuscript. Andrea Sinesi contributed to the design of the research and provided detailed feedback on the manuscript. Rose Coates contributed to the design of the research and provided detailed feedback on the manuscript. Sally Hogg contributed to the design of the research and provided detailed feedback on the manuscript. Susan Ayers was the project manager, was involved in the conceptualisation of the project, the design of the research, analysis, and provided detailed feedback on the manuscript. The MATRIx study team Elaine Clark, Evelyn

For peer review only

Frame, Simon Gilbody, Sarah McMullen, Camilla Rosan, Debra Salmon, Clare Thompson, and Louise R Williams for their input throughout the project.

b. Competing interests: None

c. Funding: National Institute for Health Research. Health Services and Delivery Research Programme. NIHR128068

d. Data sharing statement: Data is available on request to the corresponding author: Rebecca.Webb.2@city.ac.uk

e. Ethics statement: This study does not involve human participants. This is an evidence synthesis, therefore approval by an ethics committee is not necessary.

For peer review only

References

1. Falah-Hassani K, Shiri R, Dennis CL. The prevalence of antenatal and postnatal co-morbid anxiety and depression: A meta-analysis. Psychological Medicine. 2017.

2. Knight M, Bunch K, Tuffnell D, Shakespeare J, Kotnis R, Kenyon S, et al. Saving Lives, Improving Mothers' Care: Lessons learned to inform maternity care from the UK and Ireland Confidential Enquiries into Maternal Deaths and Morbidity 2016-18. MBRRACE-UK - Mothers and Babies: Reducing Risk throught Audits and Confidential Enquiries across the UK. Oxford; 2020.

3. Vigod SN, Kurdyak PA, Dennis CL, Gruneir A, Newman A, Seeman M V, et al. Maternal and newborn outcomes among women with schizophrenia: a retrospective population-based cohort study. BJOG Int J Obstet Gynaecol. 2014;121(5):566–74.

4. McAllister-Williams RH, Baldwin DS, Cantwell R, Easter A, Gilvarry E, Glover V, et al. British Association for Psychopharmacology consensus guidance on the use of psychotropic medication preconception, in pregnancy and postpartum 2017. J Psychopharmacol (Oxf). 2017;31(5):519–52.

5. Rusner M, Berg M, Begley C. Bipolar disorder in pregnancy and childbirth: a systematic review of outcomes. BMC Pregnancy Childbirth. 2016;16(1):1–18.

6. Glover V. Maternal stress during pregnancy and infant and child outcome. In: The Oxford handbook of perinatal psychology. 2016.

7. Ding XX, Wu Y Le, Xu SJ, Zhu RP, Jia XM, Zhang SF, et al. Maternal anxiety during pregnancy and adverse birth outcomes: A systematic review and meta-analysis of prospective cohort studies. Journal of Affective Disorders. 2014.

8. Grigoriadis S, Wilton AS, Kurdyak PA, Rhodes AE, VonderPorten EH, Levitt A, et al. Perinatal suicide in Ontario, Canada: a 15-year population-based study. Cmaj. 2017;189(34):E1085–92.

9. Palladino CL, Singh V, Campbell J, Flynn H, Gold K. Homicide and suicide during the perinatal period: findings from the National Violent Death Reporting System. Obstet Gynecol. 2011;118(5):1056.

10. Fuhr DC, Calvert C, Ronsmans C, Chandra PS, Sikander S, De Silva MJ, et al. Contribution of suicide and injuries to pregnancy-related mortality in low-income and middle-income countries: a systematic review and meta-analysis. Lancet Psychiatry. 2014;1(3):213–25.

11. Murray L, Fiori-Cowley A, Hooper R, Cooper P. The Impact of Postnatal Depression and Associated Adversity on Early Mother-Infant Interactions and Later Infant Outcome. Child Dev. 1996;

For peer review only

12. Liu Y, Kaaya S, Chai J, McCoy DC, Surkan PJ, Black MM, et al. Maternal depressive symptoms and early childhood cognitive development: A meta-analysis. Psychol Med. 2017;

13. Netsi E, Pearson RM, Murray L, Cooper P, Craske MG, Stein A. Association of persistent and severe postnatal depression with child outcomes. JAMA Psychiatry. 2018;

14. Oyetunji A, Chandra P. Postpartum stress and infant outcome: A review of current literature. Psychiatry Research. 2020.

15. Prenoveau JM, Craske MG, West V, Giannakakis A, Zioga M, Lehtonen A, et al. Maternal postnatal depression and anxiety and their association with child emotional negativity and behavior problems at two years. Dev Psychol. 2017;

16. Letourneau N, Leung B, Ntanda H, Dewey D, Deane AJ, Giesbrecht GF. Maternal and paternal perinatal depressive symptoms associate with 2-and 3-year-old children's behaviour: Findings from the APrON longitudinal study. BMC Pediatr. 2019;

17. O'Donnell M, Maclean MJ, Sims S, Morgan VA, Leonard H, Stanley FJ. Maternal mental health and risk of child protection involvement: Mental health diagnoses associated with increased risk. J Epidemiol Community Health. 2015;

18. Rees S, Channon S, Waters CS. The impact of maternal prenatal and postnatal anxiety on children's emotional problems: a systematic review. Eur Child Adolesc Psychiatry. 2019;

19. Glasheen C, Richardson GA, Fabio A. A systematic review of the effects of postnatal maternal anxiety on children. Archives of Women's Mental Health. 2010.

20. Garthus-Niegel S, Horsch A, Handtke E, von Soest T, Ayers S, Weidner K, et al. The impact of postpartum posttraumatic stress and depression symptoms on couples' relationship satisfaction: A population-based prospective study. Front Psychol. 2018;

21. Nicholls K, Ayers S. Childbirth-related post-traumatic stress disorder in couples: A qualitative study. Br J Health Psychol. 2007;

22. Delicate A, Ayers S, Easter A, McMullen S. The impact of childbirth-related post-traumatic stress on a couple's relationship: a systematic review and meta-synthesis. Journal of Reproductive and Infant Psychology. 2018.

23. Hinton L, Locock L, Knight M. Support for mothers and their families after life-threatening illness in pregnancy and childbirth:a qualitative study in primary care. Br J Gen Pract. 2015;

24. Bauer A, Parsonage M, Knapp M, Iemmi V, Adelaja B. The costs of perinatal mental health problems [Internet]. Centre for Mental Health and London School of Economics; 2014. Available from: https://www.centreformentalhealth.org.uk/sites/default/files/2018-09/costsofperinatal.pdf

For peer review only

25. Huang L, Zhao Y, Qiang C, Fan B. Is cognitive behavioral therapy a better choice for women with postnatal depression? A systematic review and meta-analysis. PLoS ONE. 2018;

26. Loughnan SA, Wallace M, Joubert AE, Haskelberg H, Andrews G, Newby JM. A systematic review of psychological treatments for clinical anxiety during the perinatal period. Archives of Women's Mental Health. 2018.

27. Molyneaux E, Telesia LA, Henshaw C, Boath E, Bradley E, Howard LM. Antidepressants for preventing postnatal depression. Cochrane Database of Systematic Reviews. 2018.

28. World Health Organization. Millennium Development Goals (MDGs). 2000.

29. World Health Organization. Improving Maternal Mental Health. Geneva; 2008.

30. Baron EC, Hanlon C, Mall S, Honikman S, Breuer E, Kathree T, et al. Maternal mental health in primary care in five low- and middle-income countries: a situational analysis. BMC Health Serv Res. 2016 Feb 16;16(1):53.

31. Moore D, Drey N, Ayers S. Use of Online Forums for Perinatal Mental Illness, Stigma, and Disclosure: An Exploratory Model. JMIR Ment Health. 2017;4(1):e6.

32. Kozhimannil KB, Adams AS, Soumerai SB, Busch AB, Huskamp HA. New Jersey's Efforts To Improve Postpartum Depression Care Did Not Change Treatment Patterns For Women On Medicaid. Health Aff (Millwood). 2011;30(2):293–301.

33. Perinatal Anxiety & Depression (PANDA). Submission to the Productivity Commission Mental Health Inquiry: The Social and Economic Benefits of Improving Perinatal Mental Health [Internet]. 2019. Available from: https://www.pc.gov.au/__data/assets/pdf_file/0012/250203/sub674-mental-health.pdf

34. Gavin NI, Meltzer-Brody S, Glover V, Gaynes BN. Is population-based identification of perinatal depression and anxiety desirable? A public health perspective on the perinatal depression care continuum. In: Milgrom J, Gemmill AW, editors. Identifying perinatal depression and anxiety: Evidence-based practice in screening, psychosocial assessment, and management. London: Wiley Blackwell; 2015. p. 11–31.

35. Royal College of Obstetricians & Gynaecologists. Maternal mental health - women's voices [Internet]. RCOG. [cited 2023 Mar 15]. Available from: https://www.rcog.org.uk/for-the-public/rcog-engagement-listening-to-patients/maternal-mental-health-womens-voices/

36. Milgrom J, Gemmill AW. Identifying perinatal depression and anxiety: evidence-based practice in screening, psychosocial assessment and management. John Wiley & Sons; 2015.

37. Webb R, Uddin N, Ford E, Easter A, Shakespeare J, Roberts N, et al. Barriers and facilitators to implementing perinatal mental health care in health and social care settings: a systematic review. Lancet Psychiatry. 2021;8(6):521–34.

For peer review only

38. Grant MJ, Booth A. A typology of reviews: An analysis of 14 review types and associated methodologies. Health Information and Libraries Journal. 2009.

39. Samnani SS, Vaska M, Ahmed S, Turin TC. Review typology: The basic types of reviews for synthesizing evidence for the purpose of knowledge translation. Journal of the College of Physicians and Surgeons Pakistan. 2017.

40. Moher D, Liberati A, Tetzlaff JM, Altman DG. Preferred reporting items for systematic reviews and meta-analyses: the PRISMA statement. PLoS Med. 2009;6(7):e1000097.

41. Shea BJ, Reeves BC, Wells GA, Thuku M, Hamel C, Moran J, et al. AMSTAR 2: A critical appraisal tool for systematic reviews that include randomised or non-randomised studies of healthcare interventions, or both. BMJ. 2017;21:358.

42. Thomas J, Harden A. Methods for the thematic synthesis of qualitative research in systematic reviews. BMC Med Res Methodol. 2008;8(1):1–10.

43. Ferlie EB, Shortell SM. Improving the quality of health care in the United Kingdom and the United States: a framework for change. Milbank Q. 2001;79(2):281–315.

44. Button S, Thornton A, Lee S, Shakespeare J, Ayers S. Seeking help for perinatal psychological distress: a meta-synthesis of women's experiences. Br J Gen Pract. 2017;67(663):e692–9.

45. Hadfield H, Wittkowski A. Women's experiences of seeking and receiving psychological and psychosocial interventions for postpartum depression: a systematic review and thematic synthesis of the qualitative literature. J Midwifery Womens Health. 2017;62(6):723–36.

46. Morrell CJ, Sutcliffe P, Booth A, Stevens J, Scope A, Stevenson M, et al. A systematic review, evidence synthesis and meta-analysis of quantitative and qualitative studies evaluating the clinical effectiveness, the cost-effectiveness, safety and acceptability of interventions to prevent postnatal depression. Health Technol Assess. 2016;20(37):1–414.

47. Sambrook Smith M, Lawrence V, Sadler E, Easter A. Barriers to accessing mental health services for women with perinatal mental illness: Systematic review and meta-synthesis of qualitative studies in the UK. BMJ Open. 2019;9(1):e024803.

48. Megnin-Viggars O, Symington I, Howard LM, Pilling S. Experience of care for mental health problems in the antenatal or postnatal period for women in the UK: a systematic review and meta-synthesis of qualitative research. Arch Womens Ment Health. 2015;18:745–59.

49. Watson H, Harrop D, Walton E, Young A, Soltani H. A systematic review of ethnic minority women's experiences of perinatal mental health conditions and services in Europe. PloS One. 2019;14(1):e0210587.

For peer review only

50. Schmied V, Black E, Naidoo N, Dahlen HG, Liamputtong P. Migrant women's experiences, meanings and ways of dealing with postnatal depression: A meta-ethnographic study. PloS One. 2017;12(3):e0172385.

51. Hansotte E, Payne SI, Babich SM. Positive postpartum depression screening practices and subsequent mental health treatment for low-income women in Western countries: a systematic literature review. Public Health Rev. 2017;38:1–17.

52. Staneva AA, Bogossian F, Wittkowski A. The experience of psychological distress, depression, and anxiety during pregnancy: A meta-synthesis of qualitative research. Midwifery. 2015;31(6):563–73.

53. Dietrich S, Mergl R, Freudenberg P, Althaus D, Hegerl U. Impact of a campaign on the public's attitudes towards depression. Health Educ Res. 2010;25(1):135–50.

54. Evans-Lacko S, Malcolm E, West K, Rose D, London J, Rüsch N, et al. Influence of Time to Change's social marketing interventions on stigma in England 2009-2011. Br J Psychiatry. 2013;202(s55):s77–88.

55. Jorm AF, Jorm AF, Christensen H, Griffiths KM, Jorm AF, Christensen H, et al. The impact of beyondblue: the national depression initiative on the Australian public's recognition of depression and beliefs about treatments. Aust N Z J Psychiatry. 2005;39(4):248–54.

56. Jorm AF, Christensen H, Griffiths KM. Changes in depression awareness and attitudes in Australia: the impact of beyondblue: the national depression initiative. Aust N Z J Psychiatry. 2006;40(1):42–6.

57. Makowski AC, Mnich EE, Ludwig J, Daubmann A, Bock T, Lambert M, et al. Changes in beliefs and attitudes toward people with depression and schizophrenia–results of a public campaign in Germany. Psychiatry Res. 2016;237:271–8.

58. Sampogna G, Bakolis I, Evans-Lacko S, Robinson E, Thornicroft G, Henderson C. The impact of social marketing campaigns on reducing mental health stigma: Results from the 2009–2014 Time to Change programme. Eur Psychiatry. 2017;40:116–22.

59. OECD - Social Policy Division. Parents' use of childbirth-related leave [Internet]. 2019. Available from: https://www.oecd.org/els/family/PF2-2-Use-childbirth-leave.pdf

60. Eagly AH, Nater C, Miller DI, Kaufmann M, Sczesny S. Gender stereotypes have changed: A cross-temporal meta-analysis of US public opinion polls from 1946 to 2018. Am Psychol. 2020;75(3):301.

61. Lopez-Zafra E, Garcia-Retamero R. Do gender stereotypes change? The dynamic of gender stereotypes in Spain. J Gend Stud. 2012;21(2):169–83.

For peer review only

62. Valiquette-Tessier SC, Gosselin J, Young M, Thomassin K. A literature review of cultural stereotypes associated with motherhood and fatherhood. Marriage Fam Rev. 2019;55(4):299–329.

63. Centre for Reproductive Rights. The World's Abortion Laws. Resources. [Internet]. 2021. Available from: https://reproductiverights.org/worldabortionlaws

64. Brealey SD, Hewitt C, Green JM, Morrell J, Gilbody S. Screening for postnatal depression– is it acceptable to women and healthcare professionals? A systematic review and meta-synthesis. J Reprod Infant Psychol. 2010;28(4):328–44.

65. Cox JL, Holden JM, Sagovsky R. Detection of Postnatal Depression: Development of the 10-item Edinburgh Postnatal Depression Scale. Br J Psychiatry. 1987 Jun;150(6):782–6.

66. Lucas G, Olander EK, Ayers S, Salmon D. No straight lines – young women's perceptions of their mental health and wellbeing during and after pregnancy: a systematic review and meta-ethnography. BMC Womens Health. 2019;19(1):152.

67. Wittkowski A, Gardner PL, Bunton P, Edge D. Culturally determined risk factors for postnatal depression in Sub-Saharan Africa: A mixed method systematic review. J Affect Disord. 2014 Jul 1;163:115–24.

68. Tobin C, Napoli D, Beck C. Refugee and Immigrant Women's Experience of Postpartum Depression: A Meta-Synthesis. J Transcult Nurs Off J Transcult Nurs Soc [Internet]. 2018 [cited 2023 Mar 15];29(1). Available from: https://pubmed.ncbi.nlm.nih.gov/28826328/

69. Dennis CL, Chung-Lee L. Postpartum Depression Help-Seeking Barriers and Maternal Treatment Preferences: A Qualitative Systematic Review. Birth. 2006;33(4):323–31.

70. Slade PP, Molyneux DR, Watt DA. A systematic review of clinical effectiveness of psychological interventions to reduce post traumatic stress symptoms following childbirth and a meta-synthesis of facilitators and barriers to uptake of psychological care. J Affect Disord. 2021 Feb 15;281:678–94.

71. Sorsa MA, Kylmä J, Bondas TE. Contemplating Help-Seeking in Perinatal Psychological Distress—A Meta-Ethnography. Int J Environ Res Public Health. 2021 Jan;18(10):5226.

72. Viveiros CJ, Darling EK. Perceptions of barriers to accessing perinatal mental health care in midwifery: A scoping review. Midwifery. 2019 Mar 1;70:106–18.

73. Forde R, Peters S, Wittkowski A. Recovery from postpartum psychosis: a systematic review and metasynthesis of women's and families' experiences. Arch Womens Ment Health. 2020 Oct 1;23(5):597–612.

74. Jones A. Help Seeking in the Perinatal Period: A Review of Barriers and Facilitators. Soc Work Public Health. 2019;34(7):596–605.

For peer review only - http://bmjopen.bmj.com/site/about/guidelines.xhtml

75. Scope A, Booth A, Morrell CJ, Sutcliffe P, Cantrell A. Perceptions and experiences of interventions to prevent postnatal depression. A systematic review and qualitative evidence synthesis. J Affect Disord. 2017 Mar 1;210:100–10.

76. Giscombe T, Hui A, Stickley T. Perinatal mental health amongst refugee and asylum-seeking women in the UK. Ment Health Rev J. 2020 Jan 1;25(3):241–53.

77. Crenshaw K. Mapping the Margins: Intersectionality, Identity Politics, and Violence against Women of Color. Stanford Law Rev. 1990 1991;43:1241.

78. Jayaweera H. Access to healthcare for vulnerable migrant women in England: A human security approach. Curr Sociol Monogr. 2018;66(2):273–85.

79. Germain S, Yong A. COVID-19 Highlighting Inequalities in Access to Healthcare in England: A Case Study of Ethnic Minority and Migrant Women. Fem Leg Stud. 2020 Nov 1;28(3):301–10.

80. Grey T, Sewell H, Shapiro G, Ashraf F. Mental Health Inequalities Facing U.K. Minority Ethnic Populations. J Psychol Issues Organ Cult. 3(S1):146–57.

81. Evans K, Spiby H, Morrell JC. Non-pharmacological interventions to reduce the symptoms of mild to moderate anxiety in pregnant women. A systematic review and narrative synthesis of women's views on the acceptability of and satisfaction with interventions. Arch Womens Ment Health. 2020 Feb 1;23(1):11–28.

82. Kassam S. Understanding Experiences of Social Support as Coping Resources among Immigrant and Refugee Women with Postpartum Depression: An Integrative Literature Review. Issues Ment Health Nurs. 2019 Dec 2;40(12):999–1011.

83. Jones CCG, Jomeen J, Hayter M. The impact of peer support in the context of perinatal mental illness: A meta-ethnography. Midwifery. 2014;30(5):491–8.

84. Bailey S, West M. Naming the issue: chronic excessive workload in the NHS [Internet]. The King's Fund. 2021 [cited 2023 Mar 15]. Available from: https://www.kingsfund.org.uk/blog/2021/06/naming-issue-chronic-excessive-workload-nhs

85. Trindade L de L, Pires DEP de. Implications of primary health care models in workloads of health professionals. Texto Contexto - Enferm. 2013 Mar;22:36–42.

86. Zantinge EM, Verhaak PF, Kerssens JJ, Bensing JM. The workload of GPs: consultations of patients with psychological and somatic problems compared. Br J Gen Pract. 2005 Aug 1;55(517):609–14.

87. Barriers and facilitators to GP–patient communication about emotional concerns in UK primary care: a systematic review | Family Practice | Oxford Academic [Internet]. [cited 2023 Mar 15]. Available from: https://academic.oup.com/fampra/article/37/4/434/5714005?login=false

For peer review only

88. Knaak S, Mantler E, Szeto A. Mental illness-related stigma in healthcare: Barriers to access and care and evidence-based solutions. Healthc Manage Forum. 2017;30(2):111–6.

89. Royal College of Midwives. Specialist Mental Health Midwives: What they do and why they matter [Internet]. 2018. Available from: https://www.rcm.org.uk/media/2370/specialist-mental-health-midwives-what-they-do-and-why-they-matter.pdf

90. Coates D, Foureur M. The role and competence of midwives in supporting women with mental health concerns during the perinatal period: A scoping review. Health Soc Care Th Community. 2019;27(4):e389–405.

91. Crear-Perry J, Maybank A, Keeys M, Mitchell N, Godbolt D. Moving towards anti-racist praxis in medicine. The Lancet. 2020 Aug 15;396(10249):451–3.

92. Davis CE, Lamson AL, Black KZ. MedFTs' Role in the Recruitment and Retention of a Diverse Physician Population: A Conceptual Model -. Contemp Fam Ther. 2022;44:88–100.

93. Kurek K, Teevan BE, Zlateva I, Anderson DR. Patient-Provider Social Concordance and Health Outcomes in Patients with Type 2 Diabetes: a Retrospective Study from a Large Federally Qualified Health Center in Connecticut. J Racial Ethn Health Disparities. 2016;3(2):217–24.

94. Thornton RLJ, Powe NR, Roter D, Cooper LA. Patient–physician social concordance, medical visit communication and patients' perceptions of health care quality. Patient Educ Couns. 2011 Dec 1;85(3):e201–8.

95. Hughes TM, Merath K, Chen Q, Sun S, Palmer E, Idrees JJ, et al. Association of shared decision-making on patient-reported health outcomes and healthcare utilization. Am J Surg. 2018 Jul 1;216(1):7–12.

96. Mozafaripour S. Diversity in Healthcare and the Importance of Representation [Internet]. University of St. Augustine for Health Sciences. 2021 [cited 2023 Mar 15]. Available from: https://www.usa.edu/blog/diversity-in-healthcare/

97. Watters E. Crazy Like Us: The Globalization of the American Psyche. New York: Free Press; 2010.

98. Ventevogel P, Jordans M, Reis R, de Jong J. Madness or sadness? Local concepts of mental illness in four conflict-affected African communities. Confl Health. 2013 Feb 18;7(1):3.

99. Hom MA, Stanley IH, Joiner Jr TE. Evaluating factors and interventions that influence help-seeking and mental health service utilization among suicidal individuals: A review of the literature -. Clin Psychol Rev. 40:28–39.

100. Wenze SJ, Battle CL. Perinatal Mental Health Treatment Needs, Preferences, and Barriers in Parents of Multiples. J Psychiatr Pract. 2018 May;24(3):158.

For peer review only

For peer review only

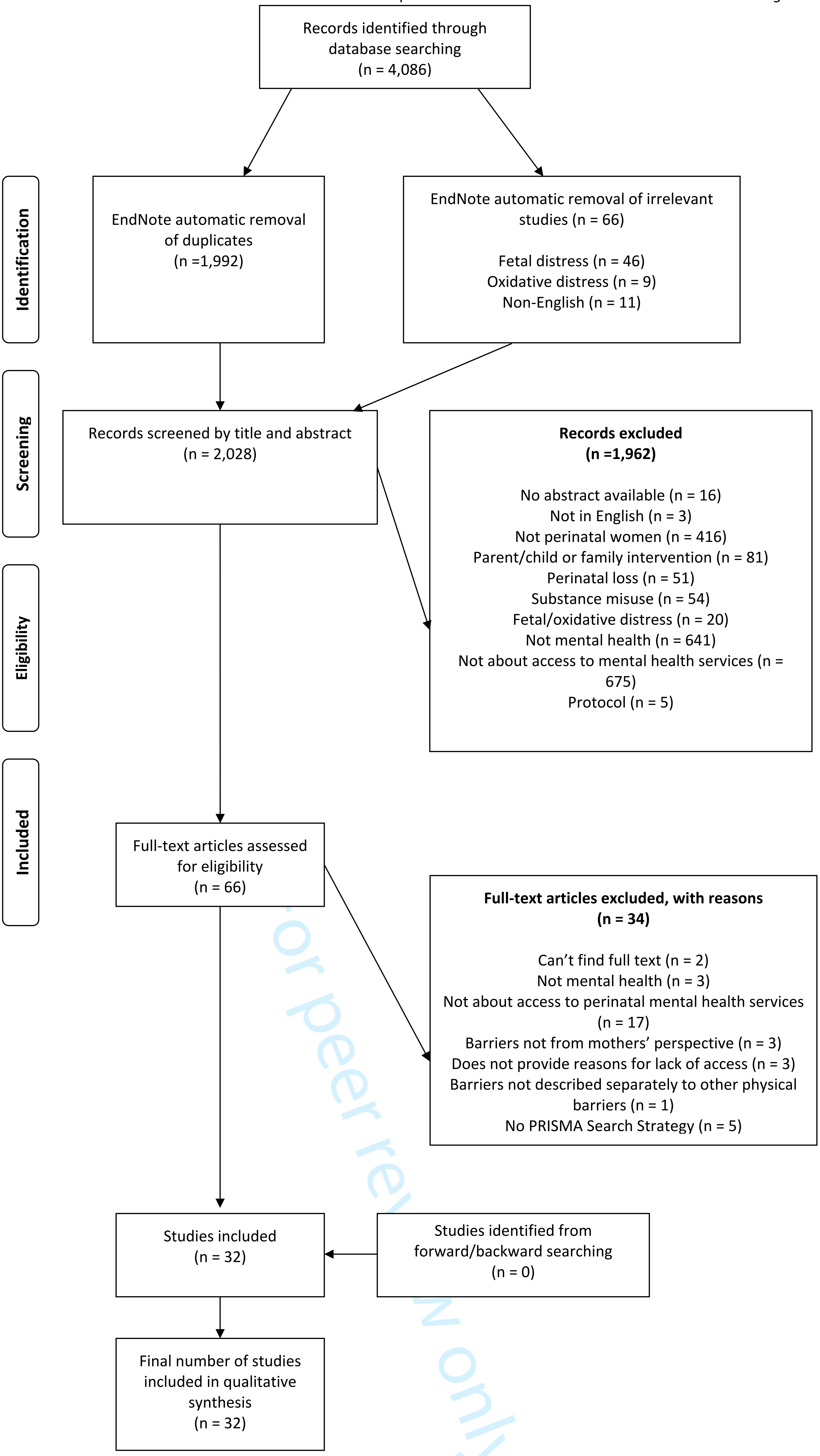

From: Moher D, Liberati A, Tetzlaff J, Altman DG, The PRISMA Group (2009). *P*referred *R*eporting *I*tems for *S*ystematic Reviews and *M*eta-*A*nalyses: The PRISMA Statement. PLoS Med 6(7): e1000097. doi:10.1371/journal.pmed1000097

For more information, visit www.prisma-statement.org.

For peer review only - http://bmjopen.bmj.com/site/about/guidelines.xhtml

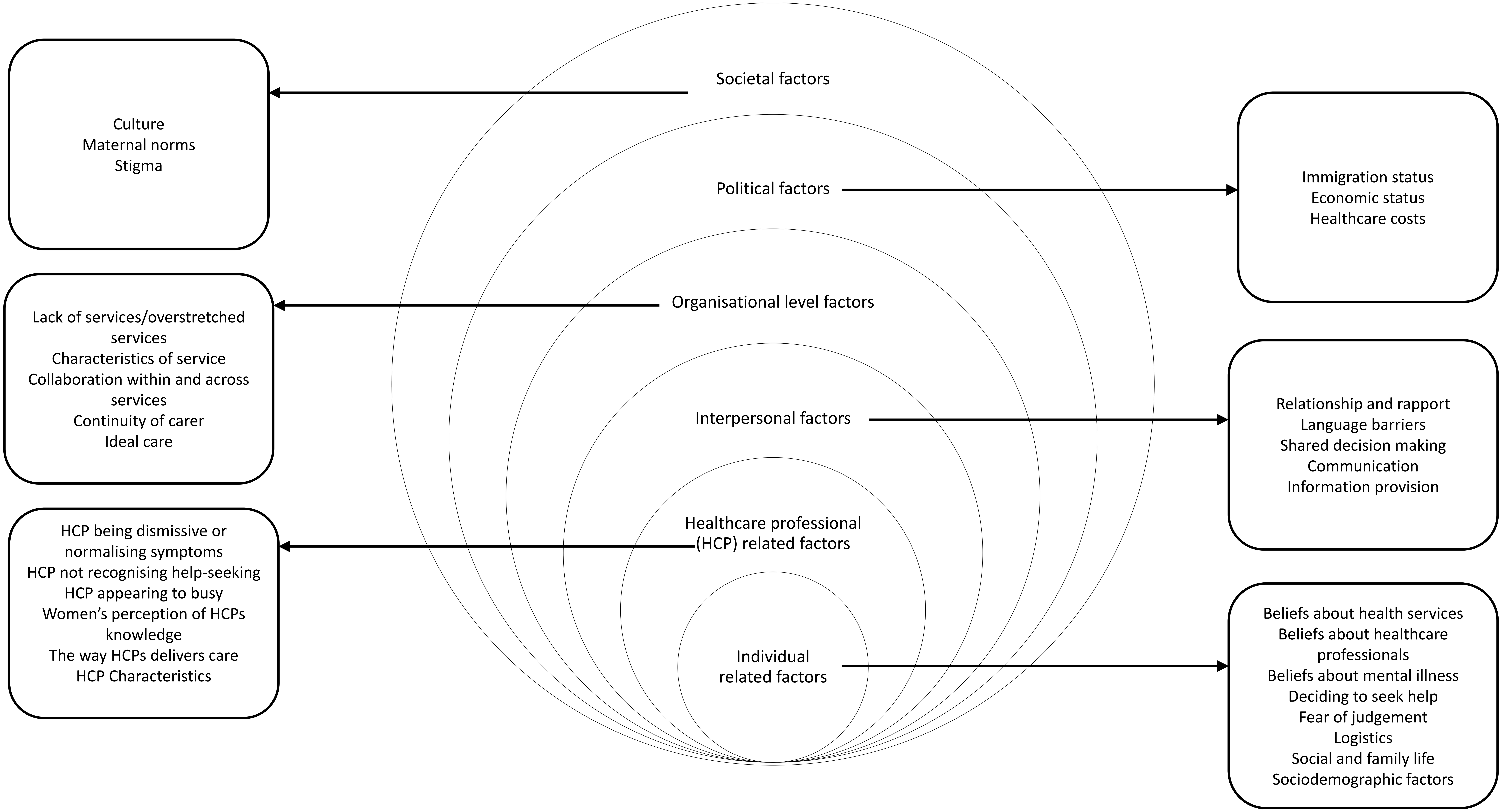
Societal factors
Political factors
Organisational level factors
Interpersonal factors
Healthcare professional (HCP) related factors
Individual related factors
Culture
Maternal norms
Stigma
Immigration status
Economic status
Healthcare costs
Lack of services/overstretched services
Characteristics of service
Collaboration within and across services
Continuity of carer
Ideal care
Relationship and rapport
Language barriers
Shared decision making
Communication
Information provision
HCP being dismissive or normalising symptoms
HCP not recognising help-seeking
HCP appearing to busy
Women's perception of HCPs knowledge
The way HCPs delivers care
HCP Characteristics
Beliefs about health services
Beliefs about healthcare professionals
Beliefs about mental illness
Deciding to seek help
Fear of judgement
Logistics
Social and family life
Sociodemographic factors

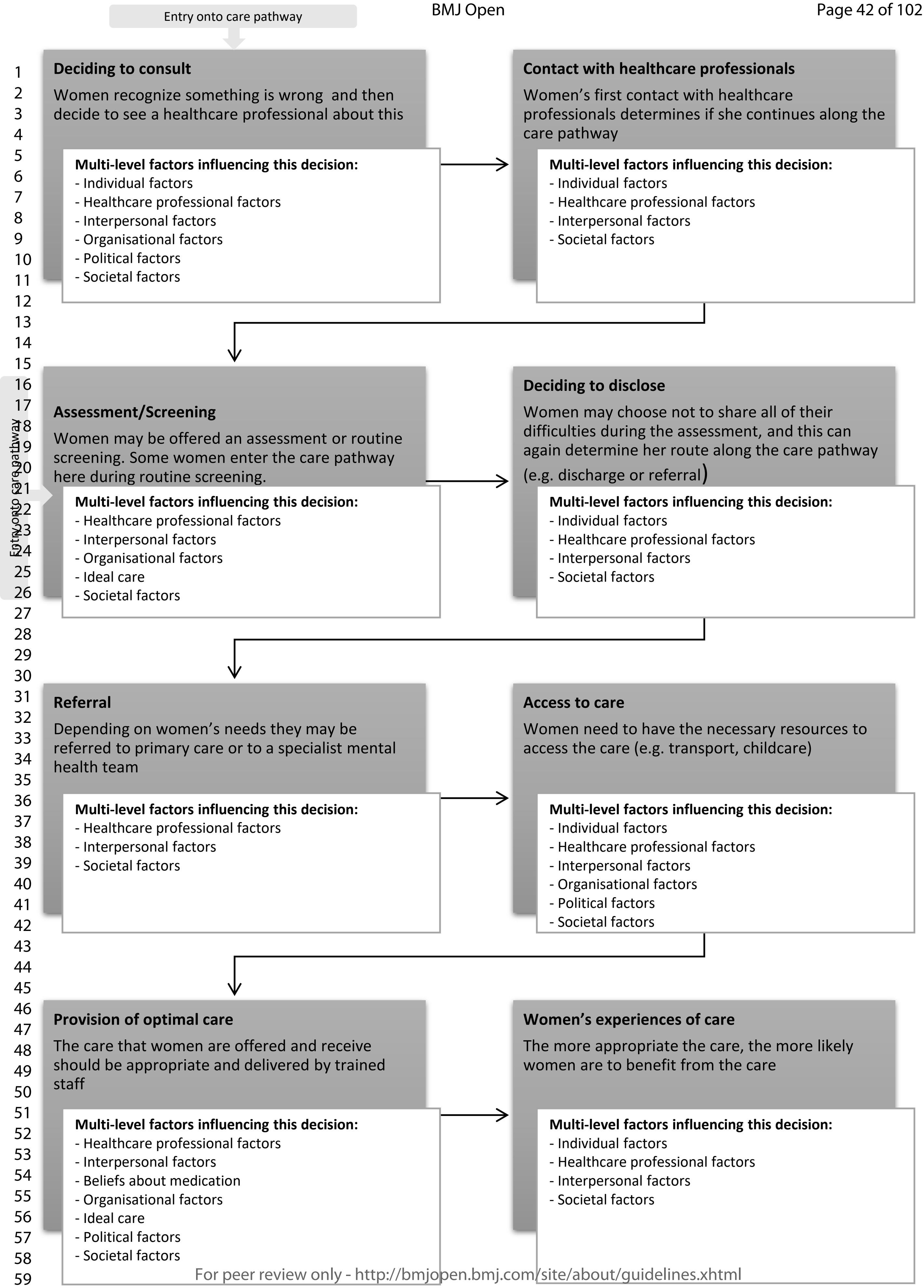

For peer review only - http://bmjopen.bmj.com/site/about/guidelines.xhtml

Note. Some parts of the pathway are redundant in health care systems where the woman can contact mental health services directly (e.g. France or via Improving Access to Psychological Therapies services in the UK). Further, the process is not always linear women might jump over certain stages.

A meta-review of the barriers to women accessing perinatal mental health care

Appendices

Contents

Appendix 1: PROSPERO Registration: CRD42019142854 2

Appendix 2: Table 1. Search terms 14

Appendix 3: Table 2. Search Results 28

Appendix 4: Table 3. Inclusion and exclusion criteria 29

Appendix 5: Detailed methodology 30

Appendix 6: Table 4. Sensitivity analysis 33

Appendix 7: *Figure 1.* Synthesis contribution vs quality appraisal criteria met 35

Appendix 8: Sensitivity analysis results 36

Appendix 9: Table 5. Summary review characteristics 37

Appendix 10: Table 6. Characteristics of included reviews 37

Appendix 11: Table 7. Quality appraisal 48

Appendix 12: Table 8. Themes 51

References 56

For peer review only

For peer review only - http://bmjopen.bmj.com/site/about/guidelines.xhtml

Appendix 1: PROSPERO Registration: CRD42019142854

PROSPERO
International prospective register of systematic reviews

National Institute for Health Research

UNIVERSITY of York
Centre for Reviews and Dissemination

Systematic review

1. * Review title.

Give the title of the review in English

Meta-review of barriers to women accessing perinatal mental healthcare and treatment

2. Original language title.

For reviews in languages other than English, give the title in the original language. This will be displayed with the English language title.

3. * Anticipated or actual start date.

Give the date the systematic review started or is expected to start.

07/06/2020

4. * Anticipated completion date.

Give the date by which the review is expected to be completed.

28/02/2021

5. * Stage of review at time of this submission.

Tick the boxes to show which review tasks have been started and which have been completed. Update this field each time any amendments are made to a published record.

Reviews that have started data extraction (at the time of initial submission) are not eligible for inclusion in PROSPERO. If there is later evidence that incorrect status and/or completion date has been supplied, the published PROSPERO record will be marked as retracted.

This field uses answers to initial screening questions. It cannot be edited until after registration.

The review has not yet started: No

Review stage	Started	Completed
Preliminary searches	Yes	No
Piloting of the study selection process	Yes	No
Formal screening of search results against eligibility criteria	No	No
Data extraction	No	No
Risk of bias (quality) assessment	No	No
Data analysis	No	No

Provide any other relevant information about the stage of the review here.

6. * Named contact.

The named contact is the guarantor for the accuracy of the information in the register record. This may be any member of the review team.

Rebecca Webb

Email salutation (e.g. "Dr Smith" or "Joanne") for correspondence:

Dr Webb

7. * Named contact email.

Give the electronic email address of the named contact.

Rebecca.Webb.2@city.ac.uk

8. Named contact address

Give the full institutional/organisational postal address for the named contact.

Centre for Maternal and Child Health Research, School of Health Sciences, City, University of London, EC1V 0HB

9. Named contact phone number.

Give the telephone number for the named contact, including international dialling code.

(+44)07810255328

10. * Organisational affiliation of the review.

Full title of the organisational affiliations for this review and website address if available. This field may be completed as 'None' if the review is not affiliated to any organisation.

City, University of London

Organisation web address:

11. * Review team members and their organisational affiliations.

Give the personal details and the organisational affiliations of each member of the review team. Affiliation refers to groups or organisations to which review team members belong. **NOTE: email and country now MUST be entered for each person, unless you are amending a published record.**

Dr Rebecca Webb. City, University of London
Dr Elizabeth Ford. Brighton and Sussex Medical School
Dr Judy Shakespeare. Retired GP
Dr Abigail Easter. King's College London
Professor Simon Gilbody. University of York
Professor Fiona Alderdice. University of Oxford
Dr Nia Roberts. Nuffield Department of Population Health, Bodleian Health Care Libraries
Professor Debra Salmon. City, University of London

Professor Helen Cheyne. University of Stirling
Ms Clare Thompson. Maternal Mental Health Change Agents
Miss Nazihah Uddin. City, University of London
Professor Susan Ayers. City, University of London

12. * Funding sources/sponsors.

Details of the individuals, organizations, groups, companies or other legal entities who have funded or sponsored the review.

NIHR Health Services and Delivery Research Grant

Grant number(s)

State the funder, grant or award number and the date of award

NIHR128068

13. * Conflicts of interest.

List actual or perceived conflicts of interest (financial or academic).

None

14. Collaborators.

Give the name and affiliation of any individuals or organisations who are working on the review but who are not listed as review team members. **NOTE: email and country must be completed for each person, unless you are amending a published record.**

Dr Agnes Hann. National Childbirth Trust
Dr Sarah McMullen. National Childbirth Trust
Dr Rose Coates. City, University of London
Dr Camilla Rosan. Anna Freud National Centre for Children and Families
Dr Sally Hogg. Parent-Infant Partnership UK
Mr Andrea Sinesi. University of Stirling

15. * Review question.

State the review question(s) clearly and precisely. It may be appropriate to break very broad questions down into a series of related more specific questions. Questions may be framed or refined using PI(E)COS or similar where relevant.

What are the individual, interpersonal, organizational, political and social factors that prevent women with perinatal mental health problems accessing care or treatment from the NHS or other health and social care services? A review of reviews.

Objectives are to:

1. Determine the barriers and facilitators to women accessing perinatal mental health care or treatment.

2. Identify differences in barriers and facilitators across different health and social care settings.

3. Evaluate the quality of published reviews.

4. Map the geographical distribution of the evidence to establish generalisability and gaps in the evidence.

16. * Searches.

State the sources that will be searched (e.g. Medline). Give the search dates, and any restrictions (e.g. language or publication date). Do NOT enter the full search strategy (it may be provided as a link or attachment below.)

PROSPERO
International prospective register of systematic reviews

National Institute for Health Research

Systematic searches will be conducted through online databases such as: MEDLINE; PsycINFO; PubMed; Cumulative Index to Nursing and Allied Health Literature (CINAHL); EMBASE; Cochrane Library; Web of Science and Scopus. The Turning Research Into Practice (TRIP) database will also be searched. Other search strategies will include: tracking citations of key papers (forward searching); examining reference lists of key papers (backwards searching). When conducting the searches search terms will be combined using Boolean terms "OR" and "AND".

To identify papers, the following parameters will be used:

• Population: Women in the perinatal period (conception to 1 year postpartum). (Search terms will include, but are not limited to: Perinatal OR postnatal OR postpartum OR antenatal).

• Intervention: Assessment, care or treatment for perinatal mental health (search terms will include: help-seeking OR Screening OR assessment OR service* AND Access* OR implement* OR seeking OR decision OR employ OR treatment seeking OR treatment engagement)

• Outcome: Barriers and facilitators, women's experiences, health and social care professionals' experiences (search terms will include: Barriers OR drawbacks OR obstacles OR issues)

Study design: Review papers where a clear systematic search strategy is used and reported (search terms will include: meta-synthesis* OR meta-ethnograph* OR meta-study)

Papers will be selected in two stages according to PRISMA guidelines. In the first stage, titles and abstracts will be screened by one researcher for relevance to the topic. Papers that are clearly not relevant will be excluded. A random selection will be screened by a second researcher and agreement between the two will be calculated using Cohen's Kappa statistic. In the second stage, full texts for papers that appear to be relevant will be obtained and final selection made by the researcher and project management group. Again, a random selection will be screened by a second researcher and agreement between the two will be calculated.

17. URL to search strategy.

Upload a file with your search strategy, or an example of a search strategy for a specific database, (including the keywords) in pdf or word format. In doing so you are consenting to the file being made publicly accessible. Or provide a URL or link to the strategy. Do NOT provide links to your search **results**.

Alternatively, upload your search strategy to CRD in pdf format. Please note that by doing so you are consenting to the file being made publicly accessible.

Do not make this file publicly available until the review is complete

18. * Condition or domain being studied.

Give a short description of the disease, condition or healthcare domain being studied in your systematic review.

Mental health problems affect up to one in five women during pregnancy and the first year after birth (the perinatal period). These include anxiety, depression, stress-related conditions and adjustment disorders. This costs the UK £8.1 billion for every year of babies born. Mental health problems can have a negative effect on women, their partners and their children. They are also one of the leading causes of maternal death. It is vitally important that women who experience mental health difficulties are identified quickly and get the treatment they need. However, only about half of women with perinatal mental health problems are identified by healthcare services and even fewer receive treatment. We will therefore include the following perinatal mental health problems in our review: depression, anxiety, adjustment disorders, OCD, PTSD, psychosis, eating disorders, personality disorders.

19. * Participants/population.

Specify the participants or populations being studied in the review. The preferred format includes details of both inclusion and exclusion criteria.

The population of interest is women in the perinatal period, who are at risk of, or who are experiencing perinatal mental health problems (including, but not limited to, anxiety, depression, PTSD, adjustment disorders). No restrictions will be placed on women's age or ethnicity. If papers include multiple stakeholder views, we will include reviews where the women's responses can be separated out.

Papers will be excluded if they: are non-English publications; are text or opinion pieces, do not have a PRISMA guided search strategy, include people outside the target population (e.g. men/partners or children) where their views cannot be separated from women's views; include interventions targeted at the parent-infant, couple or family relationships; focus on perinatal loss due to the unique focus of the treatment, or focus on substance misuse which has unique challenges in terms of assessment and treatment, or focus on oxidative stress or fetal distress.

20. * Intervention(s), exposure(s).

Give full and clear descriptions or definitions of the interventions or the exposures to be reviewed. The preferred format includes details of both inclusion and exclusion criteria.

We are interested in reviews that look at women's views and experiences of accessing perinatal mental health care across the treatment pathway from deciding to consult or help seeking and disclosing symptoms to assessment, referral, care and treatment.

21. * Comparator(s)/control.

Where relevant, give details of the alternatives against which the intervention/exposure will be compared (e.g. another intervention or a non-exposed control group). The preferred format includes details of both inclusion and exclusion criteria.

Not applicable

22. * Types of study to be included.

Give details of the study designs (e.g. RCT) that are eligible for inclusion in the review. The preferred format

For peer review only - http://bmjopen.bmj.com/site/about/guidelines.xhtml

includes both inclusion and exclusion criteria. If there are no restrictions on the types of study, this should be stated.

Review papers that have used a search strategy according the PRISMA guidelines, such as systematic reviews, about access to mental health care or treatment by women in the perinatal period across all stages of the care pathway (deciding to consult, contact with healthcare professionals, assessment/screening, deciding to disclose, referral, access to treatment and provision of optimal treatment). Assessment refers to identifying women who may be at risk for perinatal mental health problems, or who have perinatal mental health problems. Care refers to supportive care or care pathways such as that provided by health professionals who provide care to women with perinatal mental health difficulties across the care pathway. Treatment refers to any active intervention, programmes or protocols to reduce women's perinatal mental health symptoms. The perinatal period is defined as from conception to 1 year postpartum. Mental health measures need to be gold standard clinical interviews or validated self-report questionnaires.

23. Context.

Give summary details of the setting or other relevant characteristics, which help define the inclusion or exclusion criteria.

As women move through pregnancy and the postnatal period, they come into contact with different services that provide women with opportunities to disclose any mental health difficulties and access perinatal mental health care. Despite the services available for women with perinatal mental health difficulties, it is estimated that half of women are not identified despite regular routine contact with these healthcare services, and still fewer receive treatment. For example, a study of postnatal depression suggested only 40% of women with postnatal depression were identified, 24% received treatment, 10% received adequate treatment, and only 3-6% of women recovered. This is likely to be due to a range of factors at individual, interpersonal, organisational and social levels, such as healthcare professionals not asking about mental health, lack of effective assessment, barriers to women seeking help or attending treatment, clinician barriers to diagnosis and treatment, lack of services to refer onto, or limited understanding of effective treatments.

Given that women accessing care or treatment might be important in preventing adverse outcomes for women and their families, we need to identify the wide range of factors that may prevent women from accessing these. The literature on why women with perinatal mental health problems do not access care or treatment is varied and some areas are more clearly synthesised than others. At present, there is no clear overview and synthesis of how these factors may operate at different levels, which is why a systematic review of reviews is needed.

24. * Main outcome(s).

Give the pre-specified main (most important) outcomes of the review, including details of how the outcome is defined and measured and when these measurement are made, if these are part of the review inclusion criteria.

PROSPERO
International prospective register of systematic reviews

National Institute for Health Research

The main aim is to determine the individual, interpersonal, organisational and social factors that prevent women accessing care or treatment. Therefore, the main outcome will be evaluation parameters for assessment, care or treatment for perinatal mental health, including barriers, facilitators, women's experiences, and health and social care professionals' experiences.

Measures of effect

Please specify the effect measure(s) for you main outcome(s) e.g. relative risks, odds ratios, risk difference, and/or 'number needed to treat.

Not applicable.

25. * Additional outcome(s).

List the pre-specified additional outcomes of the review, with a similar level of detail to that required for main outcomes. Where there are no additional outcomes please state 'None' or 'Not applicable' as appropriate to the review

None.

Measures of effect

Please specify the effect measure(s) for you additional outcome(s) e.g. relative risks, odds ratios, risk difference, and/or 'number needed to treat.

Not applicable.

26. * Data extraction (selection and coding).

Describe how studies will be selected for inclusion. State what data will be extracted or obtained. State how this will be done and recorded.

Data will be extracted from eligible reviews using a standard data extraction form in Excel or using the data extraction tool on EPPI-Reviewer. If information is missing, corresponding authors will be contacted with a request for the information. If they do not respond within 2 weeks they will be contacted again. If they do not respond within 4 weeks, missing data will not be included.

A range of data will be extracted to enable a comprehensive meta-review. Key variables will include: Authors; Year; Country; Review design; Method; Sample size and characteristics (of parents, of health care provider); Mental health problem; Outcome measures; Type of intervention(s); Methodological quality rating.

27. * Risk of bias (quality) assessment.

State which characteristics of the studies will be assessed and/or any formal risk of bias/quality assessment tools that will be used.

Quality will be assessed using the AMSTAR checklist which is a reliable and valid measure for assessing the methodological quality of systematic reviews. One reviewer will conduct the quality assessments and reliability of these ratings will be checked="checked" value="1" by a second reviewer rating a random selection of 25% of papers. If disagreements arise then all papers will be double-rated, and disagreements resolved through discussion and consensus. Where consensus cannot be reached the project management group will be consulted and make the final decision. If agreement is low, the second rater will look at a larger

group of papers. If meta-analyses are identified, we will assess the quality of the analysis using the Scottish Intercollegiate Guidelines Network (SIGN) evidence grading system. This system grades the risk of bias associated with a particular piece of evidence on a hierarchy from meta-analysis and RCT evidence (grade 1) down to expert opinion (grade 4), with additional indicators (++, + or -) to indicate methodological quality.

28. * Strategy for data synthesis.

Describe the methods you plan to use to synthesise data. This **must not be generic text** but should be **specific to your review** and describe how the proposed approach will be applied to your data. If meta-analysis is planned, describe the models to be used, methods to explore statistical heterogeneity, and software package to be used.

We will follow a similar strategy to McNeill et al (2012). Firstly, a table summarizing the findings will be presented. Within the table we will include the following information: authors/year; aim; search strategy; number of studies included; total number of participants; results (i.e. key barriers and facilitators identified); quality rating. We will then perform a narrative synthesis of reviews identified, discussing both barriers and facilitators to women accessing perinatal mental health assessment, care and treatment across the care pathway. Where quantitative data is included, we will carry out a narrative synthesis where we indicate both the quality of the evidence (low, medium, high) and whether it is causal or associative in nature as done by Greaves et al., (2011).

Greaves, C.J., Sheppard, K.E., Abraham, C. et al. Systematic review of reviews of intervention components associated with increased effectiveness in dietary and physical activity interventions. BMC Public Health 11, 119 (2011). https://doi.org/10.1186/1471-2458-11-119

McNeill, J., Lynn, F. & Alderdice, F. Public health interventions in midwifery: a systematic review of systematic reviews. BMC Public Health 12, 955 (2012). https://doi.org/10.1186/1471-2458-12-955

29. * Analysis of subgroups or subsets.

State any planned investigation of 'subgroups'. Be clear and specific about which type of study or participant will be included in each group or covariate investigated. State the planned analytic approach.

We will examine recommendations for healthcare practice and research separately, including which countries have a sparsity of data and recommendations for quality improvement in research methods.

30. * Type and method of review.

Select the type of review, review method and health area from the lists below.

Type of review

Cost effectiveness
No

Diagnostic
No

Epidemiologic
No

For peer review only - http://bmjopen.bmj.com/site/about/guidelines.xhtml

PROSPERO
International prospective register of systematic reviews

Individual patient data (IPD) meta-analysis
No

Intervention
No

Living systematic review
No

Meta-analysis
No

Methodology
No

Narrative synthesis
Yes

Network meta-analysis
No

Pre-clinical
No

Prevention
No

Prognostic
No

Prospective meta-analysis (PMA)
No

Review of reviews
Yes

Service delivery
No

Synthesis of qualitative studies
Yes

Systematic review
Yes

Other
No

Health area of the review

Alcohol/substance misuse/abuse
No

Blood and immune system
No

Cancer
No

Cardiovascular
No

Care of the elderly

For peer review only - http://bmjopen.bmj.com/site/about/guidelines.xhtml

PROSPERO
International prospective register of systematic reviews

National Institute for Health Research

No

Child health
No

Complementary therapies
No

COVID-19
No

Crime and justice
No

Dental
No

Digestive system
No

Ear, nose and throat
No

Education
No

Endocrine and metabolic disorders
No

Eye disorders
No

General interest
No

Genetics
No

Health inequalities/health equity
No

Infections and infestations
No

International development
No

Mental health and behavioural conditions
Yes

Musculoskeletal
No

Neurological
No

Nursing
No

Obstetrics and gynaecology
No

Oral health
No

Palliative care
No

Perioperative care
No

Physiotherapy
No

Pregnancy and childbirth
Yes

Public health (including social determinants of health)
No

Rehabilitation
No

Respiratory disorders
No

Service delivery
No

Skin disorders
No

Social care
No

Surgery
No

Tropical Medicine
No

Urological
No

Wounds, injuries and accidents
No

Violence and abuse
No

31. Language.

Select each language individually to add it to the list below, use the bin icon to remove any added in error.
English

There is not an English language summary

32. * Country.

Select the country in which the review is being carried out. For multi-national collaborations select all the countries involved.

England

33. Other registration details.

Name any other organisation where the systematic review title or protocol is registered (e.g. Campbell, or

The Joanna Briggs Institute) together with any unique identification number assigned by them. If extracted data will be stored and made available through a repository such as the Systematic Review Data Repository (SRDR), details and a link should be included here. If none, leave blank.

34. Reference and/or URL for published protocol.

If the protocol for this review is published provide details (authors, title and journal details, preferably in Vancouver format)

Add web link to the published protocol.

Or, upload your published protocol here in pdf format. Note that the upload will be publicly accessible.

No I do not make this file publicly available until the review is complete

Please note that the information required in the PROSPERO registration form must be completed in full even if access to a protocol is given.

35. Dissemination plans.

Do you intend to publish the review on completion?

Yes

Give brief details of plans for communicating review findings.?

We plan to publish this review in a high impact peer reviewed journal.

36. Keywords.

Give words or phrases that best describe the review. Separate keywords with a semicolon or new line. Keywords help PROSPERO users find your review (keywords do not appear in the public record but are included in searches). Be as specific and precise as possible. Avoid acronyms and abbreviations unless these are in wide use.

Perinatal mental health; Barriers; Access; Healthcare; Treatment

37. Details of any existing review of the same topic by the same authors.

If you are registering an update of an existing review give details of the earlier versions and include a full bibliographic reference, if available.

38. * Current review status.

Update review status when the review is completed and when it is published.New registrations must be ongoing so this field is not editable for initial submission.
Please provide anticipated publication date

Review_Ongoing

39. Any additional information.

Provide any other information relevant to the registration of this review.

40. Details of final report/publication(s) or preprints if available.

Leave empty until publication details are available OR you have a link to a preprint (NOTE: this field is not editable for initial submission). List authors, title and journal details preferably in Vancouver format.

Give the link to the published review or preprint.

Appendix 2: Table 1. Search terms

# ▲	Searches
	**MEDLINE (1946-present)**
1	prenatal care/ or perinatal care/ or postnatal care/
2	Pregnancy/
3	Pregnant Women/
4	(pregnancy or pregnant or pre-nat* or prenat* or prepart* or prepart* or ante-nat* or antenat* or ante-part* or antepart* or peri-nat* or perinat* or peri-part* or peripart* or puerper* or post-nat* or postnat* or post-part* or postpart*).ti.
5	((parent? or mother* or maternal or father* or paternal) and (infan* or newborn? or neonat* or baby or babies)).ti.
6	1 or 2 or 3 or 4 or 5
7	mental disorders/ or exp anxiety disorders/ or exp mood disorders/ or exp "trauma and stressor related disorders"/
8	Stress, Psychological/
9	Adaptation, Psychological/
10	(mental* or psych* or anxiety or anxious or depress* or mood? or affect* or distress* or stress or trauma* or posttrauma* or post-trauma* or adjustment disorder* or phobia* or phobic or obsessive compulsive or wellbeing or well-being).ti.
11	7 or 8 or 9 or 10
12	6 and 11
13	Depression, Postpartum/
14	Pregnant Women/px [Psychology]
15	((pregnancy or pregnant or pre-nat* or prenat* or prepart* or prepart* or ante-nat* or antenat* or ante-part* or antepart* or peri-nat* or perinat* or peri-part* or peripart* or puerper* or post-nat* or postnat* or post-part* or postpart*) adj5 (mental* or psych* or anxiety or anxious or depress* or mood? or affect* or distress* or stress or trauma* or posttrauma* or post-trauma* or adjustment disorder* or phobia* or phobic or obsessive compulsive or wellbeing or well-being)).ti,ab.
16	(((parent? or mother* or maternal or father* or paternal) adj5 (infan* or newborn? or neonat* or baby or babies)) and (mental* or psych* or anxiety or anxious or depress* or mood? or affect* or distress* or stress or trauma* or posttrauma* or post-trauma* or adjustment disorder* or phobia* or phobic or obsessive compulsive or wellbeing or well-being)).ti,ab.

For peer review only - http://bmjopen.bmj.com/site/about/guidelines.xhtml

17	12 or 13 or 14 or 15 or 16
18	Mass Screening/
19	diagnosis/ or early diagnosis/
20	(screen* or detect* or diagnos* or assess* or identifi* or prevent* or prophyla*).ti.
21	psychotherapy/ or behavior therapy/ or exp cognitive behavioral therapy/
22	counseling/ or exp directive counseling/
23	exp antidepressive agents/ or exp anti-anxiety agents/
24	(intervention? or counsel* or therap* or healing or listen* support* or care or healthcare or service or medication* or drug? or antidepress* or anti-depress* or antianxiety or anti-anxiety).ti.
25	("improving access to psychological therap*" or iapt).ti,ab.
26	((mental* or psych* or anxiety or anxious or depress* or mood? or affect* or distress* or stress or trauma* or posttrauma* or post-trauma* or adjustment disorder* or phobia* or phobic or obsessive compulsive or wellbeing or well-being) adj5 (screen* or detect* or diagnos* or assess* or identifi* or prevent* or prophyla*)).ti,ab.
27	((mental* or psych* or anxiety or anxious or depress* or mood? or affect* or distress* or stress or trauma* or posttrauma* or post-trauma* or adjustment disorder* or phobia* or phobic or obsessive compulsive or wellbeing or well-being) adj5 (intervention? or counsel* or therap* or healing or listen* support* or care or healthcare or service or medication* or drug? or antidepress* or anti-depress* or antianxiety or anti-anxiety)).ti,ab.
28	18 or 19 or 20 or 21 or 22 or 23 or 24 or 25 or 26 or 27
29	17 and 28
30	Depression, Postpartum/di, dh, dt, pc, th
31	29 or 30
32	Implementation Science/ or Health Plan Implementation/
33	Program Evaluation/
34	(implement* or impact*).ti,ab.
35	(feasib* or acceptab*).ti,ab.
36	(barrier? or challenge? or obstacle? or hurdle? or obstruct* or drawback? or issue? or difficult? or promot* or support or encourag* or factor? or facilitat* or enabl* or opportunit* or engage* or assist*).ti,ab.
37	((process or project* or system*) adj5 evaluat*).ti,ab.
38	32 or 33 or 34 or 35 or 36 or 37

For peer review only - http://bmjopen.bmj.com/site/about/guidelines.xhtml

39	31 and 38
40	medline.ti,ab.
41	systematic review.pt.
42	meta-analysis.pt.
43	systematic review.ti,ab.
44	(evidence synthesis or realist synthesis or realist review).ti,ab.
45	(Qualitative and synthesis).ti,ab.
46	(meta-synthesis* or meta synthesis* or metasynthesis).ti,ab.
47	(meta-ethnograph* or metaethnograph* or meta ethnograph*).ti,ab.
48	(meta-study or metastudy or meta study).ti,ab.
49	40 or 41 or 42 or 43 or 44 or 45 or 46 or 47 or 48
50	39 and 49
51	(comment or editorial or letter or historical article).pt.
52	50 not 51
53	exp animals/ not humans/
54	52 not 53
55	limit 54 to english language
	**EMBASE (1974 – present)**
1	prenatal care/ or newborn period/ or perinatal period/ or prenatal period/
2	*Pregnancy/
3	(pregnancy or pregnant or pre-nat* or prenat* or prepart* or prepart* or ante-nat* or antenat* or ante-part* or antepart* or peri-nat* or perinat* or peri-part* or peripart* or puerper* or post-nat* or postnat* or post-part* or postpart*).ti.
4	((parent? or mother* or maternal or father* or paternal) and (infan* or newborn? or neonat* or baby or babies)).ti.
5	1 or 2 or 3 or 4
6	mental disease/ or exp anxiety disorder/ or exp mood disorder/
7	mental stress/
8	(mental* or psych* or anxiety or anxious or depress* or mood? or affect* or distress* or stress or trauma* or posttrauma* or post-trauma* or adjustment disorder* or phobia* or phobic or obsessive compulsive or wellbeing or well-being).ti.
9	6 or 7 or 8

For peer review only - http://bmjopen.bmj.com/site/about/guidelines.xhtml

10	5 and 9
11	exp perinatal depression/
12	((pregnancy or pregnant or pre-nat* or prenat* or prepart* or prepart* or ante-nat* or antenat* or ante-part* or antepart* or peri-nat* or perinat* or peri-part* or peripart* or puerper* or post-nat* or postnat* or post-part* or postpart*) adj5 (mental* or psych* or anxiety or anxious or depress* or mood? or affect* or distress* or stress or trauma* or posttrauma* or post-trauma* or adjustment disorder* or phobia* or phobic or obsessive compulsive or wellbeing or well-being)).ti,ab.
13	(((parent? or mother* or maternal or father* or paternal) adj5 (infan* or newborn? or neonat* or baby or babies)) and (mental* or psych* or anxiety or anxious or depress* or mood? or affect* or distress* or stress or trauma* or posttrauma* or post-trauma* or adjustment disorder* or phobia* or phobic or obsessive compulsive or wellbeing or well-being)).ti,ab.
14	10 or 11 or 12 or 13
15	mass screening/ or screening test/ or screening/
16	diagnosis/ or early diagnosis/
17	(screen* or detect* or diagnos* or assess* or identifi* or prevent* or prophyla*).ti.
18	exp counseling/ or early intervention/ or exp psychotherapy/
19	(intervention? or counsel* or therap* or healing or listen* support* or care or healthcare or service or medication* or drug? or antidepress* or anti-depress* or antianxiety or anti-anxiety).ti.
20	("improving access to psychological therap*" or iapt).ti,ab.
21	((mental* or psych* or anxiety or anxious or depress* or mood? or affect* or distress* or stress or trauma* or posttrauma* or post-trauma* or adjustment disorder* or phobia* or phobic or obsessive compulsive or wellbeing or well-being) adj5 (screen* or detect* or diagnos* or assess* or identifi* or prevent* or prophyla*)).ti,ab.
22	((mental* or psych* or anxiety or anxious or depress* or mood? or affect* or distress* or stress or trauma* or posttrauma* or post-trauma* or adjustment disorder* or phobia* or phobic or obsessive compulsive or wellbeing or well-being) adj5 (intervention? or counsel* or therap* or healing or listen* support* or care or healthcare or service or medication* or drug? or antidepress* or anti-depress* or antianxiety or anti-anxiety)).ti,ab.
23	15 or 16 or 17 or 18 or 19 or 20 or 21 or 22
24	14 and 23
25	exp perinatal depression/di, dt, pc, th
26	24 or 25

For peer review only

For peer review only - http://bmjopen.bmj.com/site/about/guidelines.xhtml

27	therapy delay/
28	exp Program Evaluation/ or Implementation Science/
29	(implement* or impact*).ti,ab.
30	(feasib* or acceptab*).ti,ab.
31	(barrier? or challenge? or obstacle? or hurdle? or obstruct* or drawback? or issue? or difficult? or promot* or support or encourag* or factor? or facilitat* or enabl* or opportunit* or engage* or assist*).ti,ab.
32	((process or project* or system*) adj5 evaluat*).ti,ab.
33	27 or 28 or 29 or 30 or 31 or 32
34	26 and 33
35	medline.tw.
36	"systematic review"/
37	meta-analysis/
38	systematic review.ti,ab.
39	(evidence synthesis or realist synthesis or realist review).ti,ab.
40	(Qualitative and synthesis).ti,ab.
41	(meta-synthesis* or meta synthesis* or metasynthesis).ti,ab.
42	(meta-ethnograph* or metaethnograph* or meta ethnograph*).ti,ab.
43	(meta-study or metastudy or meta study).ti,ab.
44	35 or 36 or 37 or 38 or 39 or 40 or 41 or 42 or 43
45	34 and 44
46	(editorial or letter or note or conference*).pt.
47	45 not 46
48	(exp animals/ or nonhuman/) not human/
49	47 not 48
50	limit 49 to english language
	**PSYCHINFO (1806 – present)**
1	prenatal care/ or postnatal period/ or antepartum period/ or intrapartum period/ or perinatal period/
2	Pregnancy/

For peer review only

3	(pregnancy or pregnant or pre-nat* or prenat* or prepart* or prepart* or ante-nat* or antenat* or ante-part* or antepart* or peri-nat* or perinat* or peri-part* or peripart* or puerper* or post-nat* or postnat* or post-part* or postpart*).ti.
4	((parent? or mother* or maternal or father* or paternal) and (infan* or newborn? or neonat* or baby or babies)).ti.
5	1 or 2 or 3 or 4
6	mental disorders/ or exp affective disorders/ or exp anxiety disorders/ or exp "stress and trauma related disorders"/
7	psychological stress/
8	Emotional Adjustment/
9	(mental* or psych* or anxiety or anxious or depress* or mood? or affect* or distress* or stress or trauma* or posttrauma* or post-trauma* or adjustment disorder* or phobia* or phobic or obsessive compulsive or wellbeing or well-being).ti.
10	6 or 7 or 8 or 9
11	5 and 10
12	postpartum depression/ or postpartum psychosis/
13	((pregnancy or pregnant or pre-nat* or prenat* or prepart* or prepart* or ante-nat* or antenat* or ante-part* or antepart* or peri-nat* or perinat* or peri-part* or peripart* or puerper* or post-nat* or postnat* or post-part* or postpart*) adj5 (mental* or psych* or anxiety or anxious or depress* or mood? or affect* or distress* or stress or trauma* or posttrauma* or post-trauma* or adjustment disorder* or phobia* or phobic or obsessive compulsive or wellbeing or well-being)).ti,ab.
14	(((parent? or mother* or maternal or father* or paternal) adj5 (infan* or newborn? or neonat* or baby or babies)) and (mental* or psych* or anxiety or anxious or depress* or mood? or affect* or distress* or stress or trauma* or posttrauma* or post-trauma* or adjustment disorder* or phobia* or phobic or obsessive compulsive or wellbeing or well-being)).ti,ab.
15	11 or 12 or 13 or 14
16	screening/ or exp health screening/ or exp screening tests/
17	diagnosis/
18	(screen* or detect* or diagnos* or assess* or identifi* or prevent* or prophyla*).ti.
19	treatment/ or exp cognitive behavior therapy/ or exp cognitive techniques/ or exp counseling/ or mindfulness-based interventions/ or exp psychotherapy/
20	(intervention? or counsel* or therap* or healing or listen* support* or care or healthcare or service or medication* or drug? or antidepress* or anti-depress* or antianxiety or anti-anxiety).ti.
21	("improving access to psychological therap*" or iapt).ti,ab.

For peer review only - http://bmjopen.bmj.com/site/about/guidelines.xhtml

22	((mental* or psych* or anxiety or anxious or depress* or mood? or affect* or distress* or stress or trauma* or posttrauma* or post-trauma* or adjustment disorder* or phobia* or phobic or obsessive compulsive or wellbeing or well-being) adj5 (screen* or detect* or diagnos* or assess* or identifi* or prevent* or prophyla*)).ti,ab.
23	((mental* or psych* or anxiety or anxious or depress* or mood? or affect* or distress* or stress or trauma* or posttrauma* or post-trauma* or adjustment disorder* or phobia* or phobic or obsessive compulsive or wellbeing or well-being) adj5 (intervention? or counsel* or therap* or healing or listen* support* or care or healthcare or service or medication* or drug? or antidepress* or anti-depress* or antianxiety or anti-anxiety)).ti,ab.
24	16 or 17 or 18 or 19 or 20 or 21 or 22 or 23
25	15 and 24
26	treatment barriers/
27	exp Program Evaluation/
28	(implement* or impact*).ti,ab.
29	(feasib* or acceptab*).ti,ab.
30	(barrier? or challenge? or obstacle? or hurdle? or obstruct* or drawback? or issue? or difficult? or promot* or support or encourag* or factor? or facilitat* or enabl* or opportunit* or engage* or assist*).ti,ab.
31	((process or project* or system*) adj5 evaluat*).ti,ab.
32	26 or 27 or 28 or 29 or 30 or 31
33	25 and 32
34	medline.ti,ab.
35	exp "Systematic Review"/
36	Meta Analysis/
37	systematic review.ti,ab.
38	(evidence synthesis or realist synthesis or realist review).ti,ab.
39	(Qualitative and synthesis).ti,ab.
40	(meta-synthesis* or meta synthesis* or metasynthesis).ti,ab.
41	(meta-ethnograph* or metaethnograph* or meta ethnograph*).ti,ab.
42	(meta-study or metastudy or meta study).ti,ab.
43	34 or 35 or 36 or 37 or 38 or 39 or 40 or 41 or 42
44	33 and 43

For peer review only - http://bmjopen.bmj.com/site/about/guidelines.xhtml

For peer review only

45	(comment reply or editorial or letter or "review book" or "review media" or "review software other").dt.
46	44 not 45
47	limit 46 to english language
	**CINAHL (1982 – present)**
S30	S28 NOT S29
S29	S23 AND S27 Limiters - English Language; Publication Type: Book Review, Commentary, Editorial, Letter
S28	S23 AND S27
S27	S24 OR S25 OR S26
S26	TX ("evidence synthesis" or "realist synthesis" or "realist review") OR TX (Qualitative and synthesis) OR TX ((meta-synthesis* or "meta synthesis*" or metasynthesis) OR TX (meta-ethnograph* or metaethnograph* or "meta ethnograph*") OR TX (meta-study or metastudy or "meta study")
S25	TI (medline or "systematic review") OR AB (medline or "systematic review")
S24	(MH "Systematic Review") OR (MH "Meta Analysis") OR (MH "Meta Synthesis")
S23	S19 AND S22
S22	S20 OR S21
S21	((implement* or impact*)) OR ((implement* or impact*)) OR ((feasib* or acceptab*)) OR ((feasib* or acceptab*)) OR ((barrier? or challenge? or obstacle? or hurdle? or obstruct* or drawback? or issue? or difficult? or promot* or support or encourag* or factor? or facilitat* or enabl* or opportunit* or engage* or assist*)) OR ((barrier? or challenge? or obstacle? or hurdle? or obstruct* or drawback? or issue? or difficult? or promot* or support or encourag* or factor? or facilitat* or enabl* or opportunit* or engage* or assist*)) OR (((process or project* or system*) N5 evaluat*)) OR (((process or project* or system*) N5 evaluat*))
S20	(MH "Implementation Science") OR (MH "Program Development+")
S19	S17 OR S18
S18	(MH "Depression, Postpartum/DI/DH/DT/PC/TH") OR (MH "Postpartum Psychosis/DI/DH/DT/TH/PC")
S17	S11 AND S16
S16	S12 OR S13 OR S14 OR S15

For peer review only

S15	TI ((intervention? or counsel* or therap* or healing or listen* support* or care or healthcare or service or medication* or drug? or antidepress* or anti-depress* or antianxiety or anti-anxiety)) OR TI (("improving access to psychological therap*" or iapt)) OR AB (("improving access to psychological therap*" or iapt)) OR TI (((mental* or psych* or anxiety or anxious or depress* or mood? or affect* or distress* or stress or trauma* or posttrauma* or post-trauma* or adjustment disorder* or phobia* or phobic or obsessive compulsive or wellbeing or well-being) N5 (screen* or detect* or diagnos* or assess* or identifi* or prevent* or prophyla*))) OR AB (((mental* or psych* or anxiety or anxious or depress* or mood? or affect* or distress* or stress or trauma* or posttrauma* or post-trauma* or adjustment disorder* or phobia* or phobic or obsessive compulsive or wellbeing or well-being) N5 (screen* or detect* or diagnos* or assess* or identifi* or prevent* or prophyla*))) OR TI (((mental* or psych* or anxiety or anxious or depress* or mood? or affect* or distress* or stress or trauma* or posttrauma* or post-trauma* or adjustment disorder* or phobia* or phobic or obsessive compulsive or wellbeing or well-being) N5 (intervention? or counsel* or therap* or healing or listen* support* or care or healthcare or service or medication* or drug? or antidepress* or anti-depress* or antianxiety or anti-anxiety))) OR AB (((mental* or psych* or anxiety or anxious or depress* or mood? or affect* or distress* or stress or trauma* or posttrauma* or post-trauma* or adjustment disorder* or phobia* or phobic or obsessive compulsive or wellbeing or well-being) N5 (intervention? or counsel* or therap* or healing or listen* support* or care or healthcare or service or medication* or drug? or antidepress* or anti-depress* or antianxiety or anti-anxiety)))
S14	(MH "Antidepressive Agents+")
S13	(MH "Psychotherapy+") OR (MH "Cognitive Therapy+") OR (MH "Counseling+")
S12	(MH "Diagnosis") OR (MH "Early Diagnosis") OR (MH "Health Screening")
S11	S8 OR S9 OR S10

For peer review only - http://bmjopen.bmj.com/site/about/guidelines.xhtml

S10	TI (((pregnancy or pregnant or pre-nat* or prenat* or prepart* or prepart* or ante-nat* or antenat* or ante-part* or antepart* or peri-nat* or perinat* or peri-part* or peripart* or puerper* or post-nat* or postnat* or post-part* or postpart*) N5 (mental* or psych* or anxiety or anxious or depress* or mood? or affect* or distress* or stress or trauma* or posttrauma* or post-trauma* or "adjustment disorder*" or phobia* or phobic or "obsessive compulsive" or wellbeing or well-being))) OR AB (((pregnancy or pregnant or pre-nat* or prenat* or prepart* or prepart* or ante-nat* or antenat* or ante-part* or antepart* or peri-nat* or perinat* or peri-part* or peripart* or puerper* or post-nat* or postnat* or post-part* or postpart*) N5 (mental* or psych* or anxiety or anxious or depress* or mood? or affect* or distress* or stress or trauma* or posttrauma* or post-trauma* or "adjustment disorder*" or phobia* or phobic or "obsessive compulsive" or wellbeing or well-being))) OR TI ((((parent? or mother* or maternal or father* or paternal) N5 (infan* or newborn? or neonat* or baby or babies)) and (mental* or psych* or anxiety or anxious or depress* or mood? or affect* or distress* or stress or trauma* or posttrauma* or post-trauma* or "adjustment disorder*" or phobia* or phobic or "obsessive compulsive" or wellbeing or well-being))) AND AB ((((parent? or mother* or maternal or father* or paternal) N5 (infan* or newborn? or neonat* or baby or babies)) and (mental* or psych* or anxiety or anxious or depress* or mood? or affect* or distress* or stress or trauma* or posttrauma* or post-trauma* or "adjustment disorder*" or phobia* or phobic or "obsessive compulsive" or wellbeing or well-being)))
S9	(MH "Depression, Postpartum") OR (MH "Postpartum Psychosis") OR (MH "Expectant Mothers/PF")
S8	S4 AND S7
S7	S5 OR S6
S6	TI mental* or psych* or anxiety or anxious or depress* or mood? or affect* or distress* or stress or trauma* or posttrauma* or post-trauma* or adjustment disorder* or phobia* or phobic or obsessive compulsive or wellbeing or well-being
S5	(MH "Mental Disorders") OR (MH "Anxiety Disorders+") OR (MH "Affective Disorders+") OR (MH "Stress Disorders, Post-Traumatic+") OR (MH "Adaptation, Psychological")
S4	S1 OR S2 OR S3
S3	TI ((pregnancy or pregnant or pre-nat* or prenat* or prepart* or prepart* or ante-nat* or antenat* or ante-part* or antepart* or peri-nat* or perinat* or peri-part* or peripart* or puerper* or post-nat* or postnat* or post-part* or postpart*) OR TI (((parent? or mother* or maternal or father* or paternal) and (infan* or newborn? or neonat* or baby or babies)))
S2	(MH "Expectant Mothers")
S1	(MH "Prenatal Care") OR (MH "Postnatal Period") OR (MH "Pregnancy") OR (MH "Puerperium")
	**SCOPUS**

For peer review only - http://bmjopen.bmj.com/site/about/guidelines.xhtml

11	(((TITLE (pregnancy OR pregnant OR pre-nat* OR prenat* OR prepart* OR prepart* OR ante-nat* OR antenat* OR ante-part* OR antepart* OR peri-nat* OR perinat* OR peri-part* OR peripart* OR puerper* OR post-nat* OR postnat* OR post-part* OR postpart*) AND TITLE (mental* OR psych* OR anxiety OR anxious OR depress* OR mood* OR affect* OR distress* OR stress OR trauma* OR posttrauma* OR post-trauma* OR {adjustment disorder*} OR phobia* OR phobic OR {obsessive compulsive} OR wellbeing OR well-being))) AND ((TITLE-ABS-KEY ((screen* OR detect* OR diagnos* OR assess* OR identifi* OR prevent* OR prophyla*)) OR TITLE-ABS-KEY ((intervention* OR counsel* OR therap* OR healing OR listen* AND support* OR care OR healthcare OR service OR medication* OR drugs OR drug OR antidepress* OR anti-depress* OR antianxiety OR anti-anxiety)) OR TITLE-ABS-KEY (("improving access to psychological therap*" OR iapt)))) AND ((TITLE-ABS-KEY (implement* OR impact* OR feasib* OR acceptab*) OR TITLE-ABS-KEY ((barrier* OR challenge* OR obstacle* OR hurdle* OR obstruct* OR drawback* OR issue* OR difficult* OR promot* OR support OR encourag* OR factor? OR facilitat* OR enabl* OR opportunit* OR engage* OR assist*)) OR TITLE-ABS-KEY ("process evaluation*" OR "project evaluation*" OR "systems evaluation*" OR "system evaluation*")))) AND ((TITLE-ABS-KEY ("systematic review" OR meta-analys*) OR TITLE-ABS-KEY ("evidence synthesis" OR "realist synthesis" OR "realist review" OR meta-synthesis* OR "meta synthesis" OR metasynthesis OR meta-ethnograph* OR metaethnograph* OR "meta ethnograph*" OR meta-study OR metastudy OR "meta study") OR TITLE-ABS-KEY (qualitative AND synthesis))) AND (LIMIT-TO (LANGUAGE , "English"))
10	(((TITLE (pregnancy OR pregnant OR pre-nat* OR prenat* OR prepart* OR prepart* OR ante-nat* OR antenat* OR ante-part* OR antepart* OR peri-nat* OR perinat* OR peri-part* OR peripart* OR puerper* OR post-nat* OR postnat* OR post-part* OR postpart*) AND TITLE (mental* OR psych* OR anxiety OR anxious OR depress* OR mood* OR affect* OR distress* OR stress OR trauma* OR posttrauma* OR post-trauma* OR {adjustment disorder*} OR phobia* OR phobic OR {obsessive compulsive} OR wellbeing OR well-being))) AND ((TITLE-ABS-KEY ((screen* OR detect* OR diagnos* OR assess* OR identifi* OR prevent* OR prophyla*)) OR TITLE-ABS-KEY ((intervention* OR counsel* OR therap* OR healing OR listen* AND support* OR care OR healthcare OR service OR medication* OR drugs OR drug OR antidepress* OR anti-depress* OR antianxiety OR anti-anxiety)) OR TITLE-ABS-KEY (("improving access to psychological therap*" OR iapt)))) AND ((TITLE-ABS-KEY (implement* OR impact* OR feasib* OR acceptab*) OR TITLE-ABS-KEY ((barrier* OR challenge* OR obstacle* OR hurdle* OR obstruct* OR drawback* OR issue* OR difficult* OR promot* OR support OR encourag* OR factor? OR facilitat* OR enabl* OR opportunit* OR engage* OR assist*)) OR TITLE-ABS-KEY ("process evaluation*" OR "project evaluation*" OR "systems evaluation*" OR "system evaluation*")))) AND ((TITLE-ABS-KEY ("systematic review" OR meta-analys*) OR TITLE-ABS-KEY ("evidence synthesis" OR "realist synthesis" OR "realist

For peer review only - http://bmjopen.bmj.com/site/about/guidelines.xhtml

	review" OR meta-synthesis* OR "meta synthesis" OR metasynthesis OR meta-ethnograph* OR metaethnograph* OR "meta ethnograph*" OR meta-study OR metastudy OR "meta study") OR TITLE-ABS-KEY (qualitative AND synthesis)))
9	(TITLE-ABS-KEY ("systematic review" OR meta-analys*) OR TITLE-ABS-KEY ("evidence synthesis" OR "realist synthesis" OR "realist review" OR meta-synthesis* OR "meta synthesis" OR metasynthesis OR meta-ethnograph* OR metaethnograph* OR "meta ethnograph*" OR meta-study OR metastudy OR "meta study") OR TITLE-ABS-KEY (qualitative AND synthesis))
8	((TITLE (pregnancy OR pregnant OR pre-nat* OR prenat* OR prepart* OR prepart* OR ante-nat* OR antenat* OR ante-part* OR antepart* OR peri-nat* OR perinat* OR peri-part* OR peripart* OR puerper* OR post-nat* OR postnat* OR post-part* OR postpart*) AND TITLE (mental* OR psych* OR anxiety OR anxious OR depress* OR mood* OR affect* OR distress* OR stress OR trauma* OR posttrauma* OR post-trauma* OR {adjustment disorder*} OR phobia* OR phobic OR {obsessive compulsive} OR wellbeing OR well-being))) AND ((TITLE-ABS-KEY ((screen* OR detect* OR diagnos* OR assess* OR identifi* OR prevent* OR prophyla*)) OR TITLE-ABS-KEY ((intervention* OR counsel* OR therap* OR healing OR listen* AND support* OR care OR healthcare OR service OR medication* OR drugs OR drug OR antidepress* OR anti-depress* OR antianxiety OR anti-anxiety)) OR TITLE-ABS-KEY (("improving access to psychological therap*" OR iapt)))) AND ((TITLE-ABS-KEY (implement* OR impact* OR feasib* OR acceptab*) OR TITLE-ABS-KEY ((barrier* OR challenge* OR obstacle* OR hurdle* OR obstruct* OR drawback* OR issue* OR difficult* OR promot* OR support OR encourag* OR factor? OR facilitat* OR enabl* OR opportunit* OR engage* OR assist*)) OR TITLE-ABS-KEY ("process evaluation*" OR "project evaluation*" OR "systems evaluation*" OR "system evaluation*")))
7	(TITLE-ABS-KEY (implement* OR impact* OR feasib* OR acceptab*) OR TITLE-ABS-KEY ((barrier* OR challenge* OR obstacle* OR hurdle* OR obstruct* OR drawback* OR issue* OR difficult* OR promot* OR support OR encourag* OR factor? OR facilitat* OR enabl* OR opportunit* OR engage* OR assist*)) OR TITLE-ABS-KEY ("process evaluation*" OR "project evaluation*" OR "systems evaluation*" OR "system evaluation*"))
	**COCHRANE**
#1	MeSH descriptor: [Prenatal Care] explode all trees
#2	MeSH descriptor: [Perinatal Care] explode all trees

For peer review only - http://bmjopen.bmj.com/site/about/guidelines.xhtml

#3	MeSH descriptor: [Pregnancy] this term only
#4	MeSH descriptor: [Pregnant Women] explode all trees
#5	(pregnancy or pregnant or pre-nat* or prenat* or prepart* or prepart* or ante-nat* or antenat* or ante-part* or antepart* or peri-nat* or perinat* or peri-part* or peripart* or puerper* or post-nat* or postnat* or post-part* or postpart*):ti OR (((parent* or mother* or maternal or father* or paternal) and (infan* or newborn* or neonat* or baby or babies))):ti
#6	#1 or #2 or #3 or #4 or #5
#7	MeSH descriptor: [Mental Disorders] this term only
#8	MeSH descriptor: [Anxiety Disorders] explode all trees
#9	MeSH descriptor: [Mood Disorders] explode all trees
#10	MeSH descriptor: [Trauma and Stressor Related Disorders] explode all trees
#11	MeSH descriptor: [Stress, Psychological] explode all trees
#12	MeSH descriptor: [Adaptation, Psychological] this term only
#13	(mental* or psych* or anxiety or anxious or depress* or mood* or affect* or distress* or stress or trauma* or posttrauma* or post-trauma* or adjustment disorder* or phobia* or phobic or obsessive compulsive or wellbeing or well-being):ti
#14	#7 or #8 or #9 or #10 or #11 or #12 or #13
#15	#6 and #14
#16	MeSH descriptor: [Depression, Postpartum] explode all trees
#17	MeSH descriptor: [Pregnant Women] explode all trees and with qualifier(s): [psychology - PX]
#18	(((pregnancy or pregnant or pre-nat* or prenat* or prepart* or prepart* or ante-nat* or antenat* or ante-part* or antepart* or peri-nat* or perinat* or peri-part* or peripart* or puerper* or post-nat* or postnat* or post-part* or postpart*) NEAR/5 (mental* or psych* or anxiety or anxious or depress* or mood* or affect* or distress* or stress or trauma* or posttrauma* or post-trauma* or adjustment disorder* or phobia* or phobic or obsessive compulsive or wellbeing or well-being))):ti,ab,kw
#19	((((parent or parents or mother* or maternal or father* or paternal) NEAR/5 (infan* or newborn* or neonat* or baby or babies)) and (mental* or psych* or anxiety or anxious or depress* or mood* or affect* or distress* or stress or trauma* or posttrauma* or post-trauma* or adjustment disorder* or phobia* or phobic or obsessive compulsive or wellbeing or well-being))):ti,ab,kw
#20	#15 or #16 or #17 or #18 or #19
#21	MeSH descriptor: [Implementation Science] explode all trees

For peer review only - http://bmjopen.bmj.com/site/about/guidelines.xhtml

#22	MeSH descriptor: [Health Plan Implementation] explode all trees
#23	MeSH descriptor: [Program Evaluation] explode all trees
#24	(implement* or impact*):ti,ab,kw OR (feasib* or acceptab*):ti,ab,kw OR ((barrier* or challenge* or obstacle* or hurdle* or obstruct* or drawback* or issue* or difficult* or promot* or support or encourag* or factor* or facilitat* or enabl* or opportunit* or engage* or assist*)):ti,ab,kw OR (((process or project* or system*) NEAR/5 evaluat*)):ti,ab,kw
#25	#21 or #22 or #23 or #24
#26	#20 and #25

For peer review only

Appendix 3: Table 2. Search Results

**Database:**	**Interface:**	**Coverage:**	**Date:**	**Hits:**
CINAHL	EBSCOHost	1982-present	04/08/2021	759
Cochrane Database of Systematic Reviews	Cochrane Library, Wiley	Issue 8 of 12, August 2021	04/08/2021	384
Embase	OvidSP	1974-present	04/08/2021	1081
Medline	OvidSP	1946-present	04/08/2021	977
PsycINFO	OvidSP	1806-present	04/08/2021	286
Scopus	Elsevier		04/08/2021	599
**Total:**				**4086**
Duplicates:				1992
Papers excluded:				66
**Final total:**				**2028**

**Papers excluded:** available if needed	
Fetal distress	46
Oxidative stress	9
Non-English	11
	66

Included - 27th May 2020	1671
Included - 4th August 2021	374

For peer review only

For peer review only - http://bmjopen.bmj.com/site/about/guidelines.xhtml

Appendix 4: Table 3. Inclusion and exclusion criteria

**Category**	**Criteria**
Population	Women in the perinatal period (conception to 12 months after birth) experiencing mental health problems, who may or may not have decided to seek help, accessed help, or engaged in PNMH care. PNMH care was defined as assessment, referrals, and/or treatment/intervention programmes) from health or social care services. Conception to 12 months after birth was chosen as the target population because this is the period that many perinatal mental health services cover [1–5].<br><br>Reviews were excluded if they were not conducted on the target population (e.g., men/partners, healthcare professionals), focused on substance misuse (which has unique challenges in terms of assessment and treatment), did not focus on the mental health of perinatal women.
Outcome	Barriers and facilitators (defined as any individual, healthcare professional, interpersonal, organisational, political, or societal factors that women believed impeded (barriers) or aided (facilitators) them) to seeking, accessing, or engaging in help for PNMH problems. Studies were included if they made descriptive statements about barriers and facilitators to women deciding to seek help, accessing help, and engaging in PNMH care. These descriptions had to be drawn from perinatal women's experiences.<br><br>Reviews were excluded if they did not examine any barriers/facilitators regarding seeking help, accessing help and engaging in PNMH care.
Design	Only systematic reviews were included. Studies that did not use a clearly reported PRISMA search strategy [6]were excluded.<br><br>Non-English publications were also excluded.

For peer review only

For peer review only - http://bmjopen.bmj.com/site/about/guidelines.xhtml

Appendix 5: Detailed methodology

Protocol and registration

The protocol for this review has been registered on PROSPERO (CRD42020193107) (see appendix for full protocol).

Eligibility criteria

Studies with the following characteristics were eligible for inclusion in the review: Population: Women in the perinatal period (conception to 12 months after birth) experiencing mental health problems, who may or may not have decided to seek help, accessed help, or engaged in PNMH care. PNMH care was defined as assessment, referrals, and/or treatment/intervention programmes) from health or social care services. Conception to 12 months after birth was chosen as the target population because this is the period that many perinatal mental health services cover [1–5].

Outcome: Barriers and facilitators (defined as any individual, healthcare professional, interpersonal, organisational, political, or societal factors that women believed impeded (barriers) or aided (facilitators) them) to seeking, accessing, or engaging in help for PNMH problems.

Studies were included if they made descriptive statements about barriers and facilitators to women deciding to seek help, accessing help, and engaging in PNMH care. These descriptions had to be drawn from perinatal women's experiences. Only systematic reviews were included. Studies that did not use a clearly reported PRISMA search strategy [6]were excluded. Reviews were also excluded if they were not conducted on the target population (e.g., men/partners, healthcare professionals), focused on substance misuse (which has unique challenges in terms of assessment and treatment), did not focus on the mental health of perinatal women, did not examine any barriers/facilitators regarding seeking help, accessing help and engaging in PNMH care, and were non-English publications.

Information sources

Searches were carried out in CINAHL (1982- present); Embase (1974 – present); Medline (1946-present); and PsycINFO (1806 – present), Cochrane, SCOPUS and TRIP (Turning Research into practice) Medical Database. The date of the last search was 28th May 2020. Forward and backward searches of included studies were carried out and completed by the 26th June 2020. Searches were updated on 4th August 2021 and forward and backward searches of new included studies were completed by 8th September 2021.

Search

Pre-planned searches were carried out using both MeSH terms (i.e. prenatal care/anxiety/ diagnosis) and search terms were combined with Boolean operators "OR" and "AND" (e.g. pregnancy OR perinatal OR postnat* AND anxiety OR depress* OR wellbeing AND intervention? OR counsel* OR support OR identifi* AND OR barrier? OR facilitate*).

Review selection

Search results were imported into Endnote and duplicates and papers not meeting initial inclusion criteria (foetal distress, oxidative stress, Non-English papers) were removed by NR. The remaining studies were imported into Eppi-Reviewer 4, where results were double screened by title and abstract by two people (RW & GC). An additional proportion (n = 166, ~7%) of titles and abstracts were triple screened by NU. Decisions to include or exclude were concordant between RW & GC in 94.2% of cases and between RW & NU in 99.39% of cases. Disagreements were discussed and resolved by NU, GC, and RW by applying the relevant inclusion criteria.

For peer review only - http://bmjopen.bmj.com/site/about/guidelines.xhtml

Once title and abstract screening was complete, full text screening was carried out by two people (RW & GC). An additional proportion (n = 9, ~10%) were triple screened by NU. Decisions to include or exclude were concordant between RW & GC in 91.4% of cases and between RW & NU in 100% of cases.

Data collection process and data items

Data extraction was carried out using Microsoft Excel by RW. Each paper was read in full, and relevant parts of the text input into the relevant part of the spreadsheet. Review methodology was copied onto one sheet and results onto another to aid analysis. Double coding of extracted data was carried out for a proportion of included reviews (n = 3, 10%) by GC. Data extraction matched in 85% of cases.

The data that were extracted was guided by the Cochrane Systematic Review for Intervention Data Collection form [7] and the Assessing the Methodological Quality of Systematic Reviews (AMSTAR 2;[8]) Data collected included the following: Review Characteristics (year of publication, author(s), design, aim, search strategy, inclusion/exclusion criteria, screening/study selection, data extraction, quality assessment, analysis methods); Sample Characteristics (Number of studies included, total number of participants, participant demographics); Assessment/Care/Treatment Characteristics (Healthcare setting, intervention description, screening description) and outcomes (barriers and facilitators).

Critical appraisal of reviews

Methodology sections of included systematic reviews were appraised using the AMSTAR 2. Critical domains in the appraisal of systematic reviews according to AMSTAR 2 include protocol registration, adequacy of literature search, justification of study exclusion, risk of bias, appropriateness of meta-analytic methods, consideration of risk of bias when interpreting results, and assessment of publication bias. If more than one critical domain is not met (critical flaw), a systematic review should be evaluated as having critically low confidence in the results of the review. One critical flaw means reviews should be evaluated as low confidence [8].

Given that all studies in this review were qualitative, the AMSTAR 2 items related to meta-analysis were not relevant and were thus removed. Further, given the debate in the literature regarding the appropriateness of conducting risk of bias assessments on qualitative research, we downgraded the items relating to risk of bias from being critical flaws, to flaws. Quality appraisal of all studies was carried out by NU and RW. Ratings were concordant in 90% of cases.

A decision was made to continue to include reviews where confidence in results was evaluated as low and critically low because these reviews focused more on marginalised women, such as refugees, migrants, women with a low income, and women living in LMIC, to ensure the experiences of these seldom-heard women were captured. To improve the validity of results, a qualitative sensitivity analysis was carried out to assess whether themes remained consistent across all reviews, regardless of their quality rating. The methods proposed by Harden[9] and Carroll et al[10] was followed and therefore sensitivity analysis was carried out in two ways: (1) synthesis contribution; (2) evidence of adequate description of themes.

To examine whether higher quality studies contributed more to the themes, a measure of “synthesis contribution” was calculated for each study (as done by Harden, 2007[9]) by dividing the number of barriers and facilitators identified by that study, by the total number of barriers and facilitators identified in the review overall. For example, the findings from Bina (2020) [11]contributed to 31 out of 62 themes, giving this review a synthesis contribution score of 50% (see appendix, Table 3). Each study's synthesis contribution scores was plotted against the number of quality criteria the study

For peer review only - http://bmjopen.bmj.com/site/about/guidelines.xhtml

met (see appendix, Figure 1). Statistical analysis (Pearson's correlation) was used to help interpret the plots. To examine whether removing lower quality reviews influenced the number of themes, themes that were only supported by lower quality reviews were identified (see appendix, Table 6).

To examine whether removing lower quality reviews influenced the description of themes, data were assessed for "thickness" or "thinness" (as done by Carroll et al., 2012[10]). Thin description refers to a set of statements (e.g. "O'Mahoney et al. found that women also felt that providers were downplaying the symptoms they were experiencing", Hansotte et al., 2017, [12]p.12), whereas thick description provides the context of experience and circumstances [13] (e.g. "Having symptoms dismissed or attributed to factors other than PPD by health care professionals led to women 'remaining silent.' Some women perceived that their difficulties would only be taken seriously when there were concerns about risk of harm to themselves or the infant. One woman said, 'I kept going to this doctor and he used to give me a pep talk and send me home…'", Hadfield & Wittkowski, 2017[14], p.732). It is argued that the extent to which a text provides a thick description shows evidence of the authenticity of the results [15].

Synthesis of results

Results were analysed by RW using a thematic synthesis [16] in NVivo and Microsoft Excel. First, line by line data coding of statements referring to facilitators or barriers to accessing PNMH care from the results section of each paper was carried out. Next, codes were revisited and assigned a descriptive theme based on their meaning and content. Themes were developed and revised as each review was re-read. Once all codes had been assigned into themes, the themes were mapped onto a multi-level framework adapted from Ferlie and Shortell's Levels of Change framework [17] and a previous systematic review on barriers and facilitators to implementation of PNMH care carried out by the review authors. The mapping of descriptive themes was developed deductively from the initial theoretical framework and then inductively revised as new themes emerged. The mapping of descriptive themes was discussed by all review authors before being finalised. A decision was taken to analyse all reviews together, regardless of the specific aims or individual inclusion criteria. This is because the majority of the reviews (n = 27) included studies carried out in a wide range of countries/settings. This, therefore, made it difficult to parse apart reviews based on sample characteristics, settings, or country of included studies.

For peer review only

Appendix 6: Table 4. Sensitivity analysis

Study	Number of themes	Unique synthesis contribution	Overall synthesis contribution (all themes)
Bina, 2020[11]	31	3.03030303	50
Brealey et al., 2010[18]	13	1.515151515	20.96774194
Button et al., 2017[19]	26	0	41.93548387
Dennis & Chung-Lee, 2006[20]	28	0	45.16129032
Evans et al., 2020[21]	8	0	12.90322581
Giscombe et al., 2020[22]	6	0	9.677419355
Forde,et al. 2020[46]	20	0	29.03
Hadfield & Wittkowski, 2017[14]	25	0	40.32258065
Hansotte et al., 2017[12]	19	1.515151515	30.64516129
Hewitt et al., 2009[23]	13	0	20.96774194
Holopainen & Hakulinen, 2019[24]	6	0	9.677419355
Jones et al., 2014[25]	10	0	16.12903226
Jones, 2019[26]	19	0	30.64516129
Kassam, 2019[27]	8	0	12.90322581
Lucas et al., 2019[28]	9	0	14.51612903
Megnin-Viggars et al., 2015[29]	26	0	41.93548387
Mollard et al., 2016[30]	5	1.515151515	8.064516129
Morrell et al., 2016[31]	16	0	25.80645161
Newman et al., 2019[32]	13	0	20.96774194
Nilaweera et al., 2014[33]	6	0	9.677419355
Praetorius et al., 2020[34]	3	0	4.838709677
Randall & Briscoe, 2018[35]	2	0	3.225806452
Sambrook-Smith et al., 2019[36]	19	0	30.64516129
Schmied et al., 2017[37]	27	1.515151515	43.5483871
Scope et al., 2017[38]	13	0	20.96774194
Slade et al., 2020[39]	15	0	24.19354839
Sorsa et al., 2021[40]	19	0	30.64516129

For peer review only - http://bmjopen.bmj.com/site/about/guidelines.xhtml

For peer review only

Staneva et al., 2015[41]	11	0	17.74193548
Tobin et al., 2018[42]	19	0	30.64516129
Viveiros & Darling, 2018[43]	16	1.515151515	25.80645161
Watson et al., 2019[44]	28	0	45.16129032
Wittkowski et al., 2014[45]	2	1.515151515	3.225806452

For peer review only

For peer review only - http://bmjopen.bmj.com/site/about/guidelines.xhtml

Appendix 7: *Figure 1.* Synthesis contribution vs quality appraisal criteria met

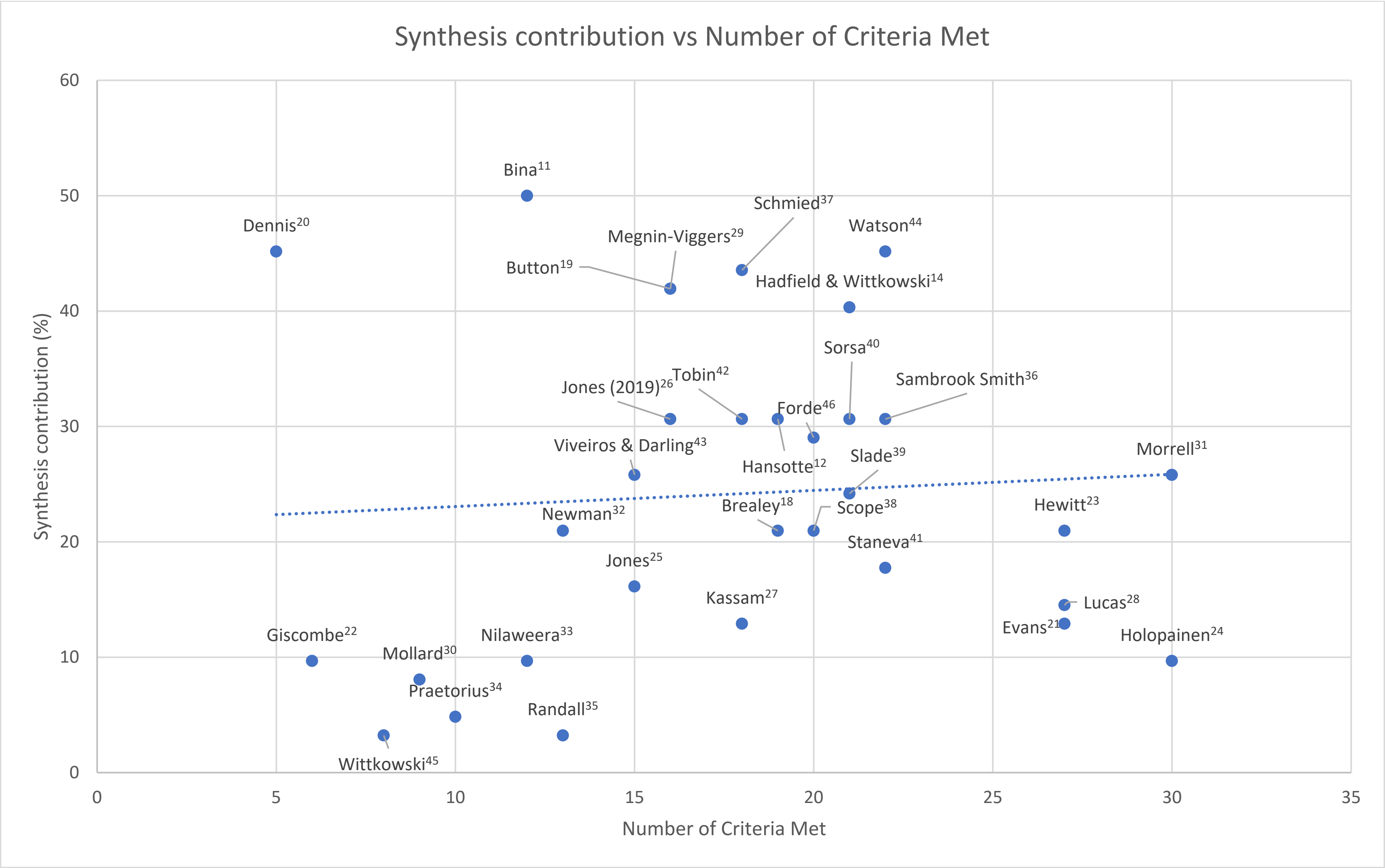

35

Appendix 8: Sensitivity analysis results

Synthesis contribution. There was no correlation between synthesis contribution and the number of criteria each review met (r = .142, p = .437; see appendix, Figure 1). Furthermore, only four themes (**cultural/spiritual** causes of mental illness, **age, previous diagnoses,** and **appropriateness of care)** were only identified by lower quality studies showing the majority of themes (58 out of 62; 93.55%) were supported by both higher quality and lower quality papers.

Richness of data. The removal of lower quality papers meant that the theme **language barriers** lost some of its richness. For example, it led to the removal of quotes expressing frustration from women whose first language was not English:

> '. . .you don't know where to go, what to do, who to trust, especially when you are coming by yourself. . . you believe that you speak English, but when you get here you realize that you don't.' [37]
>
> 'Sometimes when you have a baby, a woman comes from the hospital. Bengali girls don't come with the midwife, we don't understand what they say, we just sit there staring at their faces.' [19]

The removal of lower quality papers from the theme **fear of being seen as a bad mum** led to the loss of richness of data including the removal of a quote from women who have migrated from their country of birth:

> 'Back home, if someone has this problem, everyone gossips, you get this feeling that people are not dealing with you normally or as if you are abnormal almost. . .' [37]

Lastly, the removal of lower quality studies meant important information was removed from the **characteristics of service** theme, such as women feeling services prioritise physical needs (n = 2), lack information about screening guidelines (n = 2), and the logistics (e.g. location, time of appointments) of the care (n = 3)

For peer review only

For peer review only - http://bmjopen.bmj.com/site/about/guidelines.xhtml

Appendix 9: Table 5. Summary review characteristics

**Characteristics**	**Range; Mean (M), Median (Mdn); Interquartile Range (IQR)**
Year	2006-2022; M = 2017, Mdn = 2018; IQR = 2016-2019
No. studies included in each review	4-40; M = 16, Mdn = 13, IQR = 9-19
No. women included in each review	95-85,190; M = 5080; Mdn = 463; IQR = 226-1,715
Countries	N = 24

Appendix 10: Table 6. Characteristics of included reviews

	**Review details**			**Participant details**					
**Author & Year**	**N studies about women (Total N)**<br><br>**Years (Range)**	**Inclusion criteria**	**Country of studies**	**N**<br><br>**M (SD)**	**Age**	**Perinatal period**	**Ethnicity**	**Mental illness**	**Socioeconomic status**
Bina (2020)[11]	31 (35)<br><br>1993-2018	Service use for postpartum depression or “distress” from women (and HCP) perspectives	11 countries (4 LMIC)	7219<br><br>232.9 (414.7)	NR	2 weeks - up to 3 years postnatal	6 studies specified recruitment of migrant women or women of colour[(EA; H; Ar)]	Depressive symptoms, emotional difficulties or current/past diagnosis of mood disorder	2 studies recruited women with low income.<br>1 study recruited women using Medicaid.
Brealey et al. (2010)[18]	13 (16)<br><br>1997-2007	Acceptability to women (and HCP) of screening to identify women with increased risk of	5 countries (all HIC)	1715<br><br>131.9 (253.06)	24-34<br>M (n = 8) = 29.63	First antenatal appointment – 12 months after birth	2 studies recruited women of colour [(B; EA; Ar)]	Women at risk of postnatal depression	One study reported marriage (29/30 women were married)

For peer review only - http://bmjopen.bmj.com/site/about/guidelines.xhtml

		postnatal depression							
Button et al. (2017)[19]	24 (24)<br><br>1993-2016	Help seeking for postnatal depression	9 studies carried out in UK. No other countries reported.	NR	NR	Postnatal	9 studies recruited women of colour. 3 studies had mixed samples.	Postnatal depression	NR
Dennis and Chung-Lee (2006)[20]	40 (40)<br><br>NR	Maternal help-seeking barriers and facilitators and treatment preferences for postnatal depression	3 were explicitly stated (all HIC)	NR	NR	Up to 1 year after birth	Three studies recruited women of colour[(SA; EA;B;Ar)]	Postnatal depression	NR
Evans et al. (2020)[21]	14 (14)<br><br>2009-2015	Acceptability of non-pharmacological interventions for antenatal anxiety	6 countries (all HIC)	235<br><br>16.8 (9.6)	NR	Between 6-40 weeks gestation	NR	8 studies recruited women with a history of mood concerns/ anxiety or depression	2 studies recruited women with "social risk factors"
Forde, Peters & Wittkowski (2020)[46]	13 (15)<br><br>2003-2018	Published empirical studies exploring women's or family members' experiences of PP and/or recovery using	4 countries (all HIC)	103<br>7.92 (2.96)	Range: 23-62	All postnatal, ranging from 4 months to 26 years after onset of postnatal psychosis	One woman was an Orthodox Jewish woman	All women had recovered from, or were currently experiencing postnatal psychosis	NR

For peer review only - http://bmjopen.bmj.com/site/about/guidelines.xhtml

		a qualitative methodology							
Giscombe, Hui & Stickley (2020)[22]	8 (8)<br><br>2008-2017	Refugee or asylum-seeking women, with mental health complications during perinatal period	3 countries (all HIC)	NR	NR	NR	Syrian refugees, Eritrean refugees	6 studies recruited women with depression; 3 with PTSD (1 study recruited both depression & PTSD)	All women were refugees or asylum seekers
Hadfield and Wittkowski (2017)[14]	17 (17)<br><br>2004-2015	Mothers with postnatal depression and their experiences about help seeking for psychosocial support	4 countries (all HIC)	532<br><br>31.3 (25.97)	Range 18-45<br><br>M (n = 2) = 30.2	Postnatal	3 studies recruited women who weren't born in the UK[(B;EA)]	Postnatal depression	NR
Hansotte et al. (2017)[12]	18 (18)<br><br>2004-2015	Screening for postnatal depression and barriers to accessing treatment in low-income women in western countries.	2 countries (all HIC)	85190<br><br>5011 (11613)	M (n = 11) = 25.11	Postnatal	All studies recruited a diverse sample of migrant women or women of colour [(B; L; W; As; NI)]	Self-report depression symptoms or depression diagnosis	All women were low income living in high income western country.
Hewitt et al. (2009)[23]	13 (16)<br><br>1997-2007	Acceptability to women (and HCP) about methods to	5 countries (all HIC)	1715<br><br>131.9 (253.06)	M (n = 8) = 29.63	Postnatal: 1-12 months	4 studies recruited women of	Perinatal depression	2 studies looked at marriage. The majority of women

For peer review only - http://bmjopen.bmj.com/site/about/guidelines.xhtml

		identify postnatal depression				Antenatal: all trimesters	colour (Ar; EA; B; NI; NS)		were married (87-97%)
Holopainen and Hakulinen (2019)[24]	13 (15)<br><br>2005-2015	Mothers (and fathers) experiences of postnatal depression symptoms	7 countries (all HIC)	199<br><br>15.31 (8.21)	Ages ranged from 16-45	1-12 months after birth	5 studies recruited women of colour (B, L, H, SA, EA)	Most studies looked at symptoms of depression, 2 looked at diagnoses	1 study recruited low-income women, one recruited adolescent mothers. Most women were married (n = 3; 59-66%). Most women had more than 9 years of education (n = 2; 87-100%)
Jones et al. (2014)[25]	5 (5)<br><br>1995-2012	Women's experiences of peer support for any degree of perinatal mental illness	3 countries (all HIC)	95<br><br>19 (18.93)	NR	6 weeks - 2 years after birth	NR	Postnatal depression diagnosis or symptoms	NR
Jones (2019)[26]	19 (19)<br><br>2008-2017	Help seeking in women with perinatal depression	All USA	6089<br><br>358.90 (1226.22)	NR	Pregnancy – 6 months after birth	6 studies recruited women of colour (B, L, SA, EA, NS)	All had perinatal depression identified through screening measures, or self-reported.	All women had pregnancy complications. 3 studies recruited women on a low income.
Kassam (2009)[27]	11 separate populations<br><br>1999-2013	Voices of immigrant and refugee women with postnatal depression in terms of social support as a	3 countries stated (HIC & UMIC countries)	191<br><br>23.88 (10.89)	All aged over 17	Screened high on a postnatal depression scale within 2 weeks - 5 years after birth	All studies recruited migrant women or women of colour (NS; As; Ar; SA; H)	Most had postnatal depressive symptoms, identified through screening. One study reported	One study looked at risk profile of women (e.g. low income, experienced violence, experienced war, previous mental health difficulty).

For peer review only - http://bmjopen.bmj.com/site/about/guidelines.xhtml

		coping resource						depression diagnosis	All women in 2 studies were married or in a relationship. One study recruited low-income women.
Lucas et al. (2019)[28]	19 (19)<br><br>1999-2017	Young women's perception of their mental health and wellbeing	3 countries (all HIC)	356<br><br>18.74 (10.02)	Ages ranged from 13-25.<br><br>M (n = 2) = 18.75	11 studies recruited were parents (3 months - 2 years postnatal). 2 studies recruited pregnant women. Remaining studies recruited both pregnant and postnatal women	Majority of studies (15) recruited ethnically diverse [L, B, H, SA, M, As] samples. 4 studies did not report ethnicity	Depressive symptoms, depression diagnosis, other diagnoses (bipolar, panic disorder, mood disorder).	All women were young (maximum age 25)
Megnin-Viggars et al. (2015)[29]	39 (39)<br><br>2001-2013	Women with, or at risk of developing a postnatal mental health problem and their views on factors that improve or	Only reported for 3 studies (all UK)	955<br><br>24.49 (43.77)	1 study recruited teenage mother. No other ages reported.	Antenatal and postnatal	5 studies recruited ethnically diverse samples [B, NS, SA]	Most studies recruited women with depression (n = 14) or women at risk (n = 18) of perinatal mental health problems.	1 study recruited teenage mothers

For peer review only - http://bmjopen.bmj.com/site/about/guidelines.xhtml

		diminish access to perinatal mental health services							
Mollard et al. (2016)[30]	11 (11)<br><br>1995-2014	Women living in rural areas of the USA with PPD. Looking at screening uptake, intervention acceptability, lived experience, help-seeking.	All USA	1610<br><br>146.36 (159.57)	NR	Postnatal	5 studies recruited ethnically diverse samples (NI; B; NS; H)	Postnatal depression symptoms, most used EPDS[1] (n = 6) screen	All women lived in rural location, 3 studies recruited low income women
Morrell et al. (2016)[31]	38 individual samples of women in the qualitative review<br><br>1987-2013	Pregnant and postnatal women, views on preventative or targeted services for PND	8 countries (1 LMIC – India)	1673 (34 studies reported sample size)<br><br>49.21 (98.49)	Ages ranged from 15-54<br><br>M (n = 12) = 28.62	Pregnancy and postnatal	10 recruited ethnically diverse samples (SA; EA, B; H; NI; L; M; NS)	Depression - both symptoms and diagnoses	25 studies reported sociodemographic characteristics. 16 studies reported marital status, in all but 1 study the majority of women were married/cohabiting/in a relationship. 8 studies reported education status: most had completed high school or above. 4 studies recruited low-income women or those living in an

For peer review only - http://bmjopen.bmj.com/site/about/guidelines.xhtml

									impoverished/deprive d area
Newman et al. (2019)[32]	4 (4)<br><br>2008-2016	Women with depression during the postnatal period sharing views on help-seeking	3 countries (all HIC)	118<br><br>29.5 (9)	M (n = 3) = 31.97	Postnatal	NR	Depressive symptoms, measured by EPDS[1]	NR
Nilaweera et al. (2014)[33]	9 (15)	Women who have migrated from South Asian countries to live in high-income countries, barriers and enablers to health care access	4 countries (all HIC)	20,788<br><br>2309.78 (3926.13)	NR	2 weeks to 5 years postnatal	All studies recruited women born in South Asia	Most (n = 5) used EPDS[1] to assess postnatal depression symptoms	NR
Praetorius, Maxwell & Alam (2020)[34]	8 (8)<br><br>1999-2016	Mothers with depression and suicidality	5 countries (3 HIC, 1 UMIC, 1LMIC)	199<br><br>24.88 (12.52)	Ages range from 17-44	Pregnancy and postnatal	All studies recruited diverse samples (B, L, M, SA, EA, Ar, W)	All women had depression and suicidality	NR
Randall and Briscoe (2018)[35]	4 (4)<br><br>2005-2014	Women's decision-making process around antidepressant	2 countries (all HIC)	368<br><br>92 (37.09)	Ages ranged from 25-34	Pregnancy	3 studies reported ethnicity. The majority of women	Depression – 1 study used the CES-D[2] to identify depressive symptoms	3 studies report education, the majority (82.5-100%) were educated to above high school level. 3 studies

For peer review only - http://bmjopen.bmj.com/site/about/guidelines.xhtml

		use during pregnancy			M (n = 2) = 31		were white (77.5-95%)		reported relationship status, the majority (80-98%) were married/living with partner
Slade, Molyneux & Watt (2021)[39]	13 (13 – qualitative papers only)<br><br>2007-2019	Help seeking for birth trauma/ postnatal PTSD	7 countries (1 UMIC; 6 HIC)	394<br><br>30.31 (32.85)	Ages range from 2-45<br><br>M (n=4) = 32	Up to 18 months after birth	8 studies reported ethnicity. One study reported recruiting women of colour (B, H)	All PTSD after birth	One study recruited low-income women. 2 studies reported marital status, over 58% were married. 2 studies reported higher education, at least 50% of women had completed this.
Sambrook-Smith et al. (2019)[36]	24 (35)<br><br>2007 - 2018	Barriers to accessing perinatal mental health care from the perspective of women (families & HCP)	All UK	384<br><br>16 (8.80)	NR	Postnatal	9 recruited women of colour (B; SA; EA)	Most looked at depressive symptoms (n = 12). Studies also recruited women with antenatal anxiety (n = 1), postnatal psychosis (n = 5), PTSD (n = 1) and substance misuse (n =1)	NR
Sorsa, Kylma and Bondas (2021)[40]	14 (14)<br><br>2002-2018	Helpseeking in women with perinatal distress	5 countries (all HIC)	345<br><br>24.65 (11.99)	Ages ranged from 18-55	Antenatal and postnatal	NR	Postnatal depression (n -= 8); prenatal depression (n = 2); Perinatal	NR

For peer review only - http://bmjopen.bmj.com/site/about/guidelines.xhtml

					M (n = 7) = 30.21			mental health needs (n = 2); Postpartum mood disorder (n = 1), Bipolar disorder (n = 1)	
Schmied et al. (2017)[37]	12 individual samples<br><br>1999-2015	Migrant women living in high income countries	4 countries (all HIC)	250<br><br>20.83 (12.52)	M (n = 5) = 29.4	Postnatal	All studies recruited migrant women or women of colour (SA; EA; H; B; Ar; L)	Depressive symptoms or formal diagnosis	1 study recruited low income women
Scope et al. (2017)[38]	22 individual samples<br><br>1987 - 2014	Service user views on uptake, acceptability of preventative interventions for PND	7 countries (all HIC)	982 (reported by author)	13-45 years	Antenatal and postnatal	NR	NR	NR
Staneva et al. (2015)[41]	8 (8)<br><br>2006-2012	Womens experience of antenatal mental health difficulties	5 countries (1 LMIC - Cambodia)	1094<br><br>14 (6.26)	Ages ranged from 16-47	Antenatal	Most studies (n = 6) recruited ethnically diverse samples (B; M)	Self-report distress, depression (n = 5); diagnoses depression/anxiety (n = 2); FOC = 1	50-100% of women were in a relationship
Tobin et al. (2018)[42]	8 (individual samples)	Refugee or immigrant women's experiences of	3 countries (all HIC)	139<br><br>17.38 (7.98)	Age ranges between	Postnatal	All studies recruited migrant women	Postnatal depression	6 studies reported relationship status 50-85% of women were

For peer review only - http://bmjopen.bmj.com/site/about/guidelines.xhtml

	2004-2013	postpartum depression			17-54 years		and women of colour[L; H; SA;EA;B]		married/in a relationship
Viveiros and Darling (2019)[43]	7 (26)<br><br>2009-2018	To explore women (and midwives) perceptions on factors that impede access to perinatal mental health care in high resource settings	2 countries (both HIC)	301<br><br>43 (66.30)	1 study reported age range from 23-40	Antenatal and postnatal	2 studies recruited 'BAME' women, one recruited all Black women	PTSD symptoms (n = 1); mental health problems (n = 2); mental illness diagnosis (n = 1)	NR
Watson et al. (2019)[44]	15 (15)<br><br>1994-2015	Ethnic minority women's experience of perinatal mental ill health, help-seeking and perinatal mental health services in Europe	All UK	4970<br><br>331.33 (1173.09)	NR	Antenatal and postnatal	All studies recruited women of colour [SA; NS; N; EA; M]	Distress, depression, mood and mental health, well-being	NR
Wittkowski et al. (2014)[45]	12 (12)<br><br>1983 - 2009	Culturally determined risk factors of PND in Sub-Saharan Africa	3 countries – all Sub-Saharan Africa	3642<br><br>404.67 (343.16)	NR	Postnatal	NR	All used self-report measures of depression	NR

For peer review only - http://bmjopen.bmj.com/site/about/guidelines.xhtml

Note. Where studies recruited populations that were not perinatal women, the information from these studies are not included in this table. HCP = Healthcare professional; LMIC = Lower-Middle Income Country; HIC = Higher Income Country; PTSD = Post-traumatic stress disorder; FOC = Fear of Childbirth. 1 = Edinburgh Postnatal Depression Scale (Cox et al., 1987); 2 = Center for Epidemiological Studies-Depression (Radloff, 1977).

For ethnicities: As = Asian (where the area of Asia was not specified in the study); EA = East Asian (e.g. Vietnamese; Chinese; Thai); SA = South Asian (e.g. Indian; Bangladeshi; Pakistani; Sri-Lankan); Ar = Arab countries (e.g. Jordanian, Egyptian); Ar = Arabic; B = Black; H = Hispanic; L = Latina; M = mixed or multiple ethnic groups; NI = Native/Indigenous; NS = Not specified; W = White.

For peer review only

For peer review only - http://bmjopen.bmj.com/site/about/guidelines.xhtml

Appendix 11: Table 7. Quality appraisal

Author, year	Q1. PICO	Q2. Protocol*	Q3. Study design	Q4. Literature search*	Q5. Study selection	Q6. Data extraction	Q7. Excluded studies*	Q8. Included studies	Q9. ROB	Q.10 Funding	Q13. ROB interpretation	Q14. Heterogeneity	Q16. conflict of interest*	Rating
**Bina, 2020[11]**	Yes	Yes	No	Partial yes	Yes	No	Partial yes	Yes	No	No	No	Yes	No	LOW
**Brealey et al., 2010[18]**	Yes	Yes	Yes	Yes	Yes	Yes	No	Yes	Partial yes	No	Yes	Yes	No	CRITICALLY LOW
**Button et al., 2017[19]**	Yes	No	Yes	Yes	No	No	Partial yes	Partial yes	Yes	No	Yes	Yes	Yes	LOW
**Dennis & Chung-Lee, 2006[20]**	Yes	No	Yes	Yes	No	Yes	No	Partial yes	No	No	No	No	No	CRITICALLY LOW
**Evans et al., 2020[21]**	Yes	Yes	Yes	Yes	Yes	Yes	Partial yes	Yes	Yes	No	Yes	Yes	Yes	HIGH
**Forde et al., 2020[46]**	Yes	Yes	Yes	Partial yes	Yes	No	Partial yes	Ys	Yes	No	Yes	Yes	Yes	MODERATE
**Giscombe et al., 2020[22]**	Yes	No	Yes	Partial yes	No	No	Partial yes	Yes	Yes	No	No	Yes	No	CRITICALLY LOW
**Hadfield & Wittkowski, 2017[14]**	Yes	Yes	Yes	Yes	No	No	Partial yes	Yes	Yes	No	Yes	Yes	Yes	MODERATE
**Hansotte et al., 2017[12]**	Yes	No	Yes	Yes	Yes	Yes	Partial yes	Yes	No	No	No	Yes	Yes	LOW
**Hewitt et al., 2009[23]**	Yes	Yes	Yes	Yes	Yes	Yes	Partial yes	Yes	Yes	No	Yes	Yes	Yes	HIGH
**Holopainen & Hakulinen, 2019[24]**	Yes	Yes	Yes	Yes	Yes	Yes	Yes	Yes	Yes	No	Yes	Yes	Yes	HIGH

For peer review only - http://bmjopen.bmj.com/site/about/guidelines.xhtml

**Jones et al., 2014[25]**	Yes	No	Yes	Yes	No	Yes	No	Partial yes	Yes	No	Yes	Yes	Yes	CRITICALLY LOW
**Jones, 2019[26]**	Yes	No	Yes	Partial yes	Yes	No	Partial yes	Yes	No	No	Yes	Yes	Yes	LOW
**Kassam, 2019[27]**	Yes	Yes	Yes	Partial yes	No	No	Partial yes	Yes	Yes	No	Yes	Yes	Yes	MODERATE
**Lucas et al., 2019[28]**	Yes	Yes	Yes	Yes	Yes	Yes	Partial yes	Yes	Yes	No	Yes	Yes	Yes	HIGH
**Megnin-Viggars et al., 2015[29]**	Yes	Yes	Yes	Partial yes	No	Yes	Partial yes	Yes	Yes	No	Yes	Yes	No	LOW
**Mollard et al., 2016[30]**	Yes	No	Yes	Partial yes	No	No	Partial yes	Partial yes	No	No	No	Yes	Yes	LOW
**Morell et al. 2016[31]**	Yes	Yes	Yes	Yes	Yes	Yes	Yes	Yes	Ye	No	Yes	Yes	Yes	HIGH
**Newman et al., 2019[32]**	Yes	No	Yes	Yes	No	No	Partial yes	Yes	Yes	No	No	No	Yes	LOW
**Nilaweera et al., 2014[33]**	Yes	No	No	Partial yes	No	No	Partial yes	Yes	Yes	No	Yes	Yes	Yes	LOW
**Praetorius et al., 2020[34]**	No	No	Yes	Yes	No	Yes	Partial yes	Partial yes	No	No	No	No	Yes	LOW
**Randall & Briscoe, 2018[35]**	Yes	No	No	Partial yes	Yes	No	Partial yes	Partial yes	Yes	No	No	Yes	Yes	LOW
**Sambrook-Smith et al., 2019[36]**	Yes	Yes	Yes	Partial yes	Yes	No	Partial yes	Yes	Yes	No	Yes	Yes	Yes	MODERATE
**Schmied et al., 2017[37]**	Yes	No	Yes	Partial yes	Yes	No	Partial yes	Yes	Yes	No	Yes	Yes	Yes	LOW
**Scope et al., 2017[38]**	Yes	Yes	Yes	Partial yes	Yes	Yes	Partial yes	Yes	Yes	No	Yes	Yes	No	LOW

For peer review only - http://bmjopen.bmj.com/site/about/guidelines.xhtml

**Staneva et al., 2015[41]**	Yes	Yes	Yes	Partial yes	Yes	No	Partial yes	Yes	Yes	No	Yes	Yes	Yes	MODERATE
**Slade et al., 2020[39]**	Yes	Yes	Yes	Yes	No	No	Partial yes	Yes	Yes	No	Yes	Yes	Yes	MODERATE
**Sorsa et al., 2021[40]**	Yes	No	Yes	Partial yes	Partial yes	Yes	Partial yes	Yes	Yes	No	Yes	Yes	Yes	LOW
**Tobin et al., 2018[42]**	Yes	Yes	Yes	Yes	No	No	No	Yes	Yes	No	No	Yes	Yes	MODERATE
**Viveiros & Darling, 2018[43]**	Yes	No	No	Yes	Yes	No	Partial yes	Yes	No	No	No	Yes	Yes	LOW
**Watson et al., 2019[44]**	Yes	Yes	Yes	Partial yes	Yes	No	Partial yes	Yes	Yes	No	Yes	Yes	Yes	MODERATE
**Wittkowski et al., 2014[45]**	Yes	No	Yes	Partial yes	No	No	Partial yes	Yes	Yes	No	Yes	Yes	No	CRITICALLY LOW

* = Critical domain

1. Did the research questions and inclusion criteria for the review include the components of PICO? *2.* Did the report of the review contain an explicit statement that the review methods were established prior to the conduct of the review and did the report justify any significant deviations from the protocol?*3.* Did the review authors explain their selection of the study designs for inclusion in the review?4. Did the review authors use a comprehensive literature search strategy? 5. Did the review authors perform study selection in duplicate? 6. Did the review authors perform data extraction in duplicate? 7. Did the review authors provide a list of excluded studies and justify the exclusions? 8. Did the review authors describe the included studies in adequate detail? 9. Did the review authors use a satisfactory technique for assessing the risk of bias (RoB) in individual studies that were included in the review? 10. Did the review authors report on the sources of funding for the studies included in the review? 11. If meta-analysis was performed, did the review authors use appropriate methods for statistical combination of results? *(not applicable)* 12. If meta-analysis was performed, did the review authors assess the potential impact of RoB in individual studies on the results of the meta-analysis or other evidence synthesis? *(not applicable)* 13. Did the review authors account for RoB in primary studies when interpreting/discussing the results of the review? 14. Did the review authors provide a satisfactory explanation for, and discussion of, any heterogeneity observed in the results of the review? 15. If they performed quantitative synthesis did the review authors carry out an adequate investigation of publication bias (small study bias) and discuss its likely impact on the results of the review? *(not applicable)* 16. Did the review authors report any potential sources of conflict of interest, including any funding they received for conducting the review?

For peer review only - http://bmjopen.bmj.com/site/about/guidelines.xhtml

Appendix 12: Table 8. Themes

Theme	Studies reflecting this theme
**1. Women**	
**1.1 Beliefs about health services**	
**1.1.1 Medication only**	Bina, 2020; Button et al., 2017; Jones, 2019; Megnin-Viggars et al., 2015; Nilaweera et al., 2014; Sorsa et al., 2021; Tobin et al., 2018
**1.1.2 Stretched**	Hadfield & Wittkowski, 2017
**1.2 Beliefs about healthcare professionals**	
**1.2.1 What is their role?**	Brealey et al., 2010; Button et al., 2017; Dennis & Chung-Lee, 2006; Hadfield & Wittkowski, 2017; Hewitt et al., 2009; Megnin-Viggars et al., 2015; Mollard et al., 2016; Morrell et al., 2016; Nilaweera et al., 2014; Schmied et al., 2017; Scope et al., 2017; Smith et al., 2019
**1.2.2 They won't be interested**	Bina, 2020; Hadfield & Wittkowski, 2017
**1.3 Beliefs about mental illness**	
**1.3.1 Not knowing what it is**	Bina, 2020; Button et al., 2017; Dennis & Chung-Lee, 2006; Hadfield & Wittkowski, 2017; Hansotte et al., 2017; Jones, 2019; Lucas et al., 2019; Megnin-Viggars et al., 2015; Morrell et al., 2016; Newman et al., 2019; Schmied et al., 2017; Scope et al., 2017; Smith et al., 2019; Staneva et al., 2015; Tobin et al., 2018; Watson et al., 2019
**1.3.1.1. Not having the language to describe perinatal mental illness**	Brealey et al., 2010; Staneva et al., 2015; Tobin et al., 2018; Watson et al., 2019
**1.3.2 Causes**	
**1.3.2.1 Cultural/spiritual**	Schmied et al., 2017; Wittkowski et al., 2014
**1.3.2.2 External factors**	Bina, 2020; Button et al., 2017; Dennis & Chung-Lee, 2006; Lucas et al., 2019; Schmied et al., 2017; Staneva et al., 2015; Tobin et al., 2018; Watson et al., 2019
**1.3.2.3 Physical factors**	Bina, 2020; Button et al., 2017; Dennis & Chung-Lee, 2006; Forde et al., 2020; Jones et al., 2014; Newman et al., 2019; Schmied et al., 2017; Smith et al., 2019; Staneva et al., 2015; Watson et al., 2019
**1.3.2.4 A normal response to motherhood?**	Dennis & Chung-Lee, 2006; Giscombe et al., 2020; Jones et al., 2014; Schmied et al., 2017; Slade et al., 2020; Smith et al., 2019; Sorsa et al., 2021
**1.3.3 How to deal with symptoms**	
**1.3.3.1 Ignore them**	Bina, 2020; Hadfield & Wittkowski, 2017; Jones, 2019; Newman et al., 2019; Schmied et al., 2017; Slade et al., 2020
**1.3.3.2 Seek spiritual guidance**	Hansotte et al., 2017; Kassam, 2019; Schmied et al., 2017; Watson et al., 2019
**1.4 Deciding to seek help**	

For peer review only - http://bmjopen.bmj.com/site/about/guidelines.xhtml

**1.4.1 Recognising something is wrong**	Bina, 2020; Button et al., 2017; Forde et al., 2020; Hadfield & Wittkowski, 2017; Hansotte et al., 2017; Slade et al., 2020; Sorsa et al., 2021; Staneva et al., 2015; Viveiros & Darling, 2018
**1.4.2 Where do I go to seek help?**	Bina, 2020; Dennis & Chung-Lee, 2006; Hansotte et al., 2017; Jones, 2019; Megnin-Viggars et al., 2015; Schmied et al., 2017; Smith et al., 2019; Sorsa et al., 2021; Tobin et al., 2018
**1.5 Fear of judgement**	
**1.5.1 Fear of being seen as a bad mum**	Bina, 2020; Brealey et al., 2010; Button et al., 2017; Forde et al., 2020; Jones et al., 2014; Lucas et al., 2019; Schmied et al., 2017; Slade et al., 2020; Sorsa et al., 2021; Viveiros & Darling, 2018; Watson et al., 2019
**1.5.2 Social services/removal of child**	Bina, 2020; Brealey et al., 2010; Button et al., 2017; Dennis & Chung-Lee, 2006; Evans et al., 2020; Forde et al., 2020; Hadfield & Wittkowski, 2017; Hewitt et al., 2009; Jones, 2019; Megnin-Viggars et al., 2015; Newman et al., 2019; Tobin et al., 2018; Watson et al., 2019
**1.5.3 Symptom minimisation**	Bina, 2020; Dennis & Chung-Lee, 2006; Forde et al., 2020; Hewitt et al., 2009; Holopainen & Hakulinen, 2019; Jones et al., 2014; Kassam, 2019; Lucas et al., 2019; Megnin-Viggars et al., 2015; Schmied et al., 2017; Staneva et al., 2015; Watson et al., 2019
**1.6 Logistics**	
**1.6.1 Childcare**	Bina, 2020; Button et al., 2017; Dennis & Chung-Lee, 2006; Hansotte et al., 2017; Morrell et al., 2016; Newman et al., 2019; Scope et al., 2017; Smith et al., 2019; Tobin et al., 2018; Watson et al., 2019
**1.6.2 Timing**	Bina, 2020; Dennis & Chung-Lee, 2006; Newman et al., 2019; Scope et al., 2017; Watson et al., 2019
**1.6.3 Location/travel**	Bina, 2020; Hansotte et al., 2017; Jones, 2019; Mollard et al., 2016; Morrell et al., 2016; Newman et al., 2019; Schmied et al., 2017; Sorsa et al., 2021; Tobin et al., 2018; Watson et al., 2019
**1.7 Social and family life**	
**1.7.1 Social isolation/support**	Bina, 2020; Giscombe et al., 2020; Holopainen & Hakulinen, 2019; Jones, 2019; Jones et al., 2014; Kassam, 2019; Lucas et al., 2019; Tobin et al., 2018; Viveiros & Darling, 2018
**1.7.1. 1 Exacerbated by mental illness**	Holopainen & Hakulinen, 2019; Jones et al., 2014; Watson et al., 2019
**1.7.2 Family and friends**	Bina, 2020; Button et al., 2017; Dennis & Chung-Lee, 2006; Forde et al., 2020; Hadfield & Wittkowski, 2017; Hansotte et al., 2017; Holopainen & Hakulinen, 2019; Jones, 2019; Lucas et al., 2019; Nilaweera et al., 2014; Schmied et al., 2017; Smith et al., 2019; Sorsa et al., 2021; Viveiros & Darling, 2018; Watson et al., 2019
**1.8 Sociodemographic factors**	
**1.8.1 Ethnicity**	Bina, 2020; Dennis & Chung-Lee, 2006; Hansotte et al., 2017; Watson et al., 2019
**1.8.2 Age**	Bina, 2020; Hansotte et al., 2017
**1.8.3 Previous experiences**	Button et al., 2017; Evans et al., 2020; Hadfield & Wittkowski, 2017; Hansotte et al., 2017; Jones, 2019; Sorsa et al., 2021; Watson et al., 2019
**1.8.4 Previous Diagnoses/symptoms**	Bina, 2020; Viveiros & Darling, 2018

For peer review only - http://bmjopen.bmj.com/site/about/guidelines.xhtml

**2. HCP**	
**2.1 HCP being dismissive or normalising symptoms**	Button et al., 2017; Dennis & Chung-Lee, 2006; Forde et al., 2020; Hadfield & Wittkowski, 2017; Hansotte et al., 2017; Megnin-Viggars et al., 2015; Newman et al., 2019; Sorsa et al., 2021; Watson et al., 2019
**2.2 HCP not recognising help seeking**	Bina, 2020; Button et al., 2017; Megnin-Viggars et al., 2015; Tobin et al., 2018; Watson et al., 2019
**2.3 HCP appearing too busy**	Bina, 2020; Button et al., 2017; Dennis & Chung-Lee, 2006; Hewitt et al., 2009; Megnin-Viggars et al., 2015; Slade et al., 2020; Viveiros & Darling, 2018; Watson et al., 2019
**2.3 Women's perceptions of HCPs knowledge**	
**2.3.1 Perception of HCP knowledge about PNMI**	Dennis & Chung-Lee, 2006; Forde et al., 2020; Hansotte et al., 2017; Jones, 2019; Megnin-Viggars et al., 2015; Morrell et al., 2016
**2.3.2 Perception of HCP knowledge about services/referral pathways**	Dennis & Chung-Lee, 2006; Smith et al., 2019; Viveiros & Darling, 2018
**2.4 The way the HCP delivers the care**	Button et al., 2017; Forde et al., 2020; Kassam, 2019; Megnin-Viggars et al., 2015; Nilaweera et al., 2014; Schmied et al., 2017; Slade et al., 2020; Smith et al., 2019; Sorsa et al., 2021
**2.5 HCP characteristics**	Brealey et al., 2010; Button et al., 2017; Dennis & Chung-Lee, 2006; Forde et al., 2020; Hadfield & Wittkowski, 2017; Hewitt et al., 2009; Megnin-Viggars et al., 2015; Morrell et al., 2016; Newman et al., 2019; Schmied et al., 2017; Slade et al., 2020; Staneva et al., 2015; Viveiros & Darling, 2018
**3. Interpersonal**	
**3.1 Relationship and rapport**	Bina, 2020; Brealey et al., 2010; Dennis & Chung-Lee, 2006; Hadfield & Wittkowski, 2017; Hewitt et al., 2009; Megnin-Viggars et al., 2015; Morrell et al., 2016; Scope et al., 2017; Slade et al., 2020; Tobin et al., 2018
**3.2 Language barriers**	Button et al., 2017; Dennis & Chung-Lee, 2006; Hansotte et al., 2017; Megnin-Viggars et al., 2015; Schmied et al., 2017; Smith et al., 2019
**3.3 Shared decision making**	Bina, 2020; Hadfield & Wittkowski, 2017; Megnin-Viggars et al., 2015; Morrell et al., 2016; Randall & Briscoe, 2018; Scope et al., 2017
**3.4 Communication**	Brealey et al., 2010; Forde et al., 2020; Hadfield & Wittkowski, 2017; Hewitt et al., 2009
**3.5 Information provision**	Bina, 2020; Button et al., 2017; Megnin-Viggars et al., 2015; Randall & Briscoe, 2018; Slade et al., 2020; Smith et al., 2019; Sorsa et al., 2021; Tobin et al., 2018
**4. Organisational**	
**4.1 Lack of services/Overstretched**	Bina, 2020; Button et al., 2017; Forde et al., 2020; Jones, 2019; Megnin-Viggars et al., 2015; Smith et al., 2019; Tobin et al., 2018; Viveiros & Darling, 2018
**4.2 Characteristics of service**	Bina, 2020; Button et al., 2017; Dennis & Chung-Lee, 2006; Forde et al., 2020; Jones, 2019; Newman et al., 2019; Scope et al., 2017; Viveiros & Darling, 2018; Watson et al., 2019

For peer review only - http://bmjopen.bmj.com/site/about/guidelines.xhtml

**4.3 Collaboration within and across services**	Bina, 2020; Megnin-Viggars et al., 2015; Newman et al., 2019; Smith et al., 2019; Watson et al., 2019
**4.4 Continuity of carer**	Brealey et al., 2010; Button et al., 2017; Hadfield & Wittkowski, 2017; Megnin-Viggars et al., 2015; Slade et al., 2020; Smith et al., 2019; Tobin et al., 2018; Viveiros & Darling, 2018; Watson et al., 2019
**4.5 Ideal care**	
**4.5.1 Screening**	
**4.5.1.1 Screening acceptability**	Brealey et al., 2010; Button et al., 2017; Evans et al., 2020; Hewitt et al., 2009; Megnin-Viggars et al., 2015; Mollard et al., 2016; Smith et al., 2019
**4.5.1.2 Wording/contents**	Brealey et al., 2010; Hewitt et al., 2009
**4.5.1.3 Delivery**	Brealey et al., 2010; Button et al., 2017; Evans et al., 2020; Hewitt et al., 2009; Smith et al., 2019
**4.5.2 Optimal treatment**	
**4.5.2.1 Opportunity to talk**	Dennis & Chung-Lee, 2006; Evans et al., 2020; Hadfield & Wittkowski, 2017; Jones, 2019; Kassam, 2019; Morrell et al., 2016; Praetorius et al., 2020; Staneva et al., 2015
**4.5.2.2 Location**	Hadfield & Wittkowski, 2017; Hansotte et al., 2017; Jones, 2019; Jones et al., 2014; Mollard et al., 2016; Newman et al., 2019; Praetorius et al., 2020; Sorsa et al., 2021
**4.5.2.3 Appropriate**	Evans et al., 2020; Megnin-Viggars et al., 2015; Scope et al., 2017; Sorsa et al., 2021
**4.5.2.4 Individualised**	Evans et al., 2020; Hadfield & Wittkowski, 2017; Megnin-Viggars et al., 2015; Morrell et al., 2016; Schmied et al., 2017; Scope et al., 2017; Slade et al., 2020; Sorsa et al., 2021; Viveiros & Darling, 2018; Watson et al., 2019
**4.5.2.5 Length**	Hadfield & Wittkowski, 2017; Morrell et al., 2016; Schmied et al., 2017; Watson et al., 2019
**4.5.2.6 Group/Peer support**	Dennis & Chung-Lee, 2006; Evans et al., 2020; Forde et al., 2020; Hadfield & Wittkowski, 2017; Jones et al., 2014; Megnin-Viggars et al., 2015; Morrell et al., 2016; Schmied et al., 2017; Scope et al., 2017; Slade et al., 2020; Tobin et al., 2018; Watson et al., 2019
**4.5.2.6.1 Validation provided by peer support**	Jones et al., 2014; Morrell et al., 2016; Schmied et al., 2017; Slade et al., 2020
**4.5.2.7 Culturally appropriate**	Button et al., 2017; Dennis & Chung-Lee, 2006; Giscombe et al., 2020; Hadfield & Wittkowski, 2017; Jones, 2019; Schmied et al., 2017; Smith et al., 2019; Tobin et al., 2018; Watson et al., 2019
**4.5.2.8 Information provision**	Forde et al., 2020; Hadfield & Wittkowski, 2017; A. Jones, 2019; Megnin-Viggars et al., 2015; Morrell et al., 2016; Schmied et al., 2017; Scope et al., 2017; Slade et al., 2020; Sorsa et al., 2021
**4.5.2.9 Medication**	Dennis & Chung-Lee, 2006; Forde et al., 2020; Jones, 2019; Sorsa et al., 2021; Tobin et al., 2018
**5. Political**	
**5.1 Immigration status**	Bina, 2020; Giscombe et al., 2020; Hansotte et al., 2017; Kassam, 2019; Lucas et al., 2019; Schmied et al., 2017

For peer review only - http://bmjopen.bmj.com/site/about/guidelines.xhtml

**5.2. Economic status**	Schmied et al., 2017; Tobin et al., 2018; Watson et al., 2019
**5.2.1 Healthcare costs**	Bina, 2020; Hansotte et al., 2017; Schmied et al., 2017; Tobin et al., 2018; Viveiros & Darling, 2018
**6. Societal**	
**6.1. Culture**	Bina, 2020; Brealey et al., 2010; Button et al., 2017; Dennis & Chung-Lee, 2006; Giscombe et al., 2020; Hansotte et al., 2017; Hewitt et al., 2009; Jones, 2019; Kassam, 2019; Megnin-Viggars et al., 2015; Nilaweera et al., 2014; Praetorius et al., 2020; Schmied et al., 2017; Smith et al., 2019; Sorsa et al., 2021; Staneva et al., 2015; Tobin et al., 2018; Viveiros & Darling, 2018; Watson et al., 2019; Wittkowski et al., 2014
**6.2. Maternal norms**	Bina, 2020; Brealey et al., 2010; Button et al., 2017; Dennis & Chung-Lee, 2006; Forde et al., 2020; Hadfield & Wittkowski, 2017; Hansotte et al., 2017; Hewitt et al., 2009; Holopainen & Hakulinen, 2019; Jones et al., 2014; Lucas et al., 2019; Mollard et al., 2016; Morrell et al., 2016; Newman et al., 2019; Schmied et al., 2017; Slade et al., 2020; Smith et al., 2019; Sorsa et al., 2021; Staneva et al., 2015; Viveiros & Darling, 2018
**6.3. Stigma**	Bina, 2020; Button et al., 2017; Dennis & Chung-Lee, 2006; Forde et al., 2020; Giscombe et al., 2020; Hadfield & Wittkowski, 2017; Hansotte et al., 2017; Hewitt et al., 2009; Holopainen & Hakulinen, 2019; Jones, 2019; Kassam, 2019; Lucas et al., 2019; Megnin-Viggars et al., 2015; Morrell et al., 2016; Nilaweera et al., 2014; Scope et al., 2017; Smith et al., 2019; Sorsa et al., 2021; Staneva et al., 2015; Tobin et al., 2018; Viveiros & Darling, 2018; Watson et al., 2019

*Note: highlighted yellow are themes only represented by lower-quality studies

For peer review only

For peer review only - http://bmjopen.bmj.com/site/about/guidelines.xhtml

References

1. Curry SJ, Krist AH, Owens DK, et al. Interventions to Prevent Perinatal Depression: US Preventive Services Task Force Recommendation Statement. *JAMA*. 2019;321(6):580-587. doi:10.1001/JAMA.2019.0007

2. What is the CPMHC? – CANADIAN PERINATAL MENTAL HEALTH COLLABORATIVE / COLLECTIF CANADIEN POUR LA SANTÉ MENTALE PÉRINATALE. Accessed April 12, 2022. https://cpmhc.ca/what-is-the-cpmhc/

3. PMHP - Perinatal Mental Health Project. Accessed April 12, 2022. https://pmhp.za.org/

4. Perinatal Anxiety & Depression Australia (PANDA). *Submission to the Productivity Commission Mental Health Inquiry: The Social and Economic Benefits of Improving Perinatal Mental Health.*; 2019.

5. National Institute for Health and Care Excellence (NICE). *Antenatal and Postnatal Mental Health Clinical Management and Service*.; 2014.

6. Moher D, Liberati A, Tetzlaff JM, Altman DG. Preferred reporting items for systematic reviews and meta-analyses: the PRISMA statement. *PLoS Med*. 2009;6(7):e1000097. doi:10.1016/j.jclinepi.2009.06.005

7. The Cochrane Collaboration. Cochrane data collection form for intervention reviews for RCTs only. *The cochrane Collaboration*. 2014;(April):2014. http://www.cochrane.org/

8. Shea BJ, Reeves BC, Wells GA, et al. AMSTAR 2: A critical appraisal tool for systematic reviews that include randomised or non-randomised studies of healthcare interventions, or both. *BMJ*. 2017;21:358. doi:10.1136/bmj.j4008

9. Harden A. *"Qualitative" Research, Systematic Reviews, and Evidence-Informed Policy and Practice*. University of London; 2007.

10. Carroll C, Booth A, Lloyd-Jones M. Should we exclude inadequately reported studies from qualitative systematic reviews? An evaluation of sensitivity analyses in two case study reviews. *Qualitative Health Research*. 2012;22(10):1425-1434.

11. Bina R. Predictors of postpartum depression service use: A theory-informed, integrative systematic review. *Women and Birth*. 2020;33(1):e24-e32. doi:10.1016/j.wombi.2019.01.006

12. Hansotte E, Payne SI, Babich SM. Positive postpartum depression screening practices and subsequent mental health treatment for low-income women in Western countries: A systematic literature review. *Public Health Reviews*. 2017;38(1):1-17. doi:10.1186/s40985-017-0050-y

13. Geertz C. *The Interpretation of Cultures: Selected Essays*. Basic Books; 1973.

14. Hadfield H, Wittkowski A. Women's Experiences of Seeking and Receiving Psychological and Psychosocial Interventions for Postpartum Depression: A Systematic Review and Thematic Synthesis of the Qualitative Literature. *Journal of Midwifery and Women's Health*. 2017;62(6):723-736. doi:10.1111/jmwh.12669

15. Popay J, Rogers A, Williams G. Rationale and standards for the systematic review of qualitative literature in health services research. *Qualitative Health Research*. 1998;8(3):341-351. doi:10.1177/104973239800800305

For peer review only - http://bmjopen.bmj.com/site/about/guidelines.xhtml

16. Thomas J, Harden A. Methods for the thematic synthesis of qualitative research in systematic reviews. *BMC Medical Research Methodology*. 2008;8(1):1-10. doi:10.1186/1471-2288-8-45

17. Ferlie EB, Shortell SM. Improving the Quality of Health Care in the United Kingdom and the United States: A Framework for Change. *Milbank Quarterly*. 2001;79(2):281-315. doi:10.1111/1468-0009.00206

18. Brealey SD, Hewitt C, Green JM, Morrell J, Gilbody S. Screening for postnatal depression - is it acceptable to women and healthcare professionals? A systematic review and meta-synthesis. *Journal of Reproductive and Infant Psychology*. 2010;28(4):328-344. doi:10.1080/02646838.2010.513045

19. Button S, Thornton A, Lee S, Ayers S, Shakespeare J. Seeking help for perinatal psychological distress: A meta-synthesis of women's experiences. *British Journal of General Practice*. 2017;67(663):e692-e699. doi:10.3399/bjgp17X692549

20. Dennis CL, Chung-Lee L. Postpartum depression help-seeking barriers and maternal treatment preferences: A qualitative systematic review. *Birth*. 2006;33(4):323-331. doi:10.1111/j.1523-536X.2006.00130.x

21. Evans K, Spiby H, Morrell J. Non-pharmacological interventions to reduce the symptoms of mild to moderate anxiety in pregnant women. A systematic review and narrative synthesis of women's views on the acceptability of and satisfaction with interventions. *Archives of Women's Mental Health*. 2020;23:11-28. doi:10.1007/s00737-018-0936-9

22. Giscombe T, Hui A, Stickley T. Perinatal mental health amongst refugee and asylum-seeking women in the UK. *Mental Health Review Journal*. 2020;25(3).

23. Hewitt CE, Gilbody S, Brealey SD, et al. Methods to identify postnatal depression in primary care: An integrated evidence synthesis and value of information analysis. *Health Technology Assessment*. 2009;13(36):1-145. doi:10.3310/hta13360

24. Holopainen A, Hakulinen T. New parents' experiences of postpartum depression: A systematic review of qualitative evidence. *JBI Database of Systematic Reviews and Implementation Reports*. 2019;17(9):1731-1769. doi:10.11124/JBISRIR-2017-003909

25. Jones CCG, Jomeen J, Hayter M. The impact of peer support in the context of perinatal mental illness: A meta-ethnography. *Midwifery*. 2014;30(5):491-498. doi:10.1016/j.midw.2013.08.003

26. Jones A. Help seeking in the perinatal period: a review of barriers and facilitators. *Soc Work Public Health*. 2019;34(7):596-605.

27. Kassam S. Understanding Experiences of Social Support as Coping Resources among Immigrant and Refugee Women with Postpartum Depression: An Integrative Literature Review. *Issues in Mental Health Nursing*. 2019;40(12):999-1011. doi:10.1080/01612840.2019.1585493

28. Lucas G, Olander EK, Ayers S, Salmon D. No straight lines - Young women's perceptions of their mental health and wellbeing during and after pregnancy: A systematic review and meta-ethnography. *BMC Women's Health*. 2019;19(1):1-17. doi:10.1186/s12905-019-0848-5

29. Megnin-Viggars O, Symington I, Howard LM, Pilling S. Experience of care for mental health problems in the antenatal or postnatal period for women in the UK: a systematic review and

For peer review only - http://bmjopen.bmj.com/site/about/guidelines.xhtml

meta-synthesis of qualitative research. *Archives of Women's Mental Health*. 2015;18(6):245-759. doi:10.1007/s00737-015-0548-6

30. Mollard E, Hudson DB, Ford A, Pullen C. An Integrative Review of Postpartum Depression in Rural U.S. Communities. *Archives of Psychiatric Nursing*. 2016;30(3):418-424. doi:10.1016/j.apnu.2015.12.003

31. Morrell J, Sutcliffe P, Booth A, et al. A systematic review, evidence synthesis and meta-analysis of quantitative and qualitative studies evaluating the clinical effectiveness, the cost-effectiveness, safety and acceptability of interventions to prevent postnatal depression. *Health Technology Assessment*. 2016;20(37). doi:10.3310/hta20370

32. Newman TC, Hirst J, Darwin Z. What enables or prevents women with depressive symptoms seeking help in the postnatal period? *British Journal of Midwifery*. 2019;27(4):219-227. doi:10.12968/bjom.2019.27.4.219

33. Nilaweera I, Doran F, Fisher J. Prevalence, nature and determinants of postpartum mental health problems among women who have migrated from South Asian to high-income countries: A systematic review of the evidence. *Journal of Affective Disorders*. 2014;166:213-226. doi:10.1016/j.jad.2014.05.021

34. Praetorius R, Maxwell D, Alam K. Wearing a happy mask: mother's expressions of suicidality with postpartum depression. *Social Work in Mental Health*. 2020;18(4):429-459.

35. Randall L, Briscoe L. Are women empowered to make decisions about the use of antidepressants in pregnancy? *British Journal of Midwifery*. 2018;26(5):329-335. doi:10.12968/bjom.2018.26.5.329

36. Sambrook Smith M, Lawrence V, Sadler E, Easter A. Barriers to accessing mental health services for women with perinatal mental illness: Systematic review and meta-synthesis of qualitative studies in the UK. *BMJ Open*. 2019;9(1):e024803. doi:10.1136/bmjopen-2018-024803

37. Schmied V, Black E, Naidoo N, Dahlen HG, Liamputtong P. Migrant women's experiences, meanings and ways of dealing with postnatal depression: A meta-ethnographic study. *PLoS ONE*. 2017;12(3). doi:10.1371/journal.pone.0172385

38. Scope A, Booth A, Morrell J, Sutcliffe P, Cantrell A. Perceptions and experiences of interventions to prevent postnatal depression. A systematic review and qualitative evidence synthesis. *Journal of Affective Disorders*. 2017;210:100-110. doi:10.1016/j.jad.2016.12.017

39. Slade P, Molyneux R, Watt A. A systematic review of clinical effectiveness of psychological interventions to reduce post traumatic stress symptoms following childbirth and a meta-synthesis of facilitators and barriers to uptake of psychological care. *J Affect Disord*. 2020;281:678-694.

40. Sorsa MA, Kylmä J, Bondas TE. Contemplating Help-Seeking in Perinatal Psychological Distress—A Meta-Ethnography. *Int J Environ Res Public Health*. 2021;18(10):5226.

41. Staneva AA, Bogossian F, Wittkowski A. The experience of psychological distress, depression, and anxiety during pregnancy: A meta-synthesis of qualitative research. *Midwifery*. 2015;31(6):563-573. doi:10.1016/j.midw.2015.03.015

For peer review only - http://bmjopen.bmj.com/site/about/guidelines.xhtml

42. Tobin CL, di Napoli P, Beck CT. Refugee and Immigrant Women's Experience of Postpartum Depression: A Meta-Synthesis. *J Transcult Nurs*. 2018;29(1):84-100. doi:10.1177/1043659616686167

43. Viveiros CJ, Darling EK. Perceptions of barriers to accessing perinatal mental health care in midwifery: A scoping review. *Midwifery*. 2019;70:106-118. doi:10.1016/j.midw.2018.11.011

44. Watson H, Harrop D, Walton E, Young A, Soltani H. A systematic review of ethnic minority women's experiences of perinatal mental health conditions and services in Europe. *PLoS ONE*. 2019;14(1):e0210587. doi:10.1371/journal.pone.0210587

45. Wittkowski A, Gardner PL, Bunton P, Edge D. Culturally determined risk factors for postnatal depression in Sub-Saharan Africa: A mixed method systematic review. *Journal of Affective Disorders*. 2014;163:115-124. doi:10.1016/j.jad.2013.12.028

46. Forde R, Peters S, Wittkowski A. Recovery from postpartum psychosis: a systematic review and metasynthesis of women's and families' experiences. *Archives of Women's Mental Health*. 2020;23(5):597-612. doi:10.1007/s00737-020-01025-z

For peer review only

For peer review only - http://bmjopen.bmj.com/site/about/guidelines.xhtml

PRISMA 2020 Checklist

Section and Topic	Item #	Checklist item	Location where item is reported
**TITLE**			
Title	1	Identify the report as a systematic review.	1
**ABSTRACT**			
Abstract	2	See the PRISMA 2020 for Abstracts checklist.	3
**INTRODUCTION**			
Rationale	3	Describe the rationale for the review in the context of existing knowledge.	5
Objectives	4	Provide an explicit statement of the objective(s) or question(s) the review addresses.	5
**METHODS**			
Eligibility criteria	5	Specify the inclusion and exclusion criteria for the review and how studies were grouped for the syntheses.	6 & Appendix
Information sources	6	Specify all databases, registers, websites, organisations, reference lists and other sources searched or consulted to identify studies. Specify the date when each source was last searched or consulted.	6 & Appendix
Search strategy	7	Present the full search strategies for all databases, registers and websites, including any filters and limits used.	Appendix
Selection process	8	Specify the methods used to decide whether a study met the inclusion criteria of the review, including how many reviewers screened each record and each report retrieved, whether they worked independently, and if applicable, details of automation tools used in the process.	6 & Appendix
Data collection process	9	Specify the methods used to collect data from reports, including how many reviewers collected data from each report, whether they worked independently, any processes for obtaining or confirming data from study investigators, and if applicable, details of automation tools used in the process.	6 & Appendix
Data items	10a	List and define all outcomes for which data were sought. Specify whether all results that were compatible with each outcome domain in each study were sought (e.g. for all measures, time points, analyses), and if not, the methods used to decide which results to collect.	6 & Appendix
	10b	List and define all other variables for which data were sought (e.g. participant and intervention characteristics, funding sources). Describe any assumptions made about any missing or unclear information.	6 & Appendix
Study risk of bias assessment	11	Specify the methods used to assess risk of bias in the included studies, including details of the tool(s) used, how many reviewers assessed each study and whether they worked independently, and if applicable, details of automation tools used in the process.	6-7 & Appendix
Effect measures	12	Specify for each outcome the effect measure(s) (e.g. risk ratio, mean difference) used in the synthesis or presentation of results.	N/A
Synthesis methods	13a	Describe the processes used to decide which studies were eligible for each synthesis (e.g. tabulating the study intervention characteristics and comparing against the planned groups for each synthesis (item #5)).	7
	13b	Describe any methods required to prepare the data for presentation or synthesis, such as handling of missing summary statistics, or data conversions.	7
	13c	Describe any methods used to tabulate or visually display results of individual studies and syntheses.	7
	13d	Describe any methods used to synthesize results and provide a rationale for the choice(s). If meta-analysis was performed, describe the model(s), method(s) to identify the presence and extent of statistical heterogeneity, and software package(s) used.	7
	13e	Describe any methods used to explore possible causes of heterogeneity among study results (e.g. subgroup analysis, meta-regression).	N/A
	13f	Describe any sensitivity analyses conducted to assess robustness of the synthesized results.	7 & Appendix
Reporting bias assessment	14	Describe any methods used to assess risk of bias due to missing results in a synthesis (arising from reporting biases).	7 & Appendix

For peer review only - http://bmjopen.bmj.com/site/about/guidelines.xhtml

PRISMA 2020 Checklist

Section and Topic	Item #	Checklist item	Location where item is reported
Certainty assessment	15	Describe any methods used to assess certainty (or confidence) in the body of evidence for an outcome.	7 & Appendix
**RESULTS**			
Study selection	16a	Describe the results of the search and selection process, from the number of records identified in the search to the number of studies included in the review, ideally using a flow diagram.	Figure 1
	16b	Cite studies that might appear to meet the inclusion criteria, but which were excluded, and explain why they were excluded.	Figure 1
Study characteristics	17	Cite each included study and present its characteristics.	7 & Appendix
Risk of bias in studies	18	Present assessments of risk of bias for each included study.	Appendix
Results of individual studies	19	For all outcomes, present, for each study: (a) summary statistics for each group (where appropriate) and (b) an effect estimate and its precision (e.g. confidence/credible interval), ideally using structured tables or plots.	N/A
Results of syntheses	20a	For each synthesis, briefly summarise the characteristics and risk of bias among contributing studies.	Appendix
	20b	Present results of all statistical syntheses conducted. If meta-analysis was done, present for each the summary estimate and its precision (e.g. confidence/credible interval) and measures of statistical heterogeneity. If comparing groups, describe the direction of the effect.	N/A
	20c	Present results of all investigations of possible causes of heterogeneity among study results.	N/A
	20d	Present results of all sensitivity analyses conducted to assess the robustness of the synthesized results.	Appendix
Reporting biases	21	Present assessments of risk of bias due to missing results (arising from reporting biases) for each synthesis assessed.	N/A
Certainty of evidence	22	Present assessments of certainty (or confidence) in the body of evidence for each outcome assessed.	Appendix
**DISCUSSION**			
Discussion	23a	Provide a general interpretation of the results in the context of other evidence.	18-21
	23b	Discuss any limitations of the evidence included in the review.	21
	23c	Discuss any limitations of the review processes used.	21
	23d	Discuss implications of the results for practice, policy, and future research.	21
**OTHER INFORMATION**			
Registration and protocol	24a	Provide registration information for the review, including register name and registration number, or state that the review was not registered.	4
	24b	Indicate where the review protocol can be accessed, or state that a protocol was not prepared.	4
	24c	Describe and explain any amendments to information provided at registration or in the protocol.	N/A
Support	25	Describe sources of financial or non-financial support for the review, and the role of the funders or sponsors in the review.	4
Competing interests	26	Declare any competing interests of review authors.	N/A
Availability of data, code and other materials	27	Report which of the following are publicly available and where they can be found: template data collection forms; data extracted from included studies; data used for all analyses; analytic code; any other materials used in the review.	Appendix

For peer review only

PRISMA 2020 Checklist

From: Page MJ, McKenzie JE, Bossuyt PM, Boutron I, Hoffmann TC, Mulrow CD, et al. The PRISMA 2020 statement: an updated guideline for reporting systematic reviews. BMJ 2021;372:n71. doi: 10.1136/bmj.n71

For more information, visit: http://www.prisma-statement.org/

For peer review only

BMJ Open

A meta-review of the barriers and facilitators to women accessing perinatal mental health care

Journal:	*BMJ Open*
Manuscript ID	bmjopen-2022-066703.R2
Article Type:	Original research
Date Submitted by the Author:	27-Jun-2023
Complete List of Authors:	Webb, Rebecca; City University of London, Centre for Maternal and Child Health Research<br>Uddin, Nazihah; City University of London, Centre for Maternal and Child Health Research<br>Constantinou, Georgie; City University of London, Centre for Maternal and Child Health Research<br>Ford, Elizabeth; Brighton and Sussex Medical School<br>Easter, Abigail; King's College London, Department of Women and Children's Health, School of Life Course Sciences, ; King's College London, Section of Women's Mental Health, Institute of Psychiatry, Psychology & Neuroscience<br>Shakespeare, Judy; Retired,<br>Hann, Agnes; National Childbirth Trust<br>Roberts, Nia; University of Oxford, Bodleian Health Care Libraries,<br>Alderdice, Fiona; National Perinatal Epidemiology Unit, Nuffield Department of Population Health<br>Sinesi, Andrea; University of Stirling, Nursing, Midwifery and Allied Health Professions Research Unit (NMAHP RU)<br>Coates, Rose ; City University of London, Centre for Maternal and Child Health Research<br>Hogg, Sally; University of Cambridge, Faculty of Education<br>Ayers, Susan; City University, Centre for Maternal and Child Health Research<br>Study Team, The MATRIx; City University of London, Centre for Maternal and Child Health Research
**Primary Subject Heading**:	Mental health
Secondary Subject Heading:	Evidence based practice, Obstetrics and gynaecology, Public health
Keywords:	PSYCHIATRY, Health policy < HEALTH SERVICES ADMINISTRATION & MANAGEMENT, Depression & mood disorders < PSYCHIATRY, Anxiety disorders < PSYCHIATRY

SCHOLARONE™
Manuscripts

I, the Submitting Author has the right to grant and does grant on behalf of all authors of the Work (as defined in the below author licence), an exclusive licence and/or a non-exclusive licence for contributions from authors who are: i) UK Crown employees; ii) where BMJ has agreed a CC-BY licence shall apply, and/or iii) in accordance with the terms applicable for US Federal Government officers or employees acting as part of their official duties; on a worldwide, perpetual, irrevocable, royalty-free basis to BMJ Publishing Group Ltd ("BMJ") its licensees and where the relevant Journal is co-owned by BMJ to the co-owners of the Journal, to publish the Work in this journal and any other BMJ products and to exploit all rights, as set out in our licence.

The Submitting Author accepts and understands that any supply made under these terms is made by BMJ to the Submitting Author unless you are acting as an employee on behalf of your employer or a postgraduate student of an affiliated institution which is paying any applicable article publishing charge ("APC") for Open Access articles. Where the Submitting Author wishes to make the Work available on an Open Access basis (and intends to pay the relevant APC), the terms of reuse of such Open Access shall be governed by a Creative Commons licence – details of these licences and which Creative Commons licence will apply to this Work are set out in our licence referred to above.

Other than as permitted in any relevant BMJ Author's Self Archiving Policies, I confirm this Work has not been accepted for publication elsewhere, is not being considered for publication elsewhere and does not duplicate material already published. I confirm all authors consent to publication of this Work and authorise the granting of this licence.

For peer review only

A meta-review of the barriers and facilitators to women accessing perinatal mental health care

Rebecca Webb, Nazihah Uddin, Georgina Constantinou, Elizabeth Ford, Abigail Easter, Judy Shakespeare, Agnes Hann, Nia Roberts, Fiona Alderdice, Andrea Sinesi, Rose Coates, Sally Hogg, Susan Ayers, and the MATRIx Study Team

Rebecca Webb (PhD) Centre for Maternal and Child Health, City, University of London, EC1V 0HB, United Kingdom

Nazihah Uddin (MSc) Centre for Maternal and Child Health, City, University of London, EC1V 0HB, United Kingdom

Georgina Constantinou (PhD) Centre for Maternal and Child Health, City, University of London, EC1V 0HB, United Kingdom

Elizabeth Ford (PhD) Brighton & Sussex Medical School, Village Way, Falmer, BN1 9PH, United Kingdom

Abigail Easter (PhD) Department of Women and Children's Health, School of Life Course Sciences, King's College London, London, SE5 8AB, United Kingdom

Judy Shakespeare (BM, BCh) Retired GP, Oxford, OX2 7AG.

Agnes Hann (PhD) NCT, Brunel House, 11 The Promenade, Clifton Down, Bristol BS8 3NG

Nia Roberts (PhD) Nuffield Department of Population Health, Bodleian Health Care Libraries, Oxford, OX3 9DU

Fiona Alderdice (Professor, PhD) National Perinatal Epidemiology Unit, Nuffield Department of Population Health, University of Oxford, Oxford, OX3 7LF

For peer review only

Andrea Sinesi (PhD) Nursing, Midwifery and Allied Health Professions Research Unit (NMAHP RU), University of Stirling, FK9 4LA, United Kingdom

Rose Coates (PhD), Centre for Maternal and Child Health, City, University of London, EC1V 0HB, United Kingdom

Sally Hogg (MA) Faculty of Education, University of Cambridge, 184 Hills Rd, Cambridge, CB2 8PQ

Susan Ayers (Professor, PhD) Professor of Maternal and Child Health, Centre for Maternal and Child Health, City, University of London, EC1V 0HB, United Kingdom

The MATRIx Study Team, Centre for Maternal and Child Health, City, University of London, EC1V 0HB, United Kingdom

*Corresponding Author: Rebecca Webb, Centre for Maternal and Child Health, City, University of London, EC1V 0HB, United Kingdom; Rebecca.Webb.2@city.ac.uk

Acknowledgements: We gratefully acknowledge the contribution of the MATRIx study team: Elaine Clark, Evelyn Frame, Simon Gilbody, Sarah McMullen, Camilla Rosan, Debra Salmon, Clare Thompson, and Louise R Williams.

Word count: 5478

Abstract

Perinatal mental health (PMH) problems are common and can have an adverse impact on women and their families. However, research suggests that a substantial proportion of women with PMH problems do not access care.

Objectives: To synthesise the results from previous systematic reviews of barriers and facilitators to women to seeking help, accessing help, and engaging in PMH care, and to suggest recommendations for clinical practice and policy.

Design: A meta-review of systematic reviews

Review methods: Seven databases were searched and reviewed using a PRISMA (Preferred Reporting Items for Systematic Reviews and Meta Analyses) search strategy. Studies that focused on the views of women seeking help and accessing PMH care were included. Data were analysed using thematic synthesis. Assessing the Methodological Quality of Systematic Reviews (AMSTAR 2) was used to assess review methodology. To improve validity of results, a qualitative sensitivity analysis was conducted to assess whether themes remained consistent across all reviews, regardless of their quality rating.

Results: A total of 32 reviews were included. A wide range of barriers and facilitators to women accessing PMH care were identified. These mapped across a multi-level model of influential factors (individual, healthcare professional, interpersonal, organisational, political, and societal) and across the care pathway (from decision to consult to receiving care). Evidence based recommendations to support the design and delivery of PMH care were produced based on identified barriers and facilitators.

Conclusion: The identified barriers and facilitators point to a complex interplay of many factors, highlighting the need for an international effort to increase awareness of PMH problems, reduce

For peer review only

mental health stigma, and provide woman-centred, flexible care, delivered by well-trained and culturally sensitive primary care, maternity, and psychiatric health professionals.

Funding: NIHR128068

Registration: PROSPERO CRD42019142854

Keywords: Perinatal mental health; Implementation; Mental health services; barriers; facilitators

For peer review only

Strengths and Weaknesses

- This meta-review synthesised a large amount of information from 32 systematic reviews.
- Title and abstracts and full texts were double screened by two reviewers.
- Only reviews published in academic journals and written in English language were included.
- Only 10% of studies had duplicate data extraction.

For peer review only

Introduction

Perinatal mental health (PMH) problems commonly consist of anxiety disorders, depression, post-traumatic stress disorder (PTSD), and stress-related conditions such as adjustment disorder. They can also include more severe difficulties such as postpartum psychosis, and many PMH problems are co-morbid (1,2).

PMH problems can adversely impact women and their families. They are associated with obstetric physical health complications, such as increased risk of pre-eclampsia, antepartum and postpartum haemorrhage, placental abruption, stillbirth (3–5), and pre-term birth (6,7). Furthermore, suicide is a leading cause of death during the perinatal period in higher-income countries (HIC), accounting for 5 to 20% of maternal deaths (2,8,9). Perinatal suicide accounts for between 0.65-3.55% of pregnancy-related deaths in lower-middle income countries (LMIC) (10). Research has also found PMH problems are associated with a child's cognitive, language (11–14), and behavioural development (13,15,16). PMH problems may also mean a woman's child is at an increased risk of developing mental health difficulties themselves (17–19). Furthermore, PMH problems can impact on a woman's relationships with her partner, such as by a decline in relationship satisfaction (20), increased strain on the couple relationship (21,22), and relationship breakdown (23). There is also a large cost to society and healthcare services, with PMH problems costing the UK approximately £8.1 billion every year (24).

Evidence-based PMH care can reduce the negative impacts on women and their families. For example cognitive behavioural therapy (CBT) (25), psychological therapies (26), and certain anti-depressant medications (27) have been shown to be effective in reducing PMH symptoms.

Globally, evidence-based guidelines exist for PMH care. The World Health Organization Millennium Development Goal 5 is to improve maternal health (28), and states that a mental

For peer review only

health component should be incorporated as an integral part of maternal health policies, plans, and activities in all countries (29). However, research suggests access to PMH care is variable (30–33) with only 30-50% of women with PMH problems identified, and less than 10% referred to specialist care (34–36). This variable access could be due to multiple reasons, such as difficulties with implementing PMH services (37), or due to barriers experienced by women.

Multiple systematic reviews have explored barriers and facilitators to women accessing PMH care. Each systematic review varies slightly in relation to its aim and methods making it hard to extract the information needed to design PMH services in a more accessible way. A systematic review of systematic reviews, or a meta-review, is arguably the most suitable way to synthesise results by combining results from multiple reviews into a single body of evidence. This allows for comparison of results from multiple reviews. A meta-review would make it easier for healthcare providers and policy makers to access the information and use it to inform their decisions (38,39). Therefore, the primary aim of this research is to determine the key barriers and facilitators to women deciding to seek help, access help, and engage in PMH care using a meta-review.

Method

The protocol for this review has been registered on PROSPERO (CRD42020193107) (see Appendix 1).

Patient and Public Involvement (PPI)

This project was developed with PPI representatives from the NCT in England, and the Maternal Mental Health Change Agents (MMHCA), a group of women with lived experience of PMH problems in Scotland.

Data sources and searches

For peer review only

Searches were carried out by NR in CINAHL (1982- present); Embase (1974 – present); Medline (1946- present); PsycINFO (1806 – present), Cochrane, SCOPUS, and TRIP (Turning Research into Practice) Medical Database. Searches were completed on 4th August 2021 and forward and backward searches were completed by 8th September 2021. See Appendix 2 & 3 for full search syntax and results.

Study selection

Reviews were included if they used a Preferred Reporting Items for Systematic Reviews and Meta Analyses (PRISMA (40)) search strategy and focused on the views of women seeking help and accessing care for perinatal mental illness. See Appendix 4 for full inclusion criteria. Search results were imported into Endnote and duplicates and papers not meeting initial inclusion criteria (foetal distress, oxidative stress, and non-English papers due to translation times and costs) were removed by NR. The remaining studies were imported into Eppi-Reviewer 4, where results were double screened by title and abstract by two people (RW & GC). Following this, full text screening was carried out by two people (RW & GC).

Data collection process and data items

Data extraction was carried out using Microsoft Excel by RW. Double coding of extracted data was carried out for a proportion of included reviews (n = 3, 10%) by GC.

Critical appraisal of reviews

Methodology sections of included systematic reviews were appraised using the Assessing the Methodological Quality of Systematic Reviews (AMSTAR 2(41)). A decision was made to include reviews where confidence in results was evaluated as low and critically low because these reviews focused more on marginalised women, such as refugees, migrants, women with a low income, and women living in LMICs, to ensure the experiences of these seldom-heard

For peer review only

women were captured. To improve the validity of results, a qualitative sensitivity analysis was carried out to assess whether themes remained consistent across all reviews, regardless of their quality rating (see Appendix 5-8).

Synthesis of results

Results were analysed by RW using a thematic synthesis (42) in NVivo and Microsoft Excel. Themes were mapped onto a multi-level framework adapted from Ferlie and Shortell's Levels of Change framework (individual level, group/team level, organisational level, and larger system/environment level(43) and utilised in a previous systematic review on barriers and facilitators to implementing PMH care, carried out by the review authors (37). The levels identified in the previous review reflect the reviewed literature and the complexities of the health services and are as follows: individual, health professional (HP), interpersonal, organisational, political, and societal. These will be described in more detail below. The mapping of descriptive themes was developed deductively from the initial theoretical framework and then inductively revised as new themes emerged. The mapping of descriptive themes was discussed by all review authors before being finalised. Differences of opinion were resolved through discussion. Recommendations were developed for policy and practice based on the most cited themes. For a more detailed methodology please see Appendix 5.

Results

Review selection and review characteristics

Screening identified 32 reviews to be included in the meta-review (see Figure 1). See Appendix 9-10 for review characteristics.

Risk of bias within studies

For peer review only

Most reviews were evaluated as having low (n = 14) or critically low (n = 5) confidence with their results. The remainder had moderate (n = 8) or high (n = 5) confidence (see Appendix 11).

Synthesis of results.

Determining the barriers and facilitators to women help-seeking and accessing PMH care.

A total of six overarching themes, mapped onto a multi-level framework (43), made up of 62 subthemes were identified (see Appendix 12). The multi-level framework is an extension of Ferlie and Shortell's Levels of Change framework (43) with six levels, instead of four. The first level is the individual level, which reflects factors related to the person themselves. The second level is HP, which reflects factors related to the HP. Interpersonal refers to the relationship between women and HPs, this is an extension of Ferlie and Shortell's work and was included because this theme was represented in the literature (37). The next theme is organisational, which relates to how the organisation is run, and the type of care the organisation delivers. The literature provided multiple examples of how women wanted their care designed. As the organisation is in charge of designing and providing care, ideal care was mapped as a sub-theme under this theme. The political level relates to the policies and governing that may impact on women, and healthcare. The societal level relates to larger societal factors, such as stigma. It is important to note that these levels do not exist in isolation but often impact one another, for example a lack of political funding and policy will have a negative impact on how an organisation is run, staff burnout and thus the care delivered to women.

Each level of the multi-level framework (Figure 2) maps on to at least one part of the care pathway (Figure 3). Each level of the multi-level framework will be outlined below, and within each level, the most cited barriers and facilitators will be presented following the chronology of

For peer review only

the care pathway outlined in Figure 3. Recommendations for practice and policy can be found in Table 1. It should be noted that the review draws on international evidence, and not all the factors identified will exist to the same extent in all places.

Individual level factors.

Individual level factors were identified by 25 reviews.

Deciding to consult

Barriers that prevented women from deciding to consult included, not understanding the role of HPs (n = 6), and not knowing what perinatal mental illness is (n = 14):

> *'I don't know what postnatal depression is — how you're supposed to feel, look, or whatever. I don't know. I have no idea … what exactly is postnatal depression? What are you supposed to be doing, saying, or whatever? I don't know.'* (44, p.e694)

Not knowing what perinatal mental illness is led to some women believing their symptoms were a normal part of motherhood (n = 8), or led to women attributing their symptoms to external causes (e.g. job loss; n = 8), or physical causes such as hormones (n = 9):

> *'I thought it was just lack of sleep and this heavy cold. I thought that after a good night's sleep it would get better, and I would be able to manage'* (44, p.e696)

Other barriers at this stage of the care pathway included dealing with symptoms by ignoring them (n = 6), or minimising them (n = 12); not knowing where to go in order to seek help (n = 7); and the fear of being seen as a bad mum or fear of social services involvement (n = 7).

Facilitators to deciding to consult was recognising that something was wrong (n = 9) and having supportive family and friends (n = 5):

For peer review only

'That's when I thought, you know: "Something is really wrong here, I need to go to the doctors if I'm thinking about killing myself."' (44, p.e694) [Recognising something is wrong]

'It was sort of my partner saying to me: "Right, if you don't go, I'm basically making you an appointment … You can't just keep feeling like this."' (44, p.e694) [Supportive family and friends]

Deciding to disclose

One barrier at this stage of the care pathway was not understanding the HP's role, perceiving them as agents of social control (n = 4):

'I don't really know what their job is. Nobody gave me, like, the parameters of this role of the health visitor [maternal and child health nurse]….'(44, p.e695)

Linked to this was the fear of social services involvement and the removal of their child (n = 7), as well as fears of being judged to be a bad mum (n = 8):

'I even went in at 3 months and I talked to a health nurse, and I just lied through my teeth because I thought, what are they going to do if they find out I can't be a good mom?' (45, p.732-733)

Access to care

The most cited barrier at this stage of the care pathway was logistical reasons (n = 13) such as travel costs, lack of childcare and timing of services.

HP related factors.

HP level factors were reported by 18 reviews.

First contact with HPs

HPs not recognising women's help-seeking or symptoms (n = 4), and being dismissive or

For peer review only

normalising women's symptoms (n = 8) were barriers at this early stage in the care pathway:

'I did ask for support, but I didn't really get any. And the health visitor's response — "Well you seem like you're doing all right" – which kind of closes it off, doesn't it' (44, p.e696)

Linked to this, HPs appearing to not have enough time to address women's concerns was also a barrier

'The health visitor said something like: "You know, in this community we have to look after a thousand and something babies." And that instilled in me the feeling, like: "Oh, they are very busy these people, and I don't have to be bothering them all the time' (44, p.e696)

Assessment/Screening

Assessment being carried out in a formulaic tick-box way, or not being carried out at all (n = 3) was the most cited barrier.

Deciding to disclose

The most reported barrier at this stage of the care pathway was HPs appearing to not have enough time (n = 4) or HPs being dismissive or normalising women's symptoms (n = 4).

Referral

Women's perception of HPs knowledge of referral pathways/other services (n = 3) and HPs not recognising women's help-seeking or symptoms (n = 2) were barriers to referral:

'I purposely circled the things 'cos I'm struggling … the health visitor didn't get back to me, which I'm really disappointed about.' (44, p.e696)

Access to care, provision of optimal care, and women's experiences of care

These stages of the care pathway were mainly influenced by the characteristics of HPs.

For peer review only

For example, HPs who were trustworthy, responsive, non-judgemental, understanding, caring, interested, warm, empathetic, and positive (n = 12) were facilitators. On the other hand, unhelpful or uninterested staff were barriers (n = 2).

Interpersonal factors.

Interpersonal level factors were identified by 14 reviews.

Deciding to consult, deciding to disclose, and women's experience of care.

The development of a strong and trusting relationship with a HP (n = 10) was a facilitator to women at each of these stages of the care pathway:

'She's a supplement to my own mother. She's easy to talk to. I depend on her. She's not just there to take care of the baby but for the mothers too. She started a group for us new mothers.' (46, p.79)

First contact with HPs, assessment, and provision of optimal care

Language difficulties (n = 6), and a lack of shared decision making (n = 6) were barriers at these stages of the care pathway:

'When the midwife visits, I can only speak the sentences about requesting a translator … They said that this kind of service is limited … that is what is difficult being Chinese—language barrier.' (47, p.6) [Language difficulties]

'… it would have been good I think to have been listened to about the side effects. I was on a very high dose of Olanzapine [sic] and it just knocks you out …' (48, p.754) [Shared decision making]

Organisational factors.

Organisational level factors were identified by 21 reviews.

For peer review only

Screening/Assessment

The most cited barriers to screening/assessment was the wording or contents of the tool (n = 2), or if the tool was delivered in a tick-box way (n = 6).

'There's so much more that you want to say, rather than just answering quite closed questions.' (44, p.e695)

Some women found screening tools particularly problematic if the tool was not in her first language, indicating that cultural factors can overlap with organisational factors. For example, one review reported that certain questions may not elicit true feelings from Vietnamese women living in the UK because of the shame of admitting to these (49). Further, question Q10 on the EPDS (50) ('the thought of harming myself has occurred to me') was seen as problematic to Arabic, Vietnamese, and Black Caribbean mothers (49) living in the UK or USA, highlighting the need for culturally sensitive and relevant assessment tools.

Access to care

Practical characteristics (n = 5) of the organisation and services offered, such as a lack of childcare facilities, hard to reach locations, and timing of appointments were a barrier to access:

'You have to have someone to look after your baby so who am I going to get to look after [my baby]' (44, p.e695)

Other barriers at this stage of the care pathway included, a lack of services or overstretched services (n = 7), a lack of collaboration across services (n = 3), and lack of continuity of care (n = 2):

'You shouldn't have to press that danger button of 'I'm gonna self-harm' or 'I'm gonna hurt my children' for someone to help you.' (48, p.756) [Lack of services]

'My GP [general practitioner/family doctor] says go the HV [health visitor] and HV says

For peer review only

go to GP. I don't know what to do, I need help, don't know where to go, or who to turn to' (47, p.5) [Lack of collaboration across services]

'Every time I went to see the midwife, or…, I always had somebody different, and I don't want to tell 10 people my story.' (48, p.752) [Lack of continuity of care]

Women reported wanting care that gave them an opportunity to talk to someone and discuss their emotional difficulties (n = 8); some women wanted this opportunity within a peer support or group setting (n = 12) and reported that an appropriate peer group could provide them with validation for their feelings (n = 3). Care also needed to be individualised (n = 10), and be culturally sensitive (n = 8):

'In Pakistan we only saw lady professionals, but here you don't have a choice, you have to see the men as well otherwise you don't get to see a doctor...' (51, p.10)

Women also appreciated care that provided them with information about PMH problems (n = 5). Further, the location of the care needs to be easy to reach or carried out in women's homes (n = 7), and women should not be discharged too early from these services (n = 4).

Political factors.

Political factors were defined as factors that governmental agencies have influence over (e.g. poverty, immigration, housing). Eight women identified these factors.

Deciding to consult and access to care.

Immigration status (n = 4) and economic status (n = 8) influenced women's decision to consult and access to care:

'Because when you're legal you can take the child to the day-care and look for a job. . . if you don't work, it's like you're dead, being alive.. . .' (52, p.13) [Immigration status]

'…if she has no money, how is she going to find help [with PPD]?' (53, p.12) [Economic

For peer review only

status]

This is due to the costs of healthcare and women's fear of being deported if they access help. Economic status was often exacerbated by immigration status with women reporting not being able to get health insurance due to their immigration status (n = 4).

Women's experience of care

Economic status also impacted women's experience of care in terms of women not being able to feel any sense of wellbeing when they were unable to fulfil basic needs such as '…*Not having enough money to make ends meet…*' (54, p.12) (n = 4).

Societal factors.

Societal factors were identified in 24 reviews. The main societal factors that influenced a woman's journey along the care pathway were culture, societies' norms of what a "good mum" should look like (maternal norms), and stigma. All these factors intertwine and influence one another. There was only one review that only included studies from LMICs(55), therefore these results mainly refer to western cultures.

Deciding to consult and deciding to disclose.

Stigma (n = 21) and the maternal norm for women to show they are strong, that they can cope and be a good mother (n = 18), prevented women from deciding to consult, and deciding to disclose:

> '*Mothers tend to think they should always be there. And mothers are supposed to be always rock solid, aren't they? Everyone assumes that.*' (56, p.568)

Adherence to cultural traditions (n = 16) in women who had moved to Western countries, impacted their decision to consult and disclose. Two reviews reported that Hispanic women living in the USA felt they needed to remain strong (n = 2), feeling they needed to show that they

For peer review only

could cope, and that stigma prevented them from seeking help; they did not want to be seen as "*crazy*" or "*loco*" (57, p.97).

Four reviews found that South Asian women living in the UK did not consult or disclose for similar cultural reasons e.g. "*for fear of an inability to perform their role as a woman and a mother*" (58, p. 325), perceiving symptoms in religious terms "*All illness is coming from God*" (44, p.e649) , and stigma:

> *There is a huge stigma of being mentally ill in the public, but for us Asians there is a double disadvantage. I really fear that work will find out.'* Pakistani woman living in the UK (47, p.5)

Black African and Caribbean women living in the UK or USA were also deterred from deciding to consult and disclose because of the expectation of women to be strong and be able to cope (n = 4).

Access to care, provision of optimal care and women's experience of care

Women's cultural backgrounds highlighted the need for culturally sensitive care. The lack of this care was as a barrier to access, provision of optimal care and women's experiences of care (n = 8). Two reviews explained how Hispanic women living in the USA felt that language barriers, cultural insensitivity, and financial difficulties were a barrier to them accessing care (52,53). Further, Jordanian women (living in Australia) spoke of being torn between their own cultural practices and Western health advice, having HPs putting pressure demands upon them to change their beliefs and behaviours (52). For women living in sub-Saharan Africa, the cultural tradition of confinement after birth meant women felt unable to leave their house for fear of being shamed. This was further exacerbated by the attribution of postnatal ill health to inadequate adherence to tradition (55).

For peer review only

Table 1. Recommendations for improving PMH care for women.

System level factor	Theme	Recommendation
Individual	Beliefs about health services<br>Beliefs about HPs<br>Beliefs about mental illness<br>Fear of judgement<br>Logistics	Improvement of mental health literacy for, women, family, friends, and all who meet perinatal women[1,2]<br>Free access to healthcare[3]<br>Woman-centred care[4]
Healthcare professional	Characteristics<br>Time<br>Training and knowledge	Attend training in communication skills[5]<br>Attend training in perinatal mental health to reduce stigma[5]<br>Attend training in cross-cultural presentations of mental health difficulties[5]
Interpersonal	Relationship and rapport<br>Language barriers<br>Shared decision making<br>Communication<br>Information provision	Healthcare professional to attend training in communication skills[5]<br>Healthcare professional to attend training in PMH to reduce stigma[5]<br>Healthcare professional to attend training in cross-cultural presentations of mental health difficulties[5]<br>Provision of continuity of carer[4]
Organisational (including ideal care)	Lack of services/overstretched services<br>Characteristics of the service including continuity of carer<br>Collaboration across services	Individualised and culturally appropriate care co-designed with women.[4]<br>Improved funding for PMH services.[3]<br>Improved guidance for implementing PMH care*.[1,6]
Political	Immigration and economic status<br>Healthcare costs	Equal rights to healthcare[3]<br>Free healthcare[3]<br>Laws to protect those with immigration status[3]<br>International policy that supports the funding and implementation of personalised culturally appropriate care[1]

For peer review only

Societal	Stigma<br>Culture<br>Maternal norms	International, culturally sensitive public mental health campaigns to increase knowledge about mental illness and improve attitudes about people with mental illness(59-64)[1]<br><br>The continuation of international policies to promote gender equality, higher paid parental leave (65), increased opportunity for women in the labour force (66–68), the right to access contraception and abortion (69).[3]

*Recommendations for implementing PMH assessment, care and treatment can be found in (37)

1. Recommendations for public health services (e.g., the NHS, the European Public Health Association, Public Health Association of Australia etc.)
2. Recommendations for third sector organisations (e.g., the National Childbirth Trust, UK; The Babes Project, Australia etc.)
3. Recommendation for the government
4. Recommendation for organisation
5. Recommendations for healthcare professionals
6. Recommendations for academics/researchers

For peer review only

Discussion

This meta-review identified a wide range of barriers and facilitators to women accessing PMH care, that were influential at different levels (Figure 2) and across different stages of the care pathway (Figure 3).

Previous research has identified multiple factors that act as barriers to women seeking and accessing help for PMH problems. The factors include women not recognising the need to seek help (44,56,70–72), the need for HPs to receive training on perinatal mental illness and cultural sensitivity (44–46,49, 51,73–75), continuity of care (44–46,48,51, 58,70,75), and stigma (44,45,48, 52-54, 56,57,74,76). Our findings are in line with these previous studies and adds to the body of evidence by identifying barriers and facilitators to PMH care, across the globe, and presenting them on a multi-level model, and at different stages of the care pathway. This provides opportunities for HPs, service managers and policy makers to identify barriers and facilitators that are most relevant to their context. The mapping of barriers and facilitators in this way, has also led to the development of evidence-based recommendations for design and delivery of PMH care.

Recommendations for PMH care

The results from this meta-review can be used to inform healthcare providers and policy makers on the optimal characteristics of PMH care and are summarised in Table 1. This meta-review showed a complex interplay of multi-level factors that influence women's help-seeking and access to PMH care. Thus, recommendations for policy and practice also relate to both international level guidelines, and guidelines for national and individual level care. International level guidelines should facilitate more personalised care and should feed into national guidelines and be adopted where appropriate.

For peer review only

Societal factors such as stigma, maternal norms, and culture play a large role in women accessing care. Research suggests that public mental health campaigns can increase knowledge about mental illness and improve attitudes about people with mental illness (64). Therefore, increasing women's, families', those who have regular contact with women in the perinatal period, and the public's mental health literacy, could be carried out on an international level. This could be done through public health campaigns, and education within the community, such as antenatal education, and at healthcare appointments.

Maternal norms identified in this meta-review related to women believing that they needed to be strong and show they could cope. There may be some potential to change societal beliefs around maternal norms through increasing societal expectations about fathers' role in the family through more equal parental leave. For example, in countries where parental leave is more equal (e.g. Finland), the uptake of paid paternity leave is higher (65). Changing society's maternal norms could also be done by increasing women's equality. Research suggests that stereotypes of what a mother or a woman should look like are beginning to change in countries where women have gained more participation in the labour force (68) and have the right to access contraception and abortion (69). However, research is needed to corroborate these findings.

At the political level, immigration and economic status, and healthcare costs were barriers to women accessing healthcare. The results from this meta-review show how race and gender interact to influence women's experiences of the healthcare system (intersectionality) (77). White women living within their country of birth who try to access PMH care are faced with barriers (e.g. no childcare support), but women of colour, migrant women, or migrant women of colour are faced with additional barriers (e.g. language barriers, structural/systematic

For peer review only - http://bmjopen.bmj.com/site/about/guidelines.xhtml

discrimination). This finding is supported by research in general healthcare that has found ethnic minority and migrant women are disproportionately affected by existing barriers to accessing healthcare (78). As found in this meta-review, these barriers include language and communication barriers, stigma, the cost of healthcare (79), and the inability to access culturally appropriate services (80). This shows the need for equal rights to healthcare, regardless of immigration or economic status. Further, changes at the legislative level are needed to protect those who have migrated to a different country from being penalised for accessing healthcare (79).

At the organisational level this meta-review identified a range of factors that women viewed as ideal care. Women appreciated the opportunity to discuss screening results with HPs and for it not to be filled out as a "tick box" exercise (47). In terms of treatment, women wanted the opportunity to talk to someone (a HP or a peer) about their difficulties (45,46,56,58,81,82). They found peer support offered them a sense of validation which they appreciated (83). To overcome logistical barriers, the location of services should be easily accessible, or in women's homes (45,48,49, 53). Further, the length of treatment should be flexible and based on women's needs. Women did not want a "one size fits all" approach but wanted individualised treatment that was culturally appropriate (44–46,48,52,57,72,75,81).

At the interpersonal and HP level, the characteristics of the HPs were important, as was their communication with women. Women reported that many HPs normalised their symptoms or were dismissive of their attempts to seek help. This may reflect the heavy workload experienced by many HP (84–86). For example, research suggests that consultations where mental health problems are discussed take longer, and HPs often feel there is not enough time to address concerns fully (86,87). This finding could also reflect inadequate training (88). Within

For peer review only

the UK, guidance states that all midwives and health visitors should receive training in order for them to identify, care for and refer women with PMH problems (89). However, a synthesis of 30 studies found that midwives lack the confidence, knowledge, and training to do this (90), therefore training around mental health is important. Another key training need is cultural sensitivity and cross-cultural understanding of PMH. Some systematic reviews in this meta-review identified that women were treated in a culturally insensitive way by HPs and that women of colour were less likely to be offered treatment or be asked about their mental health. It has been suggested that training given at medical and nursing school does not do enough to reduce unconscious biases against marginalised groups, which in turn influences treatment provided by healthcare providers (91).

Improved interpretation services within PMH care may aid culturally sensitive care. Another potential way to improve culturally sensitive care is through the recruitment and retention of healthcare providers from diverse backgrounds (92). This strategy has the potential to improve interpersonal relationships between HPs and patients (93,94), which may therefore increase disclosure of PMH problems to HPs. In addition, research suggests increased representation of diverse populations in health care is associated with improved communication between health providers (95,96), which therefore may reduce the risk of women falling through gaps in the care pathway.

Further, it has been argued that the way the western world views mental illness is very ethnocentric (97) and that culture and society influences what is viewed as a mental illness (98). This may mean that some women's attempts to seek help are missed by HPs. It is therefore crucial that cultural sensitivity and cross-cultural mental health training is provided to HPs.

For peer review only

In terms of individual level factors, many of these barriers can be improved through the recommendations suggested above. For example, improvement of knowledge around mental health is likely to reduce women's fear of judgement and self-stigma and increase her awareness of the symptoms she is experiencing, which may encourage help-seeking (99). Re-design of care, such as providing easily accessible healthcare may reduce the logistical barriers women experience.

Strengths and limitations

The strength of this meta-review is the synthesis of a large amount of information from 32 systematic reviews from many different countries in order to identify barriers and facilitators to women deciding to seek help, access help, and engage in PMH care. This information was then used to provide recommendations for the design and delivery of care. A limitation of the methodology is that only reviews published in academic journals and written in English language were included. Relevant reviews from health services, charities, third sector organisations, and other grey literature may have been missed. Another limitation is that only 10% of studies had duplicate data extraction. However, concordance was high, and it is therefore unlikely that any key themes were missed.

A limitation about the papers included in the meta-review was that most of them were rated as having low or critically low quality, meaning less confidence can be placed on their results. However, the qualitative sensitivity analysis found that most themes were supported in both the higher quality and lower quality reviews and including all reviews meant there was more focus on marginalised women, such as refugees, migrants and women living in sub-Saharan Africa. This shows that the results from this meta-review can be interpreted with reasonable confidence.

For peer review only

Implications for future research

This review has revealed several limitations with the current evidence base on this topic. Very few systematic reviews (n = 2) addressed the severity of illness, only one review looked at severe PMH problems (73) and most reviews (n = 24) focussed only on depression. There may be different barriers for other PMH problems therefore future research should focus on researching the barriers and facilitators to women with disorders other than depression. Another limitation with the identified reviews is that no reviews specified whether women had given birth to singletons only, or twins/higher-order multiples. This is important, as parents of twins or multiples report unique experiences in accessing PMH care (100).

Furthermore, reviews only covered the inclusion of studies carried out in 25 countries, and only one review included studies that were only carried out in LMICs (55). More research is needed in other countries to further aid our understanding of help-seeking and accessing care in women with PMH problems. In addition, none of the identified reviews included studies from diverse families, including same-sex couples, and the transgender community. It is important that future research recruits more diverse populations to ensure all voices are heard.

Most reviews were rated as having low or critically low quality meaning less confidence can be placed on their results. However, the qualitative sensitivity analysis found that most themes were supported in both the higher quality and lower quality reviews and including all reviews meant there was more focus on marginalised women, such as refugees, migrants, and women living in sub-Saharan Africa. This shows that the results from this meta-review can be interpreted with reasonable confidence.

Conclusion

For peer review only

The findings from this review point to a complex interplay of individual and system level factors across different stages of the care pathway that can influence whether women seek help and access care for PMH problems. These factors should all be considered by policy makers to improve the identification and treatment of PMH problems. Recommendations for the design and delivery of PMH care have been produced, building on the barriers and facilitators identified in this review. The identified barriers and facilitators point to the need for an international effort to reduce mental health stigma, and increase woman-centred, flexible care, delivered by well-trained and culturally competent HPs.

For peer review only

Figure 1. PRISMA Flow Diagram

Figure 2. The MATRIx multi-level model of barriers and facilitators to women accessing PMH care.

Figure 3. Barriers and facilitators mapped onto the MATRIx care pathway.

a. Contributorship statement: Rebecca Webb was involved in the design of the research and carried out screening, quality appraisal, analysis, write up of manuscript and editing of manuscript. Nazihah Uddin contributed to screening and quality appraisal of papers and provided detailed feedback on the manuscript. Georgina Constantinou contributed to screening and quality appraisal of papers provided detailed feedback on the manuscript. Elizabeth Ford was involved in the conceptualisation of the project, the design of the research, analysis, and provided detailed feedback on the manuscript. Abigail Easter was involved in the conceptualisation of the project, the design of the research, analysis, and provided detailed feedback on the manuscript. Judy Shakespeare was involved in the conceptualisation of the project, the design of the research, analysis, and provided detailed feedback on the manuscript. Agnes Hann provided PPI input and detailed feedback on the manuscript. Nia Roberts completed the literature searches. Fiona Alderdice contributed to the design of the research and provided detailed feedback on the manuscript. Andrea Sinesi contributed to the design of the research and provided detailed feedback on the manuscript. Rose Coates contributed to the design of the research and provided detailed feedback on the manuscript. Sally Hogg contributed to the design of the research and provided detailed feedback on the manuscript. Susan Ayers was the project manager, was involved in the conceptualisation of the project, the design of the research, analysis, and

For peer review only

provided detailed feedback on the manuscript. The MATRIx study team provided oversight to the running of the project, and feedback on the work produced.

b. Competing interests: None.

c. Funding: National Institute for Health Research. Health Services and Delivery Research Programme. NIHR128068

d. Data sharing statement: Data is available on request to the corresponding author: Rebecca.Webb.2@city.ac.uk

e. Ethics statement: This study does not involve human participants. This is an evidence synthesis, therefore approval by an ethics committee is not necessary.

f. Collaborators: The MATRIx Study Team: Elaine Clark, Helen Cheyne, Evelyn Frame, Simon Gilbody, Agnes Hann, Sarah McMullen Camilla Rosan, Debra Salmon, Andrea Sinesi, Clare Thompson, and Louise Williams.

For peer review only

References

1. Falah-Hassani K, Shiri R, Dennis CL. The prevalence of antenatal and postnatal co-morbid anxiety and depression: A meta-analysis. Psychological Medicine. 2017.

2. Knight M, Bunch K, Tuffnell D, Shakespeare J, Kotnis R, Kenyon S, et al. Saving Lives, Improving Mothers' Care: Lessons learned to inform maternity care from the UK and Ireland Confidential Enquiries into Maternal Deaths and Morbidity 2016-18. MBRRACE-UK - Mothers and Babies: Reducing Risk throught Audits and Confidential Enquiries across the UK. Oxford; 2020.

3. Vigod SN, Kurdyak PA, Dennis CL, Gruneir A, Newman A, Seeman M V, et al. Maternal and newborn outcomes among women with schizophrenia: a retrospective population-based cohort study. BJOG Int J Obstet Gynaecol. 2014;121(5):566–74.

4. McAllister-Williams RH, Baldwin DS, Cantwell R, Easter A, Gilvarry E, Glover V, et al. British Association for Psychopharmacology consensus guidance on the use of psychotropic medication preconception, in pregnancy and postpartum 2017. J Psychopharmacol (Oxf). 2017;31(5):519–52.

5. Rusner M, Berg M, Begley C. Bipolar disorder in pregnancy and childbirth: a systematic review of outcomes. BMC Pregnancy Childbirth. 2016;16(1):1–18.

6. Glover V. Maternal stress during pregnancy and infant and child outcome. In: The Oxford handbook of perinatal psychology. 2016.

7. Ding XX, Wu Y Le, Xu SJ, Zhu RP, Jia XM, Zhang SF, et al. Maternal anxiety during pregnancy and adverse birth outcomes: A systematic review and meta-analysis of prospective cohort studies. Journal of Affective Disorders. 2014.

8. Grigoriadis S, Wilton AS, Kurdyak PA, Rhodes AE, VonderPorten EH, Levitt A, et al. Perinatal suicide in Ontario, Canada: a 15-year population-based study. Cmaj. 2017;189(34):E1085–92.

9. Palladino CL, Singh V, Campbell J, Flynn H, Gold K. Homicide and suicide during the perinatal period: findings from the National Violent Death Reporting System. Obstet Gynecol. 2011;118(5):1056.

10. Fuhr DC, Calvert C, Ronsmans C, Chandra PS, Sikander S, De Silva MJ, et al. Contribution of suicide and injuries to pregnancy-related mortality in low-income and middle-income countries: a systematic review and meta-analysis. Lancet Psychiatry. 2014;1(3):213–25.

11. Murray L, Fiori-Cowley A, Hooper R, Cooper P. The Impact of Postnatal Depression and Associated Adversity on Early Mother-Infant Interactions and Later Infant Outcome. Child Dev. 1996.

For peer review only

12. Liu Y, Kaaya S, Chai J, McCoy DC, Surkan PJ, Black MM, et al. Maternal depressive symptoms, and early childhood cognitive development: A meta-analysis. Psychol Med. 2017

13. Netsi E, Pearson RM, Murray L, Cooper P, Craske MG, Stein A. Association of persistent and severe postnatal depression with child outcomes. JAMA Psychiatry. 2018.

14. Oyetunji A, Chandra P. Postpartum stress, and infant outcome: A review of current literature. Psychiatry Research. 2020.

15. Prenoveau JM, Craske MG, West V, Giannakakis A, Zioga M, Lehtonen A, et al. Maternal postnatal depression and anxiety and their association with child emotional negativity and behavior problems at two years. Dev Psychol. 2017.

16. Letourneau N, Leung B, Ntanda H, Dewey D, Deane AJ, Giesbrecht GF. Maternal and paternal perinatal depressive symptoms associate with 2-and 3-year-old children's behaviour: Findings from the APrON longitudinal study. BMC Pediatr. 2019.

17. O'Donnell M, Maclean MJ, Sims S, Morgan VA, Leonard H, Stanley FJ. Maternal mental health and risk of child protection involvement: Mental health diagnoses associated with increased risk. J Epidemiol Community Health. 2015.

18. Rees S, Channon S, Waters CS. The impact of maternal prenatal and postnatal anxiety on children's emotional problems: a systematic review. Eur Child Adolesc Psychiatry. 2019.

19. Glasheen C, Richardson GA, Fabio A. A systematic review of the effects of postnatal maternal anxiety on children. Archives of Women's Mental Health. 2010.

20. Garthus-Niegel S, Horsch A, Handtke E, von Soest T, Ayers S, Weidner K, et al. The impact of postpartum posttraumatic stress and depression symptoms on couples' relationship satisfaction: A population-based prospective study. Front Psychol. 2018.

21. Nicholls K, Ayers S. Childbirth-related post-traumatic stress disorder in couples: A qualitative study. Br J Health Psychol. 2007.

22. Delicate A, Ayers S, Easter A, McMullen S. The impact of childbirth-related post-traumatic stress on a couple's relationship: a systematic review and meta-synthesis. Journal of Reproductive and Infant Psychology. 2018.

23. Hinton L, Locock L, Knight M. Support for mothers and their families after life-threatening illness in pregnancy and childbirth: a qualitative study in primary care. Br J Gen Pract. 2015.

24. Bauer A, Parsonage M, Knapp M, Iemmi V, Adelaja B. The costs of perinatal mental health problems [Internet]. Centre for Mental Health and London School of Economics; 2014. Available from: https://www.centreformentalhealth.org.uk/sites/default/files/2018-09/costsofperinatal.pdf

For peer review only

25. Huang L, Zhao Y, Qiang C, Fan B. Is cognitive behavioral therapy a better choice for women with postnatal depression? A systematic review and meta-analysis. PLoS ONE. 2018.

26. Loughnan SA, Wallace M, Joubert AE, Haskelberg H, Andrews G, Newby JM. A systematic review of psychological treatments for clinical anxiety during the perinatal period. Archives of Women's Mental Health. 2018.

27. Molyneaux E, Telesia LA, Henshaw C, Boath E, Bradley E, Howard LM. Antidepressants for preventing postnatal depression. Cochrane Database of Systematic Reviews. 2018.

28. World Health Organization. Millennium Development Goals (MDGs). 2000.

29. World Health Organization. Improving Maternal Mental Health. Geneva; 2008.

30. Baron EC, Hanlon C, Mall S, Honikman S, Breuer E, Kathree T, et al. Maternal mental health in primary care in five low- and middle-income countries: a situational analysis. BMC Health Serv Res. 2016 Feb 16;16(1):53.

31. Moore D, Drey N, Ayers S. Use of Online Forums for Perinatal Mental Illness, Stigma, and Disclosure: An Exploratory Model. JMIR Ment Health. 2017;4(1):e6.

32. Kozhimannil KB, Adams AS, Soumerai SB, Busch AB, Huskamp HA. New Jersey's Efforts to Improve Postpartum Depression Care Did Not Change Treatment Patterns for Women on Medicaid. Health Aff (Millwood). 2011;30(2):293–301.

33. Perinatal Anxiety & Depression (PANDA). Submission to the Productivity Commission Mental Health Inquiry: The Social and Economic Benefits of Improving Perinatal Mental Health [Internet]. 2019. Available from: https://www.pc.gov.au/__data/assets/pdf_file/0012/250203/sub674-mental-health.pdf

34. Gavin NI, Meltzer-Brody S, Glover V, Gaynes BN. Is population-based identification of perinatal depression and anxiety desirable? A public health perspective on the perinatal depression care continuum. In: Milgrom J, Gemmill AW, editors. Identifying perinatal depression and anxiety: Evidence-based practice in screening, psychosocial assessment, and management. London: Wiley Blackwell; 2015. p. 11–31.

35. Royal College of Obstetricians & Gynaecologists. Maternal mental health - women's voices [Internet]. RCOG. [cited 2023 Mar 15]. Available from: https://www.rcog.org.uk/for-the-public/rcog-engagement-listening-to-patients/maternal-mental-health-womens-voices/

36. Milgrom J, Gemmill AW. Identifying perinatal depression and anxiety: evidence-based practice in screening, psychosocial assessment and management. John Wiley & Sons; 2015.

37. Webb R, Uddin N, Ford E, Easter A, Shakespeare J, Roberts N, et al. Barriers and facilitators to implementing perinatal mental health care in health and social care settings: a systematic review. Lancet Psychiatry. 2021;8(6):521–34.

For peer review only

38. Grant MJ, Booth A. A typology of reviews: An analysis of 14 review types and associated methodologies. Health Information and Libraries Journal. 2009.

39. Samnani SS, Vaska M, Ahmed S, Turin TC. Review typology: The basic types of reviews for synthesizing evidence for the purpose of knowledge translation. Journal of the College of Physicians and Surgeons Pakistan. 2017.

40. Moher D, Liberati A, Tetzlaff JM, Altman DG. Preferred reporting items for systematic reviews and meta-analyses: the PRISMA statement. PLoS Med. 2009;6(7):e1000097.

41. Shea BJ, Reeves BC, Wells GA, Thuku M, Hamel C, Moran J, et al. AMSTAR 2: A critical appraisal tool for systematic reviews that include randomised or non-randomised studies of healthcare interventions, or both. BMJ. 2017;21:358.

42. Thomas J, Harden A. Methods for the thematic synthesis of qualitative research in systematic reviews. BMC Med Res Methodol. 2008;8(1):1–10.

43. Ferlie EB, Shortell SM. Improving the quality of health care in the United Kingdom and the United States: a framework for change. Milbank Q. 2001;79(2):281–315.

44. Button S, Thornton A, Lee S, Shakespeare J, Ayers S. Seeking help for perinatal psychological distress: a meta-synthesis of women's experiences. Br J Gen Pract. 2017;67(663):e692–9.

45. Hadfield H, Wittkowski A. Women's experiences of seeking and receiving psychological and psychosocial interventions for postpartum depression: a systematic review and thematic synthesis of the qualitative literature. J Midwifery Womens Health. 2017;62(6):723–36.

46. Morrell CJ, Sutcliffe P, Booth A, Stevens J, Scope A, Stevenson M, et al. A systematic review, evidence synthesis and meta-analysis of quantitative and qualitative studies evaluating the clinical effectiveness, the cost-effectiveness, safety and acceptability of interventions to prevent postnatal depression. Health Technol Assess. 2016;20(37):1–414.

47. Sambrook Smith M, Lawrence V, Sadler E, Easter A. Barriers to accessing mental health services for women with perinatal mental illness: Systematic review and meta-synthesis of qualitative studies in the UK. BMJ Open. 2019;9(1):e024803.

48. Megnin-Viggars O, Symington I, Howard LM, Pilling S. Experience of care for mental health problems in the antenatal or postnatal period for women in the UK: a systematic review and meta-synthesis of qualitative research. Arch Womens Ment Health. 2015;18:745–59.

49. Brealey SD, Hewitt C, Green JM, Morrell J, Gilbody S. Screening for postnatal depression– is it acceptable to women and healthcare professionals? A systematic review and meta-synthesis. J Reprod Infant Psychol. 2010;28(4):328–44.

50. Cox JL, Holden JM, Sagovsky R. Detection of Postnatal Depression: Development of the 10-item Edinburgh Postnatal Depression Scale. Br J Psychiatry. 1987 Jun;150(6):782–6.

For peer review only

51. Watson H, Harrop D, Walton E, Young A, Soltani H. A systematic review of ethnic minority women's experiences of perinatal mental health conditions and services in Europe. PloS One. 2019;14(1):e0210587.

52. Schmied V, Black E, Naidoo N, Dahlen HG, Liamputtong P. Migrant women's experiences, meanings and ways of dealing with postnatal depression: A meta-ethnographic study. PloS One. 2017;12(3):e0172385.

53. Hansotte E, Payne SI, Babich SM. Positive postpartum depression screening practices and subsequent mental health treatment for low-income women in Western countries: a systematic literature review. Public Health Rev. 2017;38:1–17.

54. Lucas G, Olander EK, Ayers S, Salmon D. No straight lines – young women's perceptions of their mental health and wellbeing during and after pregnancy: a systematic review and meta-ethnography. BMC Womens Health. 2019;19(1):152.

55. Wittkowski A, Gardner PL, Bunton P, Edge D. Culturally determined risk factors for postnatal depression in Sub-Saharan Africa: A mixed method systematic review. J Affect Disord. 2014 Jul 1;163:115–24. 56. Staneva AA, Bogossian F, Wittkowski A. The experience of psychological distress, depression, and anxiety during pregnancy: A meta-synthesis of qualitative research. Midwifery. 2015;31(6):563–73.

57. Tobin C, Napoli D, Beck C. Refugee and Immigrant Women's Experience of Postpartum Depression: A Meta-Synthesis. J Transcult Nurs Off J Transcult Nurs Soc [Internet]. 2018 [cited 2023 Mar 15];29(1). Available from: https://pubmed.ncbi.nlm.nih.gov/28826328/58. Dennis CL, Chung-Lee L. Postpartum Depression Help-Seeking Barriers and Maternal Treatment Preferences: A Qualitative Systematic Review. Birth. 2006;33(4):323–31.

59. Dietrich S, Mergl R, Freudenberg P, Althaus D, Hegerl U. Impact of a campaign on the public's attitudes towards depression. Health Educ Res. 2010;25(1):135–50.

60. Evans-Lacko S, Malcolm E, West K, Rose D, London J, Rüsch N, et al. Influence of Time to Change's social marketing interventions on stigma in England 2009-2011. Br J Psychiatry. 2013;202(s55):s77–88.

61. Jorm AF, Jorm AF, Christensen H, Griffiths KM, Jorm AF, Christensen H, et al. The impact of beyondblue: the national depression initiative on the Australian public's recognition of depression and beliefs about treatments. Aust N Z J Psychiatry. 2005;39(4):248–54.

62. Jorm AF, Christensen H, Griffiths KM. Changes in depression awareness and attitudes in Australia: the impact of beyondblue: the national depression initiative. Aust N Z J Psychiatry. 2006;40(1):42–6.

63. Makowski AC, Mnich EE, Ludwig J, Daubmann A, Bock T, Lambert M, et al. Changes in beliefs and attitudes toward people with depression and schizophrenia–results of a public campaign in Germany. Psychiatry Res. 2016;237:271–8.

For peer review only

64. Sampogna G, Bakolis I, Evans-Lacko S, Robinson E, Thornicroft G, Henderson C. The impact of social marketing campaigns on reducing mental health stigma: Results from the 2009–2014 Time to Change programme. Eur Psychiatry. 2017;40:116–22.

65. OECD - Social Policy Division. Parents' use of childbirth-related leave [Internet]. 2019. Available from: https://www.oecd.org/els/family/PF2-2-Use-childbirth-leave.pdf

66. Eagly AH, Nater C, Miller DI, Kaufmann M, Sczesny S. Gender stereotypes have changed: A cross-temporal meta-analysis of US public opinion polls from 1946 to 2018. Am Psychol. 2020;75(3):301.

67. Lopez-Zafra E, Garcia-Retamero R. Do gender stereotypes change? The dynamic of gender stereotypes in Spain. J Gend Stud. 2012;21(2):169–83.

68. Valiquette-Tessier SC, Gosselin J, Young M, Thomassin K. A literature review of cultural stereotypes associated with motherhood and fatherhood. Marriage Fam Rev. 2019;55(4):299–329.

69. Centre for Reproductive Rights. The World's Abortion Laws. Resources. [Internet]. 2021. Available from: https://reproductiverights.org/worldabortionlaws

70. Slade PP, Molyneux DR, Watt DA. A systematic review of clinical effectiveness of psychological interventions to reduce post traumatic stress symptoms following childbirth and a meta-synthesis of facilitators and barriers to uptake of psychological care. J Affect Disord. 2021 Feb 15;281:678–94.

71. Sorsa MA, Kylmä J, Bondas TE. Contemplating Help-Seeking in Perinatal Psychological Distress—A Meta-Ethnography. Int J Environ Res Public Health. 2021 Jan;18(10):5226.

72. Viveiros CJ, Darling EK. Perceptions of barriers to accessing perinatal mental health care in midwifery: A scoping review. Midwifery. 2019 Mar 1;70:106–18.

73. Forde R, Peters S, Wittkowski A. Recovery from postpartum psychosis: a systematic review and metasynthesis of women's and families' experiences. Arch Womens Ment Health. 2020 Oct 1;23(5):597–612.

74. Jones A. Help Seeking in the Perinatal Period: A Review of Barriers and Facilitators. Soc Work Public Health. 2019;34(7):596–605.

75. Scope A, Booth A, Morrell CJ, Sutcliffe P, Cantrell A. Perceptions and experiences of interventions to prevent postnatal depression. A systematic review and qualitative evidence synthesis. J Affect Disord. 2017 Mar 1;210:100–10.

76. Giscombe T, Hui A, Stickley T. Perinatal mental health amongst refugee and asylum-seeking women in the UK. Ment Health Rev J. 2020 Jan 1;25(3):241–53.

77. Crenshaw K. Mapping the Margins: Intersectionality, Identity Politics, and Violence against Women of Color. Stanford Law Rev. 1990 1991;43:1241.

For peer review only

78. Jayaweera H. Access to healthcare for vulnerable migrant women in England: A human security approach. Curr Sociol Monogr. 2018;66(2):273–85.

79. Germain S, Yong A. COVID-19 Highlighting Inequalities in Access to Healthcare in England: A Case Study of Ethnic Minority and Migrant Women. Fem Leg Stud. 2020 Nov 1;28(3):301–10.

80. Grey T, Sewell H, Shapiro G, Ashraf F. Mental Health Inequalities Facing U.K. Minority Ethnic Populations. J Psychol Issues Organ Cult. 3(S1):146–57.

81. Evans K, Spiby H, Morrell JC. Non-pharmacological interventions to reduce the symptoms of mild to moderate anxiety in pregnant women. A systematic review and narrative synthesis of women's views on the acceptability of and satisfaction with interventions. Arch Womens Ment Health. 2020 Feb 1;23(1):11–28.

82. Kassam S. Understanding Experiences of Social Support as Coping Resources among Immigrant and Refugee Women with Postpartum Depression: An Integrative Literature Review. Issues Ment Health Nurs. 2019 Dec 2;40(12):999–1011.

83. Jones CCG, Jomeen J, Hayter M. The impact of peer support in the context of perinatal mental illness: A meta-ethnography. Midwifery. 2014;30(5):491–8.

84. Bailey S, West M. Naming the issue: chronic excessive workload in the NHS [Internet]. The King's Fund. 2021 [cited 2023 Mar 15]. Available from: https://www.kingsfund.org.uk/blog/2021/06/naming-issue-chronic-excessive-workload-nhs

85. Trindade L de L, Pires DEP de. Implications of primary health care models in workloads of health professionals. Texto Contexto - Enferm. 2013 Mar;22:36–42.

86. Zantinge EM, Verhaak PF, Kerssens JJ, Bensing JM. The workload of GPs: consultations of patients with psychological and somatic problems compared. Br J Gen Pract. 2005 Aug 1;55(517):609–14.

87. Barriers and facilitators to GP–patient communication about emotional concerns in UK primary care: a systematic review | Family Practice | Oxford Academic [Internet]. [cited 2023 Mar 15]. Available from: https://academic.oup.com/fampra/article/37/4/434/5714005?login=false

88. Knaak S, Mantler E, Szeto A. Mental illness-related stigma in healthcare: Barriers to access and care and evidence-based solutions. Healthc Manage Forum. 2017;30(2):111–6.

89. Royal College of Midwives. Specialist Mental Health Midwives: What they do and why they matter [Internet]. 2018. Available from: https://www.rcm.org.uk/media/2370/specialist-mental-health-midwives-what-they-do-and-why-they-matter.pdf

For peer review only

90. Coates D, Foureur M. The role and competence of midwives in supporting women with mental health concerns during the perinatal period: A scoping review. Health Soc Care Th Community. 2019;27(4):e389–405.

91. Crear-Perry J, Maybank A, Keeys M, Mitchell N, Godbolt D. Moving towards anti-racist praxis in medicine. The Lancet. 2020 Aug 15;396(10249):451–3.

92. Davis CE, Lamson AL, Black KZ. MedFTs' Role in the Recruitment and Retention of a Diverse Physician Population: A Conceptual Model -. Contemp Fam Ther. 2022;44:88–100.

93. Kurek K, Teevan BE, Zlateva I, Anderson DR. Patient-Provider Social Concordance and Health Outcomes in Patients with Type 2 Diabetes: a Retrospective Study from a Large Federally Qualified Health Center in Connecticut. J Racial Ethn Health Disparities. 2016;3(2):217–24.

94. Thornton RLJ, Powe NR, Roter D, Cooper LA. Patient–physician social concordance, medical visit communication and patients' perceptions of health care quality. Patient Educ Couns. 2011 Dec 1;85(3):e201–8.

95. Hughes TM, Merath K, Chen Q, Sun S, Palmer E, Idrees JJ, et al. Association of shared decision-making on patient-reported health outcomes and healthcare utilization. Am J Surg. 2018 Jul 1;216(1):7–12.

96. Mozafaripour S. Diversity in Healthcare and the Importance of Representation [Internet]. University of St. Augustine for Health Sciences. 2021 [cited 2023 Mar 15]. Available from: https://www.usa.edu/blog/diversity-in-healthcare/

97. Watters E. Crazy Like Us: The Globalization of the American Psyche. New York: Free Press; 2010.

98. Ventevogel P, Jordans M, Reis R, de Jong J. Madness or sadness? Local concepts of mental illness in four conflict-affected African communities. Confl Health. 2013 Feb 18;7(1):3.

99. Hom MA, Stanley IH, Joiner Jr TE. Evaluating factors and interventions that influence help-seeking and mental health service utilization among suicidal individuals: A review of the literature -. Clin Psychol Rev. 40:28–39.

100. Wenze SJ, Battle CL. Perinatal Mental Health Treatment Needs, Preferences, and Barriers in Parents of Multiples. J Psychiatr Pract. 2018 May;24(3):158.

For peer review only

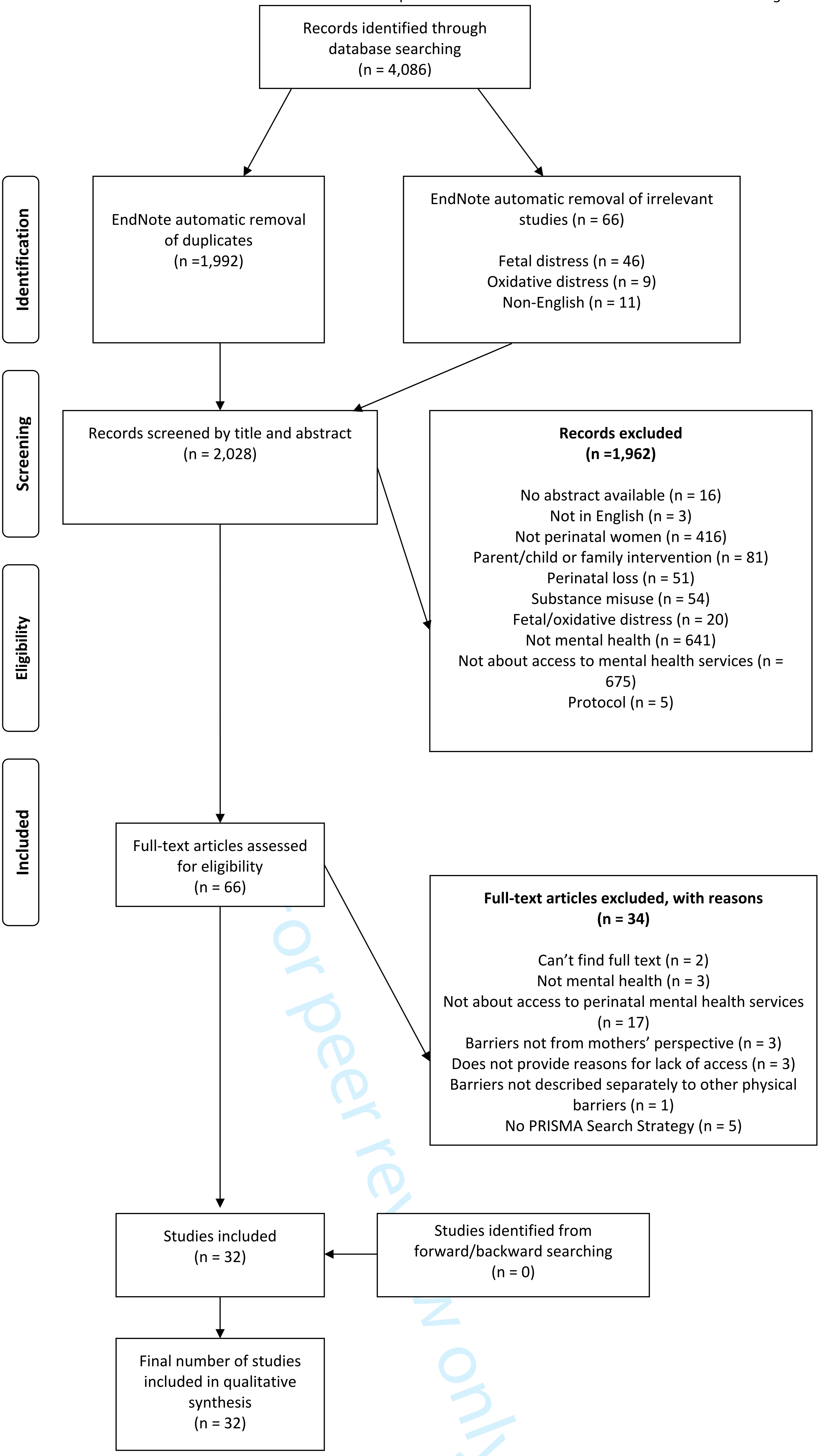

From: Moher D, Liberati A, Tetzlaff J, Altman DG, The PRISMA Group (2009). *P*referred *R*eporting *I*tems for *S*ystematic Reviews and *M*eta-*A*nalyses: The PRISMA Statement. PLoS Med 6(7): e1000097. doi:10.1371/journal.pmed1000097

For more information, visit www.prisma-statement.org.

For peer review only - http://bmjopen.bmj.com/site/about/guidelines.xhtml

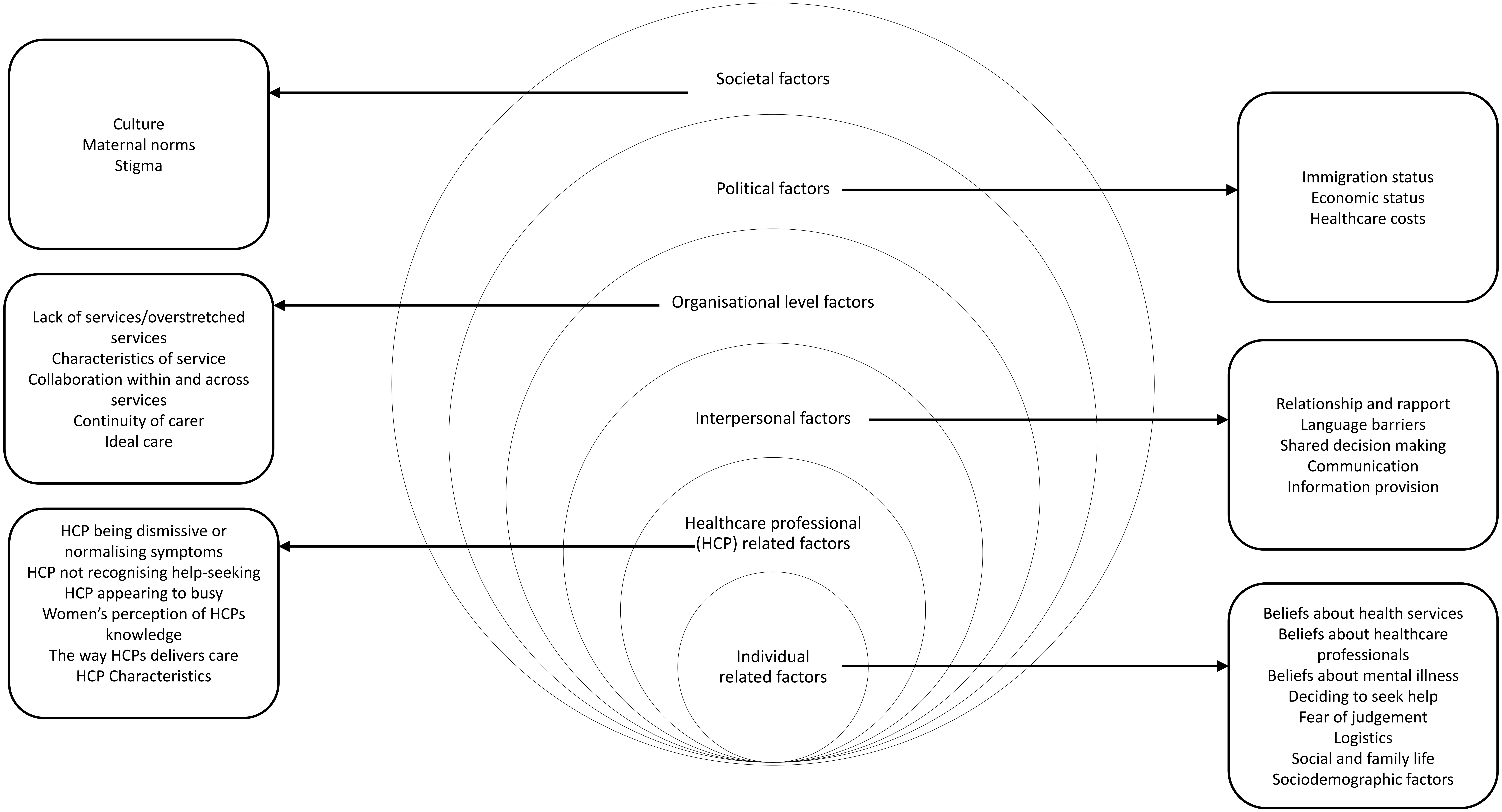
Societal factors
Culture
Maternal norms
Stigma
Political factors
Immigration status
Economic status
Healthcare costs
Organisational level factors
Lack of services/overstretched services
Characteristics of service
Collaboration within and across services
Continuity of carer
Ideal care
Interpersonal factors
Relationship and rapport
Language barriers
Shared decision making
Communication
Information provision
Healthcare professional (HCP) related factors
HCP being dismissive or normalising symptoms
HCP not recognising help-seeking
HCP appearing to busy
Women's perception of HCPs knowledge
The way HCPs delivers care
HCP Characteristics
Individual related factors
Beliefs about health services
Beliefs about healthcare professionals
Beliefs about mental illness
Deciding to seek help
Fear of judgement
Logistics
Social and family life
Sociodemographic factors

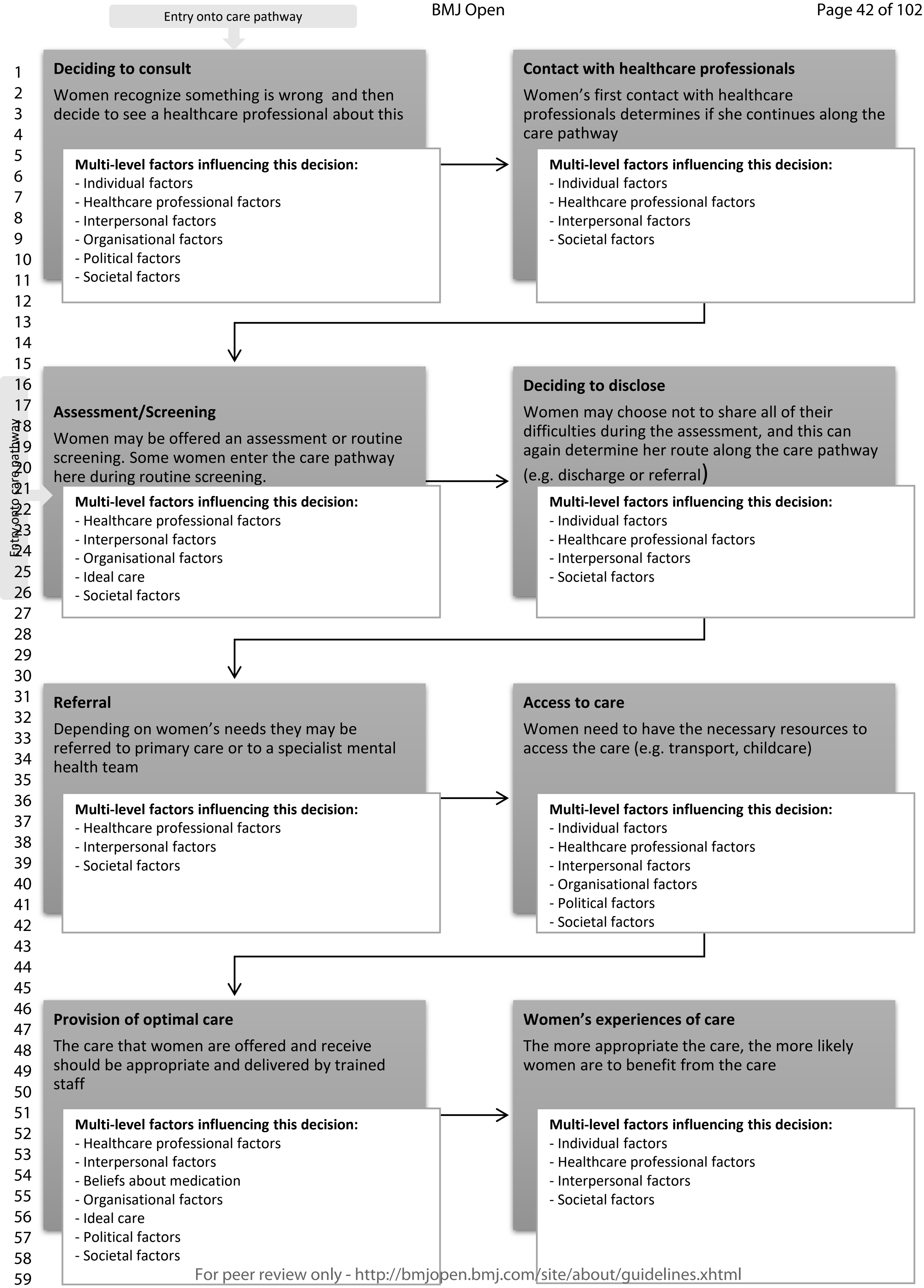

Note. Some parts of the pathway are redundant in health care systems where the woman can contact mental health services directly (e.g. France or via Improving Access to Psychological Therapies services in the UK). Further, the process is not always linear women might jump over certain stages.

A meta-review of the barriers to women accessing perinatal mental health care

Appendices

Contents

Appendix 1: PROSPERO Registration: CRD42019142854 2
Appendix 2: Table 1. Search terms 14
Appendix 3: Table 2. Search Results 28
Appendix 4: Table 3. Inclusion and exclusion criteria 29
Appendix 5: Detailed methodology 30
Appendix 6: Table 4. Sensitivity analysis 33
Appendix 7: *Figure 1.* Synthesis contribution vs quality appraisal criteria met 35
Appendix 8: Sensitivity analysis results 36
Appendix 9: Table 5. Summary review characteristics 37
Appendix 10: Table 6. Characteristics of included reviews 37
Appendix 11: Table 7. Quality appraisal 48
Appendix 12: Table 8. Themes 51
References 56

For peer review only

Appendix 1: PROSPERO Registration: CRD42019142854

PROSPERO
International prospective register of systematic reviews

National Institute for Health Research

UNIVERSITY of York
Centre for Reviews and Dissemination

Systematic review

1. * Review title.

Give the title of the review in English

Meta-review of barriers to women accessing perinatal mental healthcare and treatment

2. Original language title.

For reviews in languages other than English, give the title in the original language. This will be displayed with the English language title.

3. * Anticipated or actual start date.

Give the date the systematic review started or is expected to start.

07/06/2020

4. * Anticipated completion date.

Give the date by which the review is expected to be completed.

28/02/2021

5. * Stage of review at time of this submission.

Tick the boxes to show which review tasks have been started and which have been completed. Update this field each time any amendments are made to a published record.

Reviews that have started data extraction (at the time of initial submission) are not eligible for inclusion in PROSPERO. If there is later evidence that incorrect status and/or completion date has been supplied, the published PROSPERO record will be marked as retracted.

This field uses answers to initial screening questions. It cannot be edited until after registration.

The review has not yet started: No

**Review stage**	**Started**	**Completed**
Preliminary searches	Yes	No
Piloting of the study selection process	Yes	No
Formal screening of search results against eligibility criteria	No	No
Data extraction	No	No
Risk of bias (quality) assessment	No	No
Data analysis	No	No

PROSPERO
International prospective register of systematic reviews

National Institute for Health Research

Provide any other relevant information about the stage of the review here.

6. * Named contact.

The named contact is the guarantor for the accuracy of the information in the register record. This may be any member of the review team.

Rebecca Webb

Email salutation (e.g. "Dr Smith" or "Joanne") for correspondence:

Dr Webb

7. * Named contact email.

Give the electronic email address of the named contact.

Rebecca.Webb.2@city.ac.uk

8. Named contact address

Give the full institutional/organisational postal address for the named contact.

Centre for Maternal and Child Health Research, School of Health Sciences, City, University of London, EC1V 0HB

9. Named contact phone number.

Give the telephone number for the named contact, including international dialling code.

(+44)07810255328

10. * Organisational affiliation of the review.

Full title of the organisational affiliations for this review and website address if available. This field may be completed as 'None' if the review is not affiliated to any organisation.

City, University of London

Organisation web address:

11. * Review team members and their organisational affiliations.

Give the personal details and the organisational affiliations of each member of the review team. Affiliation refers to groups or organisations to which review team members belong. **NOTE: email and country now MUST be entered for each person, unless you are amending a published record.**

Dr Rebecca Webb. City, University of London
Dr Elizabeth Ford. Brighton and Sussex Medical School
Dr Judy Shakespeare. Retired GP
Dr Abigail Easter. King's College London
Professor Simon Gilbody. University of York
Professor Fiona Alderdice. University of Oxford
Dr Nia Roberts. Nuffield Department of Population Health, Bodleian Health Care Libraries
Professor Debra Salmon. City, University of London

Professor Helen Cheyne. University of Stirling
Ms Clare Thompson. Maternal Mental Health Change Agents
Miss Nazihah Uddin. City, University of London
Professor Susan Ayers. City, University of London

12. * Funding sources/sponsors.

Details of the individuals, organizations, groups, companies or other legal entities who have funded or sponsored the review.

NIHR Health Services and Delivery Research Grant

Grant number(s)

State the funder, grant or award number and the date of award

NIHR128068

13. * Conflicts of interest.

List actual or perceived conflicts of interest (financial or academic).

None

14. Collaborators.

Give the name and affiliation of any individuals or organisations who are working on the review but who are not listed as review team members. **NOTE: email and country must be completed for each person, unless you are amending a published record.**

Dr Agnes Hann. National Childbirth Trust
Dr Sarah McMullen. National Childbirth Trust
Dr Rose Coates. City, University of London
Dr Camilla Rosan. Anna Freud National Centre for Children and Families
Dr Sally Hogg. Parent-Infant Partnership UK
Mr Andrea Sinesi. University of Stirling

15. * Review question.

State the review question(s) clearly and precisely. It may be appropriate to break very broad questions down into a series of related more specific questions. Questions may be framed or refined using PI(E)COS or similar where relevant.

What are the individual, interpersonal, organizational, political and social factors that prevent women with perinatal mental health problems accessing care or treatment from the NHS or other health and social care services? A review of reviews.

Objectives are to:

1. Determine the barriers and facilitators to women accessing perinatal mental health care or treatment.

2. Identify differences in barriers and facilitators across different health and social care settings.

3. Evaluate the quality of published reviews.

4. Map the geographical distribution of the evidence to establish generalisability and gaps in the evidence.

16. * Searches.

State the sources that will be searched (e.g. Medline). Give the search dates, and any restrictions (e.g. language or publication date). Do NOT enter the full search strategy (it may be provided as a link or attachment below.)

PROSPERO
International prospective register of systematic reviews

National Institute for Health Research

Systematic searches will be conducted through online databases such as: MEDLINE; PsycINFO; PubMed; Cumulative Index to Nursing and Allied Health Literature (CINAHL); EMBASE; Cochrane Library; Web of Science and Scopus. The Turning Research Into Practice (TRIP) database will also be searched. Other search strategies will include: tracking citations of key papers (forward searching); examining reference lists of key papers (backwards searching). When conducting the searches search terms will be combined using Boolean terms "OR" and "AND".

To identify papers, the following parameters will be used:

• Population: Women in the perinatal period (conception to 1 year postpartum). (Search terms will include, but are not limited to: Perinatal OR postnatal OR postpartum OR antenatal).

• Intervention: Assessment, care or treatment for perinatal mental health (search terms will include: help-seeking OR Screening OR assessment OR service* AND Access* OR implement* OR seeking OR decision OR employ OR treatment seeking OR treatment engagement)

• Outcome: Barriers and facilitators, women's experiences, health and social care professionals' experiences (search terms will include: Barriers OR drawbacks OR obstacles OR issues)

Study design: Review papers where a clear systematic search strategy is used and reported (search terms will include: meta-synthesis* OR meta-ethnograph* OR meta-study)

Papers will be selected in two stages according to PRISMA guidelines. In the first stage, titles and abstracts will be screened by one researcher for relevance to the topic. Papers that are clearly not relevant will be excluded. A random selection will be screened by a second researcher and agreement between the two will be calculated using Cohen's Kappa statistic. In the second stage, full texts for papers that appear to be relevant will be obtained and final selection made by the researcher and project management group. Again, a random selection will be screened by a second researcher and agreement between the two will be calculated.

17. URL to search strategy.

Upload a file with your search strategy, or an example of a search strategy for a specific database, (including the keywords) in pdf or word format. In doing so you are consenting to the file being made publicly accessible. Or provide a URL or link to the strategy. Do NOT provide links to your search **results**.

Alternatively, upload your search strategy to CRD in pdf format. Please note that by doing so you are consenting to the file being made publicly accessible.

Do not make this file publicly available until the review is complete

18. * Condition or domain being studied.

Give a short description of the disease, condition or healthcare domain being studied in your systematic review.

Mental health problems affect up to one in five women during pregnancy and the first year after birth (the perinatal period). These include anxiety, depression, stress-related conditions and adjustment disorders. This costs the UK £8.1 billion for every year of babies born. Mental health problems can have a negative effect on women, their partners and their children. They are also one of the leading causes of maternal death. It is vitally important that women who experience mental health difficulties are identified quickly and get the treatment they need. However, only about half of women with perinatal mental health problems are identified by healthcare services and even fewer receive treatment. We will therefore include the following perinatal mental health problems in our review: depression, anxiety, adjustment disorders, OCD, PTSD, psychosis, eating disorders, personality disorders.

19. * Participants/population.

Specify the participants or populations being studied in the review. The preferred format includes details of both inclusion and exclusion criteria.

The population of interest is women in the perinatal period, who are at risk of, or who are experiencing perinatal mental health problems (including, but not limited to, anxiety, depression, PTSD, adjustment disorders). No restrictions will be placed on women's age or ethnicity. If papers include multiple stakeholder views, we will include reviews where the women's responses can be separated out.

Papers will be excluded if they: are non-English publications; are text or opinion pieces, do not have a PRISMA guided search strategy, include people outside the target population (e.g. men/partners or children) where their views cannot be separated from women's views; include interventions targeted at the parent-infant, couple or family relationships; focus on perinatal loss due to the unique focus of the treatment, or focus on substance misuse which has unique challenges in terms of assessment and treatment, or focus on oxidative stress or fetal distress.

20. * Intervention(s), exposure(s).

Give full and clear descriptions or definitions of the interventions or the exposures to be reviewed. The preferred format includes details of both inclusion and exclusion criteria.

We are interested in reviews that look at women's views and experiences of accessing perinatal mental health care across the treatment pathway from deciding to consult or help seeking and disclosing symptoms to assessment, referral, care and treatment.

21. * Comparator(s)/control.

Where relevant, give details of the alternatives against which the intervention/exposure will be compared (e.g. another intervention or a non-exposed control group). The preferred format includes details of both inclusion and exclusion criteria.

Not applicable

22. * Types of study to be included.

Give details of the study designs (e.g. RCT) that are eligible for inclusion in the review. The preferred format

For peer review only - http://bmjopen.bmj.com/site/about/guidelines.xhtml

includes both inclusion and exclusion criteria. If there are no restrictions on the types of study, this should be stated.

Review papers that have used a search strategy according the PRISMA guidelines, such as systematic reviews, about access to mental health care or treatment by women in the perinatal period across all stages of the care pathway (deciding to consult, contact with healthcare professionals, assessment/screening, deciding to disclose, referral, access to treatment and provision of optimal treatment). Assessment refers to identifying women who may be at risk for perinatal mental health problems, or who have perinatal mental health problems. Care refers to supportive care or care pathways such as that provided by health professionals who provide care to women with perinatal mental health difficulties across the care pathway. Treatment refers to any active intervention, programmes or protocols to reduce women's perinatal mental health symptoms. The perinatal period is defined as from conception to 1 year postpartum. Mental health measures need to be gold standard clinical interviews or validated self-report questionnaires.

23. Context.

Give summary details of the setting or other relevant characteristics, which help define the inclusion or exclusion criteria.

As women move through pregnancy and the postnatal period, they come into contact with different services that provide women with opportunities to disclose any mental health difficulties and access perinatal mental health care. Despite the services available for women with perinatal mental health difficulties, it is estimated that half of women are not identified despite regular routine contact with these healthcare services, and still fewer receive treatment. For example, a study of postnatal depression suggested only 40% of women with postnatal depression were identified, 24% received treatment, 10% received adequate treatment, and only 3-6% of women recovered. This is likely to be due to a range of factors at individual, interpersonal, organisational and social levels, such as healthcare professionals not asking about mental health, lack of effective assessment, barriers to women seeking help or attending treatment, clinician barriers to diagnosis and treatment, lack of services to refer onto, or limited understanding of effective treatments.

Given that women accessing care or treatment might be important in preventing adverse outcomes for women and their families, we need to identify the wide range of factors that may prevent women from accessing these. The literature on why women with perinatal mental health problems do not access care or treatment is varied and some areas are more clearly synthesised than others. At present, there is no clear overview and synthesis of how these factors may operate at different levels, which is why a systematic review of reviews is needed.

24. * Main outcome(s).

Give the pre-specified main (most important) outcomes of the review, including details of how the outcome is defined and measured and when these measurement are made, if these are part of the review inclusion criteria.

PROSPERO
International prospective register of systematic reviews

NHS National Institute for Health Research

The main aim is to determine the individual, interpersonal, organisational and social factors that prevent women accessing care or treatment. Therefore, the main outcome will be evaluation parameters for assessment, care or treatment for perinatal mental health, including barriers, facilitators, women's experiences, and health and social care professionals' experiences.

Measures of effect

Please specify the effect measure(s) for you main outcome(s) e.g. relative risks, odds ratios, risk difference, and/or 'number needed to treat.

Not applicable.

25. * Additional outcome(s).

List the pre-specified additional outcomes of the review, with a similar level of detail to that required for main outcomes. Where there are no additional outcomes please state 'None' or 'Not applicable' as appropriate to the review

None.

Measures of effect

Please specify the effect measure(s) for you additional outcome(s) e.g. relative risks, odds ratios, risk difference, and/or 'number needed to treat.

Not applicable.

26. * Data extraction (selection and coding).

Describe how studies will be selected for inclusion. State what data will be extracted or obtained. State how this will be done and recorded.

Data will be extracted from eligible reviews using a standard data extraction form in Excel or using the data extraction tool on EPPI-Reviewer. If information is missing, corresponding authors will be contacted with a request for the information. If they do not respond within 2 weeks they will be contacted again. If they do not respond within 4 weeks, missing data will not be included.

A range of data will be extracted to enable a comprehensive meta-review. Key variables will include: Authors; Year; Country; Review design; Method; Sample size and characteristics (of parents, of health care provider); Mental health problem; Outcome measures; Type of intervention(s); Methodological quality rating.

27. * Risk of bias (quality) assessment.

State which characteristics of the studies will be assessed and/or any formal risk of bias/quality assessment tools that will be used.

Quality will be assessed using the AMSTAR checklist which is a reliable and valid measure for assessing the methodological quality of systematic reviews. One reviewer will conduct the quality assessments and reliability of these ratings will be checked="checked" value="1" by a second reviewer rating a random selection of 25% of papers. If disagreements arise then all papers will be double-rated, and disagreements resolved through discussion and consensus. Where consensus cannot be reached the project management group will be consulted and make the final decision. If agreement is low, the second rater will look at a larger

group of papers. If meta-analyses are identified, we will assess the quality of the analysis using the Scottish Intercollegiate Guidelines Network (SIGN) evidence grading system. This system grades the risk of bias associated with a particular piece of evidence on a hierarchy from meta-analysis and RCT evidence (grade 1) down to expert opinion (grade 4), with additional indicators (++, + or -) to indicate methodological quality.

28. * Strategy for data synthesis.

Describe the methods you plan to use to synthesise data. This **must not be generic text** but should be **specific to your review** and describe how the proposed approach will be applied to your data. If meta-analysis is planned, describe the models to be used, methods to explore statistical heterogeneity, and software package to be used.

We will follow a similar strategy to McNeill et al (2012). Firstly, a table summarizing the findings will be presented. Within the table we will include the following information: authors/year; aim; search strategy; number of studies included; total number of participants; results (i.e. key barriers and facilitators identified); quality rating. We will then perform a narrative synthesis of reviews identified, discussing both barriers and facilitators to women accessing perinatal mental health assessment, care and treatment across the care pathway. Where quantitative data is included, we will carry out a narrative synthesis where we indicate both the quality of the evidence (low, medium, high) and whether it is causal or associative in nature as done by Greaves et al., (2011).

Greaves, C.J., Sheppard, K.E., Abraham, C. et al. Systematic review of reviews of intervention components associated with increased effectiveness in dietary and physical activity interventions. BMC Public Health 11, 119 (2011). https://doi.org/10.1186/1471-2458-11-119

McNeill, J., Lynn, F. & Alderdice, F. Public health interventions in midwifery: a systematic review of systematic reviews. BMC Public Health 12, 955 (2012). https://doi.org/10.1186/1471-2458-12-955

29. * Analysis of subgroups or subsets.

State any planned investigation of 'subgroups'. Be clear and specific about which type of study or participant will be included in each group or covariate investigated. State the planned analytic approach.

We will examine recommendations for healthcare practice and research separately, including which countries have a sparsity of data and recommendations for quality improvement in research methods.

30. * Type and method of review.

Select the type of review, review method and health area from the lists below.

Type of review

Cost effectiveness
No

Diagnostic
No

Epidemiologic
No

PROSPERO
International prospective register of systematic reviews

National Institute for Health Research

Individual patient data (IPD) meta-analysis
No

Intervention
No

Living systematic review
No

Meta-analysis
No

Methodology
No

Narrative synthesis
Yes

Network meta-analysis
No

Pre-clinical
No

Prevention
No

Prognostic
No

Prospective meta-analysis (PMA)
No

Review of reviews
Yes

Service delivery
No

Synthesis of qualitative studies
Yes

Systematic review
Yes

Other
No

Health area of the review

Alcohol/substance misuse/abuse
No

Blood and immune system
No

Cancer
No

Cardiovascular
No

Care of the elderly

PROSPERO
International prospective register of systematic reviews

National Institute for Health Research

No

Child health
No

Complementary therapies
No

COVID-19
No

Crime and justice
No

Dental
No

Digestive system
No

Ear, nose and throat
No

Education
No

Endocrine and metabolic disorders
No

Eye disorders
No

General interest
No

Genetics
No

Health inequalities/health equity
No

Infections and infestations
No

International development
No

Mental health and behavioural conditions
Yes

Musculoskeletal
No

Neurological
No

Nursing
No

Obstetrics and gynaecology
No

Oral health
No

Palliative care
No

Perioperative care
No

Physiotherapy
No

Pregnancy and childbirth
Yes

Public health (including social determinants of health)
No

Rehabilitation
No

Respiratory disorders
No

Service delivery
No

Skin disorders
No

Social care
No

Surgery
No

Tropical Medicine
No

Urological
No

Wounds, injuries and accidents
No

Violence and abuse
No

31. Language.

Select each language individually to add it to the list below, use the bin icon to remove any added in error.
English

There is not an English language summary

32. * Country.

Select the country in which the review is being carried out. For multi-national collaborations select all the countries involved.

England

33. Other registration details.

Name any other organisation where the systematic review title or protocol is registered (e.g. Campbell, or

The Joanna Briggs Institute) together with any unique identification number assigned by them. If extracted data will be stored and made available through a repository such as the Systematic Review Data Repository (SRDR), details and a link should be included here. If none, leave blank.

34. Reference and/or URL for published protocol.

If the protocol for this review is published provide details (authors, title and journal details, preferably in Vancouver format)

Add web link to the published protocol.

Or, upload your published protocol here in pdf format. Note that the upload will be publicly accessible.

No I do not make this file publicly available until the review is complete

Please note that the information required in the PROSPERO registration form must be completed in full even if access to a protocol is given.

35. Dissemination plans.

Do you intend to publish the review on completion?

Yes

Give brief details of plans for communicating review findings.?

We plan to publish this review in a high impact peer reviewed journal.

36. Keywords.

Give words or phrases that best describe the review. Separate keywords with a semicolon or new line. Keywords help PROSPERO users find your review (keywords do not appear in the public record but are included in searches). Be as specific and precise as possible. Avoid acronyms and abbreviations unless these are in wide use.

Perinatal mental health; Barriers; Access; Healthcare; Treatment

37. Details of any existing review of the same topic by the same authors.

If you are registering an update of an existing review give details of the earlier versions and include a full bibliographic reference, if available.

38. * Current review status.

Update review status when the review is completed and when it is published.New registrations must be ongoing so this field is not editable for initial submission.
Please provide anticipated publication date

Review_Ongoing

39. Any additional information.

Provide any other information relevant to the registration of this review.

40. Details of final report/publication(s) or preprints if available.

Leave empty until publication details are available OR you have a link to a preprint (NOTE: this field is not editable for initial submission). List authors, title and journal details preferably in Vancouver format.

Give the link to the published review or preprint.

Appendix 2: Table 1. Search terms

	MEDLINE (1946-present)
**# ▲**	**Searches**
1	prenatal care/ or perinatal care/ or postnatal care/
2	Pregnancy/
3	Pregnant Women/
4	(pregnancy or pregnant or pre-nat* or prenat* or prepart* or prepart* or ante-nat* or antenat* or ante-part* or antepart* or peri-nat* or perinat* or peri-part* or peripart* or puerper* or post-nat* or postnat* or post-part* or postpart*).ti.
5	((parent? or mother* or maternal or father* or paternal) and (infan* or newborn? or neonat* or baby or babies)).ti.
6	1 or 2 or 3 or 4 or 5
7	mental disorders/ or exp anxiety disorders/ or exp mood disorders/ or exp "trauma and stressor related disorders"/
8	Stress, Psychological/
9	Adaptation, Psychological/
10	(mental* or psych* or anxiety or anxious or depress* or mood? or affect* or distress* or stress or trauma* or posttrauma* or post-trauma* or adjustment disorder* or phobia* or phobic or obsessive compulsive or wellbeing or well-being).ti.
11	7 or 8 or 9 or 10
12	6 and 11
13	Depression, Postpartum/
14	Pregnant Women/px [Psychology]
15	((pregnancy or pregnant or pre-nat* or prenat* or prepart* or prepart* or ante-nat* or antenat* or ante-part* or antepart* or peri-nat* or perinat* or peri-part* or peripart* or puerper* or post-nat* or postnat* or post-part* or postpart*) adj5 (mental* or psych* or anxiety or anxious or depress* or mood? or affect* or distress* or stress or trauma* or posttrauma* or post-trauma* or adjustment disorder* or phobia* or phobic or obsessive compulsive or wellbeing or well-being)).ti,ab.
16	(((parent? or mother* or maternal or father* or paternal) adj5 (infan* or newborn? or neonat* or baby or babies)) and (mental* or psych* or anxiety or anxious or depress* or mood? or affect* or distress* or stress or trauma* or posttrauma* or post-trauma* or adjustment disorder* or phobia* or phobic or obsessive compulsive or wellbeing or well-being)).ti,ab.

For peer review only - http://bmjopen.bmj.com/site/about/guidelines.xhtml

17	12 or 13 or 14 or 15 or 16
18	Mass Screening/
19	diagnosis/ or early diagnosis/
20	(screen* or detect* or diagnos* or assess* or identifi* or prevent* or prophyla*).ti.
21	psychotherapy/ or behavior therapy/ or exp cognitive behavioral therapy/
22	counseling/ or exp directive counseling/
23	exp antidepressive agents/ or exp anti-anxiety agents/
24	(intervention? or counsel* or therap* or healing or listen* support* or care or healthcare or service or medication* or drug? or antidepress* or anti-depress* or antianxiety or anti-anxiety).ti.
25	("improving access to psychological therap*" or iapt).ti,ab.
26	((mental* or psych* or anxiety or anxious or depress* or mood? or affect* or distress* or stress or trauma* or posttrauma* or post-trauma* or adjustment disorder* or phobia* or phobic or obsessive compulsive or wellbeing or well-being) adj5 (screen* or detect* or diagnos* or assess* or identifi* or prevent* or prophyla*)).ti,ab.
27	((mental* or psych* or anxiety or anxious or depress* or mood? or affect* or distress* or stress or trauma* or posttrauma* or post-trauma* or adjustment disorder* or phobia* or phobic or obsessive compulsive or wellbeing or well-being) adj5 (intervention? or counsel* or therap* or healing or listen* support* or care or healthcare or service or medication* or drug? or antidepress* or anti-depress* or antianxiety or anti-anxiety)).ti,ab.
28	18 or 19 or 20 or 21 or 22 or 23 or 24 or 25 or 26 or 27
29	17 and 28
30	Depression, Postpartum/di, dh, dt, pc, th
31	29 or 30
32	Implementation Science/ or Health Plan Implementation/
33	Program Evaluation/
34	(implement* or impact*).ti,ab.
35	(feasib* or acceptab*).ti,ab.
36	(barrier? or challenge? or obstacle? or hurdle? or obstruct* or drawback? or issue? or difficult? or promot* or support or encourag* or factor? or facilitat* or enabl* or opportunit* or engage* or assist*).ti,ab.
37	((process or project* or system*) adj5 evaluat*).ti,ab.
38	32 or 33 or 34 or 35 or 36 or 37

For peer review only - http://bmjopen.bmj.com/site/about/guidelines.xhtml

39	31 and 38	
40	medline.ti,ab.	
41	systematic review.pt.	
42	meta-analysis.pt.	
43	systematic review.ti,ab.	
44	(evidence synthesis or realist synthesis or realist review).ti,ab.	
45	(Qualitative and synthesis).ti,ab.	
46	(meta-synthesis* or meta synthesis* or metasynthesis).ti,ab.	
47	(meta-ethnograph* or metaethnograph* or meta ethnograph*).ti,ab.	
48	(meta-study or metastudy or meta study).ti,ab.	
49	40 or 41 or 42 or 43 or 44 or 45 or 46 or 47 or 48	
50	39 and 49	
51	(comment or editorial or letter or historical article).pt.	
52	50 not 51	
53	exp animals/ not humans/	
54	52 not 53	
55	limit 54 to english language	
	**EMBASE (1974 – present)**	
1	prenatal care/ or newborn period/ or perinatal period/ or prenatal period/	
2	*Pregnancy/	
3	(pregnancy or pregnant or pre-nat* or prenat* or prepart* or prepart* or ante-nat* or antenat* or ante-part* or antepart* or peri-nat* or perinat* or peri-part* or peripart* or puerper* or post-nat* or postnat* or post-part* or postpart*).ti.	
4	((parent? or mother* or maternal or father* or paternal) and (infan* or newborn? or neonat* or baby or babies)).ti.	
5	1 or 2 or 3 or 4	
6	mental disease/ or exp anxiety disorder/ or exp mood disorder/	
7	mental stress/	
8	(mental* or psych* or anxiety or anxious or depress* or mood? or affect* or distress* or stress or trauma* or posttrauma* or post-trauma* or adjustment disorder* or phobia* or phobic or obsessive compulsive or wellbeing or well-being).ti.	
9	6 or 7 or 8	

For peer review only - http://bmjopen.bmj.com/site/about/guidelines.xhtml

10	5 and 9
11	exp perinatal depression/
12	((pregnancy or pregnant or pre-nat* or prenat* or prepart* or prepart* or ante-nat* or antenat* or ante-part* or antepart* or peri-nat* or perinat* or peri-part* or peripart* or puerper* or post-nat* or postnat* or post-part* or postpart*) adj5 (mental* or psych* or anxiety or anxious or depress* or mood? or affect* or distress* or stress or trauma* or posttrauma* or post-trauma* or adjustment disorder* or phobia* or phobic or obsessive compulsive or wellbeing or well-being)).ti,ab.
13	(((parent? or mother* or maternal or father* or paternal) adj5 (infan* or newborn? or neonat* or baby or babies)) and (mental* or psych* or anxiety or anxious or depress* or mood? or affect* or distress* or stress or trauma* or posttrauma* or post-trauma* or adjustment disorder* or phobia* or phobic or obsessive compulsive or wellbeing or well-being)).ti,ab.
14	10 or 11 or 12 or 13
15	mass screening/ or screening test/ or screening/
16	diagnosis/ or early diagnosis/
17	(screen* or detect* or diagnos* or assess* or identifi* or prevent* or prophyla*).ti.
18	exp counseling/ or early intervention/ or exp psychotherapy/
19	(intervention? or counsel* or therap* or healing or listen* support* or care or healthcare or service or medication* or drug? or antidepress* or anti-depress* or antianxiety or anti-anxiety).ti.
20	("improving access to psychological therap*" or iapt).ti,ab.
21	((mental* or psych* or anxiety or anxious or depress* or mood? or affect* or distress* or stress or trauma* or posttrauma* or post-trauma* or adjustment disorder* or phobia* or phobic or obsessive compulsive or wellbeing or well-being) adj5 (screen* or detect* or diagnos* or assess* or identifi* or prevent* or prophyla*)).ti,ab.
22	((mental* or psych* or anxiety or anxious or depress* or mood? or affect* or distress* or stress or trauma* or posttrauma* or post-trauma* or adjustment disorder* or phobia* or phobic or obsessive compulsive or wellbeing or well-being) adj5 (intervention? or counsel* or therap* or healing or listen* support* or care or healthcare or service or medication* or drug? or antidepress* or anti-depress* or antianxiety or anti-anxiety)).ti,ab.
23	15 or 16 or 17 or 18 or 19 or 20 or 21 or 22
24	14 and 23
25	exp perinatal depression/di, dt, pc, th
26	24 or 25

For peer review only - http://bmjopen.bmj.com/site/about/guidelines.xhtml

27	therapy delay/
28	exp Program Evaluation/ or Implementation Science/
29	(implement* or impact*).ti,ab.
30	(feasib* or acceptab*).ti,ab.
31	(barrier? or challenge? or obstacle? or hurdle? or obstruct* or drawback? or issue? or difficult? or promot* or support or encourag* or factor? or facilitat* or enabl* or opportunit* or engage* or assist*).ti,ab.
32	((process or project* or system*) adj5 evaluat*).ti,ab.
33	27 or 28 or 29 or 30 or 31 or 32
34	26 and 33
35	medline.tw.
36	"systematic review"/
37	meta-analysis/
38	systematic review.ti,ab.
39	(evidence synthesis or realist synthesis or realist review).ti,ab.
40	(Qualitative and synthesis).ti,ab.
41	(meta-synthesis* or meta synthesis* or metasynthesis).ti,ab.
42	(meta-ethnograph* or metaethnograph* or meta ethnograph*).ti,ab.
43	(meta-study or metastudy or meta study).ti,ab.
44	35 or 36 or 37 or 38 or 39 or 40 or 41 or 42 or 43
45	34 and 44
46	(editorial or letter or note or conference*).pt.
47	45 not 46
48	(exp animals/ or nonhuman/) not human/
49	47 not 48
50	limit 49 to english language
**PSYCHINFO (1806 – present)**	
1	prenatal care/ or postnatal period/ or antepartum period/ or intrapartum period/ or perinatal period/
2	Pregnancy/

For peer review only - http://bmjopen.bmj.com/site/about/guidelines.xhtml

3	(pregnancy or pregnant or pre-nat* or prenat* or prepart* or prepart* or ante-nat* or antenat* or ante-part* or antepart* or peri-nat* or perinat* or peri-part* or peripart* or puerper* or post-nat* or postnat* or post-part* or postpart*).ti.
4	((parent? or mother* or maternal or father* or paternal) and (infan* or newborn? or neonat* or baby or babies)).ti.
5	1 or 2 or 3 or 4
6	mental disorders/ or exp affective disorders/ or exp anxiety disorders/ or exp "stress and trauma related disorders"/
7	psychological stress/
8	Emotional Adjustment/
9	(mental* or psych* or anxiety or anxious or depress* or mood? or affect* or distress* or stress or trauma* or posttrauma* or post-trauma* or adjustment disorder* or phobia* or phobic or obsessive compulsive or wellbeing or well-being).ti.
10	6 or 7 or 8 or 9
11	5 and 10
12	postpartum depression/ or postpartum psychosis/
13	((pregnancy or pregnant or pre-nat* or prenat* or prepart* or prepart* or ante-nat* or antenat* or ante-part* or antepart* or peri-nat* or perinat* or peri-part* or peripart* or puerper* or post-nat* or postnat* or post-part* or postpart*) adj5 (mental* or psych* or anxiety or anxious or depress* or mood? or affect* or distress* or stress or trauma* or posttrauma* or post-trauma* or adjustment disorder* or phobia* or phobic or obsessive compulsive or wellbeing or well-being)).ti,ab.
14	(((parent? or mother* or maternal or father* or paternal) adj5 (infan* or newborn? or neonat* or baby or babies)) and (mental* or psych* or anxiety or anxious or depress* or mood? or affect* or distress* or stress or trauma* or posttrauma* or post-trauma* or adjustment disorder* or phobia* or phobic or obsessive compulsive or wellbeing or well-being)).ti,ab.
15	11 or 12 or 13 or 14
16	screening/ or exp health screening/ or exp screening tests/
17	diagnosis/
18	(screen* or detect* or diagnos* or assess* or identifi* or prevent* or prophyla*).ti.
19	treatment/ or exp cognitive behavior therapy/ or exp cognitive techniques/ or exp counseling/ or mindfulness-based interventions/ or exp psychotherapy/
20	(intervention? or counsel* or therap* or healing or listen* support* or care or healthcare or service or medication* or drug? or antidepress* or anti-depress* or antianxiety or anti-anxiety).ti.
21	("improving access to psychological therap*" or iapt).ti,ab.

For peer review only - http://bmjopen.bmj.com/site/about/guidelines.xhtml

22	((mental* or psych* or anxiety or anxious or depress* or mood? or affect* or distress* or stress or trauma* or posttrauma* or post-trauma* or adjustment disorder* or phobia* or phobic or obsessive compulsive or wellbeing or well-being) adj5 (screen* or detect* or diagnos* or assess* or identifi* or prevent* or prophyla*)).ti,ab.
23	((mental* or psych* or anxiety or anxious or depress* or mood? or affect* or distress* or stress or trauma* or posttrauma* or post-trauma* or adjustment disorder* or phobia* or phobic or obsessive compulsive or wellbeing or well-being) adj5 (intervention? or counsel* or therap* or healing or listen* support* or care or healthcare or service or medication* or drug? or antidepress* or anti-depress* or antianxiety or anti-anxiety)).ti,ab.
24	16 or 17 or 18 or 19 or 20 or 21 or 22 or 23
25	15 and 24
26	treatment barriers/
27	exp Program Evaluation/
28	(implement* or impact*).ti,ab.
29	(feasib* or acceptab*).ti,ab.
30	(barrier? or challenge? or obstacle? or hurdle? or obstruct* or drawback? or issue? or difficult? or promot* or support or encourag* or factor? or facilitat* or enabl* or opportunit* or engage* or assist*).ti,ab.
31	((process or project* or system*) adj5 evaluat*).ti,ab.
32	26 or 27 or 28 or 29 or 30 or 31
33	25 and 32
34	medline.ti,ab.
35	exp "Systematic Review"/
36	Meta Analysis/
37	systematic review.ti,ab.
38	(evidence synthesis or realist synthesis or realist review).ti,ab.
39	(Qualitative and synthesis).ti,ab.
40	(meta-synthesis* or meta synthesis* or metasynthesis).ti,ab.
41	(meta-ethnograph* or metaethnograph* or meta ethnograph*).ti,ab.
42	(meta-study or metastudy or meta study).ti,ab.
43	34 or 35 or 36 or 37 or 38 or 39 or 40 or 41 or 42
44	33 and 43

For peer review only - http://bmjopen.bmj.com/site/about/guidelines.xhtml

For peer review only

45	(comment reply or editorial or letter or "review book" or "review media" or "review software other").dt.
46	44 not 45
47	limit 46 to english language
	**CINAHL (1982 – present)**
S30	S28 NOT S29
S29	S23 AND S27 Limiters - English Language; Publication Type: Book Review, Commentary, Editorial, Letter
S28	S23 AND S27
S27	S24 OR S25 OR S26
S26	TX ("evidence synthesis" or "realist synthesis" or "realist review") OR TX (Qualitative and synthesis) OR TX ((meta-synthesis* or "meta synthesis*" or metasynthesis) OR TX (meta-ethnograph* or metaethnograph* or "meta ethnograph*") OR TX (meta-study or metastudy or "meta study")
S25	TI (medline or "systematic review") OR AB (medline or "systematic review")
S24	(MH "Systematic Review") OR (MH "Meta Analysis") OR (MH "Meta Synthesis")
S23	S19 AND S22
S22	S20 OR S21
S21	((implement* or impact*)) OR ((implement* or impact*)) OR ((feasib* or acceptab*)) OR ((feasib* or acceptab*)) OR ((barrier? or challenge? or obstacle? or hurdle? or obstruct* or drawback? or issue? or difficult? or promot* or support or encourag* or factor? or facilitat* or enabl* or opportunit* or engage* or assist*)) OR ((barrier? or challenge? or obstacle? or hurdle? or obstruct* or drawback? or issue? or difficult? or promot* or support or encourag* or factor? or facilitat* or enabl* or opportunit* or engage* or assist*)) OR (((process or project* or system*) N5 evaluat*)) OR (((process or project* or system*) N5 evaluat*))
S20	(MH "Implementation Science") OR (MH "Program Development+")
S19	S17 OR S18
S18	(MH "Depression, Postpartum/DI/DH/DT/PC/TH") OR (MH "Postpartum Psychosis/DI/DH/DT/TH/PC")
S17	S11 AND S16
S16	S12 OR S13 OR S14 OR S15

For peer review only - http://bmjopen.bmj.com/site/about/guidelines.xhtml

S15	TI ((intervention? or counsel* or therap* or healing or listen* support* or care or healthcare or service or medication* or drug? or antidepress* or anti-depress* or antianxiety or anti-anxiety)) OR TI (("improving access to psychological therap*" or iapt)) OR AB (("improving access to psychological therap*" or iapt)) OR TI (((mental* or psych* or anxiety or anxious or depress* or mood? or affect* or distress* or stress or trauma* or posttrauma* or post-trauma* or adjustment disorder* or phobia* or phobic or obsessive compulsive or wellbeing or well-being) N5 (screen* or detect* or diagnos* or assess* or identifi* or prevent* or prophyla*))) OR AB (((mental* or psych* or anxiety or anxious or depress* or mood? or affect* or distress* or stress or trauma* or posttrauma* or post-trauma* or adjustment disorder* or phobia* or phobic or obsessive compulsive or wellbeing or well-being) N5 (screen* or detect* or diagnos* or assess* or identifi* or prevent* or prophyla*))) OR TI (((mental* or psych* or anxiety or anxious or depress* or mood? or affect* or distress* or stress or trauma* or posttrauma* or post-trauma* or adjustment disorder* or phobia* or phobic or obsessive compulsive or wellbeing or well-being) N5 (intervention? or counsel* or therap* or healing or listen* support* or care or healthcare or service or medication* or drug? or antidepress* or anti-depress* or antianxiety or anti-anxiety))) OR AB (((mental* or psych* or anxiety or anxious or depress* or mood? or affect* or distress* or stress or trauma* or posttrauma* or post-trauma* or adjustment disorder* or phobia* or phobic or obsessive compulsive or wellbeing or well-being) N5 (intervention? or counsel* or therap* or healing or listen* support* or care or healthcare or service or medication* or drug? or antidepress* or anti-depress* or antianxiety or anti-anxiety)))
S14	(MH "Antidepressive Agents+")
S13	(MH "Psychotherapy+") OR (MH "Cognitive Therapy+") OR (MH "Counseling+")
S12	(MH "Diagnosis") OR (MH "Early Diagnosis") OR (MH "Health Screening")
S11	S8 OR S9 OR S10

For peer review only

For peer review only - http://bmjopen.bmj.com/site/about/guidelines.xhtml

S10	TI (((pregnancy or pregnant or pre-nat* or prenat* or prepart* or prepart* or ante-nat* or antenat* or ante-part* or antepart* or peri-nat* or perinat* or peri-part* or peripart* or puerper* or post-nat* or postnat* or post-part* or postpart*) N5 (mental* or psych* or anxiety or anxious or depress* or mood? or affect* or distress* or stress or trauma* or posttrauma* or post-trauma* or "adjustment disorder*" or phobia* or phobic or "obsessive compulsive" or wellbeing or well-being))) OR AB (((pregnancy or pregnant or pre-nat* or prenat* or prepart* or prepart* or ante-nat* or antenat* or ante-part* or antepart* or peri-nat* or perinat* or peri-part* or peripart* or puerper* or post-nat* or postnat* or post-part* or postpart*) N5 (mental* or psych* or anxiety or anxious or depress* or mood? or affect* or distress* or stress or trauma* or posttrauma* or post-trauma* or "adjustment disorder*" or phobia* or phobic or "obsessive compulsive" or wellbeing or well-being))) OR TI ((((parent? or mother* or maternal or father* or paternal) N5 (infan* or newborn? or neonat* or baby or babies)) and (mental* or psych* or anxiety or anxious or depress* or mood? or affect* or distress* or stress or trauma* or posttrauma* or post-trauma* or "adjustment disorder*" or phobia* or phobic or "obsessive compulsive" or wellbeing or well-being))) AND AB ((((parent? or mother* or maternal or father* or paternal) N5 (infan* or newborn? or neonat* or baby or babies)) and (mental* or psych* or anxiety or anxious or depress* or mood? or affect* or distress* or stress or trauma* or posttrauma* or post-trauma* or "adjustment disorder*" or phobia* or phobic or "obsessive compulsive" or wellbeing or well-being)))
S9	(MH "Depression, Postpartum") OR (MH "Postpartum Psychosis") OR (MH "Expectant Mothers/PF")
S8	S4 AND S7
S7	S5 OR S6
S6	TI mental* or psych* or anxiety or anxious or depress* or mood? or affect* or distress* or stress or trauma* or posttrauma* or post-trauma* or adjustment disorder* or phobia* or phobic or obsessive compulsive or wellbeing or well-being
S5	(MH "Mental Disorders") OR (MH "Anxiety Disorders+") OR (MH "Affective Disorders+") OR (MH "Stress Disorders, Post-Traumatic+") OR (MH "Adaptation, Psychological")
S4	S1 OR S2 OR S3
S3	TI ((pregnancy or pregnant or pre-nat* or prenat* or prepart* or prepart* or ante-nat* or antenat* or ante-part* or antepart* or peri-nat* or perinat* or peri-part* or peripart* or puerper* or post-nat* or postnat* or post-part* or postpart*) OR TI (((parent? or mother* or maternal or father* or paternal) and (infan* or newborn? or neonat* or baby or babies)))
S2	(MH "Expectant Mothers")
S1	(MH "Prenatal Care") OR (MH "Postnatal Period") OR (MH "Pregnancy") OR (MH "Puerperium")
	**SCOPUS**

For peer review only - http://bmjopen.bmj.com/site/about/guidelines.xhtml

11	(((TITLE (pregnancy OR pregnant OR pre-nat* OR prenat* OR prepart* OR prepart* OR ante-nat* OR antenat* OR ante-part* OR antepart* OR peri-nat* OR perinat* OR peri-part* OR peripart* OR puerper* OR post-nat* OR postnat* OR post-part* OR postpart*) AND TITLE (mental* OR psych* OR anxiety OR anxious OR depress* OR mood* OR affect* OR distress* OR stress OR trauma* OR posttrauma* OR post-trauma* OR {adjustment disorder*} OR phobia* OR phobic OR {obsessive compulsive} OR wellbeing OR well-being))) AND ((TITLE-ABS-KEY ((screen* OR detect* OR diagnos* OR assess* OR identifi* OR prevent* OR prophyla*)) OR TITLE-ABS-KEY ((intervention* OR counsel* OR therap* OR healing OR listen* AND support* OR care OR healthcare OR service OR medication* OR drugs OR drug OR antidepress* OR anti-depress* OR antianxiety OR anti-anxiety)) OR TITLE-ABS-KEY (("improving access to psychological therap*" OR iapt)))) AND ((TITLE-ABS-KEY (implement* OR impact* OR feasib* OR acceptab*) OR TITLE-ABS-KEY ((barrier* OR challenge* OR obstacle* OR hurdle* OR obstruct* OR drawback* OR issue* OR difficult* OR promot* OR support OR encourag* OR factor? OR facilitat* OR enabl* OR opportunit* OR engage* OR assist*)) OR TITLE-ABS-KEY ("process evaluation*" OR "project evaluation*" OR "systems evaluation*" OR "system evaluation*")))) AND ((TITLE-ABS-KEY ("systematic review" OR meta-analys*) OR TITLE-ABS-KEY ("evidence synthesis" OR "realist synthesis" OR "realist review" OR meta-synthesis* OR "meta synthesis" OR metasynthesis OR meta-ethnograph* OR metaethnograph* OR "meta ethnograph*" OR meta-study OR metastudy OR "meta study") OR TITLE-ABS-KEY (qualitative AND synthesis))) AND (LIMIT-TO (LANGUAGE , "English"))
10	(((TITLE (pregnancy OR pregnant OR pre-nat* OR prenat* OR prepart* OR prepart* OR ante-nat* OR antenat* OR ante-part* OR antepart* OR peri-nat* OR perinat* OR peri-part* OR peripart* OR puerper* OR post-nat* OR postnat* OR post-part* OR postpart*) AND TITLE (mental* OR psych* OR anxiety OR anxious OR depress* OR mood* OR affect* OR distress* OR stress OR trauma* OR posttrauma* OR post-trauma* OR {adjustment disorder*} OR phobia* OR phobic OR {obsessive compulsive} OR wellbeing OR well-being))) AND ((TITLE-ABS-KEY ((screen* OR detect* OR diagnos* OR assess* OR identifi* OR prevent* OR prophyla*)) OR TITLE-ABS-KEY ((intervention* OR counsel* OR therap* OR healing OR listen* AND support* OR care OR healthcare OR service OR medication* OR drugs OR drug OR antidepress* OR anti-depress* OR antianxiety OR anti-anxiety)) OR TITLE-ABS-KEY (("improving access to psychological therap*" OR iapt)))) AND ((TITLE-ABS-KEY (implement* OR impact* OR feasib* OR acceptab*) OR TITLE-ABS-KEY ((barrier* OR challenge* OR obstacle* OR hurdle* OR obstruct* OR drawback* OR issue* OR difficult* OR promot* OR support OR encourag* OR factor? OR facilitat* OR enabl* OR opportunit* OR engage* OR assist*)) OR TITLE-ABS-KEY ("process evaluation*" OR "project evaluation*" OR "systems evaluation*" OR "system evaluation*")))) AND ((TITLE-ABS-KEY ("systematic review" OR meta-analys*) OR TITLE-ABS-KEY ("evidence synthesis" OR "realist synthesis" OR "realist

For peer review only - http://bmjopen.bmj.com/site/about/guidelines.xhtml

	review" OR meta-synthesis* OR "meta synthesis" OR metasynthesis OR meta-ethnograph* OR metaethnograph* OR "meta ethnograph*" OR meta-study OR metastudy OR "meta study") OR TITLE-ABS-KEY (qualitative AND synthesis)))
9	(TITLE-ABS-KEY ("systematic review" OR meta-analys*) OR TITLE-ABS-KEY ("evidence synthesis" OR "realist synthesis" OR "realist review" OR meta-synthesis* OR "meta synthesis" OR metasynthesis OR meta-ethnograph* OR metaethnograph* OR "meta ethnograph*" OR meta-study OR metastudy OR "meta study") OR TITLE-ABS-KEY (qualitative AND synthesis))
8	((TITLE (pregnancy OR pregnant OR pre-nat* OR prenat* OR prepart* OR prepart* OR ante-nat* OR antenat* OR ante-part* OR antepart* OR peri-nat* OR perinat* OR peri-part* OR peripart* OR puerper* OR post-nat* OR postnat* OR post-part* OR postpart*) AND TITLE (mental* OR psych* OR anxiety OR anxious OR depress* OR mood* OR affect* OR distress* OR stress OR trauma* OR posttrauma* OR post-trauma* OR {adjustment disorder*} OR phobia* OR phobic OR {obsessive compulsive} OR wellbeing OR well-being))) AND ((TITLE-ABS-KEY ((screen* OR detect* OR diagnos* OR assess* OR identifi* OR prevent* OR prophyla*)) OR TITLE-ABS-KEY ((intervention* OR counsel* OR therap* OR healing OR listen* AND support* OR care OR healthcare OR service OR medication* OR drugs OR drug OR antidepress* OR anti-depress* OR antianxiety OR anti-anxiety)) OR TITLE-ABS-KEY (("improving access to psychological therap*" OR iapt)))) AND ((TITLE-ABS-KEY (implement* OR impact* OR feasib* OR acceptab*) OR TITLE-ABS-KEY ((barrier* OR challenge* OR obstacle* OR hurdle* OR obstruct* OR drawback* OR issue* OR difficult* OR promot* OR support OR encourag* OR factor? OR facilitat* OR enabl* OR opportunit* OR engage* OR assist*)) OR TITLE-ABS-KEY ("process evaluation*" OR "project evaluation*" OR "systems evaluation*" OR "system evaluation*")))
7	(TITLE-ABS-KEY (implement* OR impact* OR feasib* OR acceptab*) OR TITLE-ABS-KEY ((barrier* OR challenge* OR obstacle* OR hurdle* OR obstruct* OR drawback* OR issue* OR difficult* OR promot* OR support OR encourag* OR factor? OR facilitat* OR enabl* OR opportunit* OR engage* OR assist*)) OR TITLE-ABS-KEY ("process evaluation*" OR "project evaluation*" OR "systems evaluation*" OR "system evaluation*"))
	**COCHRANE**
#1	MeSH descriptor: [Prenatal Care] explode all trees
#2	MeSH descriptor: [Perinatal Care] explode all trees

For peer review only - http://bmjopen.bmj.com/site/about/guidelines.xhtml

#3	MeSH descriptor: [Pregnancy] this term only
#4	MeSH descriptor: [Pregnant Women] explode all trees
#5	(pregnancy or pregnant or pre-nat* or prenat* or prepart* or prepart* or ante-nat* or antenat* or ante-part* or antepart* or peri-nat* or perinat* or peri-part* or peripart* or puerper* or post-nat* or postnat* or post-part* or postpart*):ti OR (((parent* or mother* or maternal or father* or paternal) and (infan* or newborn* or neonat* or baby or babies))):ti
#6	#1 or #2 or #3 or #4 or #5
#7	MeSH descriptor: [Mental Disorders] this term only
#8	MeSH descriptor: [Anxiety Disorders] explode all trees
#9	MeSH descriptor: [Mood Disorders] explode all trees
#10	MeSH descriptor: [Trauma and Stressor Related Disorders] explode all trees
#11	MeSH descriptor: [Stress, Psychological] explode all trees
#12	MeSH descriptor: [Adaptation, Psychological] this term only
#13	(mental* or psych* or anxiety or anxious or depress* or mood* or affect* or distress* or stress or trauma* or posttrauma* or post-trauma* or adjustment disorder* or phobia* or phobic or obsessive compulsive or wellbeing or well-being):ti
#14	#7 or #8 or #9 or #10 or #11 or #12 or #13
#15	#6 and #14
#16	MeSH descriptor: [Depression, Postpartum] explode all trees
#17	MeSH descriptor: [Pregnant Women] explode all trees and with qualifier(s): [psychology - PX]
#18	(((pregnancy or pregnant or pre-nat* or prenat* or prepart* or prepart* or ante-nat* or antenat* or ante-part* or antepart* or peri-nat* or perinat* or peri-part* or peripart* or puerper* or post-nat* or postnat* or post-part* or postpart*) NEAR/5 (mental* or psych* or anxiety or anxious or depress* or mood* or affect* or distress* or stress or trauma* or posttrauma* or post-trauma* or adjustment disorder* or phobia* or phobic or obsessive compulsive or wellbeing or well-being))):ti,ab,kw
#19	((((parent or parents or mother* or maternal or father* or paternal) NEAR/5 (infan* or newborn* or neonat* or baby or babies)) and (mental* or psych* or anxiety or anxious or depress* or mood* or affect* or distress* or stress or trauma* or posttrauma* or post-trauma* or adjustment disorder* or phobia* or phobic or obsessive compulsive or wellbeing or well-being))):ti,ab,kw
#20	#15 or #16 or #17 or #18 or #19
#21	MeSH descriptor: [Implementation Science] explode all trees

For peer review only - http://bmjopen.bmj.com/site/about/guidelines.xhtml

#22	MeSH descriptor: [Health Plan Implementation] explode all trees
#23	MeSH descriptor: [Program Evaluation] explode all trees
#24	(implement* or impact*):ti,ab,kw OR (feasib* or acceptab*):ti,ab,kw OR ((barrier* or challenge* or obstacle* or hurdle* or obstruct* or drawback* or issue* or difficult* or promot* or support or encourag* or factor* or facilitat* or enabl* or opportunit* or engage* or assist*)):ti,ab,kw OR (((process or project* or system*) NEAR/5 evaluat*)):ti,ab,kw
#25	#21 or #22 or #23 or #24
#26	#20 and #25

For peer review only

For peer review only - http://bmjopen.bmj.com/site/about/guidelines.xhtml

Appendix 3: Table 2. Search Results

**Database:**	**Interface:**	**Coverage:**	**Date:**	**Hits:**
CINAHL	EBSCOHost	1982-present	04/08/2021	759
Cochrane Database of Systematic Reviews	Cochrane Library, Wiley	Issue 8 of 12, August 2021	04/08/2021	384
Embase	OvidSP	1974-present	04/08/2021	1081
Medline	OvidSP	1946-present	04/08/2021	977
PsycINFO	OvidSP	1806-present	04/08/2021	286
Scopus	Elsevier		04/08/2021	599
**Total:**				**4086**
Duplicates:				1992
Papers excluded:				66
**Final total:**				**2028**

**Papers excluded:** available if needed	
Fetal distress	46
Oxidative stress	9
Non-English	11
	66
Included - 27th May 2020	1671
Included - 4th August 2021	374

For peer review only - http://bmjopen.bmj.com/site/about/guidelines.xhtml

Appendix 4: Table 3. Inclusion and exclusion criteria

**Category**	**Criteria**
Population	Women in the perinatal period (conception to 12 months after birth) experiencing mental health problems, who may or may not have decided to seek help, accessed help, or engaged in PNMH care. PNMH care was defined as assessment, referrals, and/or treatment/intervention programmes) from health or social care services. Conception to 12 months after birth was chosen as the target population because this is the period that many perinatal mental health services cover [1–5].<br><br>Reviews were excluded if they were not conducted on the target population (e.g., men/partners, healthcare professionals), focused on substance misuse (which has unique challenges in terms of assessment and treatment), did not focus on the mental health of perinatal women.
Outcome	Barriers and facilitators (defined as any individual, healthcare professional, interpersonal, organisational, political, or societal factors that women believed impeded (barriers) or aided (facilitators) them) to seeking, accessing, or engaging in help for PNMH problems. Studies were included if they made descriptive statements about barriers and facilitators to women deciding to seek help, accessing help, and engaging in PNMH care. These descriptions had to be drawn from perinatal women's experiences.<br><br>Reviews were excluded if they did not examine any barriers/facilitators regarding seeking help, accessing help and engaging in PNMH care.
Design	Only systematic reviews were included. Studies that did not use a clearly reported PRISMA search strategy [6]were excluded.<br><br>Non-English publications were also excluded.

For peer review only - http://bmjopen.bmj.com/site/about/guidelines.xhtml

Appendix 5: Detailed methodology

Protocol and registration

The protocol for this review has been registered on PROSPERO (CRD42020193107) (see appendix for full protocol).

Eligibility criteria

Studies with the following characteristics were eligible for inclusion in the review: Population: Women in the perinatal period (conception to 12 months after birth) experiencing mental health problems, who may or may not have decided to seek help, accessed help, or engaged in PNMH care. PNMH care was defined as assessment, referrals, and/or treatment/intervention programmes) from health or social care services. Conception to 12 months after birth was chosen as the target population because this is the period that many perinatal mental health services cover [1–5].

Outcome: Barriers and facilitators (defined as any individual, healthcare professional, interpersonal, organisational, political, or societal factors that women believed impeded (barriers) or aided (facilitators) them) to seeking, accessing, or engaging in help for PNMH problems.

Studies were included if they made descriptive statements about barriers and facilitators to women deciding to seek help, accessing help, and engaging in PNMH care. These descriptions had to be drawn from perinatal women's experiences. Only systematic reviews were included. Studies that did not use a clearly reported PRISMA search strategy [6]were excluded. Reviews were also excluded if they were not conducted on the target population (e.g., men/partners, healthcare professionals), focused on substance misuse (which has unique challenges in terms of assessment and treatment), did not focus on the mental health of perinatal women, did not examine any barriers/facilitators regarding seeking help, accessing help and engaging in PNMH care, and were non-English publications.

Information sources

Searches were carried out in CINAHL (1982- present); Embase (1974 – present); Medline (1946-present); and PsycINFO (1806 – present), Cochrane, SCOPUS and TRIP (Turning Research into practice) Medical Database. The date of the last search was 28th May 2020. Forward and backward searches of included studies were carried out and completed by the 26th June 2020. Searches were updated on 4th August 2021 and forward and backward searches of new included studies were completed by 8th September 2021.

Search

Pre-planned searches were carried out using both MeSH terms (i.e. prenatal care/anxiety/ diagnosis) and search terms were combined with Boolean operators "OR" and "AND" (e.g. pregnancy OR perinatal OR postnat* AND anxiety OR depress* OR wellbeing AND intervention? OR counsel* OR support OR identifi* AND OR barrier? OR facilitate*).

Review selection

Search results were imported into Endnote and duplicates and papers not meeting initial inclusion criteria (foetal distress, oxidative stress, Non-English papers) were removed by NR. The remaining studies were imported into Eppi-Reviewer 4, where results were double screened by title and abstract by two people (RW & GC). An additional proportion (n = 166, ~7%) of titles and abstracts were triple screened by NU. Decisions to include or exclude were concordant between RW & GC in 94.2% of cases and between RW & NU in 99.39% of cases. Disagreements were discussed and resolved by NU, GC, and RW by applying the relevant inclusion criteria.

For peer review only - http://bmjopen.bmj.com/site/about/guidelines.xhtml

Once title and abstract screening was complete, full text screening was carried out by two people (RW & GC). An additional proportion (n = 9, ~10%) were triple screened by NU. Decisions to include or exclude were concordant between RW & GC in 91.4% of cases and between RW & NU in 100% of cases.

Data collection process and data items

Data extraction was carried out using Microsoft Excel by RW. Each paper was read in full, and relevant parts of the text input into the relevant part of the spreadsheet. Review methodology was copied onto one sheet and results onto another to aid analysis. Double coding of extracted data was carried out for a proportion of included reviews (n = 3, 10%) by GC. Data extraction matched in 85% of cases.

The data that were extracted was guided by the Cochrane Systematic Review for Intervention Data Collection form [7] and the Assessing the Methodological Quality of Systematic Reviews (AMSTAR 2;[8]) Data collected included the following: Review Characteristics (year of publication, author(s), design, aim, search strategy, inclusion/exclusion criteria, screening/study selection, data extraction, quality assessment, analysis methods); Sample Characteristics (Number of studies included, total number of participants, participant demographics); Assessment/Care/Treatment Characteristics (Healthcare setting, intervention description, screening description) and outcomes (barriers and facilitators).

Critical appraisal of reviews

Methodology sections of included systematic reviews were appraised using the AMSTAR 2. Critical domains in the appraisal of systematic reviews according to AMSTAR 2 include protocol registration, adequacy of literature search, justification of study exclusion, risk of bias, appropriateness of meta-analytic methods, consideration of risk of bias when interpreting results, and assessment of publication bias. If more than one critical domain is not met (critical flaw), a systematic review should be evaluated as having critically low confidence in the results of the review. One critical flaw means reviews should be evaluated as low confidence [8].

Given that all studies in this review were qualitative, the AMSTAR 2 items related to meta-analysis were not relevant and were thus removed. Further, given the debate in the literature regarding the appropriateness of conducting risk of bias assessments on qualitative research, we downgraded the items relating to risk of bias from being critical flaws, to flaws. Quality appraisal of all studies was carried out by NU and RW. Ratings were concordant in 90% of cases.

A decision was made to continue to include reviews where confidence in results was evaluated as low and critically low because these reviews focused more on marginalised women, such as refugees, migrants, women with a low income, and women living in LMIC, to ensure the experiences of these seldom-heard women were captured. To improve the validity of results, a qualitative sensitivity analysis was carried out to assess whether themes remained consistent across all reviews, regardless of their quality rating. The methods proposed by Harden[9] and Carroll et al[10] was followed and therefore sensitivity analysis was carried out in two ways: (1) synthesis contribution; (2) evidence of adequate description of themes.

To examine whether higher quality studies contributed more to the themes, a measure of "synthesis contribution" was calculated for each study (as done by Harden, 2007[9]) by dividing the number of barriers and facilitators identified by that study, by the total number of barriers and facilitators identified in the review overall. For example, the findings from Bina (2020) [11]contributed to 31 out of 62 themes, giving this review a synthesis contribution score of 50% (see appendix, Table 3). Each study's synthesis contribution scores was plotted against the number of quality criteria the study

For peer review only - http://bmjopen.bmj.com/site/about/guidelines.xhtml

met (see appendix, Figure 1). Statistical analysis (Pearson's correlation) was used to help interpret the plots. To examine whether removing lower quality reviews influenced the number of themes, themes that were only supported by lower quality reviews were identified (see appendix, Table 6).

To examine whether removing lower quality reviews influenced the description of themes, data were assessed for "thickness" or "thinness" (as done by Carroll et al., 2012[10]). Thin description refers to a set of statements (e.g. "O'Mahoney et al. found that women also felt that providers were downplaying the symptoms they were experiencing", Hansotte et al., 2017, [12]p.12), whereas thick description provides the context of experience and circumstances [13] (e.g. "Having symptoms dismissed or attributed to factors other than PPD by health care professionals led to women 'remaining silent.' Some women perceived that their difficulties would only be taken seriously when there were concerns about risk of harm to themselves or the infant. One woman said, 'I kept going to this doctor and he used to give me a pep talk and send me home…'", Hadfield & Wittkowski, 2017[14], p.732). It is argued that the extent to which a text provides a thick description shows evidence of the authenticity of the results [15].

Synthesis of results

Results were analysed by RW using a thematic synthesis [16] in NVivo and Microsoft Excel. First, line by line data coding of statements referring to facilitators or barriers to accessing PNMH care from the results section of each paper was carried out. Next, codes were revisited and assigned a descriptive theme based on their meaning and content. Themes were developed and revised as each review was re-read. Once all codes had been assigned into themes, the themes were mapped onto a multi-level framework adapted from Ferlie and Shortell's Levels of Change framework [17] and a previous systematic review on barriers and facilitators to implementation of PNMH care carried out by the review authors. The mapping of descriptive themes was developed deductively from the initial theoretical framework and then inductively revised as new themes emerged. The mapping of descriptive themes was discussed by all review authors before being finalised. A decision was taken to analyse all reviews together, regardless of the specific aims or individual inclusion criteria. This is because the majority of the reviews (n = 27) included studies carried out in a wide range of countries/settings. This, therefore, made it difficult to parse apart reviews based on sample characteristics, settings, or country of included studies.

For peer review only

For peer review only - http://bmjopen.bmj.com/site/about/guidelines.xhtml

Appendix 6: Table 4. Sensitivity analysis

Study	Number of themes	Unique synthesis contribution	Overall synthesis contribution (all themes)
Bina, 2020[11]	31	3.03030303	50
Brealey et al., 2010[18]	13	1.515151515	20.96774194
Button et al., 2017[19]	26	0	41.93548387
Dennis & Chung-Lee, 2006[20]	28	0	45.16129032
Evans et al., 2020[21]	8	0	12.90322581
Giscombe et al., 2020[22]	6	0	9.677419355
Forde,et al. 2020[46]	20	0	29.03
Hadfield & Wittkowski, 2017[14]	25	0	40.32258065
Hansotte et al., 2017[12]	19	1.515151515	30.64516129
Hewitt et al., 2009[23]	13	0	20.96774194
Holopainen & Hakulinen, 2019[24]	6	0	9.677419355
Jones et al., 2014[25]	10	0	16.12903226
Jones, 2019[26]	19	0	30.64516129
Kassam, 2019[27]	8	0	12.90322581
Lucas et al., 2019[28]	9	0	14.51612903
Megnin-Viggars et al., 2015[29]	26	0	41.93548387
Mollard et al., 2016[30]	5	1.515151515	8.064516129
Morrell et al., 2016[31]	16	0	25.80645161
Newman et al., 2019[32]	13	0	20.96774194
Nilaweera et al., 2014[33]	6	0	9.677419355
Praetorius et al., 2020[34]	3	0	4.838709677
Randall & Briscoe, 2018[35]	2	0	3.225806452
Sambrook-Smith et al., 2019[36]	19	0	30.64516129
Schmied et al., 2017[37]	27	1.515151515	43.5483871
Scope et al., 2017[38]	13	0	20.96774194
Slade et al., 2020[39]	15	0	24.19354839
Sorsa et al., 2021[40]	19	0	30.64516129

For peer review only - http://bmjopen.bmj.com/site/about/guidelines.xhtml

For peer review only

Staneva et al., 2015[41]	11	0	17.74193548
Tobin et al., 2018[42]	19	0	30.64516129
Viveiros & Darling, 2018[43]	16	1.515151515	25.80645161
Watson et al., 2019[44]	28	0	45.16129032
Wittkowski et al., 2014[45]	2	1.515151515	3.225806452

For peer review only

Appendix 7: *Figure 1.* Synthesis contribution vs quality appraisal criteria met

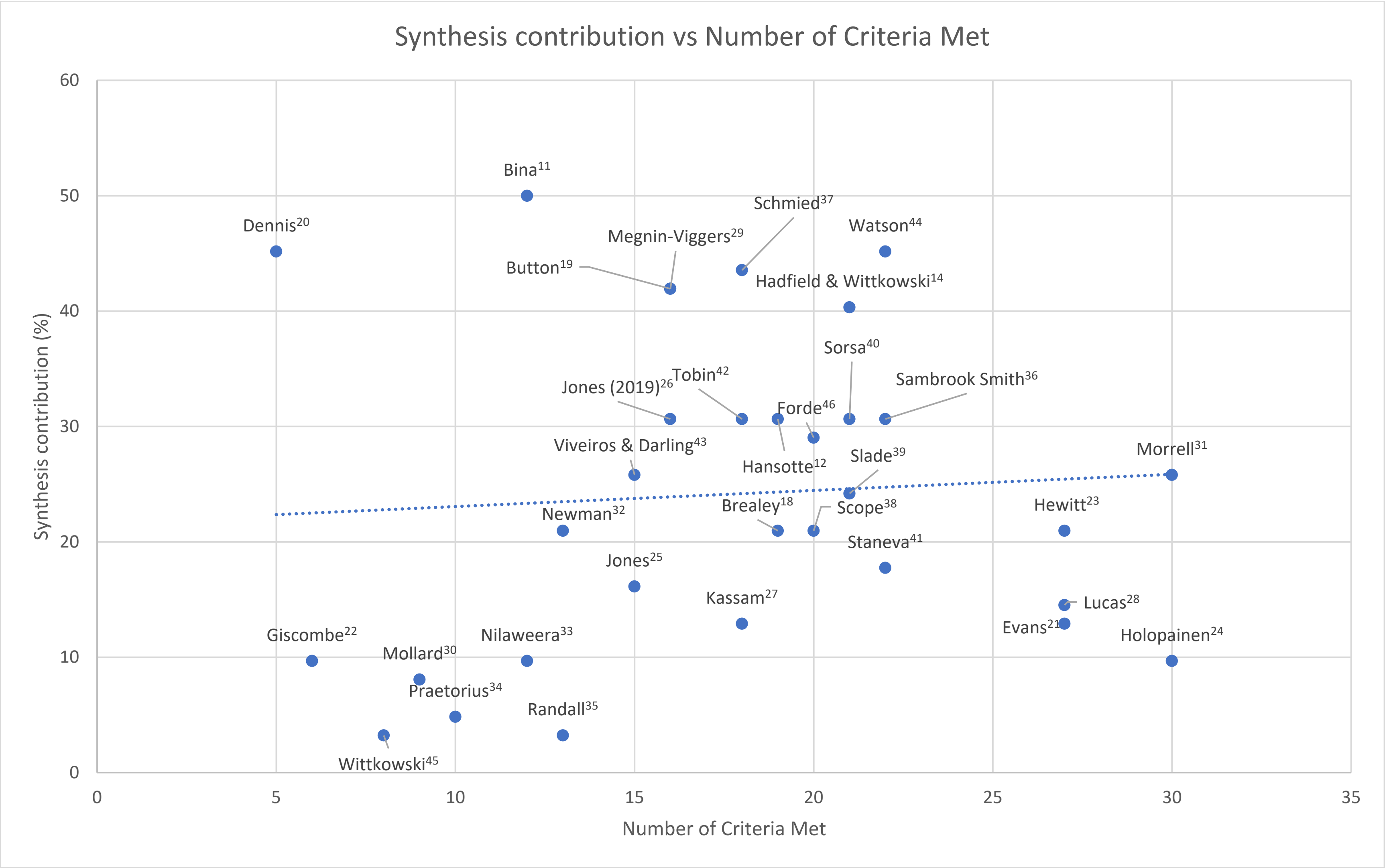

35

Appendix 8: Sensitivity analysis results

Synthesis contribution. There was no correlation between synthesis contribution and the number of criteria each review met (r = .142, p = .437; see appendix, Figure 1). Furthermore, only four themes (**cultural/spiritual** causes of mental illness, **age, previous diagnoses,** and **appropriateness of care)** were only identified by lower quality studies showing the majority of themes (58 out of 62; 93.55%) were supported by both higher quality and lower quality papers.

Richness of data. The removal of lower quality papers meant that the theme **language barriers** lost some of its richness. For example, it led to the removal of quotes expressing frustration from women whose first language was not English:

> '. . .you don't know where to go, what to do, who to trust, especially when you are coming by yourself. . . you believe that you speak English, but when you get here you realize that you don't.' [37]
>
> 'Sometimes when you have a baby, a woman comes from the hospital. Bengali girls don't come with the midwife, we don't understand what they say, we just sit there staring at their faces.' [19]

The removal of lower quality papers from the theme **fear of being seen as a bad mum** led to the loss of richness of data including the removal of a quote from women who have migrated from their country of birth:

> 'Back home, if someone has this problem, everyone gossips, you get this feeling that people are not dealing with you normally or as if you are abnormal almost. . .' [37]

Lastly, the removal of lower quality studies meant important information was removed from the **characteristics of service** theme, such as women feeling services prioritise physical needs (n = 2), lack information about screening guidelines (n = 2), and the logistics (e.g. location, time of appointments) of the care (n = 3)

For peer review only

Appendix 9: Table 5. Summary review characteristics

Characteristics	Range; Mean (M), Median (Mdn); Interquartile Range (IQR)
Year	2006-2022; M = 2017, Mdn = 2018; IQR = 2016-2019
No. studies included in each review	4-40; M = 16, Mdn = 13, IQR = 9-19
No. women included in each review	95-85,190; M = 5080; Mdn = 463; IQR = 226-1,715
Countries	N = 24

Appendix 10: Table 6. Characteristics of included reviews

	Review details			Participant details					
**Author & Year**	**N studies about women (Total N)**<br><br>**Years (Range)**	**Inclusion criteria**	**Country of studies**	**N**<br><br>**M (SD)**	**Age**	**Perinatal period**	**Ethnicity**	**Mental illness**	**Socioeconomic status**
Bina (2020)[11]	31 (35)<br><br>1993-2018	Service use for postpartum depression or "distress" from women (and HCP) perspectives	11 countries (4 LMIC)	7219<br><br>232.9 (414.7)	NR	2 weeks - up to 3 years postnatal	6 studies specified recruitment of migrant women or women of colour[(EA; H; Ar)]	Depressive symptoms, emotional difficulties or current/past diagnosis of mood disorder	2 studies recruited women with low income.<br>1 study recruited women using Medicaid.
Brealey et al. (2010)[18]	13 (16)<br><br>1997-2007	Acceptability to women (and HCP) of screening to identify women with increased risk of	5 countries (all HIC)	1715<br><br>131.9 (253.06)	24-34<br>M (n = 8) = 29.63	First antenatal appointment – 12 months after birth	2 studies recruited women of colour [(B; EA; Ar)]	Women at risk of postnatal depression	One study reported marriage (29/30 women were married)

For peer review only - http://bmjopen.bmj.com/site/about/guidelines.xhtml

		postnatal depression							
Button et al. (2017)[19]	24 (24)<br><br>1993-2016	Help seeking for postnatal depression	9 studies carried out in UK. No other countries reported.	NR	NR	Postnatal	9 studies recruited women of colour. 3 studies had mixed samples.	Postnatal depression	NR
Dennis and Chung-Lee (2006)[20]	40 (40)<br><br>NR	Maternal help-seeking barriers and facilitators and treatment preferences for postnatal depression	3 were explicitly stated (all HIC)	NR	NR	Up to 1 year after birth	Three studies recruited women of colour[SA; EA;B;Ar]	Postnatal depression	NR
Evans et al. (2020)[21]	14 (14)<br><br>2009-2015	Acceptability of non-pharmacological interventions for antenatal anxiety	6 countries (all HIC)	235<br><br>16.8 (9.6)	NR	Between 6-40 weeks gestation	NR	8 studies recruited women with a history of mood concerns/ anxiety or depression	2 studies recruited women with “social risk factors”
Forde, Peters & Wittkowski (2020)[46]	13 (15)<br><br>2003-2018	Published empirical studies exploring women’s or family members’ experiences of PP and/or recovery using	4 countries (all HIC)	103<br>7.92 (2.96)	Range: 23-62	All postnatal, ranging from 4 months to 26 years after onset of postnatal psychosis	One woman was an Orthodox Jewish woman	All women had recovered from, or were currently experiencing postnatal psychosis	NR

For peer review only - http://bmjopen.bmj.com/site/about/guidelines.xhtml

		a qualitative methodology							
Giscombe, Hui & Stickley (2020)[22]	8 (8)<br><br>2008-2017	Refugee or asylum-seeking women, with mental health complications during perinatal period	3 countries (all HIC)	NR	NR	NR	Syrian refugees, Eritrean refugees	6 studies recruited women with depression; 3 with PTSD (1 study recruited both depression & PTSD)	All women were refugees or asylum seekers
Hadfield and Wittkowski (2017)[14]	17 (17)<br><br>2004-2015	Mothers with postnatal depression and their experiences about help seeking for psychosocial support	4 countries (all HIC)	532<br><br>31.3 (25.97)	Range 18-45<br><br>M (n = 2) = 30.2	Postnatal	3 studies recruited women who weren't born in the UK[B;EA]	Postnatal depression	NR
Hansotte et al. (2017)[12]	18 (18)<br><br>2004-2015	Screening for postnatal depression and barriers to accessing treatment in low-income women in western countries.	2 countries (all HIC)	85190<br><br>5011 (11613)	M (n = 11) = 25.11	Postnatal	All studies recruited a diverse sample of migrant women or women of colour [B; L; W; As; NI]	Self-report depression symptoms or depression diagnosis	All women were low income living in high income western country.
Hewitt et al. (2009)[23]	13 (16)<br><br>1997-2007	Acceptability to women (and HCP) about methods to	5 countries (all HIC)	1715<br><br>131.9 (253.06)	M (n = 8) = 29.63	Postnatal: 1-12 months	4 studies recruited women of	Perinatal depression	2 studies looked at marriage. The majority of women

For peer review only - http://bmjopen.bmj.com/site/about/guidelines.xhtml

		identify postnatal depression				Antenatal: all trimesters	colour (Ar; EA; B; NI; NS)		were married (87-97%)
Holopainen and Hakulinen (2019)[24]	13 (15)<br><br>2005-2015	Mothers (and fathers) experiences of postnatal depression symptoms	7 countries (all HIC)	199<br><br>15.31 (8.21)	Ages ranged from 16-45	1-12 months after birth	5 studies recruited women of colour (B, L, H, SA, EA)	Most studies looked at symptoms of depression, 2 looked at diagnoses	1 study recruited low-income women, one recruited adolescent mothers. Most women were married (n = 3; 59-66%). Most women had more than 9 years of education (n = 2; 87-100%)
Jones et al. (2014)[25]	5 (5)<br><br>1995-2012	Women's experiences of peer support for any degree of perinatal mental illness	3 countries (all HIC)	95<br><br>19 (18.93)	NR	6 weeks - 2 years after birth	NR	Postnatal depression diagnosis or symptoms	NR
Jones (2019)[26]	19 (19)<br><br>2008-2017	Help seeking in women with perinatal depression	All USA	6089<br><br>358.90 (1226.22)	NR	Pregnancy – 6 months after birth	6 studies recruited women of colour (B, L, SA, EA, NS)	All had perinatal depression identified through screening measures, or self-reported.	All women had pregnancy complications. 3 studies recruited women on a low income.
Kassam (2009)[27]	11 separate populations<br><br>1999-2013	Voices of immigrant and refugee women with postnatal depression in terms of social support as a	3 countries stated (HIC & UMIC countries)	191<br><br>23.88 (10.89)	All aged over 17	Screened high on a postnatal depression scale within 2 weeks - 5 years after birth	All studies recruited migrant women or women of colour (NS; As; Ar; SA; H)	Most had postnatal depressive symptoms, identified through screening. One study reported	One study looked at risk profile of women (e.g. low income, experienced violence, experienced war, previous mental health difficulty).

For peer review only - http://bmjopen.bmj.com/site/about/guidelines.xhtml

		coping resource						depression diagnosis	All women in 2 studies were married or in a relationship. One study recruited low-income women.
Lucas et al. (2019)[28]	19 (19)<br><br>1999-2017	Young women's perception of their mental health and wellbeing	3 countries (all HIC)	356<br><br>18.74 (10.02)	Ages ranged from 13-25.<br><br>M (n = 2) = 18.75	11 studies recruited were parents (3 months - 2 years postnatal). 2 studies recruited pregnant women. Remaining studies recruited both pregnant and postnatal women	Majority of studies (15) recruited ethnically diverse (L, B, H, SA, M, As) samples. 4 studies did not report ethnicity	Depressive symptoms, depression diagnosis, other diagnoses (bipolar, panic disorder, mood disorder).	All women were young (maximum age 25)
Megnin-Viggars et al. (2015)[29]	39 (39)<br><br>2001-2013	Women with, or at risk of developing a postnatal mental health problem and their views on factors that improve or	Only reported for 3 studies (all UK)	955<br><br>24.49 (43.77)	1 study recruited teenage mother. No other ages reported.	Antenatal and postnatal	5 studies recruited ethnically diverse samples (B, NS, SA)	Most studies recruited women with depression (n = 14) or women at risk (n = 18) of perinatal mental health problems.	1 study recruited teenage mothers

For peer review only - http://bmjopen.bmj.com/site/about/guidelines.xhtml

		diminish access to perinatal mental health services							
Mollard et al. (2016)[30]	11 (11)<br><br>1995-2014	Women living in rural areas of the USA with PPD. Looking at screening uptake, intervention acceptability, lived experience, help-seeking.	All USA	1610<br><br>146.36 (159.57)	NR	Postnatal	5 studies recruited ethnically diverse samples (NI; B; NS; H)	Postnatal depression symptoms, most used EPDS[1] (n = 6) screen	All women lived in rural location, 3 studies recruited low income women
Morrell et al. (2016)[31]	38 individual samples of women in the qualitative review<br><br>1987-2013	Pregnant and postnatal women, views on preventative or targeted services for PND	8 countries (1 LMIC – India)	1673 (34 studies reported sample size)<br><br>49.21 (98.49)	Ages ranged from 15-54<br><br>M (n = 12) = 28.62	Pregnancy and postnatal	10 recruited ethnically diverse samples (SA; EA, B; H; NI; L; M; NS)	Depression - both symptoms and diagnoses	25 studies reported sociodemographic characteristics. 16 studies reported marital status, in all but 1 study the majority of women were married/cohabiting/in a relationship. 8 studies reported education status: most had completed high school or above. 4 studies recruited low-income women or those living in an

For peer review only - http://bmjopen.bmj.com/site/about/guidelines.xhtml

									impoverished/deprived area
Newman et al. (2019)[32]	4 (4)<br><br>2008-2016	Women with depression during the postnatal period sharing views on help-seeking	3 countries (all HIC)	118<br><br>29.5 (9)	M (n = 3) = 31.97	Postnatal	NR	Depressive symptoms, measured by EPDS[1]	NR
Nilaweera et al. (2014)[33]	9 (15)	Women who have migrated from South Asian countries to live in high-income countries, barriers and enablers to health care access	4 countries (all HIC)	20,788<br><br>2309.78 (3926.13)	NR	2 weeks to 5 years postnatal	All studies recruited women born in South Asia	Most (n = 5) used EPDS[1] to assess postnatal depression symptoms	NR
Praetorius, Maxwell & Alam (2020)[34]	8 (8)<br><br>1999-2016	Mothers with depression and suicidality	5 countries (3 HIC, 1 UMIC, 1LMIC)	199<br><br>24.88 (12.52)	Ages range from 17-44	Pregnancy and postnatal	All studies recruited diverse samples (B, L, M, SA, EA, Ar, W)	All women had depression and suicidality	NR
Randall and Briscoe (2018)[35]	4 (4)<br><br>2005-2014	Women's decision-making process around antidepressant	2 countries (all HIC)	368<br><br>92 (37.09)	Ages ranged from 25-34	Pregnancy	3 studies reported ethnicity. The majority of women	Depression – 1 study used the CES-D[2] to identify depressive symptoms	3 studies report education, the majority (82.5-100%) were educated to above high school level. 3 studies

For peer review only - http://bmjopen.bmj.com/site/about/guidelines.xhtml

		use during pregnancy			M (n = 2) = 31		were white (77.5-95%)		reported relationship status, the majority (80-98%) were married/living with partner
Slade, Molyneux & Watt (2021)[39]	13 (13 – qualitative papers only)<br><br>2007-2019	Help seeking for birth trauma/ postnatal PTSD	7 countries (1 UMIC; 6 HIC)	394<br><br>30.31 (32.85)	Ages range from 2-45<br><br>M (n=4) = 32	Up to 18 months after birth	8 studies reported ethnicity. One study reported recruiting women of colour[B, H]	All PTSD after birth	One study recruited low-income women. 2 studies reported marital status, over 58% were married. 2 studies reported higher education, at least 50% of women had completed this.
Sambrook-Smith et al. (2019)[36]	24 (35)<br><br>2007 - 2018	Barriers to accessing perinatal mental health care from the perspective of women (families & HCP)	All UK	384<br><br>16 (8.80)	NR	Postnatal	9 recruited women of colour [B; SA; EA]	Most looked at depressive symptoms (n = 12). Studies also recruited women with antenatal anxiety (n = 1), postnatal psychosis (n = 5), PTSD (n = 1) and substance misuse (n =1)	NR
Sorsa, Kylma and Bondas (2021)[40]	14 (14)<br><br>2002-2018	Helpseeking in women with perinatal distress	5 countries (all HIC)	345<br><br>24.65 (11.99)	Ages ranged from 18-55	Antenatal and postnatal	NR	Postnatal depression (n -= 8); prenatal depression (n = 2); Perinatal	NR

For peer review only - http://bmjopen.bmj.com/site/about/guidelines.xhtml

					M (n = 7) = 30.21			mental health needs (n = 2); Postpartum mood disorder (n = 1), Bipolar disorder (n = 1)	
Schmied et al. (2017)[37]	12 individual samples<br><br>1999-2015	Migrant women living in high income countries	4 countries (all HIC)	250<br><br>20.83 (12.52)	M (n = 5) = 29.4	Postnatal	All studies recruited migrant women or women of colour (SA; EA; H; B; Ar; L)	Depressive symptoms or formal diagnosis	1 study recruited low income women
Scope et al. (2017)[38]	22 individual samples<br><br>1987 - 2014	Service user views on uptake, acceptability of preventative interventions for PND	7 countries (all HIC)	982 (reported by author)	13-45 years	Antenatal and postnatal	NR	NR	NR
Staneva et al. (2015)[41]	8 (8)<br><br>2006-2012	Womens experience of antenatal mental health difficulties	5 countries (1 LMIC - Cambodia)	1094<br><br>14 (6.26)	Ages ranged from 16-47	Antenatal	Most studies (n = 6) recruited ethnically diverse samples (B; M)	Self-report distress, depression (n = 5); diagnoses depression/anxiety (n = 2); FOC = 1	50-100% of women were in a relationship
Tobin et al. (2018)[42]	8 (individual samples)	Refugee or immigrant women's experiences of	3 countries (all HIC)	139<br><br>17.38 (7.98)	Age ranges between	Postnatal	All studies recruited migrant women	Postnatal depression	6 studies reported relationship status 50-85% of women were

For peer review only - http://bmjopen.bmj.com/site/about/guidelines.xhtml

	2004-2013	postpartum depression			17-54 years		and women of colour(L; H; SA;EA;B)		married/in a relationship
Viveiros and Darling (2019)[43]	7 (26)<br><br>2009-2018	To explore women (and midwives) perceptions on factors that impede access to perinatal mental health care in high resource settings	2 countries (both HIC)	301<br><br>43 (66.30)	1 study reported age range from 23-40	Antenatal and postnatal	2 studies recruited 'BAME' women, one recruited all Black women	PTSD symptoms (n = 1); mental health problems (n = 2); mental illness diagnosis (n = 1)	NR
Watson et al. (2019)[44]	15 (15)<br><br>1994-2015	Ethnic minority women's experience of perinatal mental ill health, help-seeking and perinatal mental health services in Europe	All UK	4970<br><br>331.33 (1173.09)	NR	Antenatal and postnatal	All studies recruited women of colour (SA; NS; N; EA; M)	Distress, depression, mood and mental health, well-being	NR
Wittkowski et al. (2014)[45]	12 (12)<br><br>1983 - 2009	Culturally determined risk factors of PND in Sub-Saharan Africa	3 countries – all Sub-Saharan Africa	3642<br><br>404.67 (343.16)	NR	Postnatal	NR	All used self-report measures of depression	NR

For peer review only - http://bmjopen.bmj.com/site/about/guidelines.xhtml

Note. Where studies recruited populations that were not perinatal women, the information from these studies are not included in this table. HCP = Healthcare professional; LMIC = Lower-Middle Income Country; HIC = Higher Income Country; PTSD = Post-traumatic stress disorder; FOC = Fear of Childbirth. 1 = Edinburgh Postnatal Depression Scale (Cox et al., 1987); 2 = Center for Epidemiological Studies-Depression (Radloff, 1977).

For ethnicities: As = Asian (where the area of Asia was not specified in the study); EA = East Asian (e.g. Vietnamese; Chinese; Thai); SA = South Asian (e.g. Indian; Bangladeshi; Pakistani; Sri-Lankan); Ar = Arab countries (e.g. Jordanian, Egyptian); Ar = Arabic; B = Black; H = Hispanic; L = Latina; M = mixed or multiple ethnic groups; NI = Native/Indigenous; NS = Not specified; W = White.

For peer review only - http://bmjopen.bmj.com/site/about/guidelines.xhtml

Appendix 11: Table 7. Quality appraisal

**Author, year**	**Q1. PICO**	**Q2. Protocol***	**Q3. Study design**	**Q4. Literature search***	**Q5. Study selection**	**Q6. Data extraction**	**Q7. Excluded studies***	**Q8. Included studies**	**Q9. ROB**	**Q.10 Funding**	**Q13. ROB interpretation**	**Q14. Heterogeneity**	**Q16. conflict of interest***	**Rating**
**Bina, 2020[11]**	Yes	Yes	No	Partial yes	Yes	No	Partial yes	Yes	No	No	No	Yes	No	LOW
**Brealey et al., 2010[18]**	Yes	Yes	Yes	Yes	Yes	Yes	No	Yes	Partial yes	No	Yes	Yes	No	CRITICALLY LOW
**Button et al., 2017[19]**	Yes	No	Yes	Yes	No	No	Partial yes	Partial yes	Yes	No	Yes	Yes	Yes	LOW
**Dennis & Chung-Lee, 2006[20]**	Yes	No	Yes	Yes	No	Yes	No	Partial yes	No	No	No	No	No	CRITICALLY LOW
**Evans et al., 2020[21]**	Yes	Yes	Yes	Yes	Yes	Yes	Partial yes	Yes	Yes	No	Yes	Yes	Yes	HIGH
**Forde et al., 2020[46]**	Yes	Yes	Yes	Partial yes	Yes	No	Partial yes	Ys	Yes	No	Yes	Yes	Yes	MODERATE
**Giscombe et al., 2020[22]**	Yes	No	Yes	Partial yes	No	No	Partial yes	Yes	Yes	No	No	Yes	No	CRITICALLY LOW
**Hadfield & Wittkowski, 2017[14]**	Yes	Yes	Yes	Yes	No	No	Partial yes	Yes	Yes	No	Yes	Yes	Yes	MODERATE
**Hansotte et al., 2017[12]**	Yes	No	Yes	Yes	Yes	Yes	Partial yes	Yes	No	No	No	Yes	Yes	LOW
**Hewitt et al., 2009[23]**	Yes	Yes	Yes	Yes	Yes	Yes	Partial yes	Yes	Yes	No	Yes	Yes	Yes	HIGH
**Holopainen & Hakulinen, 2019[24]**	Yes	Yes	Yes	Yes	Yes	Yes	Yes	Yes	Yes	No	Yes	Yes	Yes	HIGH

**Jones et al., 2014[25]**	Yes	No	Yes	Yes	No	Yes	No	Partial yes	Yes	No	Yes	Yes	Yes	CRITICALLY LOW
**Jones, 2019[26]**	Yes	No	Yes	Partial yes	Yes	No	Partial yes	Yes	No	No	Yes	Yes	Yes	LOW
**Kassam, 2019[27]**	Yes	Yes	Yes	Partial yes	No	No	Partial yes	Yes	Yes	No	Yes	Yes	Yes	MODERATE
**Lucas et al., 2019[28]**	Yes	Yes	Yes	Yes	Yes	Yes	Partial yes	Yes	Yes	No	Yes	Yes	Yes	HIGH
**Megnin-Viggars et al., 2015[29]**	Yes	Yes	Yes	Partial yes	No	Yes	Partial yes	Yes	Yes	No	Yes	Yes	No	LOW
**Mollard et al., 2016[30]**	Yes	No	Yes	Partial yes	No	No	Partial yes	Partial yes	No	No	No	Yes	Yes	LOW
**Morell et al. 2016[31]**	Yes	Yes	Yes	Yes	Yes	Yes	Yes	Yes	Ye	No	Yes	Yes	Yes	HIGH
**Newman et al., 2019[32]**	Yes	No	Yes	Yes	No	No	Partial yes	Yes	Yes	No	No	No	Yes	LOW
**Nilaweera et al., 2014[33]**	Yes	No	No	Partial yes	No	No	Partial yes	Yes	Yes	No	Yes	Yes	Yes	LOW
**Praetorius et al., 2020[34]**	No	No	Yes	Yes	No	Yes	Partial yes	Partial yes	No	No	No	No	Yes	LOW
**Randall & Briscoe, 2018[35]**	Yes	No	No	Partial yes	Yes	No	Partial yes	Partial yes	Yes	No	No	Yes	Yes	LOW
**Sambrook-Smith et al., 2019[36]**	Yes	Yes	Yes	Partial yes	Yes	No	Partial yes	Yes	Yes	No	Yes	Yes	Yes	MODERATE
**Schmied et al., 2017[37]**	Yes	No	Yes	Partial yes	Yes	No	Partial yes	Yes	Yes	No	Yes	Yes	Yes	LOW
**Scope et al., 2017[38]**	Yes	Yes	Yes	Partial yes	Yes	Yes	Partial yes	Yes	Yes	No	Yes	Yes	No	LOW

For peer review only - http://bmjopen.bmj.com/site/about/guidelines.xhtml

**Staneva et al., 2015[41]**	Yes	Yes	Yes	Partial yes	Yes	No	Partial yes	Yes	Yes	No	Yes	Yes	Yes	MODERATE
**Slade et al., 2020[39]**	Yes	Yes	Yes	Yes	No	No	Partial yes	Yes	Yes	No	Yes	Yes	Yes	MODERATE
**Sorsa et al., 2021[40]**	Yes	No	Yes	Partial yes	Partial yes	Yes	Partial yes	Yes	Yes	No	Yes	Yes	Yes	LOW
**Tobin et al., 2018[42]**	Yes	Yes	Yes	Yes	No	No	No	Yes	Yes	No	No	Yes	Yes	MODERATE
**Viveiros & Darling, 2018[43]**	Yes	No	No	Yes	Yes	No	Partial yes	Yes	No	No	No	Yes	Yes	LOW
**Watson et al., 2019[44]**	Yes	Yes	Yes	Partial yes	Yes	No	Partial yes	Yes	Yes	No	Yes	Yes	Yes	MODERATE
**Wittkowski et al., 2014[45]**	Yes	No	Yes	Partial yes	No	No	Partial yes	Yes	Yes	No	Yes	Yes	No	CRITICALLY LOW

* = Critical domain

1. Did the research questions and inclusion criteria for the review include the components of PICO? *2.* Did the report of the review contain an explicit statement that the review methods were established prior to the conduct of the review and did the report justify any significant deviations from the protocol?*3.* Did the review authors explain their selection of the study designs for inclusion in the review?4. Did the review authors use a comprehensive literature search strategy? 5. Did the review authors perform study selection in duplicate? 6. Did the review authors perform data extraction in duplicate? 7. Did the review authors provide a list of excluded studies and justify the exclusions? 8. Did the review authors describe the included studies in adequate detail? 9. Did the review authors use a satisfactory technique for assessing the risk of bias (RoB) in individual studies that were included in the review? 10. Did the review authors report on the sources of funding for the studies included in the review? 11. If meta-analysis was performed, did the review authors use appropriate methods for statistical combination of results? *(not applicable)* 12. If meta-analysis was performed, did the review authors assess the potential impact of RoB in individual studies on the results of the meta-analysis or other evidence synthesis? *(not applicable)* 13. Did the review authors account for RoB in primary studies when interpreting/discussing the results of the review? 14. Did the review authors provide a satisfactory explanation for, and discussion of, any heterogeneity observed in the results of the review? 15. If they performed quantitative synthesis did the review authors carry out an adequate investigation of publication bias (small study bias) and discuss its likely impact on the results of the review? *(not applicable)* 16. Did the review authors report any potential sources of conflict of interest, including any funding they received for conducting the review?

For peer review only - http://bmjopen.bmj.com/site/about/guidelines.xhtml

Appendix 12: Table 8. Themes

Theme	Studies reflecting this theme
**1. Women**	
**1.1 Beliefs about health services**	
**1.1.1 Medication only**	Bina, 2020; Button et al., 2017; Jones, 2019; Megnin-Viggars et al., 2015; Nilaweera et al., 2014; Sorsa et al., 2021; Tobin et al., 2018
**1.1.2 Stretched**	Hadfield & Wittkowski, 2017
**1.2 Beliefs about healthcare professionals**	
**1.2.1 What is their role?**	Brealey et al., 2010; Button et al., 2017; Dennis & Chung-Lee, 2006; Hadfield & Wittkowski, 2017; Hewitt et al., 2009; Megnin-Viggars et al., 2015; Mollard et al., 2016; Morrell et al., 2016; Nilaweera et al., 2014; Schmied et al., 2017; Scope et al., 2017; Smith et al., 2019
**1.2.2 They won't be interested**	Bina, 2020; Hadfield & Wittkowski, 2017
**1.3 Beliefs about mental illness**	
**1.3.1 Not knowing what it is**	Bina, 2020; Button et al., 2017; Dennis & Chung-Lee, 2006; Hadfield & Wittkowski, 2017; Hansotte et al., 2017; Jones, 2019; Lucas et al., 2019; Megnin-Viggars et al., 2015; Morrell et al., 2016; Newman et al., 2019; Schmied et al., 2017; Scope et al., 2017; Smith et al., 2019; Staneva et al., 2015; Tobin et al., 2018; Watson et al., 2019
**1.3.1.1. Not having the language to describe perinatal mental illness**	Brealey et al., 2010; Staneva et al., 2015; Tobin et al., 2018; Watson et al., 2019
**1.3.2 Causes**	
**1.3.2.1 Cultural/spiritual**	Schmied et al., 2017; Wittkowski et al., 2014
**1.3.2.2 External factors**	Bina, 2020; Button et al., 2017; Dennis & Chung-Lee, 2006; Lucas et al., 2019; Schmied et al., 2017; Staneva et al., 2015; Tobin et al., 2018; Watson et al., 2019
**1.3.2.3 Physical factors**	Bina, 2020; Button et al., 2017; Dennis & Chung-Lee, 2006; Forde et al., 2020; Jones et al., 2014; Newman et al., 2019; Schmied et al., 2017; Smith et al., 2019; Staneva et al., 2015; Watson et al., 2019
**1.3.2.4 A normal response to motherhood?**	Dennis & Chung-Lee, 2006; Giscombe et al., 2020; Jones et al., 2014; Schmied et al., 2017; Slade et al., 2020; Smith et al., 2019; Sorsa et al., 2021
**1.3.3 How to deal with symptoms**	
**1.3.3.1 Ignore them**	Bina, 2020; Hadfield & Wittkowski, 2017; Jones, 2019; Newman et al., 2019; Schmied et al., 2017; Slade et al., 2020
**1.3.3.2 Seek spiritual guidance**	Hansotte et al., 2017; Kassam, 2019; Schmied et al., 2017; Watson et al., 2019
**1.4 Deciding to seek help**	

For peer review only - http://bmjopen.bmj.com/site/about/guidelines.xhtml

**1.4.1 Recognising something is wrong**	Bina, 2020; Button et al., 2017; Forde et al., 2020; Hadfield & Wittkowski, 2017; Hansotte et al., 2017; Slade et al., 2020; Sorsa et al., 2021; Staneva et al., 2015; Viveiros & Darling, 2018
**1.4.2 Where do I go to seek help?**	Bina, 2020; Dennis & Chung-Lee, 2006; Hansotte et al., 2017; Jones, 2019; Megnin-Viggars et al., 2015; Schmied et al., 2017; Smith et al., 2019; Sorsa et al., 2021; Tobin et al., 2018
**1.5 Fear of judgement**	
**1.5.1 Fear of being seen as a bad mum**	Bina, 2020; Brealey et al., 2010; Button et al., 2017; Forde et al., 2020; Jones et al., 2014; Lucas et al., 2019; Schmied et al., 2017; Slade et al., 2020; Sorsa et al., 2021; Viveiros & Darling, 2018; Watson et al., 2019
**1.5.2 Social services/removal of child**	Bina, 2020; Brealey et al., 2010; Button et al., 2017; Dennis & Chung-Lee, 2006; Evans et al., 2020; Forde et al., 2020; Hadfield & Wittkowski, 2017; Hewitt et al., 2009; Jones, 2019; Megnin-Viggars et al., 2015; Newman et al., 2019; Tobin et al., 2018; Watson et al., 2019
**1.5.3 Symptom minimisation**	Bina, 2020; Dennis & Chung-Lee, 2006; Forde et al., 2020; Hewitt et al., 2009; Holopainen & Hakulinen, 2019; Jones et al., 2014; Kassam, 2019; Lucas et al., 2019; Megnin-Viggars et al., 2015; Schmied et al., 2017; Staneva et al., 2015; Watson et al., 2019
**1.6 Logistics**	
**1.6.1 Childcare**	Bina, 2020; Button et al., 2017; Dennis & Chung-Lee, 2006; Hansotte et al., 2017; Morrell et al., 2016; Newman et al., 2019; Scope et al., 2017; Smith et al., 2019; Tobin et al., 2018; Watson et al., 2019
**1.6.2 Timing**	Bina, 2020; Dennis & Chung-Lee, 2006; Newman et al., 2019; Scope et al., 2017; Watson et al., 2019
**1.6.3 Location/travel**	Bina, 2020; Hansotte et al., 2017; Jones, 2019; Mollard et al., 2016; Morrell et al., 2016; Newman et al., 2019; Schmied et al., 2017; Sorsa et al., 2021; Tobin et al., 2018; Watson et al., 2019
**1.7 Social and family life**	
**1.7.1 Social isolation/support**	Bina, 2020; Giscombe et al., 2020; Holopainen & Hakulinen, 2019; Jones, 2019; Jones et al., 2014; Kassam, 2019; Lucas et al., 2019; Tobin et al., 2018; Viveiros & Darling, 2018
**1.7.1. 1 Exacerbated by mental illness**	Holopainen & Hakulinen, 2019; Jones et al., 2014; Watson et al., 2019
**1.7.2 Family and friends**	Bina, 2020; Button et al., 2017; Dennis & Chung-Lee, 2006; Forde et al., 2020; Hadfield & Wittkowski, 2017; Hansotte et al., 2017; Holopainen & Hakulinen, 2019; Jones, 2019; Lucas et al., 2019; Nilaweera et al., 2014; Schmied et al., 2017; Smith et al., 2019; Sorsa et al., 2021; Viveiros & Darling, 2018; Watson et al., 2019
**1.8 Sociodemographic factors**	
**1.8.1 Ethnicity**	Bina, 2020; Dennis & Chung-Lee, 2006; Hansotte et al., 2017; Watson et al., 2019
**1.8.2 Age**	Bina, 2020; Hansotte et al., 2017
**1.8.3 Previous experiences**	Button et al., 2017; Evans et al., 2020; Hadfield & Wittkowski, 2017; Hansotte et al., 2017; Jones, 2019; Sorsa et al., 2021; Watson et al., 2019
**1.8.4 Previous Diagnoses/symptoms**	Bina, 2020; Viveiros & Darling, 2018

For peer review only - http://bmjopen.bmj.com/site/about/guidelines.xhtml

**2. HCP**	
**2.1 HCP being dismissive or normalising symptoms**	Button et al., 2017; Dennis & Chung-Lee, 2006; Forde et al., 2020; Hadfield & Wittkowski, 2017; Hansotte et al., 2017; Megnin-Viggars et al., 2015; Newman et al., 2019; Sorsa et al., 2021; Watson et al., 2019
**2.2 HCP not recognising help seeking**	Bina, 2020; Button et al., 2017; Megnin-Viggars et al., 2015; Tobin et al., 2018; Watson et al., 2019
**2.3 HCP appearing too busy**	Bina, 2020; Button et al., 2017; Dennis & Chung-Lee, 2006; Hewitt et al., 2009; Megnin-Viggars et al., 2015; Slade et al., 2020; Viveiros & Darling, 2018; Watson et al., 2019
**2.3 Women's perceptions of HCPs knowledge**	
**2.3.1 Perception of HCP knowledge about PNMI**	Dennis & Chung-Lee, 2006; Forde et al., 2020; Hansotte et al., 2017; Jones, 2019; Megnin-Viggars et al., 2015; Morrell et al., 2016
**2.3.2 Perception of HCP knowledge about services/referral pathways**	Dennis & Chung-Lee, 2006; Smith et al., 2019; Viveiros & Darling, 2018
**2.4 The way the HCP delivers the care**	Button et al., 2017; Forde et al., 2020; Kassam, 2019; Megnin-Viggars et al., 2015; Nilaweera et al., 2014; Schmied et al., 2017; Slade et al., 2020; Smith et al., 2019; Sorsa et al., 2021
**2.5 HCP characteristics**	Brealey et al., 2010; Button et al., 2017; Dennis & Chung-Lee, 2006; Forde et al., 2020; Hadfield & Wittkowski, 2017; Hewitt et al., 2009; Megnin-Viggars et al., 2015; Morrell et al., 2016; Newman et al., 2019; Schmied et al., 2017; Slade et al., 2020; Staneva et al., 2015; Viveiros & Darling, 2018
**3. Interpersonal**	
**3.1 Relationship and rapport**	Bina, 2020; Brealey et al., 2010; Dennis & Chung-Lee, 2006; Hadfield & Wittkowski, 2017; Hewitt et al., 2009; Megnin-Viggars et al., 2015; Morrell et al., 2016; Scope et al., 2017; Slade et al., 2020; Tobin et al., 2018
**3.2 Language barriers**	Button et al., 2017; Dennis & Chung-Lee, 2006; Hansotte et al., 2017; Megnin-Viggars et al., 2015; Schmied et al., 2017; Smith et al., 2019
**3.3 Shared decision making**	Bina, 2020; Hadfield & Wittkowski, 2017; Megnin-Viggars et al., 2015; Morrell et al., 2016; Randall & Briscoe, 2018; Scope et al., 2017
**3.4 Communication**	Brealey et al., 2010; Forde et al., 2020; Hadfield & Wittkowski, 2017; Hewitt et al., 2009
**3.5 Information provision**	Bina, 2020; Button et al., 2017; Megnin-Viggars et al., 2015; Randall & Briscoe, 2018; Slade et al., 2020; Smith et al., 2019; Sorsa et al., 2021; Tobin et al., 2018
**4. Organisational**	
**4.1 Lack of services/Overstretched**	Bina, 2020; Button et al., 2017; Forde et al., 2020; Jones, 2019; Megnin-Viggars et al., 2015; Smith et al., 2019; Tobin et al., 2018; Viveiros & Darling, 2018
**4.2 Characteristics of service**	Bina, 2020; Button et al., 2017; Dennis & Chung-Lee, 2006; Forde et al., 2020; Jones, 2019; Newman et al., 2019; Scope et al., 2017; Viveiros & Darling, 2018; Watson et al., 2019

For peer review only - http://bmjopen.bmj.com/site/about/guidelines.xhtml

**4.3 Collaboration within and across services**	Bina, 2020; Megnin-Viggars et al., 2015; Newman et al., 2019; Smith et al., 2019; Watson et al., 2019
**4.4 Continuity of carer**	Brealey et al., 2010; Button et al., 2017; Hadfield & Wittkowski, 2017; Megnin-Viggars et al., 2015; Slade et al., 2020; Smith et al., 2019; Tobin et al., 2018; Viveiros & Darling, 2018; Watson et al., 2019
**4.5 Ideal care**	
**4.5.1 Screening**	
**4.5.1.1 Screening acceptability**	Brealey et al., 2010; Button et al., 2017; Evans et al., 2020; Hewitt et al., 2009; Megnin-Viggars et al., 2015; Mollard et al., 2016; Smith et al., 2019
**4.5.1.2 Wording/contents**	Brealey et al., 2010; Hewitt et al., 2009
**4.5.1.3 Delivery**	Brealey et al., 2010; Button et al., 2017; Evans et al., 2020; Hewitt et al., 2009; Smith et al., 2019
**4.5.2 Optimal treatment**	
**4.5.2.1 Opportunity to talk**	Dennis & Chung-Lee, 2006; Evans et al., 2020; Hadfield & Wittkowski, 2017; Jones, 2019; Kassam, 2019; Morrell et al., 2016; Praetorius et al., 2020; Staneva et al., 2015
**4.5.2.2 Location**	Hadfield & Wittkowski, 2017; Hansotte et al., 2017; Jones, 2019; Jones et al., 2014; Mollard et al., 2016; Newman et al., 2019; Praetorius et al., 2020; Sorsa et al., 2021
**4.5.2.3 Appropriate**	Evans et al., 2020; Megnin-Viggars et al., 2015; Scope et al., 2017; Sorsa et al., 2021
**4.5.2.4 Individualised**	Evans et al., 2020; Hadfield & Wittkowski, 2017; Megnin-Viggars et al., 2015; Morrell et al., 2016; Schmied et al., 2017; Scope et al., 2017; Slade et al., 2020; Sorsa et al., 2021; Viveiros & Darling, 2018; Watson et al., 2019
**4.5.2.5 Length**	Hadfield & Wittkowski, 2017; Morrell et al., 2016; Schmied et al., 2017; Watson et al., 2019
**4.5.2.6 Group/Peer support**	Dennis & Chung-Lee, 2006; Evans et al., 2020; Forde et al., 2020; Hadfield & Wittkowski, 2017; Jones et al., 2014; Megnin-Viggars et al., 2015; Morrell et al., 2016; Schmied et al., 2017; Scope et al., 2017; Slade et al., 2020; Tobin et al., 2018; Watson et al., 2019
**4.5.2.6.1 Validation provided by peer support**	Jones et al., 2014; Morrell et al., 2016; Schmied et al., 2017; Slade et al., 2020
**4.5.2.7 Culturally appropriate**	Button et al., 2017; Dennis & Chung-Lee, 2006; Giscombe et al., 2020; Hadfield & Wittkowski, 2017; Jones, 2019; Schmied et al., 2017; Smith et al., 2019; Tobin et al., 2018; Watson et al., 2019
**4.5.2.8 Information provision**	Forde et al., 2020; Hadfield & Wittkowski, 2017; A. Jones, 2019; Megnin-Viggars et al., 2015; Morrell et al., 2016; Schmied et al., 2017; Scope et al., 2017; Slade et al., 2020; Sorsa et al., 2021
**4.5.2.9 Medication**	Dennis & Chung-Lee, 2006; Forde et al., 2020; Jones, 2019; Sorsa et al., 2021; Tobin et al., 2018
**5. Political**	
**5.1 Immigration status**	Bina, 2020; Giscombe et al., 2020; Hansotte et al., 2017; Kassam, 2019; Lucas et al., 2019; Schmied et al., 2017

For peer review only - http://bmjopen.bmj.com/site/about/guidelines.xhtml

**5.2. Economic status**	Schmied et al., 2017; Tobin et al., 2018; Watson et al., 2019
**5.2.1 Healthcare costs**	Bina, 2020; Hansotte et al., 2017; Schmied et al., 2017; Tobin et al., 2018; Viveiros & Darling, 2018
**6. Societal**	
**6.1. Culture**	Bina, 2020; Brealey et al., 2010; Button et al., 2017; Dennis & Chung-Lee, 2006; Giscombe et al., 2020; Hansotte et al., 2017; Hewitt et al., 2009; Jones, 2019; Kassam, 2019; Megnin-Viggars et al., 2015; Nilaweera et al., 2014; Praetorius et al., 2020; Schmied et al., 2017; Smith et al., 2019; Sorsa et al., 2021; Staneva et al., 2015; Tobin et al., 2018; Viveiros & Darling, 2018; Watson et al., 2019; Wittkowski et al., 2014
**6.2. Maternal norms**	Bina, 2020; Brealey et al., 2010; Button et al., 2017; Dennis & Chung-Lee, 2006; Forde et al., 2020; Hadfield & Wittkowski, 2017; Hansotte et al., 2017; Hewitt et al., 2009; Holopainen & Hakulinen, 2019; Jones et al., 2014; Lucas et al., 2019; Mollard et al., 2016; Morrell et al., 2016; Newman et al., 2019; Schmied et al., 2017; Slade et al., 2020; Smith et al., 2019; Sorsa et al., 2021; Staneva et al., 2015; Viveiros & Darling, 2018
**6.3. Stigma**	Bina, 2020; Button et al., 2017; Dennis & Chung-Lee, 2006; Forde et al., 2020; Giscombe et al., 2020; Hadfield & Wittkowski, 2017; Hansotte et al., 2017; Hewitt et al., 2009; Holopainen & Hakulinen, 2019; Jones, 2019; Kassam, 2019; Lucas et al., 2019; Megnin-Viggars et al., 2015; Morrell et al., 2016; Nilaweera et al., 2014; Scope et al., 2017; Smith et al., 2019; Sorsa et al., 2021; Staneva et al., 2015; Tobin et al., 2018; Viveiros & Darling, 2018; Watson et al., 2019

*Note: highlighted yellow are themes only represented by lower-quality studies

For peer review only

References

1. Curry SJ, Krist AH, Owens DK, et al. Interventions to Prevent Perinatal Depression: US Preventive Services Task Force Recommendation Statement. *JAMA*. 2019;321(6):580-587. doi:10.1001/JAMA.2019.0007

2. What is the CPMHC? – CANADIAN PERINATAL MENTAL HEALTH COLLABORATIVE / COLLECTIF CANADIEN POUR LA SANTÉ MENTALE PÉRINATALE. Accessed April 12, 2022. https://cpmhc.ca/what-is-the-cpmhc/

3. PMHP - Perinatal Mental Health Project. Accessed April 12, 2022. https://pmhp.za.org/

4. Perinatal Anxiety & Depression Australia (PANDA). *Submission to the Productivity Commission Mental Health Inquiry: The Social and Economic Benefits of Improving Perinatal Mental Health*.; 2019.

5. National Institute for Health and Care Excellence (NICE). *Antenatal and Postnatal Mental Health Clinical Management and Service*.; 2014.

6. Moher D, Liberati A, Tetzlaff JM, Altman DG. Preferred reporting items for systematic reviews and meta-analyses: the PRISMA statement. *PLoS Med*. 2009;6(7):e1000097. doi:10.1016/j.jclinepi.2009.06.005

7. The Cochrane Collaboration. Cochrane data collection form for intervention reviews for RCTs only. *The cochrane Collaboration*. 2014;(April):2014. http://www.cochrane.org/

8. Shea BJ, Reeves BC, Wells GA, et al. AMSTAR 2: A critical appraisal tool for systematic reviews that include randomised or non-randomised studies of healthcare interventions, or both. *BMJ*. 2017;21:358. doi:10.1136/bmj.j4008

9. Harden A. *"Qualitative" Research, Systematic Reviews, and Evidence-Informed Policy and Practice*. University of London; 2007.

10. Carroll C, Booth A, Lloyd-Jones M. Should we exclude inadequately reported studies from qualitative systematic reviews? An evaluation of sensitivity analyses in two case study reviews. *Qualitative Health Research*. 2012;22(10):1425-1434.

11. Bina R. Predictors of postpartum depression service use: A theory-informed, integrative systematic review. *Women and Birth*. 2020;33(1):e24-e32. doi:10.1016/j.wombi.2019.01.006

12. Hansotte E, Payne SI, Babich SM. Positive postpartum depression screening practices and subsequent mental health treatment for low-income women in Western countries: A systematic literature review. *Public Health Reviews*. 2017;38(1):1-17. doi:10.1186/s40985-017-0050-y

13. Geertz C. *The Interpretation of Cultures: Selected Essays*. Basic Books; 1973.

14. Hadfield H, Wittkowski A. Women's Experiences of Seeking and Receiving Psychological and Psychosocial Interventions for Postpartum Depression: A Systematic Review and Thematic Synthesis of the Qualitative Literature. *Journal of Midwifery and Women's Health*. 2017;62(6):723-736. doi:10.1111/jmwh.12669

15. Popay J, Rogers A, Williams G. Rationale and standards for the systematic review of qualitative literature in health services research. *Qualitative Health Research*. 1998;8(3):341-351. doi:10.1177/104973239800800305

For peer review only - http://bmjopen.bmj.com/site/about/guidelines.xhtml

16. Thomas J, Harden A. Methods for the thematic synthesis of qualitative research in systematic reviews. *BMC Medical Research Methodology*. 2008;8(1):1-10. doi:10.1186/1471-2288-8-45

17. Ferlie EB, Shortell SM. Improving the Quality of Health Care in the United Kingdom and the United States: A Framework for Change. *Milbank Quarterly*. 2001;79(2):281-315. doi:10.1111/1468-0009.00206

18. Brealey SD, Hewitt C, Green JM, Morrell J, Gilbody S. Screening for postnatal depression - is it acceptable to women and healthcare professionals? A systematic review and meta-synthesis. *Journal of Reproductive and Infant Psychology*. 2010;28(4):328-344. doi:10.1080/02646838.2010.513045

19. Button S, Thornton A, Lee S, Ayers S, Shakespeare J. Seeking help for perinatal psychological distress: A meta-synthesis of women's experiences. *British Journal of General Practice*. 2017;67(663):e692-e699. doi:10.3399/bjgp17X692549

20. Dennis CL, Chung-Lee L. Postpartum depression help-seeking barriers and maternal treatment preferences: A qualitative systematic review. *Birth*. 2006;33(4):323-331. doi:10.1111/j.1523-536X.2006.00130.x

21. Evans K, Spiby H, Morrell J. Non-pharmacological interventions to reduce the symptoms of mild to moderate anxiety in pregnant women. A systematic review and narrative synthesis of women's views on the acceptability of and satisfaction with interventions. *Archives of Women's Mental Health*. 2020;23:11-28. doi:10.1007/s00737-018-0936-9

22. Giscombe T, Hui A, Stickley T. Perinatal mental health amongst refugee and asylum-seeking women in the UK. *Mental Health Review Journal*. 2020;25(3).

23. Hewitt CE, Gilbody S, Brealey SD, et al. Methods to identify postnatal depression in primary care: An integrated evidence synthesis and value of information analysis. *Health Technology Assessment*. 2009;13(36):1-145. doi:10.3310/hta13360

24. Holopainen A, Hakulinen T. New parents' experiences of postpartum depression: A systematic review of qualitative evidence. *JBI Database of Systematic Reviews and Implementation Reports*. 2019;17(9):1731-1769. doi:10.11124/JBISRIR-2017-003909

25. Jones CCG, Jomeen J, Hayter M. The impact of peer support in the context of perinatal mental illness: A meta-ethnography. *Midwifery*. 2014;30(5):491-498. doi:10.1016/j.midw.2013.08.003

26. Jones A. Help seeking in the perinatal period: a review of barriers and facilitators. *Soc Work Public Health*. 2019;34(7):596-605.

27. Kassam S. Understanding Experiences of Social Support as Coping Resources among Immigrant and Refugee Women with Postpartum Depression: An Integrative Literature Review. *Issues in Mental Health Nursing*. 2019;40(12):999-1011. doi:10.1080/01612840.2019.1585493

28. Lucas G, Olander EK, Ayers S, Salmon D. No straight lines - Young women's perceptions of their mental health and wellbeing during and after pregnancy: A systematic review and meta-ethnography. *BMC Women's Health*. 2019;19(1):1-17. doi:10.1186/s12905-019-0848-5

29. Megnin-Viggars O, Symington I, Howard LM, Pilling S. Experience of care for mental health problems in the antenatal or postnatal period for women in the UK: a systematic review and

For peer review only

meta-synthesis of qualitative research. *Archives of Women's Mental Health*. 2015;18(6):245-759. doi:10.1007/s00737-015-0548-6

30. Mollard E, Hudson DB, Ford A, Pullen C. An Integrative Review of Postpartum Depression in Rural U.S. Communities. *Archives of Psychiatric Nursing*. 2016;30(3):418-424. doi:10.1016/j.apnu.2015.12.003

31. Morrell J, Sutcliffe P, Booth A, et al. A systematic review, evidence synthesis and meta-analysis of quantitative and qualitative studies evaluating the clinical effectiveness, the cost-effectiveness, safety and acceptability of interventions to prevent postnatal depression. *Health Technology Assessment*. 2016;20(37). doi:10.3310/hta20370

32. Newman TC, Hirst J, Darwin Z. What enables or prevents women with depressive symptoms seeking help in the postnatal period? *British Journal of Midwifery*. 2019;27(4):219-227. doi:10.12968/bjom.2019.27.4.219

33. Nilaweera I, Doran F, Fisher J. Prevalence, nature and determinants of postpartum mental health problems among women who have migrated from South Asian to high-income countries: A systematic review of the evidence. *Journal of Affective Disorders*. 2014;166:213-226. doi:10.1016/j.jad.2014.05.021

34. Praetorius R, Maxwell D, Alam K. Wearing a happy mask: mother's expressions of suicidality with postpartum depression. *Social Work in Mental Health*. 2020;18(4):429-459.

35. Randall L, Briscoe L. Are women empowered to make decisions about the use of antidepressants in pregnancy? *British Journal of Midwifery*. 2018;26(5):329-335. doi:10.12968/bjom.2018.26.5.329

36. Sambrook Smith M, Lawrence V, Sadler E, Easter A. Barriers to accessing mental health services for women with perinatal mental illness: Systematic review and meta-synthesis of qualitative studies in the UK. *BMJ Open*. 2019;9(1):e024803. doi:10.1136/bmjopen-2018-024803

37. Schmied V, Black E, Naidoo N, Dahlen HG, Liamputtong P. Migrant women's experiences, meanings and ways of dealing with postnatal depression: A meta-ethnographic study. *PLoS ONE*. 2017;12(3). doi:10.1371/journal.pone.0172385

38. Scope A, Booth A, Morrell J, Sutcliffe P, Cantrell A. Perceptions and experiences of interventions to prevent postnatal depression. A systematic review and qualitative evidence synthesis. *Journal of Affective Disorders*. 2017;210:100-110. doi:10.1016/j.jad.2016.12.017

39. Slade P, Molyneux R, Watt A. A systematic review of clinical effectiveness of psychological interventions to reduce post traumatic stress symptoms following childbirth and a meta-synthesis of facilitators and barriers to uptake of psychological care. *J Affect Disord*. 2020;281:678-694.

40. Sorsa MA, Kylmä J, Bondas TE. Contemplating Help-Seeking in Perinatal Psychological Distress—A Meta-Ethnography. *Int J Environ Res Public Health*. 2021;18(10):5226.

41. Staneva AA, Bogossian F, Wittkowski A. The experience of psychological distress, depression, and anxiety during pregnancy: A meta-synthesis of qualitative research. *Midwifery*. 2015;31(6):563-573. doi:10.1016/j.midw.2015.03.015

For peer review only - http://bmjopen.bmj.com/site/about/guidelines.xhtml

42. Tobin CL, di Napoli P, Beck CT. Refugee and Immigrant Women's Experience of Postpartum Depression: A Meta-Synthesis. *J Transcult Nurs*. 2018;29(1):84-100. doi:10.1177/1043659616686167

43. Viveiros CJ, Darling EK. Perceptions of barriers to accessing perinatal mental health care in midwifery: A scoping review. *Midwifery*. 2019;70:106-118. doi:10.1016/j.midw.2018.11.011

44. Watson H, Harrop D, Walton E, Young A, Soltani H. A systematic review of ethnic minority women's experiences of perinatal mental health conditions and services in Europe. *PLoS ONE*. 2019;14(1):e0210587. doi:10.1371/journal.pone.0210587

45. Wittkowski A, Gardner PL, Bunton P, Edge D. Culturally determined risk factors for postnatal depression in Sub-Saharan Africa: A mixed method systematic review. *Journal of Affective Disorders*. 2014;163:115-124. doi:10.1016/j.jad.2013.12.028

46. Forde R, Peters S, Wittkowski A. Recovery from postpartum psychosis: a systematic review and metasynthesis of women's and families' experiences. *Archives of Women's Mental Health*. 2020;23(5):597-612. doi:10.1007/s00737-020-01025-z

For peer review only

For peer review only - http://bmjopen.bmj.com/site/about/guidelines.xhtml

PRISMA 2020 Checklist

Section and Topic	Item #	Checklist item	Location where item is reported
**TITLE**			
Title	1	Identify the report as a systematic review.	1
**ABSTRACT**			
Abstract	2	See the PRISMA 2020 for Abstracts checklist.	3
**INTRODUCTION**			
Rationale	3	Describe the rationale for the review in the context of existing knowledge.	5
Objectives	4	Provide an explicit statement of the objective(s) or question(s) the review addresses.	5
**METHODS**			
Eligibility criteria	5	Specify the inclusion and exclusion criteria for the review and how studies were grouped for the syntheses.	6 & Appendix
Information sources	6	Specify all databases, registers, websites, organisations, reference lists and other sources searched or consulted to identify studies. Specify the date when each source was last searched or consulted.	6 & Appendix
Search strategy	7	Present the full search strategies for all databases, registers and websites, including any filters and limits used.	Appendix
Selection process	8	Specify the methods used to decide whether a study met the inclusion criteria of the review, including how many reviewers screened each record and each report retrieved, whether they worked independently, and if applicable, details of automation tools used in the process.	6 & Appendix
Data collection process	9	Specify the methods used to collect data from reports, including how many reviewers collected data from each report, whether they worked independently, any processes for obtaining or confirming data from study investigators, and if applicable, details of automation tools used in the process.	6 & Appendix
Data items	10a	List and define all outcomes for which data were sought. Specify whether all results that were compatible with each outcome domain in each study were sought (e.g. for all measures, time points, analyses), and if not, the methods used to decide which results to collect.	6 & Appendix
	10b	List and define all other variables for which data were sought (e.g. participant and intervention characteristics, funding sources). Describe any assumptions made about any missing or unclear information.	6 & Appendix
Study risk of bias assessment	11	Specify the methods used to assess risk of bias in the included studies, including details of the tool(s) used, how many reviewers assessed each study and whether they worked independently, and if applicable, details of automation tools used in the process.	6-7 & Appendix
Effect measures	12	Specify for each outcome the effect measure(s) (e.g. risk ratio, mean difference) used in the synthesis or presentation of results.	N/A
Synthesis methods	13a	Describe the processes used to decide which studies were eligible for each synthesis (e.g. tabulating the study intervention characteristics and comparing against the planned groups for each synthesis (item #5)).	7
	13b	Describe any methods required to prepare the data for presentation or synthesis, such as handling of missing summary statistics, or data conversions.	7
	13c	Describe any methods used to tabulate or visually display results of individual studies and syntheses.	7
	13d	Describe any methods used to synthesize results and provide a rationale for the choice(s). If meta-analysis was performed, describe the model(s), method(s) to identify the presence and extent of statistical heterogeneity, and software package(s) used.	7
	13e	Describe any methods used to explore possible causes of heterogeneity among study results (e.g. subgroup analysis, meta-regression).	N/A
	13f	Describe any sensitivity analyses conducted to assess robustness of the synthesized results.	7 & Appendix
Reporting bias assessment	14	Describe any methods used to assess risk of bias due to missing results in a synthesis (arising from reporting biases).	7 & Appendix

For peer review only - http://bmjopen.bmj.com/site/about/guidelines.xhtml

PRISMA 2020 Checklist

Section and Topic	Item #	Checklist item	Location where item is reported
Certainty assessment	15	Describe any methods used to assess certainty (or confidence) in the body of evidence for an outcome.	7 & Appendix
**RESULTS**			
Study selection	16a	Describe the results of the search and selection process, from the number of records identified in the search to the number of studies included in the review, ideally using a flow diagram.	Figure 1
	16b	Cite studies that might appear to meet the inclusion criteria, but which were excluded, and explain why they were excluded.	Figure 1
Study characteristics	17	Cite each included study and present its characteristics.	7 & Appendix
Risk of bias in studies	18	Present assessments of risk of bias for each included study.	Appendix
Results of individual studies	19	For all outcomes, present, for each study: (a) summary statistics for each group (where appropriate) and (b) an effect estimate and its precision (e.g. confidence/credible interval), ideally using structured tables or plots.	N/A
Results of syntheses	20a	For each synthesis, briefly summarise the characteristics and risk of bias among contributing studies.	Appendix
	20b	Present results of all statistical syntheses conducted. If meta-analysis was done, present for each the summary estimate and its precision (e.g. confidence/credible interval) and measures of statistical heterogeneity. If comparing groups, describe the direction of the effect.	N/A
	20c	Present results of all investigations of possible causes of heterogeneity among study results.	N/A
	20d	Present results of all sensitivity analyses conducted to assess the robustness of the synthesized results.	Appendix
Reporting biases	21	Present assessments of risk of bias due to missing results (arising from reporting biases) for each synthesis assessed.	N/A
Certainty of evidence	22	Present assessments of certainty (or confidence) in the body of evidence for each outcome assessed.	Appendix
**DISCUSSION**			
Discussion	23a	Provide a general interpretation of the results in the context of other evidence.	18-21
	23b	Discuss any limitations of the evidence included in the review.	21
	23c	Discuss any limitations of the review processes used.	21
	23d	Discuss implications of the results for practice, policy, and future research.	21
**OTHER INFORMATION**			
Registration and protocol	24a	Provide registration information for the review, including register name and registration number, or state that the review was not registered.	4
	24b	Indicate where the review protocol can be accessed, or state that a protocol was not prepared.	4
	24c	Describe and explain any amendments to information provided at registration or in the protocol.	N/A
Support	25	Describe sources of financial or non-financial support for the review, and the role of the funders or sponsors in the review.	4
Competing interests	26	Declare any competing interests of review authors.	N/A
Availability of data, code and other materials	27	Report which of the following are publicly available and where they can be found: template data collection forms; data extracted from included studies; data used for all analyses; analytic code; any other materials used in the review.	Appendix

For peer review only

PRISMA 2020 Checklist

From: Page MJ, McKenzie JE, Bossuyt PM, Boutron I, Hoffmann TC, Mulrow CD, et al. The PRISMA 2020 statement: an updated guideline for reporting systematic reviews. BMJ 2021;372:n71. doi: 10.1136/bmj.n71

For more information, visit: http://www.prisma-statement.org/

For peer review only
